# A redox-enabled strategy for intramolecular hydroamination†

**DOI:** 10.1039/d2sc00481j

**Published:** 2022-05-30

**Authors:** Meredith A. Allen, Huy M. Ly, Geneviève F. O'Keefe, André M. Beauchemin

**Affiliations:** Centre for Catalysis Research and Innovation, Department of Chemistry and Biomolecular Sciences, University of Ottawa 10 Marie-Curie Ottawa ON K1N 6N5 Canada andre.beauchemin@uottawa.ca

## Abstract

Metal- or acid-catalyzed intramolecular hydroamination and Cope-type intramolecular hydroamination, a distinct concerted approach using hydroxylamines, typically suffer from significant synthetic limitations. Herein we report a process for intramolecular hydroamination that uses a redox-enabled strategy relying on efficient *in situ* generation of hydroxylamines by oxidation, followed by Cope-type hydroamination, then reduction of the resulting pyrrolidine *N*-oxide. The steps are performed sequentially in a single pot, no catalyst is required, the conditions are mild, the process is highly functional group tolerant, and no chromatography is generally required for isolation. A robustness screen and a gram-scale example further support the practicality of this approach.

## Abstract 2

A redox strategy enables hydroaminations: mild conditions allows efficient hydroxylamine formation & cyclization, then B_2_(OH)_4_ as reductant also facilitates isolation!

## Graphical abstract

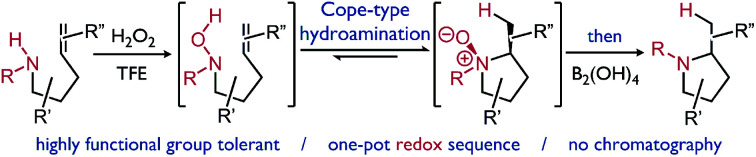

## Introduction

**Scheme 1:**
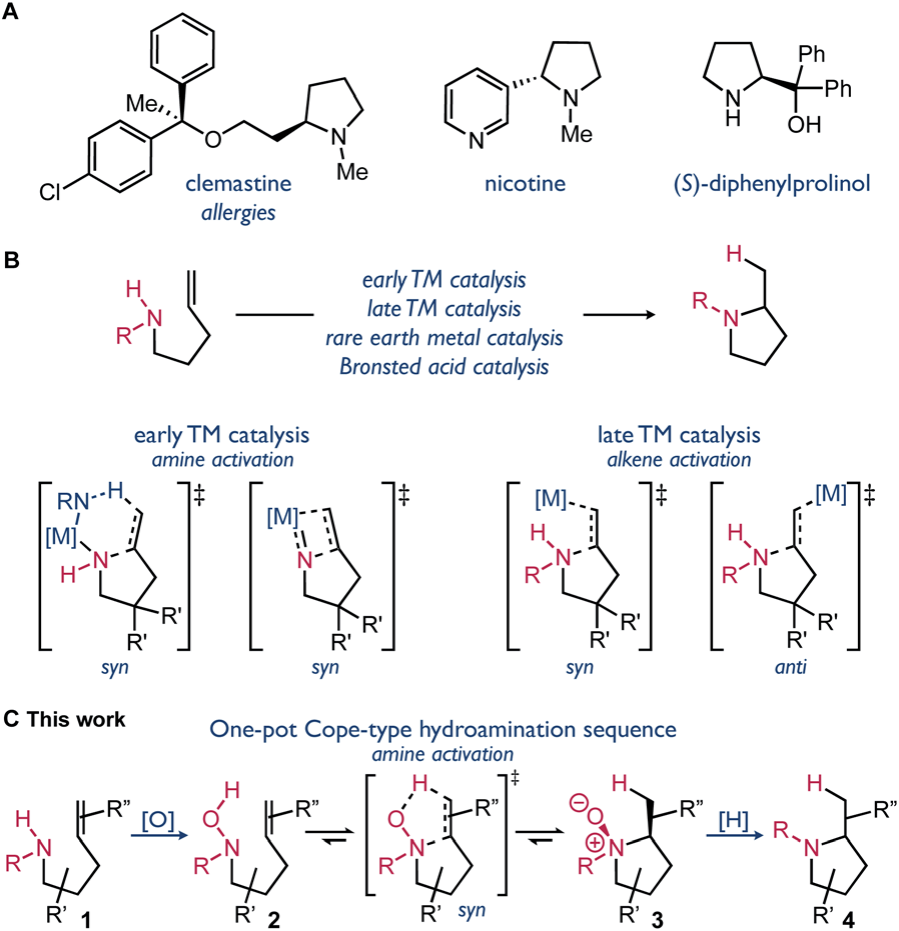
(A) Pyrrolidines in bioactive compounds and organocatalysts; (B) general activation modes for catalyzed intramolecular hydroamination; (C) this work – one-pot synthesis of pyrrolidines *via* redox-enabled hydroamination.

Amines are ubiquitous substructures within bioactive compounds. Cyclic amines, such as pyrrolidines and piperidines, are common motifs in pharmaceuticals and other fine chemicals (Scheme 1A).^1^ The syntheses of saturated cyclic amines can be accomplished using a variety of C–C or C–N bond forming reactions. Examples of efficient methods include nitrene C–H insertion, N-centered radical addition, and hydroamination reactions.^2^ Alkene hydroamination typically uses readily available reagents, amines and alkenes, and has high atom economy. Catalysis has often been required to overcome the high kinetic barrier of hydroamination reactions (Scheme 1B).^2*b*,3^ The use of metal-catalyzed processes is still often impeded by harsh reaction conditions, poor functional group tolerance, complex ligand synthesis, and the requirement for specific substitution.^4^

Cope-type hydroamination is a distinct strategy using oxidized amines, hydroxylamines, to form pyrrolidine *N*-oxides upon concerted cyclization.^5^ This reaction proceeds *via* a 5-membered transition state, resulting in a lower kinetic barrier for hydroamination. Cope-type hydroamination is a viable alternative to metal-catalyzed processes, however, it suffers from difficulties associated with the stability of the hydroxylamine reagent 2 and the isolation or reduction of the *N*-oxide product 3.^6^ This strategy for hydroamination has consequently been underappreciated.^7^ Herein we report the first redox-transfer enabled approach to hydroamination, applied to the synthesis of pyrrolidines. By using a synthetic sequence that avoids the isolation of the hydroxylamine and *N*-oxide intermediates (Scheme 1C), this methodology is designed to improve functional group tolerance and practicality relative to other intramolecular hydroamination methods.

## Results and discussion

After surveying the literature relating to oxidative hydroxylamine synthesis, it was clear that overoxidation would likely be a problem to address in the reaction optimization.^8^ We therefore focused on developing mild oxidation conditions that could also facilitate hydroamination. Inherently, rapid cyclization would prevent overoxidation of the hydroxylamine. Mild reduction conditions were also required to ensure functional group tolerance. Fortunately, several recent reports use boron-containing reagents for mild and selective reduction of *N*-oxides.^9^ With a longstanding interest in the Cope-type hydroamination field, we were also aware of the strong solvent effects for the equilibrium between hydroxylamine and *N*-oxide, with alcohol solvents often significantly benefitting reactivity.^10^ Using a urea hydrogen peroxide adduct (UHP) in 2,2,2-trifluoroethanol (TFE), we expected that efficient amine oxidation to the corresponding hydroxylamine could be achieved as inspired by analogous reactivity oxidizing sulfides and alkenes.^11^ Thus optimization began with the objective of achieving a one-pot reaction sequence that would avoid isolation of the intermediates (Table 1). Gratifyingly, efficient and selective oxidation was observed, hydroamination proceeded smoothly, and the following reduction occurred quantitatively using B_2_pin_2_ (Table 1, entry 1).

**Table 1:** Pyrrolidine synthesis optimization^a^

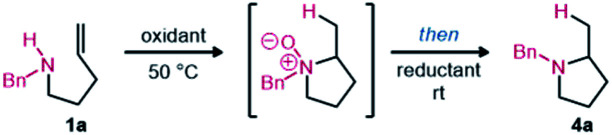				
Entry	Oxidant (equiv.)	Reductant	Solvent	Yield^b^ (%)
1	UHP (1.0)	B_2_pin_2_	TFE	71
2	UHP (1.2)	B_2_pin_2_	TFE	91
3	UHP (1.5)	B_2_pin_2_	TFE	70
4^c^	UHP (1.2)	B_2_pin_2_	TFE	0
5	30% aq. H_2_O_2_ (1.2)	B_2_pin_2_	TFE	84
6	UHP (1.2)	B_2_pin_2_	HFIP	70
7	UHP (1.2)	B_2_pin_2_	MeOH	30
8^d^	UHP (1.2)	B_2_pin_2_	TFE	33
9	UHP (1.2)	*o*-tolylB(OH)_2_	TFE	0
10^e^	UHP (1.2)	B_2_(OH)_4_	TFE	85^f^

a Conditions: amine 1a in solvent (0.1 M), then oxidant added, 50 °C, 16 h. Reductant then added (1.2 equiv.), rt, 30 min.

b ^1^H NMR yield of 4a using 1,3,5-trimethoxybenzene as an internal standard.

c B_2_pin_2_ and UHP added together.

d Reaction stirred at rt instead of 50 °C.

e Reduction step stirred at 50 °C, 1 h.

f Isolated yield. B_2_pin_2_: bis(pinacolato)diboron. HFIP: 1,1,1,3,3,3-hexafluoroisopropanol.

Using 1.2 equivalents of the oxidant and reductant proved optimal (Table 1, entries 1–3). Including the reductant along with the oxidant and amine in a single step procedure led to no desired product (entry 4). The boron reductant was instead selectively oxidized under the reaction conditions. These results therefore supported continuing optimization efforts using a one-pot, two-step procedure where amine oxidation and hydroamination occur separately from *N*-oxide reduction (see ESI, Tables S1–S4 for more details†). Modifying the oxidant to aqueous hydrogen peroxide had minimal effect on the overall yield (entry 5). The benefits of a good safety profile and minimal by-products of UHP (water) led us to continue using UHP for reaction optimization. Changing the solvent from TFE to other fluorinated or non-fluorinated alcohols led to reduced yields (entries 6 and 7). Lowering the reaction temperature led to an incomplete oxidation within the same reaction time (entry 8). Modifying the reductant to a less reactive, though widely available, *o*-tolylboronic acid led to no reduction (entry 9). Using hypodiboric acid as a reductant also led to a high yield (entry 10) and facilitated isolation. Indeed, the product 4a could be isolated in high purity after aqueous workup. Due to the simple isolation and better atom economy, hypodiboric acid was selected as the reductant.

**Scheme 2:**
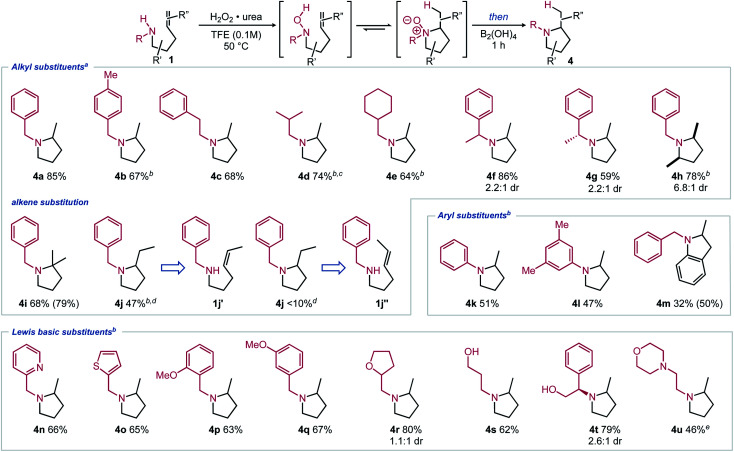
Scope of pyrrolidines 4 synthesized *via* redox-enabled hydroamination reaction sequence. Isolated yields shown with ^1^H NMR yields using 1,3,5-trimethoxybenzene as an internal standard shown in parentheses. (a) Conditions from Table 1, entry 10; (b) 2.2 equiv. of B_2_(OH)_4_ was added; (c) isolated as the HCl salt; (d) at 60 °C; (e) 2.2 equiv. of UHP and 3.2 equiv. of B_2_(OH)_4_ were added.

With an optimal one-pot procedure in hand (Table 1, entry 10), the scope of this hydroamination sequence was then explored. Given that carbon substitution has been thoroughly evaluated in prior Cope and metal-catalyzed intramolecular hydroamination reports, our efforts focused on varying nitrogen substitution.^2,3,5^ Excellent reactivity was observed for unbiased substrates with various *N*-alkyl substitution (Scheme 2, 4a–4g).^12^ Modest diastereoselectivity can be obtained when using chiral amines (4f–4g).^13^ This selectivity could possibly be elaborated into a chiral auxiliary-based route towards enantiopure α-substituted pyrrolidines. 2,5-Disubstituted pyrrolidine also formed in high yield under the reaction conditions with good *cis* diastereoselectivity (4h). Proximal alkene substitution, known to increase the rate of Cope-type hydroamination, had minimal effect on the product formation (4i). Conversely, terminal methyl substitution, known to decrease the rate of hydroamination, led to lower yields of the corresponding pyrrolidine under the optimized reaction conditions (4j).^5,14^ Notably, *trans*-substitution (1j′′) led to only trace product formation, with mostly degradation occurring, while *cis*-substituted amine 1jʹ formed a modest yield of pyrrolidine 4j*via* the two-step reaction sequence at 60 °C. Gratifyingly, aryl-substituted amines also underwent hydroamination (4k–4m). Aromatic substitution is largely missing from the Cope-type hydroamination literature, potentially due to the reduced nucleophilicity of aniline nitrogen atoms and the propensity of arylhydroxylamines to dimerize.^15^

Substrates including Lewis basic functional groups were well tolerated in contrast to related examples from metal-catalyzed processes (4n–4u).^16^ Products with heterocyclic substituents (4n–4o), alcohols and ethers (4p–4t) were isolated in good yields under modified reaction conditions. When an alkylamine substituent was included (1u), excess oxidant could be used to oxidize both nitrogen atoms, then complete reduction could occur using excess reductant, forming the corresponding pyrrolidine in an adequate yield (4u).

**Scheme 3:**
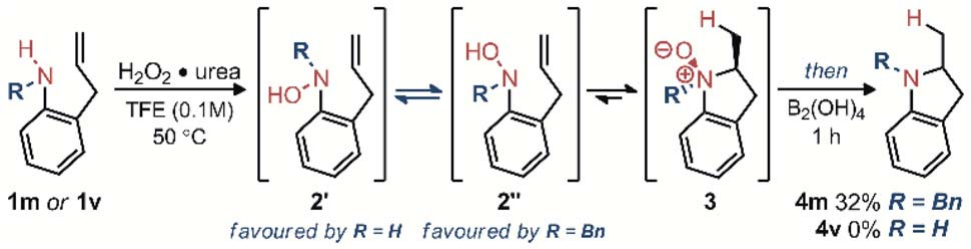
Conformational effects for aniline reagents towards the synthesis of indolines.

When 2-allylanilines (1m, 1v) were used as reagents for this reaction sequence, significant conformational effects were observed (Scheme 3). While mono-substituted aniline 1v formed no indoline upon oxidation to the corresponding hydroxylamine, the *N*-benzyl variant 1m formed a modest yield of pyrrolidine (4m). These results can be rationalized by considering the equilibrium of sp^2^-N hindered rotation of the R *vs.* OH substituents of the hydroxylamine intermediate. While the *N*-benzylhydroxylamine (2m) favours the reactive conformer 2′′, the mono-substituted hydroxylamine variant (2v) should favour the unreactive conformer 2ʹ, leading to no observed hydroamination reaction products.

Overall, this substrate scope is largely composed of products with nitrogen substitution not present in the Cope-type or transition metal-catalyzed intramolecular hydroamination literature. Intramolecular Cope-type hydroamination has historically been limited by difficult hydroxylamine reagent synthesis, leading to examples largely limited to methyl and benzyl nitrogen substitution.^5,6^ Transition metal catalysis does not suffer from this issue, however, few reported examples use Lewis basic alkylamines or other Lewis basic atoms.^16^ Additionally, geminal dialkyl substituents are common to affect a Thorpe–Ingold bias to promote cyclization. The reported redox-enabled Cope-type intramolecular hydroamination methodology is significantly less limited by substitution requirements. Additionally, most pyrrolidines could be isolated in high purity using this methodology without using chromatography (see ESI for more details†).

**Scheme 4:**
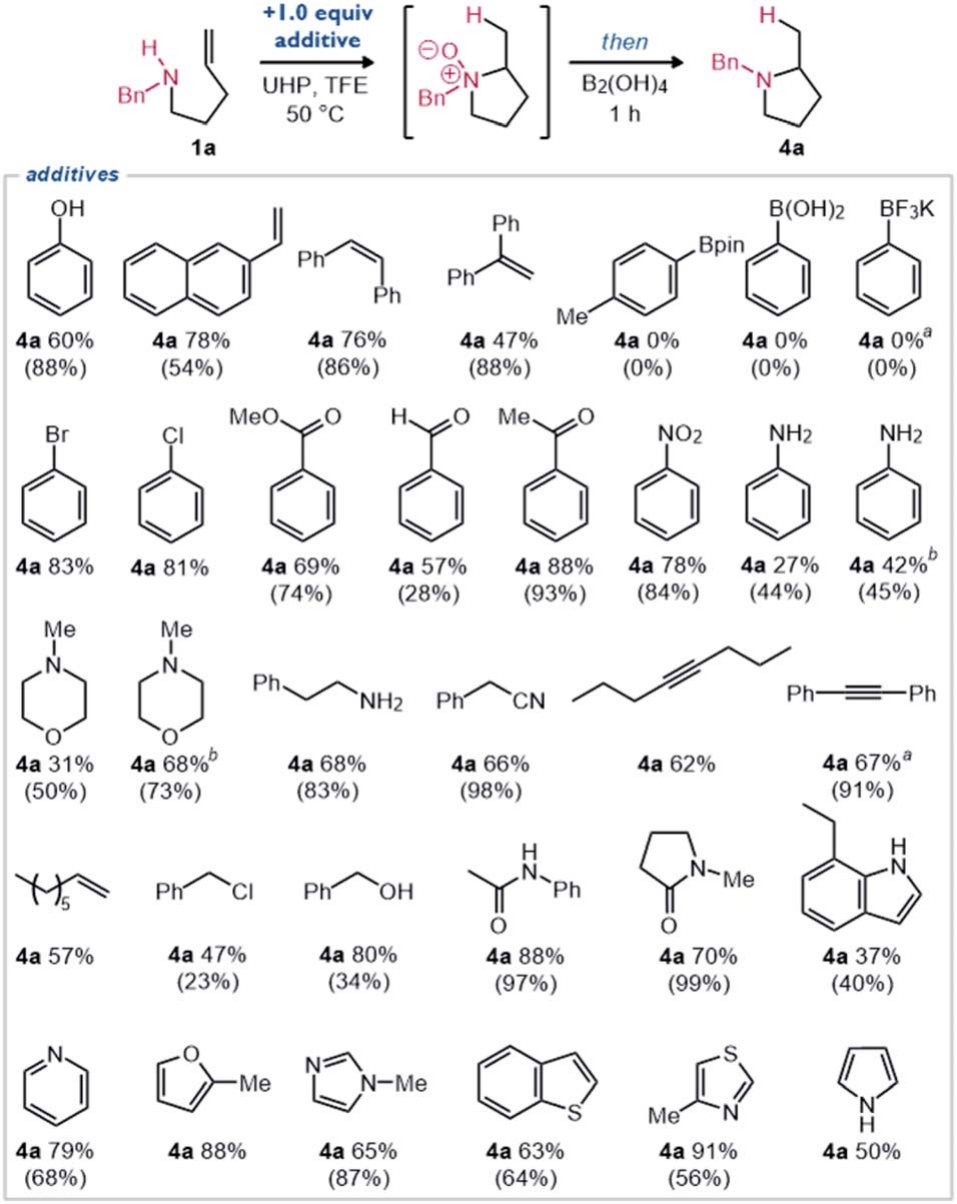
Robustness screen examining functional group compatibility. The standard reaction is undertaken in the presence of one molar equivalent of the given additive. The yield of 4a is given as a ^1^H NMR yield using 1,3,5-trimethoxybenzene as an internal standard (additive remaining in parentheses – value not included for volatile additives). (a) Partial solubility of additive; (b) 2.2 equiv. of UHP and B_2_(OH)_4_ were added.

To further evaluate the functional group tolerance of this hydroamination methodology, a robustness screen was performed (Scheme 4).^17^ Using the optimized reaction conditions with amine 1a (Table 1, entry 10), we measured the yield of pyrrolidine 4a when including 1.0 equivalents of various additives. Minimal changes were observed when phenol or aryl alkenes were added. Aryl boronic acid, ester, and trifluoroborate salt were not tolerated, as boron was completely oxidized under the reaction conditions. The inclusion of aryl halides, carbonyl-containing functional groups, and nitro groups generally led to no change in the yield of observed product. The inclusion of aniline or a secondary amine led to minimal observed product. Although, when the equivalents of oxidant and reductant were increased to 2.2, the product was again observed in higher yields. As similarly observed for the morpholine-substituted amine substrate (Scheme 2, 1u), we found that when both nitrogen atoms could be oxidized, the reaction proceeded smoothly, then global reduction afforded pyrrolidine 4a efficiently. This entry served to help validate the procedure for this robustness screen to accurately predict functional group tolerance. Primary amines, nitriles, alkynes, alkenes, alkyl chlorides, alcohols, and amides were generally well-tolerated functional groups. Various nitrogen, oxygen and sulfur-containing heterocycles were also well-tolerated. Reduced yields were observed when *N*-oxidation (*e.g.*, aniline and *N*-methylmorpholine), *N*-alkylation (*e.g.*, benzyl chloride), or other oxidation (*e.g.*, pyrrole, indole) could occur readily. Overall, these reaction conditions were observed to be highly robust as demonstrated by generally high product yields even in the presence of many common functional groups. These results demonstrate that this redox-enabled strategy for hydroamination is quite general, practical, and highly functional group tolerant.

Finally, this hydroamination methodology was evaluated on a gram scale using amine 1a. The resulting pyrrolidine 4a was still isolated in good yield after an aqueous workup (eqn (1)). The HCl salt could also easily be formed to produce a solid product.1
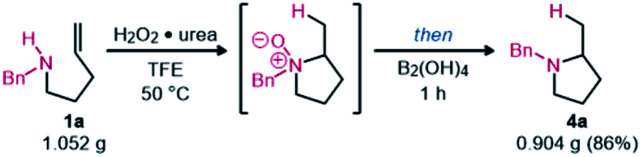


## Conclusions

In summary, secondary amines can undergo an efficient metal-free hydroamination to form pyrrolidines *via* a redox-enabled approach, featuring a concerted hydroamination reaction. Importantly, the undesirable issues associated with the synthesis and isolation of hydroxylamines and *N*-oxides are avoided using a one-pot sequence relying on new conditions to selectively oxidize secondary amines. The reaction conditions are highly functional group tolerant, as additionally demonstrated by a robustness screen, in contrast to many related transition metal-catalyzed hydroamination variants. Diastereoselective examples and gram scale reactivity are also reported. Overall, this work provides an improved method to form pyrrolidines and illustrates a practical redox-enabled process applied to hydroamination. While this stoichiometric process follows many of the principles of green chemistry due to the high atom economy, low reagent toxicity, and chromatography-free isolation, a catalytic oxygen transfer process is currently under investigation and will be reported in due course.

## Data availability

All experimental data, including additional reaction optimization data and detailed experimental procedures, is available in the ESI.†

## Author contributions

M. A. Allen and A. M. Beauchemin conceived the project. The reaction conditions were optimized by M. A. Allen and G. F. O'Keefe, and the purification procedure was determined by M. A. Allen and H. M. Ly. Substrates were synthesized by M. A. Allen, H. M. Ly and G. F. O'Keefe. The scope reported and robustness screen data were obtained by M. A. Allen and H. M. Ly. M. A. Allen wrote the manuscript with input from all authors and edited it with A. M. Beauchemin.

## Conflicts of interest

There are no conflicts to declare.

## Supplementary Material

SC-013-D2SC00481J-s001
