# Supplementary material for: A redox-enabled strategy for intramolecular hydroamination
Source: Chem Sci. 2022 May 30;13(24):7264–8. doi: 10.1039/d2sc00481j (PMC9214914; doi:10.1039/d2sc00481j)
Supplement: SC-013-D2SC00481J-s001 [file SC-013-D2SC00481J-s001.pdf]

Electronic Supplementary Material (ESI) for Chemical Science.
This journal is © The Royal Society of Chemistry 2022

Table of contents

General information…………………………………………………………………….. 1
Materials………………………………………………………………………………… 1
Additional optimization data (Tables **S1 – S4**)……………………………………… 2
General procedures……………………………………………………………………. 5
Characterization data……………………………………………………………………... 6
Gram–scale reaction procedure and characterization………………………………… 35
NMR spectra…………………………………………………………………………. 36

General information

Purification of reaction products was carried out by flash column chromatography using silica gel (40 – 63 µm), unless otherwise noted. Analytical thin layer chromatography (TLC) was performed on aluminum or glass, cut to size. Visualization was accomplished with UV light followed by staining with a potassium permanganate or ninhydrin solution and heating. ^{1}H NMR and ^{13}C NMR spectra were recorded on Bruker AVANCE 300 MHz, 400 MHz, and 500 MHz spectrometers at ambient temperature, unless otherwise indicated. Spectral data was reported in ppm using solvent as the reference ($CDCl_3$ at 7.26 ppm, CD_3OD at 3.31 ppm, or benzene-d_6 at 7.16 ppm for ^{1}H NMR and $CDCl_3$ at 77.0 ppm or CD_3OD at 49.0 ppm for ^{13}C NMR). ^{1}H NMR data was reported as: multiplicity (ap = apparent, br = broad, s = singlet, d = doublet, t = triplet, q = quartet, quint = quintet, sext = sextuplet, sept = septuplet, m = multiplet), integration and coupling constant(s) in Hz. ^{13}C NMR is reported indicating information from distortionless enhancement by polarization Transfer (DEPT) experiments. Infrared (IR) spectra were obtained on an Attenuated Total Reflectance Fourier Transform Infrared spectrometer (ATR – FTIR). High – resolution mass spectroscopy (HRMS) was performed on a mass spectrometer with an electron beam of 70 eV (EI) or Micromass Q-TOF I - Time of flight Electrospray Ionisation mass spectrometer (ESI).

Materials

Unless otherwise noted, all commercially available materials were purchased from commercial sources and used without further purification.

Additional optimization data

*Original optimization of the reported hydroamination reaction sequence used tertiary amine **S5a** to prevent over-oxidation of the amine. The resulting N-oxide **S6a** can undergo Cope elimination of acrylonitrile to yield hydroxylamine **2a**. A stronger oxidant, mCPBA, can be used for tertiary amine reagents since over-oxidation is not an issue. There are different chemoselectivity issues for the reduction step of this sequence when using this approach (N-oxide **S6a** vs. **3a**), and therefore different reductants were also optimal for this approach vs. the secondary amine approach reported.*

Table S1: Optimization of hydroamination cascade from tertiary amines[a]

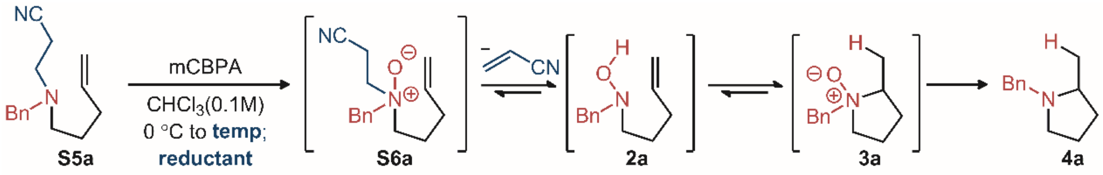

Entry	Equiv amine	Equiv mCPBA	Boron Reductant (equiv)	Temp (°C)	Yield 3a (%)[b]	Yield 4a (%)[b]
**1**	1.1	1.0	none	70	75	0
**2**	1.1	1.0	B_2pin_2 (1.1)	70	0	60
**3**	1.1	1.0	none	50	93	0
**4**	1.1	1.0	B_2pin_2 (1.1)	50	0	34
**5**	1.1	1.0	$ArB(OH)_2$ **A** (1.1)	50	46	52
**6**	1.2	1.0	$ArB(OH)_2$ **A** (1.2)	50	44	52
**7**	1.0	1.1	$ArB(OH)_2$ **A** (1.1)	50	25	39
**8**	1.0	1.1	$ArB(OH)_2$ **A** (1.5)	50	17	45
**9**	1.0	1.5	$ArB(OH)_2$ **A** (1.5)	50	8	23

(a) Conditions: amine **1a** in $CHCl_3$ (0.1 M), then mCPBA added, stirred at 0 °C, 30 min. Reductant then added, stirred at given temperature, 4 h. (b) ^{1}H NMR yield of **4a** using 1,3,5-trimethoxybenzene as an internal standard. Bpin = B(pinacolato) = $B(O_2C_2(CH_3)_4)$

Boron reductants

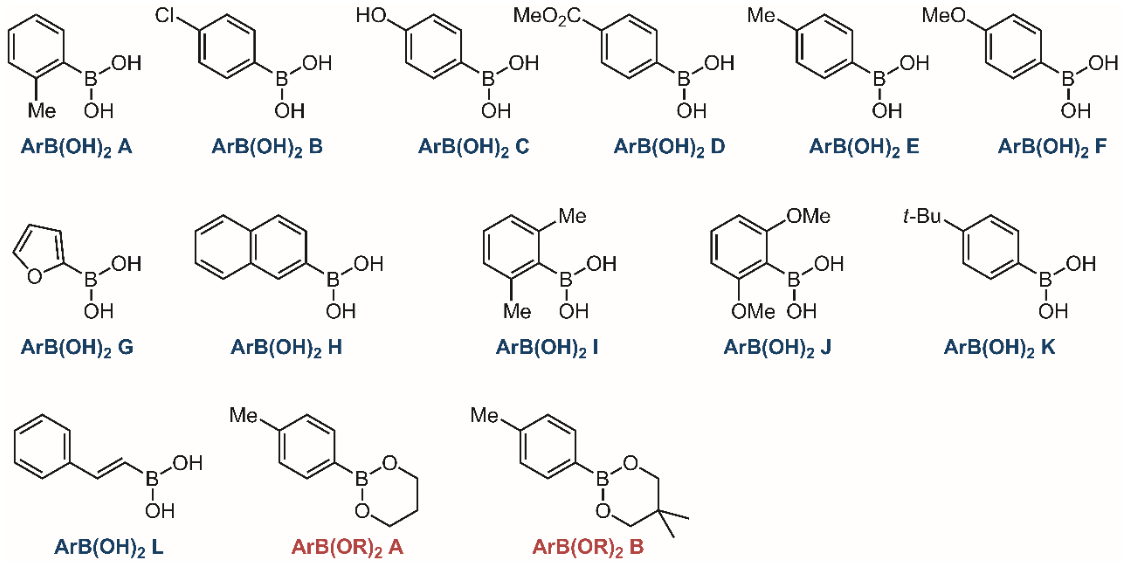

Table S2: Solvent scan for hydroamination using tertiary amine starting materials[a]

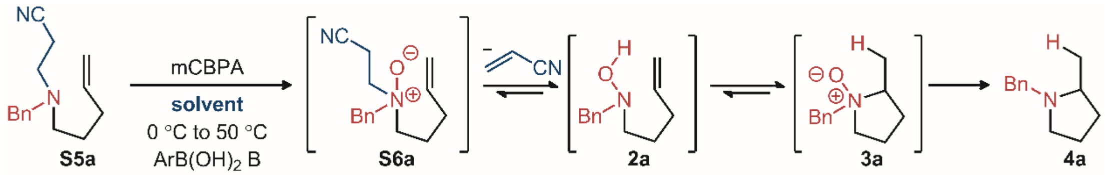

Entry	solvent	Yield 3a (%)[b]	Yield 4a (%)[b]
**1**	$CDCl_3$	40	42
**2**	MeCN	47	13
**3**	*t*-BuOH	6	trace
**4**	Cyclohexane	0	0
**5**	DCE	43	40
**6**	$PhCF_3$	34	23
**7**	Dioxane	22	19

(a) Conditions: amine **1a** (1.2 equiv) in solvent (0.1 M), then mCPBA (1.0 equiv) added, stirred at 0 °C, 30 min. 2-Methylphenyl boronic acid ($ArB(OH)_2$ **A**) then added (1.2 equiv), stirred at 50 °C, 4 h. (b) ^1H NMR yield of **4a** using 1,3,5-trimethoxybenzene as an internal standard.

Table S3: Boron reductant scan for hydroamination using tertiary amine staring materials[a]

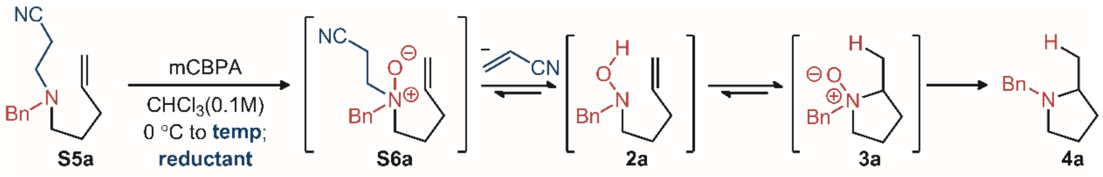

Entry	Boron Reductant	Temp (°C)	Time (h)	Yield 3a (%)[b]	Yield 4a (%)[b]
**1**	$B(C_6F_5)_3$	50	4	0	0
**2**	$B(C_6F_5)_3$	rt	4	0	13
**3**	$HOB(tol)_2$	50	4	0	8
**4**	$HOB(tol)_2$	rt	4	20	0
**5**	$Et_3SiBpin$	rt	4	12	20
**6**	$Et_3SiBpin$	50	4	30	43
**7**	$ArB(OR)_2$ **A**	50	24	100	trace
**8**	$ArB(OR)_2$ **B**	50	24	100	trace
**9**	$ArB(OH)_2$ **A**	50	4	44	52
**10**	$ArB(OH)_2$ **B**	50	4	73	22
**11**	$ArB(OH)_2$ **C**	50	24	100	trace
**12**	$ArB(OH)_2$ **D**	50	24	100	trace
**13**	$ArB(OH)_2$ **E**	50	4	75	25
**14**	$ArB(OH)_2$ **F**	50	4	83	10
**15**	$ArB(OH)_2$ **G**	50	4	95	0
**16**	$ArB(OH)_2$ **H**	50	4	55	36
**17**	$ArB(OH)_2$ **I**	50	4	69	19
**18**	$ArB(OH)_2$ **J**	50	4	98	trace
**19**	$ArB(OH)_2$ **K**	50	24	99	trace
**20**	$ArB(OH)_2$ **L**	50	24	58	30

(a) Conditions: amine **1a** (1.2 equiv) in $CHCl_3$ (0.1 M), then mCPBA (1.0 equiv) added, stirred at 0 °C, 30 min. Reductant then added (1.2 equiv), stirred at given temperature, 4 – 24 h. (b) ^{1}H NMR yield of **4a** using 1,3,5-trimethoxybenzene as an internal standard.

Table S4: Optimization of the oxidation and hydroamination of secondary amines[a]

Additional optimization data for the solvent and oxidant choice for the oxidation/Cope-type hydroamination steps of the reported reaction sequence are included. Diboron species (B_2pin_2 or $B_2(OH)_4$) were determined to be optimal reductants and were not evaluated within the following entries.

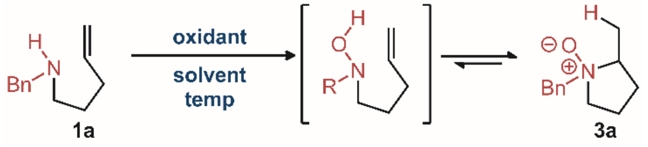

Entry	Solvent (M)	Oxidant (equiv)	Temp (°C)	Yield 3a (%)[b]
**1**	TFE (0.1)	UHP (1.2)	50	92
**2**	TFE (0.2)	UHP (1.2)	50	91
**3**	MeOH/HFIP 3:1 (0.1)	UHP (1.2)	50	52
**4**	MeOH/HFIP 1:1 (0.1)	UHP (1.2)	50	79
**5**	MeOH (0.1)	UHP (1.2)	50	30
**6**	EtOH (0.1)	UHP (1.2)	50	22
**7**	*i*-PrOH (0.1)	UHP (1.2)	50	16
**8**	*t*-BuOH (0.1)	UHP (1.2)	50	18
**9**	HFIP (0.1)	UHP (1.2)	50	70
**10**	*i*-PrOH/TFE 10:1 (0.1)	UHP (1.2)	50	27
**11**	*i*-PrOH/HFIP 10:1 (0.1)	UHP (1.2)	50	36
**12**	TFE (0.1)	30% aq. H_2O_2 (1.2)	50	42
**13**	TFE (0.1)	30% aq. H_2O_2 (1.5)	50	84
**14**	TFE (0.1)	H_2O_2 (1.5)	rt	62
**15**	TFE (0.1)	UHP (1.2)	rt	33

(a) Conditions: amine **1a** (1.0 equiv) in solvent, then oxidant added, stirred at given temperature, 16 h. (b) ^1H NMR yield of **3a** using 1,3,5-trimethoxybenzene as an internal standard. UHP = urea hydrogen peroxide adduct; TFE = 2,2,2-trifluoroethanol; HFIP = 1,1,1,3,3,3-hexafluoroisopropanol.

General procedures

General procedure A: Synthesis of secondary amines

To a round bottom flask was added 5-bromopent-1-ene (1.0 equiv) followed by dilution in EtOH (2.5 M). A primary amine (5.0 equiv) and NaI (0.05 equiv) were added, and the mixture was heated to reflux for 4 h. Upon completion, the reaction was concentrated via rotary evaporation. The reaction mixture was diluted with DCM and was extracted using 1M KOH (x1), then water (x1), and then brine (x1). The organic phase was dried with Na_2SO_4 and filtered. The filtrate was concentrated via rotary evaporation and the product was isolated using silica-gel flash column chromatography.

General procedure B: Redox–enabled hydroamination sequence

To a clean dry microwave vial was added the corresponding secondary amine **1** (1.0 equiv) followed by dilution with TFE (0.1 M). UHP was then added (1.2 equiv). The vial was sealed then stirred at 50 °C for 16 h. The reaction vessel was opened and $B_2(OH)_4$ (1.2 equiv for alkyl – substituted amines or 2.2 equiv for Lewis – base – substituted amines) was added. The vial was resealed then stirred at 50 °C for 1 h. The crude reaction mixture was concentrated via rotary evaporation, then diluted with DCM (50 mL) and water (50 mL). 10 mL of 1M HCl was added, and the phases were separated. 15 mL of 1M KOH was added to the aqueous phase, which was then extracted with DCM twice (50 mL each). The combined organic phases were washed with brine, dried over Na_2SO_4, then filtered before concentration via rotary evaporation. The crude product did not require any further purification.

General procedure C: Robustness screen[1]

To a clean dry microwave vial was added additive (0.1 mmol, 1.0 equiv), followed by amine **1a** (17.5 mg, 0.1 mmol) in a solution of TFE (0.1 M). UHP was then added (11.3 mg, 0.12 mmol). The vial was sealed then stirred at 50 °C for 16 h. The reaction vessel was opened and $B_2(OH)_4$ (10.8 mg, 0.12 mmol) was added. The vial was resealed then stirred at 50 °C for 1 h. The crude reaction mixture was concentrated via rotary evaporation, then 1,3,5-trimethoxybenzene (16.8 mg, 0.1 mmol) in a solution of $CDCl_3$ (0.2 M) was added to the crude reaction mixture. ^{1}H NMR spectra were obtained and the integration of diagnostic signals for the additive and for pyrrolidine **4a** were attained to find the amount (%) of both species. When the additive was volatile and no signals for this species could be observed, no amount remaining is reported. However, when the additive was somewhat volatile and signals for this species could be observed, likely in reduced quantities due to evaporation, the amount remaining is reported.

Characterization data

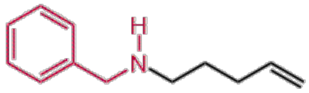

***N*-Benzylpent-4-en-1-amine 1a**
The title compound was synthesized according to **general procedure A** using 5-bromopent-1-ene (1.49 g, 10.0 mmol), benzylamine (5.50 mL, 50.0 mmol), sodium iodide (0.075 g, 0.05 mmol) in EtOH (2.5 M). The product was purified using silica-gel flash column chromatography (20% EtOAc/Hexanes to 40% EtOAc/Hexanes) to yield the title compound as a yellow oil (1.28 g, 73%). Characterization data is in good agreement with previously reported data.[2]

TLC R*f*: 0.52 in 10% MeOH/DCM

---

[1] K. D. Collins and F. Glorius, *Nat. Chem.* 2013, **5**, 597.
[2] Y.-H. Wang, J.-L. Ye, A.-E. Wang and P.-Q. Huang, *Org. Biomol. Chem.* 2012, **10**, 6504.

^{1}H NMR (500 MHz, $CDCl_3$): δ 7.39 – 7.18 (m, 5H), 5.82 (ddt, *J* = 16.9, 10.1, 6.6 Hz, 1H), 4.84 – 5.11 (m, 2H), 3.80 (s, 2H), 2.73 – 2.58 (m, 2H), 2.11 (dtt, *J* = 8.0, 6.6, 1.4 Hz, 2H), 1.63 (p, *J* = 7.4 Hz, 2H).

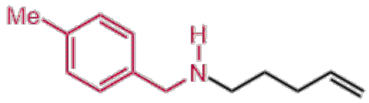

***N*-(4-Methylbenzyl)pent-4-en-1-amine 1b**
The title compound was synthesized according to **general procedure A** using 5-bromopent-1-ene (1.01 g, 6.80 mmol), *p*-tolylmethanamine (4.33 mL, 34.0 mmol), sodium iodide (0.102 g, 0.680 mmol) in EtOH (2.5 M). The product was purified using silica-gel flash column chromatography (20% EtOAc/Hexanes + 1% Et_3N) to yield the title compound as a yellow oil (1.09 g, 85%).

TLC R*f*: 0.28 in 30% EtOAc/Hexanes

^{1}H NMR (500 MHz, $CDCl_3$): δ 7.12 (m, 2H), 7.05 (m, 2H), 5.73 (ddt, *J* = 16.9, 10.2, 6.6 Hz, 1H), 4.93 (dq, *J* = 17.1, 1.7 Hz, 1H), 4.87 (ddt, *J* = 10.1, 2.2, 1.3 Hz, 1H), 3.66 (s, 2H), 2.60 – 2.52 (m, 2H), 2.25 (s, 3H), 2.06 – 1.97 (m, 2H), 1.52 (p, *J* = 7.3 Hz, 2H) , 1.21 (br s, 1H).

^{13}C NMR (125 MHz, $CDCl_3$): δ 138.6 (CH), 137.6 (C), 136.4 (C), 129.1 (CH), 128.1 (CH), 114.6 (CH_2), 53.8 (CH_2), 48.9 (CH_2), 31.6 (CH_2), 29.4 (CH_2), 21.1 (CH_3).

IR (FTIR): 3316, 3001, 2922, 1639, 1513, 1448, 1113, 908, 802 cm^{-1}.

HRMS (EI): Exact mass calcd for $C_{13}H_{19}N$ [M]+ 189.1517. Found: 189.1515.

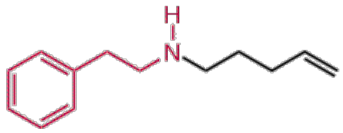

***N*-Phenethylpent-4-en-1-amine 1c**
The title compound was synthesized according to **general procedure A** using 5-bromopent-1-ene (2.51 g, 16.8 mmol), 2-phenylethane-1-amine (10.6 mL, 84.2 mmol), sodium iodide (0.252 g, 1.68 mmol) in EtOH (2.5 M). The product was purified using silica-gel flash column chromatography (20% EtOAc/Hexanes + 1% Et_3N) to yield the title compound as a yellow oil (0.59 g, 61%).

TLC R*f*: 0.43 in 10% MeOH/DCM

^{1}H NMR (500 MHz, $CDCl_3$): δ 7.29 – 7.15 (m, 2H), 7.18 – 7.09 (m, 3H), 5.72 (ddt, *J* = 16.9, 10.1, 6.7 Hz, 1H), 4.95 – 4.88 (m, 1H), 4.86 (ddt, *J* = 10.2, 2.2, 1.2 Hz, 1H), 2.85 – 2.68 (m, 4H), 2.62 – 2.50 (m, 2H), 2.04 – 1.91 (m, 2H), 1.49 (p, *J* = 7.4 Hz, 2H), 1.26 (s, 1H).

^{13}C NMR (125 MHz, $CDCl_3$): δ 140.1 (C), 138.4 (CH), 128.7 (CH), 128.5 (CH), 126.2 (CH), 114.67 (CH_2), 51.2 (CH_2), 49.3 (CH_2), 36.4 (CH_2), 31.6 (CH_2), 29.2 (CH_2).

IR (FTIR): 3030, 3021, 2917, 2801, 1636, 1464, 1451, 1126, 906, 742, 696 cm^{-1}.

HRMS (EI): Exact mass calcd for $C_{13}H_{19}N$ [M]+ 189.1517. Found: 189.1535.

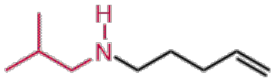

***N*-Isobutylpent-4-en-1-amine 1d**
The title compound was synthesized according to **general procedure A** using 5-bromopent-1-ene (1.01 g, 6.80 mmol), isobutylamine (3.38 mL, 34.0 mmol), sodium iodide (0.102 g, 0.680 mmol) in EtOH (2.5 M). The product was purified using silica-gel flash column chromatography (20% EtOAc/Hexanes + 1% Et_3N) to yield the title compound as a yellow oil (0.59 g, 61%).

TLC R*f*: 0.30 in 30% EtOAc/Hexanes

^{1}H NMR (500 MHz, $CDCl_3$): δ 5.82 – 5.70 (m, 1H), 4.95 (dq, *J* = 17.1, 1.6 Hz, 1H), 4.88 (ddt, *J* = 10.1, 2.3, 1.3 Hz, 1H), 2.53 (td, *J* = 7.3, 1.9 Hz, 2H), 2.34 (dt, *J* = 6.8, 1.3 Hz, 2H), 2.07 – 1.98 (m, 2H), 1.68 (dpd, *J* = 13.4, 6.7, 1.7 Hz, 1H), 1.52 (pd, *J* = 7.4, 1.8 Hz, 2H), 1.31 (br s, 1H), 0.83 (dt, *J* = 6.5, 1.3 Hz, 6H).

^{13}C NMR (125 MHz, $CDCl_3$): δ 138.6 (CH), 114.6 (CH_2), 58.1 (CH_2), 49.6 (CH_2), 31.6 (CH_2), 29.3 (CH_2), 28.3 (CH), 20.7 (CH_3).

IR (FTIR): 3325, 3074, 2952, 2869, 1640, 1465, 1126, 990, 908, 734 cm^{-1}.

HRMS (EI): Exact mass calcd for $C_9H_{18}N$ [M – H]+ 140.1439. Found: 140.1432.

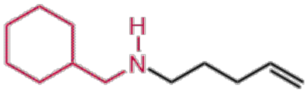

***N*-(Cyclohexylmethyl)pent-4-en-1-amine 1e**
The title compound was synthesized according to **general procedure A** using 5-bromopent-1-ene (1.49 g, 10.0 mmol), cyclohexylmethanamine (6.51 mL, 50.0 mmol), sodium iodide (0.150 g, 1.00 mmol) in EtOH (2.5 M). The product was purified using silica-gel flash column chromatography (80% EtOAc/Hexanes) to yield the title compound as a yellow oil (1.75 g, 96%).

TLC R*f*: 0.33 in 30% EtOAc/Hexanes

^1H NMR (500 MHz, $CDCl_3$): δ 5.75 (ddt, *J* = 16.9, 10.2, 6.6 Hz, 1H), 4.95 (dq, *J* = 17.1, 1.7 Hz, 1H), 4.88 (ddt, *J* = 10.2, 2.3, 1.3 Hz, 1H), 2.56 – 2.49 (m, 2H), 2.36 (d, *J* = 6.7 Hz, 2H), 2.06 – 1.98 (m, 2H), 1.71 – 1.55 (m, 5H), 1.52 (p, *J* = 7.4 Hz, 2H), 1.44 – 1.32 (m, 1H), 1.25 – 1.01 (m, 4H), 0.83 (qd, *J* = 12.5, 3.8 Hz, 2H).

^{13}C NMR (125 MHz, $CDCl_3$): δ 138.6 (CH), 114.6 (CH_2), 56.9 (CH_2), 49.7 (CH_2), 38.0 (CH), 31.7 (CH_2), 31.5 (CH_2), 29.3 (CH_2), 26.7 (CH_2), 26.1 (CH_2).

IR (FTIR): 3081, 2919, 2848, 1639, 1445, 1127, 909 cm^{-1}.

HRMS (ESI): Exact mass calcd for $C_{12}H_{24}N$ [M+H]+ 182.1909. Found: 182.1903.

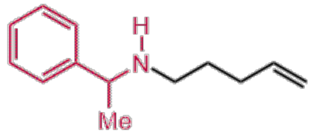

***N*-(1-Phenylethyl)pent-4-en-1-amine 1f**

The title compound was synthesized according to **general procedure A** using 5-bromopent-1-ene (1.49 g, 10.0 mmol), 1-phenylethanamine (6.40 mL, 50.0 mmol), sodium iodide (0.075 g, 0.50 mmol) in EtOH (2.5 M). The product was purified using silica-gel flash column chromatography (60% EtOAc/Hexanes) to yield the title compound as a yellow oil (1.76 g, 93%).

TLC R*f*: 0.37 in 60% EtOAc/Hexanes

^1H NMR (400 MHz, $CDCl_3$): δ 7.37 – 7.28 (m, 4H), 7.26 – 7.20 (m, 1H), 5.78 (ddt, *J* = 16.9, 10.2, 6.6 Hz, 1H), 4.98 (ap dq, *J* = 17.2, 1.7 Hz, 1H), 4.92 (ddt, *J* = 10.2, 2.2, 1.3 Hz, 1H), 3.75 (q, *J* = 6.6 Hz, 1H), 2.52 (ddd, *J* = 11.4, 7.9, 6.3 Hz, 1H), 2.43 (ddd, *J* = 11.4, 7.9, 6.8 Hz, 1H), 2.05 (dddt, *J* = 10.8, 5.6, 2.8, 1.4 Hz, 2H), 1.64 – 1.47 (m, 2H), 1.35 (d, *J* = 6.6 Hz, 3H).

^{13}C NMR (100 MHz, $CDCl_3$): δ 145.8 (C), 138.5 (CH), 128.4 (CH), 126.8 (CH), 126.5 (CH), 114.5 (CH_2), 58.3 (CH), 47.3 (CH_2), 31.6 (CH_2), 29.4 (CH_2), 24.4 (CH_3).

IR (FTIR): 3027, 3017, 2956, 2931, 2805, 1637, 1456, 1447, 1132, 910, 761, 697 cm^{-1}.

HRMS (EI): Exact mass calcd for $C_{13}H_{18}N$ [M-H]+ 188.1439. Found: 188.1449.

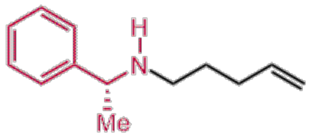

(*R*)-*N*-(1-Phenylethyl)pent-4-en-1-amine 1g

The title compound was synthesized according to **general procedure A** using 5-bromopent-1-ene (0.745 g, 5.00 mmol), (*R*)-1-phenylethanamine (3.22 mL, 25.0 mmol), sodium iodide (0.038

g, 0.25 mmol) in EtOH (2.5 M). The product was purified using silica-gel flash column chromatography (25% EtOAc/Hexanes + 1% NEt_3) to yield the title compound as a yellow oil (0.830 g, 88%).

TLC R*f*: 0.30 in 20% EtOAc/Hexanes + 1% NEt_3

^{1}H NMR (400 MHz, $CDCl_3$): δ 7.36 – 7.29 (m, 4H), 7.25 – 7.20 (m, 1H), 5.78 (ddt, *J* = 16.9, 10.2, 6.7 Hz, 1H), 4.99 (ap dq, *J* = 17.1, 1.7 Hz, 1H), 4.93 (ddt, *J* = 10.2, 2.3, 1.3 Hz, 1H), 3.75 (q, *J* = 6.6 Hz, 1H), 2.52 (ddd, *J* = 11.4, 7.9, 6.3 Hz, 1H), 2.43 (ddd, *J* = 11.4, 7.9, 6.8 Hz, 1H), 2.14 – 1.98 (m, 2H), 1.62 – 1.50 (m, 2H), 1.35 (d, *J* = 6.6 Hz, 3H).

^{13}C NMR (100 MHz, $CDCl_3$): δ 145.8 (C), 138.5 (CH), 128.4 (CH), 126.8 (CH), 126.5 (CH), 114.5 (CH_2), 58.3 (CH), 47.3 (CH_2), 31.5 (CH_2), 29.5 (CH_2), 24.4 (CH_3).

IR (FTIR): 3028, 2956, 2915, 2810, 1637, 1447, 1124, 906, 761, 697 cm^{-1}.

HRMS (EI): Exact mass calcd for $C_{13}H_{18}N$ [M – H]+ 188.1439. Found: 188.1418.

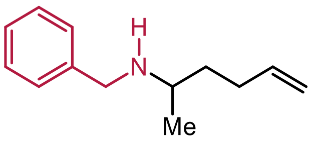

_N_-Benzylhex-5-en-2-amine 1h

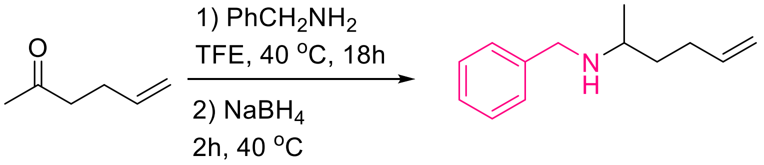

To a vial was added a solution of hex-5-en-2-one (1.0 mL, 8.6 mmol) and TFE (17 mL), which was stirred at rt for 18 h. Then, benzylamine (1.0 mL, 9.5 mmol) was added and the mixture vigorously stirred overnight. Then, $NaBH_4$ (0.39 g, 10 mmol) was added. After completion of the reaction, as monitored by TLC, the reaction mixture was filtered, washing with TFE (17 mL). The filtrate was concentrated via rotary evaporation, and the crude product was purified using silica-gel flash column chromatography (20% EtOAc/petroleum ether + 1% NEt_3) to yield the title compound as a yellow oil (0.90 g, 55% over 2 steps). Characterization data is in good agreement with previously reported data.[3]

TLC R*f*: 34 in 20% EtOAc/Petroleum ether + 1% NEt_3

^{1}H NMR (500 MHz, $CDCl_3$) δ 7.31 – 7.21 (m, 4H), 7.21 – 7.12 (m, 1H), 5.74 (ddt, *J* = 16.9, 10.1, 6.6 Hz, 1H), 5.06 – 4.74 (m, 2H), 3.76 (d, *J* = 12.9 Hz, 1H), 3.67 (d, *J* = 13.0 Hz, 1H), 2.64 (h, *J* =

[3] C. Quinet, P. Jourdain, C. Hermans, A. Ates, I. Lucas and I. E. Markó, *Tetrahedron* 2008, **64**, 1077-1087.

6.3 Hz, 1H), 2.03 (ddddd, J = 15.7, 13.2, 8.0, 6.6, 1.4 Hz, 2H), 1.57 – 1.46 (m, 1H), 1.36 (m, 2H), 1.03 (d, J = 6.3 Hz, 3H).

^{13}C NMR (125 MHz, $CDCl_3$) δ 140.8 (C), 138.8 (CH), 128.4 (CH), 128.2 (CH), 126.9 (CH), 114.5 (CH_2), 52.1 (CH), 51.4 (CH_2), 36.2 (CH_2), 30.3 (CH_2), 20.3 (CH_3).

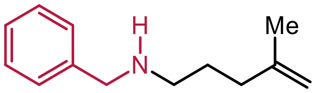

_N_-Benzyl-4-methylpent-4-en-1-amine 1i

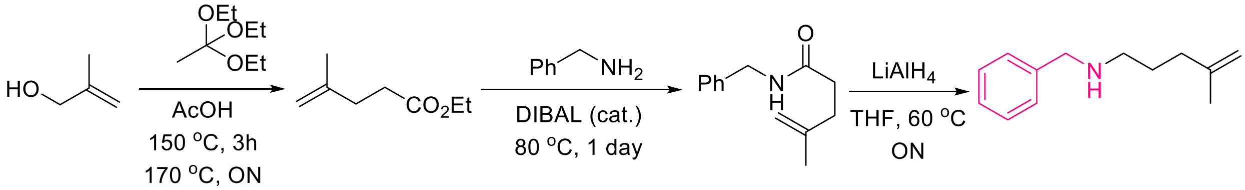

Ethyl 4-methylpent-4-enoate was prepared using a literature procedure. The obtained crude product (2.10 g, 98%) was used next step without further purification.[4] To a vial was added benzylamine (0.60 mL, 5.50 mmol) and 1M DIBAL in THF solution (1.0 mL, 1.0 mmol), which was stirred at rt. After 5 minutes, ethyl 4-methylpent-4-enoate (0.711 g, 5 mmol) was added and the mixture was vigorously stirred at 80 °C for 24 h. Upon completion by TLC, the mixture was transferred to a 100 mL round bottomed flask and the vial was rinsed with additional THF (15 mL) and cooled to 0 °C. $LiAlH_4$ was added slowly to the flask. The reaction mixture was allowed to warm to room temperature, then was and stirred overnight at 60 °C. The reaction was quenched by cooling to 0 °C and adding sequentially water (1 mL per g $LiAlH_4$), then 15% aqueous NaOH (1 mL per g $LiAlH_4$), then water (3 mL per g $LiAlH_4$), then saturated Rochelle's solution (50 mL). The solution was allowed to stir for 1 h then filtered, washing with EtOAc. The filtrate was added into an extraction funnel and the aqueous layer was extracted three times with EtOAc. The combined organic layers were washed with brine, then dried over Na_2SO_4, and concentrated via rotary evaporation. The crude product was purified by alumina column chromatography (20% EtOAc/hexanes + 1% NEt_3) to yield the title compound as a yellow oil (0.53 g, 56% over 3 steps). Characterization data is in good agreement with previously reported data.[5]

TLC R*f*: 0.2 in 20% EtOAc/Petroleum ether + 1% NEt_3

^{1}H NMR (300 MHz, $CDCl_3$) δ 7.30 – 7.22 (m, 4H), 7.22 – 7.12 (m, 1H), 4.67 – 4.56 (m, 2H), 3.71 (s, 2H), 2.62 – 2.48 (m, 2H), 2.04 – 1.92 (m, 2H), 1.64 (s, 3H), 1.64 – 1.52 (m, 2H).

[4] L. A. Adrio, L. S. Quek, J. G. Taylor and K. K. Hii, *Tetrahedron* 2009, **65**, 10334-10338.
[5] A. J. Musacchio, B. C. Lainhart, X. Zhang, S. G. Naugib, T. C. Sherwood and R. R. Knowles, *Science* 2017, **355**, 727.

^{13}C NMR (75 MHz, $CDCl_3$) δ 145.6 (C), 140.4 (C), 128.4 (CH), 128.1 (CH), 126.9 (CH), 110.0 (CH_2), 54.0 (CH_2), 49.1 (CH_2), 35.5 (CH_2), 28.0 (CH_2), 22.4 (CH_3).

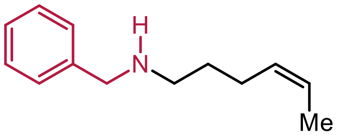

(*Z*)-*N*-Benzylhex-4-en-1-amine 1j'

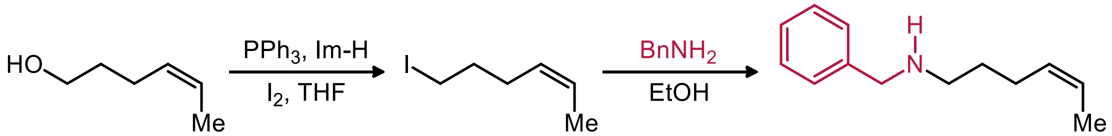

The title compound was synthesized according to a two-step procedure. To a round bottom flask was added (*Z*)-hex-4-en-1-ol (1.00 g, 10.0 mmol) followed by dilution in THF (0.2 M). PPh_3 (3.15 g, 12.0 mmol), then imidazole (2.04 g, 30.0 mmol) were added, and the mixture was cooled to 0 °C. Iodine (2.79 g, 11.0 mmol) was then added. The reaction mixture was stirred at 0 °C for 30 min then rt for 30 min. Upon completion, the reaction was concentrated via rotary evaporation. The reaction mixture was diluted with EtOAc and was extracted using water (x1) and then brine (x1). The organic phase was dried with Na_2SO_4 and filtered. The filtrate was concentrated via rotary evaporation and $OPPh_3$ was removed from the mixture via precipitation using Et_2O/hexanes. The filtrate was concentrated, and this product was used without further purification. Then, the amine was alkylated according to a modified **general procedure A** using the crude 6-iodohex-2-ene, *N*-benzylamine (5.50 mL, 50.0 mmol) in EtOH (2.5 M). The product was purified using silica-gel flash column chromatography (20% EtOAc/Hexanes + 1% NEt_3) to yield the title compound as a yellow oil (0.397 g, 21% over two steps).

TLC R*f*: 0.38 in 20% EtOAc/Hexanes + 1% NEt_3

^{1}H NMR (500 MHz, $CDCl_3$): δ 7.37 – 7.30 (m, 4H), 7.29 – 7.20 (m, 1H), 5.52 – 5.32 (m, 2H), 3.79 (s, 2H), 2.65 (dd, *J* = 7.2 Hz, 2H), 2.14 – 2.04 (m, 2H), 1.64-1.53 (m, 5H).

^{13}C NMR (125 MHz, $CDCl_3$): δ 140.5 (C), 130.1 (CH), 128.4 (CH), 128.1 (CH), 126.8 (CH), 124.2 (CH), 54.1 (CH_2), 49.1 (CH_2), 29.9 (CH_2), 24.6 (CH_2), 12.7 (CH_3).

IR (FTIR): 3012, 2923, 2807, 1653, 1494, 1452, 1117, 1028 cm^{-1}.

HRMS (EI): Exact mass calcd for $C_{13}H_{19}N$ [M]+ 189.1517. Found: 189.1503.

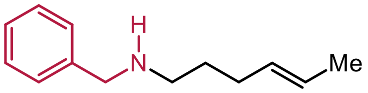

(*E*)-*N*-Benzylhex-4-en-1-amine 1j''

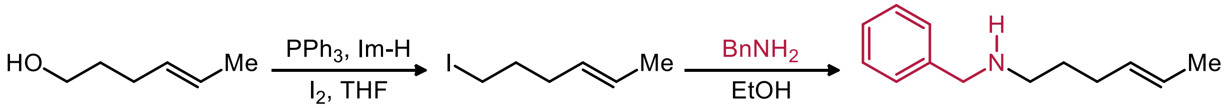

The title compound was synthesized according to a two-step procedure. To a round bottom flask was added (*E*)-hex-4-en-1-ol (1.50 g, 15.0 mmol) followed by dilution in THF (0.2 M). PPh_3 (4.72 g, 18.0 mmol), then imidazole (3.06 g, 45.0 mmol) were added, and the mixture was cooled to 0 °C. Iodine (4.18 g, 16.5 mmol) was then added. The reaction mixture was stirred at 0 °C for 30 min then rt for 30 min. Upon completion, the reaction was concentrated via rotary evaporation. The reaction mixture was diluted with EtOAc and was extracted using water (x1) and then brine (x1). The organic phase was dried with Na_2SO_4 and filtered. The filtrate was concentrated via rotary evaporation and $OPPh_3$ was removed from the mixture via recrystallization using Et_2O/hexanes. The filtrate was concentrated, and this product was used without further purification. Then, the amine was alkylated according to a modified **general procedure A** using the crude 6-iodohex-2-ene, *N*-benzylamine (8.20 mL, 75.0 mmol) in EtOH (2.5 M). The product was purified using silica-gel flash column chromatography (20% EtOAc/Hexanes + 1% NEt_3) to yield the title compound as a yellow oil (1.17 g, 41% over two steps).

TLC R*f*: 0.75 in 10% MeOH/DCM

^{1}H NMR (500 MHz, $CDCl_3$): δ 7.35 – 7.30 (m, 4H), 7.27 – 7.21 (m, 1H), 5.49 – 5.32 (m, 2H), 3.78 (s, 2H), 2.63 (t, *J* = 7.1 Hz, 2H), 2.05 – 1.99 (m, 2H), 1.63 (dt, *J* = 4.8, 1.2 Hz, 3H), 1.57 (p, J = 7.4 Hz, 3H).

^{13}C NMR (125 MHz, $CDCl_3$): δ 140.5 (C), 130.9 (CH), 128.4 (CH), 128.1 (CH), 126.9 (CH), 125.1 (CH), 54.0 (CH_2), 48.9 (CH_2), 30.3 (CH_2), 29.8 (CH_2), 17.0 (CH_3).

IR (FTIR): 3022, 2916, 2851, 2810, 1602, 1494, 1452, 1116, 1028, 963 cm^{-1}.

HRMS (EI): Exact mass calcd for $C_{13}H_{19}N$ [M]+ 189.1517. Found: 189.1508.

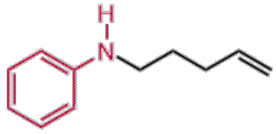

***N*-(Pent-4-en-1-yl)aniline 1k**

The title compound was synthesized according to **general procedure A** using 5-bromopent-1-ene (0.745 g, 5.0 mmol), aniline (2.3 mL, 25.0 mmol), sodium iodide (0.38 g, 0.25 mmol) in EtOH (2.5 M). The product was purified using silica gel flash column chromatography (5%

EtOAc/Hexanes) to yield the title compound as a yellow oil (0.667 g, 83%). Characterization data is in good agreement with previously reported data.[6]

TLC R*f*: 0.39 in 5% EtOAc/Hexanes

^{1}H NMR (500 MHz, $CDCl_3$): δ 7.22 – 7.11 (m, 2H), 6.72 – 6.68 (m, 1H), 6.63 – 6.58 (m, 2H), 5.85 (ddt, *J* = 16.9, 10.2, 6.6 Hz, 1H), 4.98 – 5.14 (m, 2H), 3.64 (br s, 1H), 3.15 (t, *J* = 7.1 Hz, 2H), 2.31 – 2.08 (m, 2H), 1.73 (p, *J* = 7.2 Hz, 2H).

^{13}C NMR (125 MHz, $CDCl_3$): δ148.4 (C), 138.1 (CH), 129.3 (CH), 117.2 (CH), 115.1 (CH_2), 112.8 (CH), 43.4 (CH_2), 31.3 (CH_2), 28.7 (CH_2).

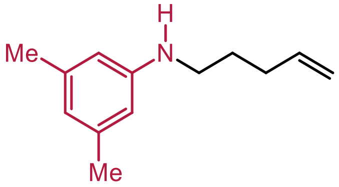

3,5-Dimethyl-*N*-(pent-4-en-1-yl)aniline 1l

The title compound was synthesized according to **general procedure A** using 5-bromopent-1-ene (1.19 g, 8.00 mmol), 3,5-dimethylaniline (5.00 mL, 40.0 mmol), sodium iodide (0.600 g, 0.400 mmol) in EtOH (2.5 M). The product was purified using silica gel flash column chromatography (5% EtOAc/Hexanes) to yield the title compound as an orange-brown oil (1.13 g, 75%).

TLC R*f*: 0.37 in 5% EtOAc/Hexanes

^{1}H NMR (400 MHz, $CDCl_3$): δ 6.37 (s, 1H), 6.26 (s, 1H), 5.85 (ddt, J = 16.9, 10.2, 6.6 Hz, 1H), 5.13 – 4.94 (m, 2H), 3.12 (t, J = 7.1 Hz, 2H), 2.25 (s, 6H), 2.21 – 2.15 (m, 2H), 1.72 (p, J = 7.2 Hz, 2H).

^{13}C NMR (100 MHz, $CDCl_3$): δ 148.4 (C), 138.9 (C), 138.1 (CH), 119.3 (CH), 115.0 (CH_2), 110.8 (CH), 43.5 (CH_2), 31.3 (CH_2), 28.7 (CH_2), 21.5 (CH_3).

IR (FTIR): 3396, 3014, 2917, 2854, 1599, 1513, 1472, 1337, 1185, 990, 908, 818 cm^{-1}.

HRMS (EI): Exact mass calcd for $C_{13}H_{19}N$ [M]+ 189.1517. Found:189.1488.

[6] T. Brown, M. Cumbes, L. J. Diorazio, G. J. Clarkson and M. Wills, *J. Org. Chem.* 2017, **82**, 10489.

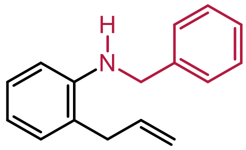

2-Allyl-*N*-benzylaniline 1m

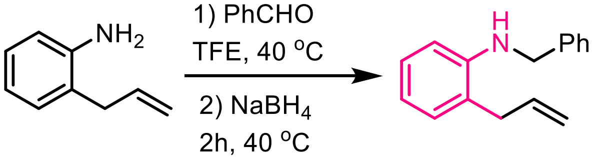

To a clean dry microwave vial was added a solution of benzaldehyde (0.3 mL, 3 mmol) and TFE (6 mL) and was magnetically stirred at RT. After 5 minutes, the respective *o*-allylanline (3 mmol) was added and the mixture vigorously stirred. After stirring for overnight, $NaBH_4$ (0.14 g, 3.6 mmol) was added. After completion of the reaction, as monitored by TLC, the mixture was filtered, washing with TFE (6 mL). The filtrate was concentrated via rotary evaporation and the crude product was purified using silica-gel flash column chromatography (30% toluene/petroleum ether) to yield the title compound as a yellow oil (0.30 g, 44% over 2 steps). Characterization data is in good agreement with previously reported data.[7]

TLC R*f*: 0.38 in 30% Toluene/Petroleum ether

^{1}H NMR (300 MHz, $CDCl_3$) δ 7.33 – 7.24 (m, 4H), 7.20 (tdd, *J* = 10.0, 4.2, 2.7 Hz, 1H), 7.11 – 6.95 (m, 2H), 6.64 (td, *J* = 7.4, 1.2 Hz, 1H), 6.59 – 6.49 (m, 1H), 5.88 (ddt, *J* = 16.6, 10.3, 6.2 Hz, 1H), 5.10 – 4.94 (m, 2H), 4.27 (s, 2H), 4.07 (br s, 1H), 3.24 (dt, *J* = 6.2, 1.7 Hz, 2H).

^{13}C NMR (75 MHz, $CDCl_3$) δ 146.1 (C), 139.4 (C), 136.0 (CH), 129.8 (CH), 128.6 (CH), 127.7 (CH), 127.4 (CH), 127.2 (CH), 123.6 (C), 117.4 (CH), 116.4 (CH_2), 110.8 (CH), 48.2 (CH_2), 36.6 (CH_2).

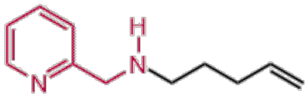

***N*-(Pyridin-2-ylmethyl)pent-4-en-1-amine 1n**

The title compound was synthesized according to **general procedure A** using 5-bromopent-1-ene (1.49 g, 10.0 mmol), pyridine-2-ylmethanamine (5.20 mL, 50.0 mmol), sodium iodide (0.075 g, 0.50 mmol) in EtOH (2.5 M). The product was purified using silica-gel flash column chromatography (8% MeOH/DCM to 12% MeOH/DCM) to yield the title compound as an orange oil (1.39 g, 79%).

TLC R*f*: 0.40 in 10% MeOH/DCM

[7] H. Shen, Q. Deng, R. Liu, Y. Feng, C. Zheng and Y. Xiong, *Org. Chem. Front.* 2017, **4**, 1806-1811.

^{1}H NMR (400 MHz, $CDCl_3$): δ 8.55 (ddd, *J* = 4.9, 1.8, 0.9 Hz, 1H), 7.63 (td, *J* = 7.6, 1.8 Hz, 1H), 7.30 (dt, *J* = 7.9, 1.1 Hz, 1H), 7.15 (ddd, *J* = 7.6, 4.9, 1.2 Hz, 1H), 5.80 (ddt, *J* = 16.9, 10.1, 6.6 Hz, 1H), 5.01 (ap dq, *J* = 17.1, 1.7 Hz, 1H), 4.94 (ddt, *J* = 10.2, 2.2, 1.3 Hz, 1H), 3.90 (s, 2H), 2.67 (t, *J* = 7.3 Hz, 2H), 2.14 (br s, 1H), 2.11 (dtt, *J* = 8.0, 6.6, 1.5 Hz, 3H), 1.64 (p, *J* = 7.4 Hz, 2H).

^{13}C NMR (100 MHz, $CDCl_3$): δ 159.7 (C), 149.3 (CH), 138.4 (CH), 136.4 (CH), 122.2 (CH), 121.9 (CH), 114.6 (CH_2), 55.2 (CH_2), 49.1 (CH_2), 31.5 (CH_2), 29.2 (CH_2).

IR (FTIR): 3083, 2912, 1636, 1588, 1470, 1434, 1129, 991, 911, 747 cm^{-1}.

HRMS (EI): Exact mass calcd for $C_{11}H_{15}N_2$ [M – H]+ 175.1235. Found: 175.1232.

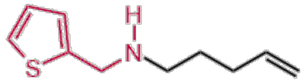

N-(Thiophen-2-ylmethyl)pent-4-en-1-amine 1o

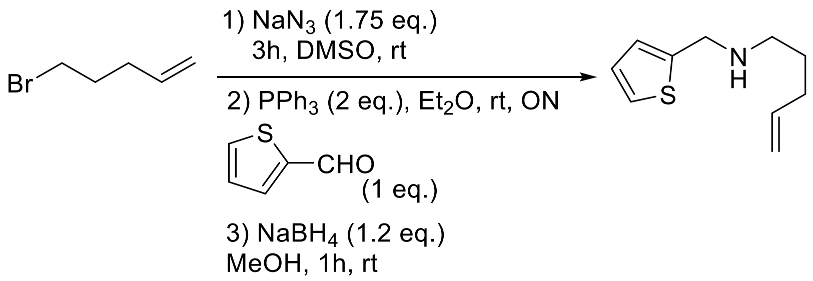

To a round bottom flask was added 5-bromopent-1-ene (1.01 g, 6.8 mmol) followed by dilution in DMSO (1 M). NaN_3 (0.771 g, 11.9 mmol) was added and the mixture was stirred at room temperature for 3 h until the solution gets cloudy. Upon completion, the reaction mixture was poured into water (50 mL) and extracted with Et_2O (50 mL x 2), then the organic layer was dried through Na_2SO_4. PPh_3 (3.56 g, 13.6 mmol) and thiophene – 2 – carbaldehyde (0.76 g, 6.8 mmol) were added to the ether solution and the mixture was stirred over night at room temperature. The reaction mixture was concentrated via rotary evaporation and added MeOH (20 mL) followed by $NaBH_4$ (0.308 g, 8.13 mmol). The mixture was stirred at room temperature for 1h. Upon completion, HCl 1 M (20 mL) and H_2O (30 mL) were added, the slurry mixture was filtered to remove phosphine-based by-products. The acidic solution was extracted with Et_2O (50 mL) to remove other impurities and then basified by NaOH 1M (25 mL). The basic solution was extracted by DCM (50 mL x 2), then the DCM layer was rinsed with water (x1), and then brine (x1). The organic phase was dried with Na_2SO_4 and filtered. The filtrate was concentrated via rotary evaporation. The product was purified using silica-gel flash column chromatography (20% EtOAc/Hexanes + 1% Et_3N) to yield the title compound as a yellow oil (0.43 g, 37%).

TLC R*f*: 0.44 in 30% EtOAc/Hexanes

^{1}H NMR (500 MHz, $CDCl_3$): δ 7.13 (dd, *J* = 5.0, 1.3 Hz, 1H), 6.95 – 6.78 (m, 2H), 5.74 (ddt, *J* = 16.9, 10.2, 6.7 Hz, 1H), 4.94 (ddt, *J* = 17.1, 2.0, 1.6 Hz, 1H), 4.88 (ddt, *J* = 10.1, 2.0, 1.2 Hz, 1H),

3.91 (d, J = 0.8 Hz, 2H), 2.72 – 2.50 (m, 2H), 2.03 (dtt, J = 7.9, 6.6, 1.4 Hz, 2H), 1.63 – 1.46 (m, 2H), 1.31 (br s, 1H).

^{13}C NMR (125 MHz, $CDCl_3$): δ 144.3 (C), 138.4 (CH), 126.5 (CH), 124.6 (CH), 124.2 (CH), 114.6 (CH_2), 48.5 (CH_2), 48.3 (CH_2), 31.4 (CH_2), 29.1 (CH_2).

IR (FTIR): 3309, 3070, 2919, 2812, 1639, 1437, 1108, 908, 691 cm^{-1}.

HRMS (EI): Exact mass calcd for $C_{10}H_{15}NS$ [M]+ 181.0925. Found: 181.0940.

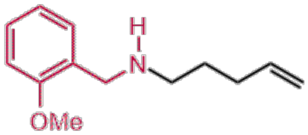

***N*-(2-Methoxybenzyl)pent-4-en-1-amine 1p**

The title compound was synthesized according to **general procedure A** using 5-bromopent-1-ene (1.013 g, 6.80 mmol), (2-methoxyphenyl)methanamine (4.44 mL, 34.0 mmol), sodium iodide (0.102 g, 0.680 mmol) in EtOH (2.5 M). The product was purified using silica-gel flash column chromatography (20% EtOAc/Hexanes + 1% Et_3N) to yield the title compound as a yellow oil (0.904 g, 65%).

TLC R*f*: 0.25 in 30% EtOAc/Hexanes

^{1}H NMR (500 MHz, $CDCl_3$): δ 7.20 – 7.12 (m, 2H), 6.84 (td, J = 7.4, 1.1 Hz, 1H), 6.79 (dd, J = 8.6, 1.1 Hz, 1H), 5.74 (ddt, J = 16.9, 10.2, 6.7 Hz, 1H), 4.93 (dq, J = 17.1, 1.7 Hz, 1H), 4.87 (ddt, J = 10.2, 2.3, 1.3 Hz, 1H), 3.76 (s, 3H), 3.71 (s, 2H), 2.54 (t, J = 7.2 Hz, 2H), 2.06 – 1.98 (m, 2H), 1.54 (m, 3H).

^{13}C NMR (125 MHz, $CDCl_3$): δ 157.7 (C), 138.7 (CH), 129.8 (CH), 128.6 (C), 128.1 (CH), 120.4 (CH), 114.5 (CH_2), 110.2 (CH), 55.2 (CH_3), 49.4 (CH_2), 48.8 (CH_2), 31.6 (CH_2), 29.3 (CH_2).

IR (FTIR): 3332, 3074, 2926, 2835, 1639, 1600, 1490, 1237, 1029, 908, 749 cm^{-1}.

HRMS (EI): Exact mass calcd for $C_{13}H_{19}NO$ [M]+ 205.1467. Found: 205.1459.

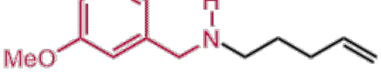

***N*-(3-Methoxybenzyl)pent-4-en-1-amine 1q**

The title compound was synthesized according to **general procedure A** using 5-bromopent-1-ene (1.01 g, 6.80 mmol), (3-methoxyphenyl)methanamine (4.35 mL, 34.0 mmol), sodium iodide (0.102 g, 0.680 mmol) in EtOH (2.5 M). The product was purified using silica-gel flash column

chromatography (20% EtOAc/Hexanes + 1% Et_3N) to yield the title compound as a yellow oil (0.97 g, 69%).

TLC R*f*: 0.3 in 30% EtOAc/Hexanes

^{1}H NMR (500 MHz, $CDCl_3$): δ 7.19 – 7.12 (m, 1H), 6.85 – 6.79 (m, 2H), 6.71 (ddd, *J* = 8.2, 2.6, 1.0 Hz, 1H), 5.74 (ddt, *J* = 17.0, 10.2, 6.7 Hz, 1H), 4.94 (dq, *J* = 17.1, 1.7 Hz, 1H), 4.87 (ddt, *J* = 10.2, 2.3, 1.3 Hz, 1H), 3.73 (s, 3H), 3.69 (s, 2H), 2.60 – 2.53 (m, 2H), 2.07 – 1.99 (m, 2H), 1.54 (p, *J* = 7.4 Hz, 2H), 1.20 (s, 1H).

^{13}C NMR (125 MHz, $CDCl_3$): δ 159.8 (C), 142.3 (C), 138.6 (CH), 129.4 (CH), 120.4 (CH), 114.6 (CH_2), 113.6 (CH), 112.4 (CH), 55.2 (CH_3), 54.0 (CH_2), 49.0 (CH_2), 31.6 (CH_2), 29.3 (CH_2).

IR (FTIR): 3310, 3075, 2925, 2831, 1584, 1451, 1260, 1152, 1042, 908, 775, 691 cm^{-1}.

HRMS (EI): Exact mass calcd for $C_{13}H_{19}NO$ [M]+ 205.1467. Found: 205.1489.

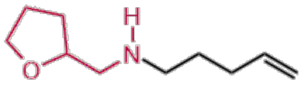

***N*-((Tetrahydrofuran-2-yl)methyl)pent-4-en-1-amine 1r**
The title compound was synthesized according to **general procedure A** using 5-bromopent-1-ene (1.49 g, 10.0 mmol), (tetrahydrofuran-2-yl)methanamine (5.20 mL, 50.0 mmol), sodium iodide (0.075 g, 0.50 mmol) in EtOH (2.5 M). The product was purified using silica-gel flash column chromatography (12% MeOH/DCM) to yield the title compound as a pale yellow oil (1.03 g, 61%).

TLC R*f*: 0.31 in 10% MeOH/DCM

^{1}H NMR (400 MHz, $CDCl_3$): δ 5.80 (ddt, *J* = 16.9, 10.2, 6.6 Hz, 1H), 5.00 (ap dq, *J* = 17.1, 1.7 Hz, 1H), 4.94 (ddt, *J* = 10.2, 2.2, 1.3 Hz, 1H), 4.00 (qd, *J* = 7.3, 3.9 Hz, 1H), 3.84 (dt, *J* = 8.4, 6.7 Hz, 1H), 3.73 (dt, *J* = 8.3, 6.8 Hz, 1H), 2.72 – 2.56 (m, 4H), 2.13 – 2.03 (m, 2H), 1.96 (dddd, *J* = 11.5, 8.5, 6.7, 5.1 Hz, 1H), 1.91 – 1.82 (m, 2H), 1.65 – 1.43 (m, 3H).

^{13}C NMR (100 MHz, $CDCl_3$): δ 138.5 (CH), 114.5 (CH_2), 78.2 (CH), 67.8 (CH_2), 54.4 (CH_2), 49.6 (CH_2), 31.5 (CH_2), 29.3 (CH_2), 29.2 (CH_2), 25.7 (CH_2).

IR (FTIR): 3054, 2959, 2912, 2847, 1637, 1462, 1129, 1056, 996, 905, 737 cm^{-1}.

HRMS (EI): Exact mass calcd for $C_{10}H_{18}NO$ [M – H]+ 168.1388. Found: 168.1387.

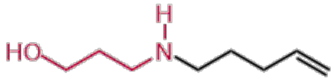

3-(Pent-4-en-1-ylamino)propan-1-ol 1s
The title compound was synthesized according to **general procedure A** using 5-bromopent-1-ene (1.01 g, 6.80 mmol), (3-methoxyphenyl)methanamine (2.59 mL, 34.0 mmol), sodium iodide (0.102 g, 0.680 mmol) in EtOH (2.5 M). The product was purified using silica-gel flash column chromatography (40% MeOH/DCM + 1% NH_4OH) to yield the title compound as a yellow oil (0.45 g, 46%).

TLC R*f*: 0.32 in 40% MeOH/DCM + 1% NH_4OH

^{1}H NMR (500 MHz, $CDCl_3$): δ 5.79 (ddt, *J* = 16.9, 10.2, 6.6 Hz, 1H), 5.01 (dq, *J* = 17.1, 1.7 Hz, 1H), 4.96 (dq, *J* = 10.1, 1.4 Hz, 1H), 3.83 – 3.77 (m, 2H), 3.07 (br s, 2H), 2.90 – 2.84 (m, 2H), 2.62 (t, *J* = 7.1 Hz, 2H), 2.13 – 2.04 (m, 2H), 1.69 (p, *J* = 5.5 Hz, 2H), 1.57 (p, *J* = 7.3 Hz, 2H).

^{13}C NMR (125 MHz, $CDCl_3$): δ 138.2 (CH), 114.9 (CH_2), 64.6 (CH_2), 50.2 (CH_2), 49.2 (CH_2), 31.4 (CH_2), 30.6 (CH_2), 29.0 (CH_2).

IR (FTIR): 3268, 3071, 2924, 2833, 1639, 1437, 1114, 1060, 907 cm^{-1}.

HRMS (ESI): Exact mass calcd for $C_8H_{18}NO$ [M+H]+ 144.1388. Found: 144.1360.

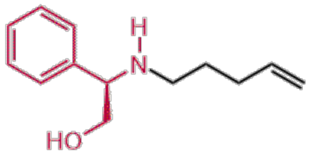

(*R*)-2-(Pent-4-en-1-ylamino)-2-phenylethanol 1t
The title compound was synthesized according to **general procedure A** using 5-bromopent-1-ene (0.745 g, 5.00 mmol), (*R*)-2-amino-2-phenylethanol (3.43 g, 25.0 mmol), sodium iodide (0.038 g, 0.025 mmol) in EtOH (2.5 M). The product was purified using silica-gel flash column chromatography (70% EtOAc/Hexanes) to yield the title compound as a pale yellow oil (0.615 g, 60%).

TLC R*f*: 0.35 in 70% EtOAc/Hexanes

^{1}H NMR (500 MHz, $CDCl_3$): δ 7.40 – 7.30 (m, 2H), 7.30 – 7.24 (m, 3H), 5.78 (ddt, *J* = 16.9, 10.2, 6.7 Hz, 1H), 4.99 (ap dq, *J* = 17.1, 1.7 Hz, 1H), 4.94 (ddt, *J* = 10.2, 2.3, 1.4 Hz, 1H), 3.75 (dd, *J* = 8.5, 4.5 Hz, 1H), 3.70 (dd, *J* = 10.5, 4.5 Hz, 1H), 3.52 (dd, *J* = 10.5, 8.5 Hz, 1H), 2.59 (dt, *J* = 11.5, 7.1 Hz, 1H), 2.49 (ddd, *J* = 11.5, 7.6, 6.5 Hz, 1H), 2.19 – 1.99 (m, 2H), 1.58 (dddd, *J* = 12.8, 11.2, 9.3, 6.5 Hz, 2H).

^{13}C NMR (125 MHz, $CDCl_3$): δ 140.9 (C), 138.4 (CH), 128.6 (CH), 127.6 (CH), 127.0 (CH), 114.7 (CH_2), 66.5 (CH_2), 64.5 (CH), 46.8 (CH_2), 31.4 (CH_2), 29.4 (CH_2).

IR (FTIR): 3336, 3053, 2908, 2823, 1643, 1472, 1449, 1041, 916, 753, 699 cm^{-1}.

HRMS (ESI): Exact mass calcd for $C_{13}H_{20}NO$ [M+H]+ 206.1545. Found: 206.1555.

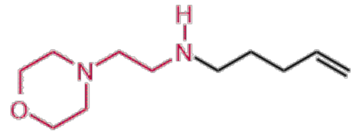

***N*-(2-Morpholinoethyl)pent-4-en-1-amine 1u**
The title compound was synthesized according to **general procedure A** using 5-bromopent-1-ene (1.49 g, 10.0 mmol), 2-morpholinoethanamine (6.60 mL, 50.0 mmol), sodium iodide (0.075 g, 0.50 mmol) in EtOH (2.5 M). The product was adequately pure with further purification yielding the title compound as a yellow oil (1.62 g, 82%).

TLC R*f*: 0.50 in 10% MeOH/DCM

^{1}H NMR (400 MHz, $CDCl_3$): δ 5.81 (ddt, *J* = 16.9, 10.1, 6.7 Hz, 1H), 5.01 (ap dq, *J* = 17.1, 1.7 Hz, 1H), 4.94 (ddt, *J* = 10.1, 2.3, 1.2 Hz, 1H), 3.77 – 3.62 (m, 4H), 2.69 (dd, *J* = 6.6, 5.6 Hz, 2H), 2.65 – 2.58 (m, 2H), 2.48 (dd, *J* = 6.6, 5.7 Hz, 2H), 2.45 – 2.35 (m, 4H), 2.15 – 2.03 (m, 2H), 1.65 – 1.49 m, 2H).

^{13}C NMR (100 MHz, $CDCl_3$): δ 138.4 (CH), 114.6 (CH_2), 67.0 (CH_2), 58.3 (CH_2), 53.7 (CH_2), 49.4 (CH_2), 46.0 (CH_2), 31.5 (CH_2), 29.1 (CH_2).

IR (FTIR): 3085, 2915, 2846, 2813, 1636, 1450, 1270, 1118, 907, 758 cm^{-1}.

HRMS (ESI): Exact mass calcd for $C_{11}H_{23}N_2O$ [M+H]+ 199.1810. Found: 199.1818.

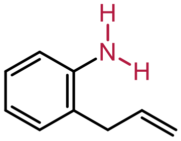

2-Allylaniline 1v

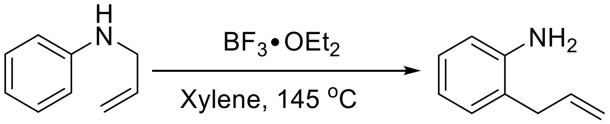

The title compound was prepared using a modified literature procedure to yield the title compound as a yellow oil (0.57 g, 57%). Characterization data is in good agreement with previously reported data.[8]

TLC R*f*: 0.38 in 10% EtOAc/Petroleum ether

^{1}H NMR (300 MHz, $CDCl_3$) δ 7.05 – 6.91 (m, 2H), 6.67 (tt, *J* = 7.4, 1.3 Hz, 1H), 6.59 (d, *J* = 7.8 Hz, 1H), 5.97 – 5.77 (m, 1H), 5.10 – 4.94 (m, 2H), 3.62 – 3.53 (m, 2H), 3.22 (dd, *J* = 6.0, 1.6 Hz, 2H).

^{13}C NMR (75 MHz, $CDCl_3$) δ 144.8 (C), 136.0 (CH), 130.2 (CH), 127.6 (CH), 124.0 (C), 118.9 (CH), 116.1 (CH_2), 115.8 (CH), 36.5 (CH_2).

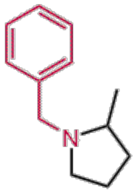

1-Benzyl-2-methylpyrrolidine 4a

The title compound was synthesized according to **general procedure B** using urea hydrogen peroxide (0.068 g, 0.72 mmol), amine **1a** (0.105 g, 0.60 mmol), $B_2(OH)_4$ (0.064 g, 0.72 mmol) in TFE (0.1 M). The product was isolated after aqueous extraction to yield the title compound as a pale yellow oil (0.090 g, 85%). Characterization data is in good agreement with previously reported data.[9]

TLC R*f*: 0.45 in 10% MeOH/DCM

^{1}H NMR (500 MHz, $CDCl_3$): δ 7.34 – 7.29 (m, 4H), 7.25 – 7.22 (m, 1H), 4.02 (d, *J* = 12.8 Hz, 1H), 3.14 (d, *J* = 12.8 Hz, 1H), 2.91 (td, *J* = 10.8, 2.6 Hz, 1H), 2.39 (dq, *J* = 13.5, 6.2 Hz, 1H), 2.10 (q, *J* = 9.0 Hz, 1H), 1.94 (dddd, *J* = 12.5, 9.7, 7.3, 5.3 Hz, 1H), 1.76 – 1.67 (m, 1H), 1.64 (dddd, *J* = 13.8, 8.7, 6.3, 3.3 Hz, 1H), 1.46 (dddd, *J* = 12.5, 10.8, 8.6, 5.8 Hz, 1H), 1.18 (d, *J* = 6.0 Hz, 3H).

^{13}C NMR (125 MHz, $CDCl_3$): δ 139.6 (C), 129.1 (CH), 128.2 (CH), 126.8 (CH), 59.6 (CH), 58.4 (CH_2), 54.1 (CH_2), 32.8 (CH_2), 21.5 (CH_2), 19.2 (CH_3).

---

[8] S. Fu, S.; H. Yang, G. Li, Y. Deng, H. Jiang and W. Zeng, *Org. Lett.* 2015, **17**, 1018–1021.
[9] J. Zhang and S. Chang, *J. Am. Chem. Soc.* 2020, **142**, 12585.

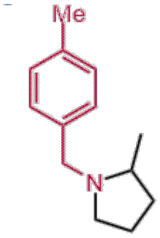

2-Methyl-1-(4-methylbenzyl)pyrrolidine 4b

The title compound was synthesized according to **general procedure B** using urea hydrogen peroxide (0.0677 g, 0.72 mmol), amine **1b** (0.114 g, 0.60 mmol), $B_2(OH)_4$ (0.118 g, 1.32 mmol) in TFE (0.1 M). The crude reaction mixture was concentrated via rotary evaporation, then diluted with DCM (50 mL) and water (50 mL). 10 mL of 1 M HCl was added, and the phases were separated. Additional 20 mL of water was added into DCM layer, the aqueous phases were separated and combined. The product was isolated after basic extraction to yield the title compound as a yellow oil (0.076 g, 67%).

TLC R*f*: 0.39 in 5% MeOH/DCM + 0.5% NH_4OH

^{1}H NMR (500 MHz, $CDCl_3$): δ 7.15 – 7.10 (m, 2H), 7.09 – 6.99 (m, 2H), 3.90 (d, *J* = 12.7 Hz, 1H), 3.05 (d, *J* = 12.8 Hz, 1H), 2.82 (ddd, *J* = 9.4, 8.1, 2.6 Hz, 1H), 2.35 – 2.26 (m, 1H), 2.25 (s, 3H), 2.02 (q, *J* = 9.0 Hz, 1H), 1.85 (dddd, *J* = 12.5, 9.7, 7.4, 5.3 Hz, 1H), 1.69 – 1.57 (m, 1H), 1.57 – 1.48 (m, 1H), 1.38 (dddd, *J* = 12.3, 10.8, 8.6, 5.8 Hz, 1H), 1.09 (d, *J* = 6.0 Hz, 3H).

^{13}C NMR (125 MHz, $CDCl_3$): δ 136.4 (C), 136.2 (C), 129.1 (CH), 128.9 (CH), 59.6 (CH_3), 57.9 (CH_2), 53.9 (CH_2), 32.8 (CH_2), 21.5 (CH_2), 21.1 (CH), 19.1 (CH_3).

IR (FTIR): 3004, 2960, 2779, 1513, 1373, 1138, 1100, 804 cm^{-1}.

HRMS (EI): Exact mass calcd for $C_{13}H_{19}N$ [M]+ 189.1517. Found: 189.1515.

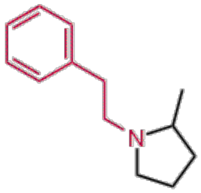

2-Methyl-1-phenethylpyrrolidine 4c

The title compound was synthesized according to a modified **general procedure B** using urea hydrogen peroxide (0.085 g, 0.90 mmol), amine **1c** (0.114 g, 0.60 mmol), $B_2(OH)_4$ (0.081 g, 0.90 mmol) in TFE (0.1 M). The product was isolated after aqueous extraction to yield the title compound as a pale yellow oil (0.078 g, 68%).

TLC R*f*: 0.45 in 10% MeOH/DCM

^1H NMR (500 MHz, $CDCl_3$): δ 7.32 – 7.25 (m, 2H), 7.22 – 7.15 (m, 3H), 3.26 (td, *J* = 8.7, 2.7 Hz, 1H), 3.03 (td, *J* = 11.5, 5.8 Hz, 1H), 2.83 (pd, *J* = 13.0, 5.6 Hz, 2H), 2.37 – 2.26 (m, 2H), 2.20 (q, *J* = 8.8 Hz, 1H), 1.93 (dddd, *J* = 12.3, 9.7, 7.2, 5.2 Hz, 1H), 1.82 (dddd, *J* = 17.3, 10.8, 8.5, 5.2 Hz, 1H), 1.71 (dddd, *J* = 12.5, 11.4, 6.0, 2.7 Hz, 1H), 1.45 (dddd, *J* = 12.3, 10.6, 8.7, 6.0 Hz, 1H), 1.12 (d, *J* = 6.1 Hz, 3H).

^{13}C NMR (125 MHz, $CDCl_3$): δ 140.7 (C), 128.6 (CH), 128.3 (CH), 126.0 (CH), 60.0 (CH), 56.1 (CH_2), 54.0 (CH_2), 35.5 (CH_2), 32.7 (CH_2), 21.7 (CH_2), 18.93 (CH_3).

IR (FTIR): 3029, 2953, 2775, 1600, 1457, 1451, 1373, 1134, 743, 695 cm^{-1}.

HRMS (EI): Exact mass calcd for $C_{13}H_{19}N$ [M]+189.1517. Found: 189.1495.

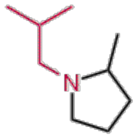

1-Isobutyl-2-methylpyrrolidine hydrochloride 4d

The title compound was synthesized according to **general procedure B** using urea hydrogen peroxide (0.0677 g, 0.72 mmol), amine **1d** (0.114 g, 0.60 mmol), $B_2(OH)_4$ (0.118 g, 1.32 mmol) in TFE (0.1 M). The crude reaction mixture was concentrated via rotary evaporation, then diluted with DCM (50 mL) and water (50 mL). 10 mL of 1M HCl was added, and the phases were separated. Additional 20 mL of water was added into DCM layer, the aqueous phases were separated and combined. The product was isolated after basic extraction and direct salt formation in DCM by HCl/dioxane 4 M solution (0.2 mL, 0,8 mmol) to yield the title compound as a white solid (0.079 g, 74%).

TLC R*f*: 0.18 in 5% MeOH/DCM + 0.5% NH_4OH

^1H NMR (500 MHz, CD_3OD): δ 3.75 (ddd, *J* = 11.6, 7.9, 5.5 Hz, 1H), 3.48 (dt, *J* = 9.9, 6.5 Hz, 1H), 3.24 – 3.11 (m, 2H), 2.93 (dd, *J* = 12.8, 5.2 Hz, 1H), 2.31 (dtd, *J* = 13.0, 7.5, 5.0 Hz, 1H), 2.10 (dqt, *J* = 12.0, 8.6, 3.6 Hz, 4H), 1.82 (dq, *J* = 13.1, 9.2 Hz, 1H), 1.49 (d, *J* = 6.5 Hz, 3H), 1.10 (d, *J* = 6.6 Hz, 3H), 1.06 (d, *J* = 6.7 Hz, 3H).

^{13}C NMR (125 MHz, CD_3OD): δ 65.8 (CH), 61.1 (CH_2), 53.9 (CH_2), 30.8 (CH_2), 25.5 (CH), 21.0 (CH_2), 19.8 (CH_3), 19.2 (CH_3), 14.6 (CH_3).

IR (FTIR): 3388, 2964, 2873, 2592, 1420, 1047 cm^{-1}.

HRMS (EI): Exact mass calcd for $C_9H_{19}N$ [M]+ 141.1517. Found: 141.1533.

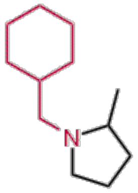

1-(Cyclohexylmethyl)-2-methylpyrrolidine 4e

The title compound was synthesized according to **general procedure B** using urea hydrogen peroxide (0.0677 g, 0.72 mmol), amine **1e** (0.109 g, 0.60 mmol), $B_2(OH)_4$ (0.118 g, 1.32 mmol) in TFE (0.1 M). The crude reaction mixture was concentrated via rotary evaporation, then diluted with DCM (50 mL) and water (50 mL). 10 mL of 1M HCl was added, and the phases were separated. Additional 20 mL of water was added into DCM layer, the aqueous phases were separated and combined. The product was isolated after basic extraction to yield the title compound as a yellow oil (0.070 g, 64%).

TLC R*f*: 0.38 in 5% MeOH/DCM + 0.5% NH_4OH

^{1}H NMR (500 MHz, $CDCl_3$): δ 3.07 (td, *J* = 8.7, 2.8 Hz, 1H), 2.44 (dd, *J* = 11.9, 9.4 Hz, 1H), 2.22 – 2.10 (m, 1H), 1.97 (q, *J* = 8.9 Hz, 1H), 1.92 – 1.75 (m, 3H), 1.75 – 1.53 (m, 6H), 1.43 – 1.29 (m, 2H), 1.24 – 1.01 (m, 3H), 1.00 (d, *J* = 6.1 Hz, 3H), 0.89 – 0.72 (m, 2H).

^{13}C NMR (125 MHz, $CDCl_3$): δ 61.8 (CH_2), 60.8 (CH), 54.6 (CH_2), 37.1 (CH), 32.6 (CH_2), 32.5 (CH_2), 32.0 (CH_2), 26.8 (CH_2), 26.3 (CH_2), 26.1 (CH_2), 21.8 (CH_2), 19.0 (CH_3).

IR (FTIR): 2958, 2918, 2849, 2779, 1447, 1374, 1170, 1118 cm^{-1}.

HRMS (EI): Exact mass calcd for $C_{12}H_{23}N$ [M]+ 181.1830. Found: 181.1821.

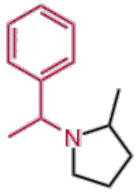

2-Methyl-1-(1-phenylethyl)pyrrolidine 4f

The title compound was synthesized according to **general procedure B** using urea hydrogen peroxide (0.068 g, 0.72 mmol), amine **1f** (0.114 g, 0.60 mmol), $B_2(OH)_4$ (0.065 g, 0.72 mmol) in TFE (0.1 M). The product was isolated after aqueous extraction to yield the title compound as a 2.2:1 diastereomeric mixture and a pale yellow oil (0.098 g, 86%).

TLC R*f*: 0.40 in 10% MeOH/DCM

^{1}H NMR (500 MHz, $CDCl_3$): δ (*major*) 7.38 – 7.20 (m, 5H), 3.86 (q, *J* = 6.9 Hz, 1H), 2.87 (td, *J* = 8.5, 3.3 Hz, 1H), 2.55 (ap h, *J* = 6.3 Hz, 1H), 2.41 (q, *J* = 8.2 Hz, 1H), 1.82 (ddt, *J* = 11.8, 9.0, 7.3 Hz, 1H), 1.74 (tdd, *J* = 9.3, 7.4, 4.4 Hz, 1H), 1.61 – 1.54 (m, 1H), 1.47 (d, *J* = 6.9 Hz, 3H), 1.43 – 1.39 (m, 1H), 1.11 (d, *J* = 6.1 Hz, 3H). δ (*minor*) 7.38 – 7.20 (m, 5H), 3.68 (q, *J* = 6.7 Hz, 1H), 2.95 – 2.89 (m, 1H), 2.77 (dt, *J* = 9.1, 3.7 Hz, 1H), 2.46 (q, *J* = 8.8 Hz, 1H), 1.92 (ddt, *J* = 12.3, 9.2, 7.7 Hz, 1H), 1.78 – 1.71 (m, 1H), 1.66 (ddd, *J* = 16.0, 7.8, 3.7 Hz, 1H),1.43 – 1.39 (m, 1H), 1.36 (d, *J* = 6.6 Hz, 3H), 0.86 (d, *J* = 6.2 Hz, 3H).

^{13}C NMR (125 MHz, $CDCl_3$): δ (*major*) 140.2 (C), 128.1 (CH), 127.9 (CH), 126.8 (CH), 58.7 (CH), 54.9 (CH), 48.2 (CH_2), 32.6 (CH_2), 21.8 (CH_2), 19.4 (CH_3), 18.4 (CH_3). δ (minor) 141.9 (C), 128.0 (CH), 127.7 (CH), 126.6 (CH), 60.4 (CH), 56.2 (CH), 49.8 (CH_2), 32.9 (CH_2), 22.3 (CH_2), 21.8 (CH_3), 19.0 (CH_3).

IR (FTIR): 3019, 2965, 2875, 2777, 1456, 1449, 1367, 1153, 757, 698 cm^{-1}.

HRMS (EI): Exact mass calcd for $C_{13}H_{19}N$ [M]+ 189.1517. Found: 189.1532.

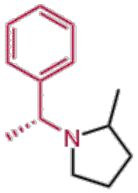

2-Methyl-1-((*R*)-1-phenylethyl)pyrrolidine 4g
The title compound was synthesized according to a modified **general procedure B** using urea hydrogen peroxide (0.068 g, 0.72 mmol), amine **1g** (0.114 g, 0.60 mmol), $B_2(OH)_4$ (0.118 g, 1.32 mmol) in TFE (0.1 M). The product was isolated after aqueous extraction to yield the title compound as a yellow oil (0.067 g, 59%).

TLC R*f*: 0.40 in 10% MeOH/DCM

^{1}H NMR (500 MHz, $CDCl_3$): δ (*major*) 7.38 – 7.20 (m, 5H), 3.86 (q, *J* = 6.9 Hz, 1H), 2.86 (td, *J* = 8.3, 3.4 Hz, 1H), 2.54 (ap h, *J* = 6.3 Hz, 1H), 2.40 (q, *J* = 8.1 Hz, 1H), 1.82 (ddt, *J* = 11.8, 9.0, 7.3 Hz, 1H), 1.74 (tdd, *J* = 9.3, 7.4, 4.4 Hz, 1H), 1.61 – 1.54 (m, 1H), 1.46 (d, *J* = 6.9 Hz, 3H), 1.43 – 1.39 (m, 1H), 1.10 (d, *J* = 6.1 Hz, 3H). δ (*minor*) 7.38 – 7.20 (m, 5H), 3.67 (q, *J* = 6.6 Hz, 1H), 2.95 – 2.89 (m, 1H), 2.77 (dt, *J* = 8.7, 3.7 Hz, 1H), 2.45 (q, *J* = 8.8 Hz, 1H), 1.92 (ddt, *J* = 12.2, 9.2, 7.7 Hz, 1H), 1.78 – 1.71 (m, 1H), 1.66 (ddd, *J* = 16.0, 7.8, 3.7 Hz, 1H),1.43 – 1.39 (m, 1H), 1.36 (d, *J* = 6.6 Hz, 3H), 0.85 (d, *J* = 6.2 Hz, 3H).

^{13}C NMR (125 MHz, $CDCl_3$): δ (*major*) 140.9 (C), 128.1 (CH), 127.9 (CH), 126.8 (CH), 58.7 (CH), 54.9 (CH), 48.2 (CH_2), 32.5 (CH_2), 21.8 (CH_2), 19.4 (CH_3), 18.4 (CH_3). δ (minor) 141.9 (C), 128.0 (CH), 127.7 (CH), 126.6 (CH), 60.4 (CH), 56.2 (CH), 49.9 (CH_2), 32.9 (CH_2), 22.3 (CH_2), 21.7 (CH_3), 19.0 (CH_3).

IR (FTIR): 3018, 2957, 2865, 2779, 1456, 1450, 1367, 1155, 763, 699 cm^{-1}.

HRMS (ESI): Exact mass calcd for $C_{13}H_{20}N$ [M+H]+ 190.1596. Found: 190.1591.

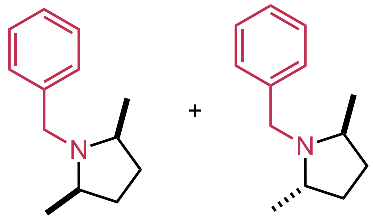

1-Benzyl-2,5-dimethylpyrrolidine 4h

The title compound was synthesized according to **general procedure B** using urea hydrogen peroxide (0.068 g, 0.72 mmol), amine **1h** (0.134 g, 0.60 mmol), $B_2(OH)_4$ (0.118 g, 1.32 mmol) in TFE (0.1 M). The crude reaction mixture was concentrated via rotary evaporation, then diluted with DCM (50 mL) and water (50 mL). 10 mL of 1M HCl was added, and the phases were separated. 15 mL of 1M KOH and 100 mL of brine were added. The product was isolated after 3 x 50 mL DCM extractions to yield the title compound as a 6.8:1 diastereomeric mixture as a pail yellow oil (0.089 g, 78%). Characterization data is in good agreement with previously reported data.[3]

TLC R*f*: 0.48 in 5% MeOH/DCM + 1% NEt_3

^{1}H NMR (500 MHz, $CDCl_3$) δ (*cis*) 7.25 – 7.09 (m, 5H), 3.65 (s, 2H), 2.55 – 2.44 (m, 2H), 1.74 – 1.64 (m, 2H), 1.34 – 1.23 (m, 2H), 0.97 (d, *J* = 6.1 Hz, 6H). δ (*trans*) 7.41 – 7.00 (m, 5H), 3.75 (d, *J* = 13.8 Hz, 1H), 3.43 (d, *J* = 13.8 Hz, 1H), 2.99 – 2.86 (m, 2H), 1.97 – 1.84 (m, 2H), 1.34 – 1.23 (m, 2H), 0.89 (d, *J* = 6.3 Hz, 6H).

^{13}C NMR (125 MHz, $CDCl_3$) δ (*cis*) 139.3 (C), 129.3 (CH), 128.0 (CH), 126.7 (CH), 59.7 (CH), 55.2 (CH_2), 31.3 (CH_2), 20.7 (CH_3). δ (*trans*) 140.4 (C), 128.6 (CH), 128.1 (CH), 126.5 (CH), 55.1 (CH), 51.7 (CH_2), 31.0 (CH_2), 17.1 (CH_3).

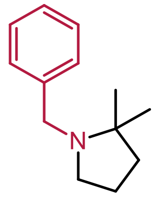

1-Benzyl-2,2-dimethylpyrrolidine 4i

The title compound was synthesized according to **general procedure B** using urea hydrogen peroxide (0.068 g, 0.72 mmol), amine **1i** (0.114 g, 0.60 mmol), $B_2(OH)_4$ (0.065 g, 0.72 mmol) in TFE (0.1 M). The crude reaction mixture was concentrated via rotary evaporation, then diluted with DCM (50 mL) and water (50 mL). 10 mL of 1 M HCl was added, and the phases were

separated. The product was isolated after basic extraction to yield the title compound as a yellow oil (0.077 g, 68%). Characterization data is in good agreement with previously reported data.[10]

TLC R*f*: 0.34 in 5% MeOH/DCM + 1% NEt_3

^{1}H NMR (300 MHz, $CDCl_3$) δ 7.30 – 7.06 (m, 5H), 3.42 (s, 2H), 2.60 – 2.45 (m, 2H), 1.73 – 1.52 (m, 4H), 1.01 (s, 6H).

^{13}C NMR (75 MHz, $CDCl_3$) δ 141.0 (C), 128.6 (CH), 128.2 (CH), 126.6 (CH), 60.2 (C), 53.2 (CH_2), 50.9 (CH_2), 40.0 (CH_2), 23.1 (CH_3), 20.5 (CH_2).

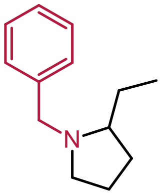

1-Benzyl-2-ethylpyrrolidine 4j

The title compound was synthesized according to a modified **general procedure B** using urea hydrogen peroxide (0.068 g, 0.72 mmol), amine **1j'** (0.114 g, 0.60 mmol), $B_2(OH)_4$ (0.118 g, 1.32 mmol) in TFE (0.1 M). The product was isolated after aqueous extraction to yield the title compound as a yellow oil (0.053 g, 47%). Characterization data is in good agreement with previously reported data.[5]

TLC R*f*: 0.34 in 10% MeOH/DCM

^{1}H NMR (500 MHz, $CDCl_3$): δ 7.39 – 7.25 (m, 5H), 4.05 (d, J = 12.9 Hz, 1H), 3.17 (d, J = 12.9 Hz, 1H), 2.93 (ddd, J = 9.1, 7.3, 2.4 Hz, 1H), 2.29 (qd, J = 8.1, 3.1 Hz, 1H), 2.12 (q, J = 9.0 Hz, 1H), 1.93 (dddd, J = 12.3, 9.5, 7.8, 6.0 Hz, 1H), 1.79 (dqd, J = 13.2, 7.5, 3.3 Hz, 1H), 1.72 – 1.59 (m, 2H), 1.51 (dddd, J = 13.2, 10.2, 8.0, 5.3 Hz, 1H), 1.43 – 1.27 (m, 1H), 0.93 (t, J = 7.4 Hz, 3H).

^{13}C NMR (125 MHz, $CDCl_3$): δ 139.1 (C), 129.1 (CH), 128.2 (CH), 126.8 (CH), 65.8 (CH), 58.5 (CH_2), 54.2 (CH_2), 29.8 (CH_2), 26.5 (CH_2), 21.8 (CH_2), 10.5 (CH_3).

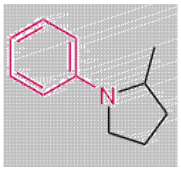

2-Methyl-1-phenylpyrrolidine 4k

The title compound was synthesized according to a modified **general procedure B** using urea hydrogen peroxide (0.068 g, 0.72 mmol), amine **1k** (0.097 g, 0.60 mmol), $B_2(OH)_4$ (0.645 g, 0.72 mmol) in TFE (0.1 M). The product was isolated after aqueous extraction to yield the title

[10] A. Agosti, S. Britto and P. Renaud, *Org. Lett.* 2008, **10**, 1417-1420.

compound as a brown oil (0.049 g, 51%). Characterization data is in good agreement with previously reported data.[11]

TLC R*f*: 0.91 in 40% EtOAc/Hexanes

^{1}H NMR (500 MHz, $CDCl_3$): δ 7.25 – 7.19 (m, 2H), 6.67 – 6.59 (m, 1H), 6.61 – 6.52 (m, 2H), 3.88 (pd, *J* = 6.2, 1.6 Hz, 1H), 3.42 (ddd, *J* = 9.2, 7.6, 2.6 Hz, 1H), 3.16 (td, *J* = 8.9, 6.8 Hz, 1H), 2.13 – 2.01 (m, 2H), 1.97 (dddd, *J* = 10.0, 7.4, 5.1, 3.0 Hz, 1H), 1.71 (dddd, *J* = 10.3, 7.8, 6.5, 2.3 Hz, 1H), 1.17 (d, *J* = 6.2 Hz, 3H).

^{13}C NMR (125 MHz, $CDCl_3$): δ 147.2 (C), 129.1 (CH), 115.1 (CH), 111.7 (CH), 53.6 (CH), 48.1 (CH_2), 33.1 (CH_2), 23.3 (CH_2), 19.4 (CH_3).

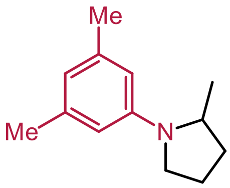

1-(3,5-Dimethylphenyl)-2-methylpyrrolidine 4l
The title compound was synthesized according to a modified **general procedure B** using urea hydrogen peroxide (0.068 g, 0.72 mmol), amine **1l** (0.114 g, 0.60 mmol), $B_2(OH)_4$ (0.118 g, 1.32 mmol) in TFE (0.1 M). The crude reaction mixture was concentrated via rotary evaporation, then diluted with DCM (50 mL) and brine (50 mL), and the phases were separated. The aqueous phase was then extracted with DCM (50 mL). The combined organic phases were dried over Na_2SO_4, then filtered before concentration via rotary evaporation. The crude reaction mixture was purified by silica plug (100% DCM) to yield the title compound a viscous orange oil (0.054 g, 47%). Characterization data is in good agreement with previously reported data.[12]

TLC R*f*: 0.84 in 20% EtOAc/Hexanes

^{1}H NMR (500 MHz, $CDCl_3$): δ 6.32 (s, 1H), 6.23 (s, 2H), 3.92 – 3.79 (m, 1H), 3.41 (t, J = 7.7 Hz, 1H), 3.19 – 3.09 (m, 1H), 2.27 (s, 6H), 2.10 – 1.90 (m, 3H), 1.73 – 1.63 (m, 1H), 1.17 (d, J = 6.3 Hz, 3H).

^{13}C NMR (100 MHz, $CDCl_3$): δ 147.4 (C), 138.7 (C), 117.2 (CH), 109.7 (CH), 53.5 (CH), 48.2 (CH_2), 33.0 (CH_2), 23.2 (CH_2), 21.7 (CH_3), 19.5 (CH_3).

[11] M. T. La, S. Kang and H.-K. Kim, *J. Org. Chem.* 2019, **84**, 6689.
[12] V. H. Tran, M. T. La, S. Kang and H.-K. Kim, *Org. Biomol. Chem.* **2020**,*18*, 5008-5016.

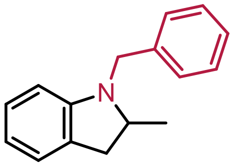

1-Benzyl-2-methylindoline 4m

The title compound was synthesized according to a modified **general procedure B** using urea hydrogen peroxide (0.068 g, 0.72 mmol), amine **1m** (0.134 g, 0.60 mmol), $B_2(OH)_4$ (0.065 g, 0.72 mmol) in TFE (0.1 M). The crude reaction mixture was concentrated via rotary evaporation, and it was purified by column chromatography (20% toluene/petroleum ether) to yield the title compound as a yellow oil (0.043 g, 32%). Characterization data is in good agreement with previously reported data.[13]

TLC R*f*: 0.3 in 20% Toluene/Petroleum ether

^{1}H NMR (500 MHz, $CDCl_3$) δ 7.31 – 7.25 (m, 2H), 7.22 (dd, *J* = 8.5, 6.8 Hz, 2H), 7.18 – 7.12 (m, 1H), 6.96 (dd, *J* = 7.1, 1.3 Hz, 1H), 6.93 – 6.86 (m, 1H), 6.54 (td, *J* = 7.3, 1.0 Hz, 1H), 6.23 (d, *J* = 7.8 Hz, 1H), 4.27 (d, *J* = 16.1 Hz, 1H), 4.11 (d, *J* = 16.1 Hz, 1H), 3.64 (ddq, *J* = 9.5, 8.6, 6.1 Hz, 1H), 3.08 (dd, *J* = 15.5, 8.6 Hz, 1H), 2.59 (ddt, *J* = 15.5, 9.5, 1.1 Hz, 1H), 1.21 (d, *J* = 6.1 Hz, 3H).

^{13}C NMR (125 MHz, $CDCl_3$) δ 152.8 (C), 139.3 (C), 128.9 (C), 128.5 (CH), 127.4 (CH), 127.4 (CH), 126.9 (CH), 124.2 (CH), 117.4 (CH), 106.9 (CH), 60.6 (CH), 51.2 (CH_2), 37.5 (CH_2), 19.7 (CH_3).

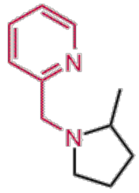

2-((2-Methylpyrrolidin-1-yl)methyl)pyridine 4n

The title compound was synthesized according to a modified **general procedure B** using urea hydrogen peroxide (0.068 g, 0.72 mmol), amine **1n** (0.106 g, 0.60 mmol), $B_2(OH)_4$ (0.118 g, 1.32 mmol) in TFE (0.1 M). The product was isolated after aqueous extraction to yield the title compound as an orange oil (0.070 g, 66%).

TLC R*f*: 0.29 in 10% MeOH/DCM

^{1}H NMR (500 MHz, $CDCl_3$): δ 8.53 (ddd, *J* = 5.0, 1.9, 0.9 Hz, 1H), 7.63 (td, *J* = 7.6, 1.8 Hz, 1H), 7.14 (dd, *J* = 7.9, 4.6 Hz, 1H), 7.41 (d, *J* = 7.8 Hz, 1H), 4.11 (d, *J* = 13.6 Hz, 1H), 3.38 (d, *J* = 13.6 Hz, 1H), 2.97 (ddd, *J* = 10.6, 8.4, 2.8 Hz, 1H), 2.50 (td, *J* = 12.3, 4.9 Hz, 1H), 2.22 (q, *J* = 8.9 Hz,

[13] A. F. G. Maier, S. Tussing, T. Schneider, U. Flörke, Z.-W. Qu, S. Grimme and J. Paradies, *Angew. Chem. Int. Ed.* 2016, **10**, 12219–12223.

1H), 1.95 (dddd, *J* = 12.5, 9.6, 7.3, 5.3 Hz, 1H), 1.80 – 1.71 (m, 1H), 1.71 – 1.58 (m, 1H), 1.47 (dddd, *J* = 12.1, 10.5, 8.5, 6.0 Hz, 1H), 1.15 (d, *J* = 6.1 Hz, 3H),

^{13}C NMR (125 MHz, $CDCl_3$): δ159.7 (C), 149.0 (CH), 136.3 (CH), 123.2 (CH), 121.8 (CH), 60.1 (CH), 60.0 (CH_2), 54.3 (CH_2), 32.6 (CH_2), 21.7 (CH_2), 19.0 (CH_3).

IR (FTIR): 3079, 2954, 2782, 1586, 1569, 1433, 1376, 1134, 991, 757 cm^{-1}.

HRMS (EI): Exact mass calcd for $C_{11}H_{16}N_2$ [M]+ 176.1313. Found: 176.1296.

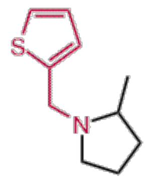

2-Methyl-1-(thiophen-2-ylmethyl)pyrrolidine 4o
The title compound was synthesized according to **general procedure B** using urea hydrogen peroxide (0.0677 g, 0.72 mmol), amine **1o** (0.109 g, 0.60 mmol), $B_2(OH)_4$ (0.118 g, 1.32 mmol) in TFE (0.1 M). The product was isolated after aqueous extraction to yield the title compound as a yellow oil (0.071 g, 65%).

TLC R*f*: 0.46 in 5% MeOH/DCM + 0.5% NH_4OH

^{1}H NMR (500 MHz, $CDCl_3$): δ 7.11 (dd, *J* = 5.1, 1.3 Hz, 1H), 6.85 (dd, *J* = 5.1, 3.4 Hz, 1H), 6.84 – 6.80 (m, 1H), 4.04 (dd, *J* = 14.0, 1.0 Hz, 1H), 3.48 (d, *J* = 14.0 Hz, 1H), 2.95 (td, *J* = 8.7, 2.7 Hz, 1H), 2.40 – 2.28 (m, 1H), 2.16 (q, *J* = 8.9 Hz, 1H), 1.84 (dddd, *J* = 12.5, 9.6, 7.3, 5.2 Hz, 1H), 1.67 (ddtd, *J* = 17.3, 10.8, 8.5, 5.2 Hz, 1H), 1.61 – 1.46 (m, 1H), 1.38 (dddd, *J* = 12.4, 10.8, 8.6, 6.0 Hz, 1H), 1.08 (d, *J* = 6.1 Hz, 3H).

^{13}C NMR (125 MHz, $CDCl_3$): δ 142.1 (C), 126.4 (CH), 125.8 (CH), 124.6 (CH), 58.7 (CH), 53.7 (CH_2), 51.6 (CH_2), 32.8 (CH_2), 21.5 (CH_2), 19.0 (CH_3).

IR (FTIR): 3064, 2959, 2780, 1457, 1374, 1172, 852, 690 cm^{-1}.

HRMS (EI): Exact mass calcd for $C_{10}H_{15}NS$ [M]+ 181.0925. Found: 181.0908.

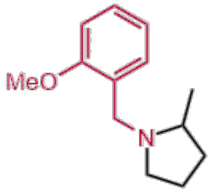

1-(2-Methoxybenzyl)-2-methylpyrrolidine 4p
The title compound was synthesized according to **general procedure B** using urea hydrogen peroxide (0.0677 g, 0.72 mmol), amine **1p** (0.123 g, 0.60 mmol), $B_2(OH)_4$ (0.118 g, 1.32 mmol) in TFE (0.1 M). The product was isolated after aqueous extraction to yield the title compound as a yellow oil (0.077 g, 63%).

TLC R*f*: 0.29 in 5% MeOH/DCM + 0.5% NH_4OH

^{1}H NMR (500 MHz, $CDCl_3$): δ 7.26 (dd, *J* = 7.4, 1.8 Hz, 1H), 7.13 (td, *J* = 7.8, 1.8 Hz, 1H), 6.83 (td, *J* = 7.4, 1.1 Hz, 1H), 6.77 (dd, *J* = 8.2, 1.1 Hz, 1H), 3.91 (d, *J* = 13.5 Hz, 1H), 3.73 (s, 3H), 3.24 (d, *J* = 13.4 Hz, 1H), 2.94 (ddd, *J* = 9.3, 8.1, 2.5 Hz, 1H), 2.41 – 2.30 (m, 1H), 2.09 (q, *J* = 9.0 Hz, 1H), 1.84 (dddd, *J* = 12.5, 9.7, 7.3, 5.2 Hz, 1H), 1.73 – 1.58 (m, 1H), 1.60 – 1.46 (m, 1H), 1.37 (dddd, *J* = 12.3, 10.8, 8.7, 5.9 Hz, 1H), 1.11 (d, *J* = 6.0 Hz, 3H).

^{13}C NMR (125 MHz, $CDCl_3$): δ 157.7 (C), 130.8 (CH), 127.9 (CH), 127.5 (C), 120.2 (CH), 110.4 (CH), 59.6 (CH), 55.4 (CH), 54.2 (CH_2), 51.5 (CH_2), 32.8 (CH_2), 21.7 (CH_2), 19.2 (CH_3).

IR (FTIR): 3033, 2960, 2873, 1589, 1490, 1237, 1030, 750 cm^{-1}.

HRMS (EI): Exact mass calcd for $C_{13}H_{19}NO$ [M]+ 205.1467. Found: 205.1450.

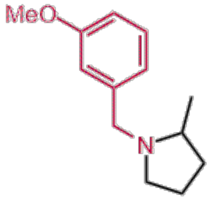

1-(3-Methoxybenzyl)-2-methylpyrrolidine 4q
The title compound was synthesized according to **general procedure B** using urea hydrogen peroxide (0.0677 g, 0.72 mmol), amine **1q** (0.123 g, 0.60 mmol), $B_2(OH)_4$ (0.118 g, 1.32 mmol) in TFE (0.1 M). The product was isolated after aqueous extraction to yield the title compound as a yellow oil (0.083 g, 67%).

TLC R*f*: 0.32 in 5% MeOH/DCM + 0.5% NH_4OH

^{1}H NMR (500 MHz, $CDCl_3$): δ 7.12 (t, *J* = 7.8 Hz, 1H), 6.85 – 6.79 (m, 2H), 6.69 (ddd, *J* = 8.2, 2.7, 1.0 Hz, 1H), 3.90 (d, *J* = 12.9 Hz, 1H), 3.71 (s, 3H), 3.04 (d, *J* = 12.9 Hz, 1H), 2.84 (ddd, *J* = 9.1,

8.0, 2.6 Hz, 1H), 2.36 – 2.25 (m, 1H), 2.02 (q, J = 9.0 Hz, 1H), 1.84 (dddd, J = 12.5, 9.7, 7.3, 5.3 Hz, 1H), 1.68 – 1.58 (m, 1H), 1.58 – 1.49 (m, 1H), 1.37 (dddd, J = 12.4, 10.8, 8.6, 5.9 Hz, 1H), 1.08 (d, J = 6.0 Hz, 3H).

^{13}C NMR (125 MHz, $CDCl_3$): δ 159.6 (C), 141.3 (C), 129.1 (CH), 121.5 (CH), 114.6 (CH), 112.2 (CH), 59.7 (CH), 58.4 (CH_2), 55.2 (CH_3), 54.1 (CH_2), 32.8 (CH_2), 21.6 (CH_2), 19.2 (CH_3).

IR (FTIR): 3009, 2957, 2779, 1585, 1486, 1260, 1148, 1042, 770, 692 cm^{-1}.

HRMS (EI): Exact mass calcd for $C_{13}H_{19}NO$ [M]+ 205.1467. Found: 205.1439.

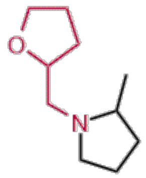

2-Methyl-1-((tetrahydrofuran-2-yl)methyl)pyrrolidine 4r

The title compound was synthesized according to a modified **general procedure B** using urea hydrogen peroxide (0.068 g, 0.72 mmol), amine **1r** (0.102 g, 0.60 mmol), $B_2(OH)_4$ (0.118 g, 1.32 mmol) in TFE (0.1 M). The product was isolated after aqueous extraction to yield the title compound as a 1.1:1 diastereomeric mixture and a yellow oil (0.081 g, 80%).

Note: No common NMR solvents tested gave adequately resolved ^{1}H NMR signals for unambiguous structural determination of the major and minor diastereomers. In $CDCl_3$, one signal was well – resolved: (*minor*) 2.91 (dd, J = 12.6, 8.1 Hz, 1H) and (*major*) 2.81 (dd, J = 12.4, 5.7 Hz, 1H). In benzene-d_6, three signals were well – resolved: (major) 4.05 (p, J = 6.5 Hz, 1H) and (minor) 3.96 (p, J = 4.8 Hz, 1H), (major) 3.30 (ddd, J = 9.8, 7.9, 2.8 Hz, 1H) and (minor) 3.22 (ddd, J = 8.9, 7.7, 3.0 Hz, 1H), (major) 2.76 (dd, J = 12.1, 6.5 Hz, 1H) and (minor) 2.85 (dd, J = 12.9, 5.8 Hz, 1H).

TLC R*f*: 0.26 in 10% MeOH/DCM

^{1}H NMR (500 MHz, benzene-d_6): δ (*major*) 4.05 (p, J = 6.5 Hz, 1H), 3.77 – 3.72 (m, 1H), 3.63 – 3.53 (m, 1H), 3.30 (ddd, J = 9.8, 7.9, 2.8 Hz, 1H), 2.76 (dd, J = 12.1, 6.5 Hz, 1H), 2.38 – 2.27 (m, 1H), 2.27 – 2.18 (m, 1H), 2.15 (q, J = 8.8 Hz, 1H), 1.79 – 1.42 (m, 7H), 1.35 – 1.27 (m, 1H), 1.06 (dd, J = 6.0 Hz, 3H). δ (*minor*) 3.96 (p, J = 4.8 Hz, 1H), 3.77 – 3.72 (m, 1H), 3.63 – 3.53 (m, 1H), 3.22 (ddd, J = 8.9, 7.7, 3.0 Hz, 1H), 2.85 (dd, J = 12.9, 5.8 Hz, 1H), 2.38 – 2.27 (m, 2H), 2.27 – 2.18 (m, 1H), 1.79 – 1.42 (m, 7H), 1.35 – 1.27 (m, 1H), 1.05 (dd, J = 6.0 Hz, 3H).

^{13}C NMR (125 MHz, $CDCl_3$): δ (*1:1 mix of two diastereomers)* 78.5 (CH), 77.5 (CH), 68.0 (CH_2), 67.8 (CH_2), 60.5 (CH, two overlapping signals), 58.9 (CH_2), 58.7 (CH_2), 54.9 (CH_2), 54.6 (CH_2), 32.4 (CH_2), 32.3 (CH_2), 30.5 (CH_2), 30.3 (CH_2), 25.5 (CH_2), 25.3 (CH_2), 21.9 (CH_2), 21.8 (CH_2), 19.0 (CH_3), 18.9 (CH_3).

IR (FTIR): 2953, 2859, 2777, 1545, 1376, 1168, 1056 cm^{-1}.

HRMS (EI): Exact mass calcd for $C_{10}H_{19}NO$ [M]+ 169.1467. Found: 169.1477.

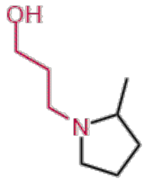

3-(2-Methylpyrrolidin-1-yl)propan-1-ol 4s
The title compound was synthesized according to **general procedure B** using urea hydrogen peroxide (0.0677 g, 0.72 mmol), amine **1s** (0.086 g, 0.60 mmol), $B_2(OH)_4$ (0.118 g, 1.32 mmol) in TFE (0.1 M). The crude reaction mixture was concentrated via rotary evaporation, then diluted with DCM (50 mL) and water (50 mL). 10 mL of 1M HCl was added, and the phases were separated. 15 mL of 1M KOH and excessive amount of NaCl were added to saturate the acidic solution. The product was isolated after aqueous extraction to yield the title compound as a yellow oil (0.053 g, 62%).

TLC R*f*: 0.46 in 40% MeOH/DCM + 1% NH_4OH

^{1}H NMR (500 MHz, $CDCl_3$) δ 4.69 (s, 1H), 3.78 – 3.68 (m, 2H), 3.26 (ddd, *J* = 9.4, 7.9, 3.2 Hz, 1H), 2.93 (td, *J* = 11.7, 4.1 Hz, 1H), 2.37 – 2.30 (m, 1H), 2.30 – 2.20 (m, 1H), 2.04 (q, *J* = 8.8 Hz, 1H), 1.91 – 1.77 (m, 2H), 1.74 – 1.54 (m, 2H), 1.49 (dp, *J* = 14.7, 3.8 Hz, 1H), 1.33 (dddd, *J* = 12.5, 10.3, 8.5, 6.2 Hz, 1H), 1.08 (d, *J* = 6.1 Hz, 3H).

^{13}C NMR (125 MHz, $CDCl_3$) δ 64.6 (CH_2), 60.4 (CH), 54.2 (CH_2), 54.0 (CH_2), 32.6 (CH_2), 29.1 (CH_2), 21.6 (CH_2), 18.9 (CH_3).

IR (FTIR): 3335, 2955, 2868, 2796, 1374, 1061 cm^{-1}.

HRMS (EI): Exact mass calcd for $C_8H_{17}NO$ [M]+ 143.1310. Found: 143.1330.

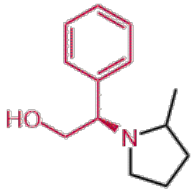

(2*R*)-2-(2-methylpyrrolidin-1-yl)-2-phenylethanol 4t
The title compound was synthesized according to a modified **general procedure B** using urea hydrogen peroxide (0.055 g, 0.60 mmol), amine **1t** (0.102 g, 0.50 mmol), $B_2(OH)_4$ (0.099 g, 1.10 mmol) in TFE (0.1 M). The product was isolated after aqueous extraction to yield the title

compound as a 2.6:1 diastereomeric mixture as a yellow oil (0.081 g, 79%). Characterization data is in good agreement with previously reported data for the major diastereomer.[14]

TLC R*f*: 0.31 and 0.53 in 10% MeOH/DCM

^{1}H NMR (500 MHz, benzene-d_6): δ (*major*) 7.15 – 7.10 (m, 3H), 6.95 – 6.88 (m, 2H), 3.98 – 3.84 (m, 2H), 3.68 (dd, *J* = 9.7, 4.5 Hz, 1H), 2.71 – 2.64 (m, 1H), 2.43 (ap h, *J* = 6.0 Hz, 1H), 2.02 (q, *J* = 8.5 Hz, 1H), 1.48 – 1.39 (m, 2H), 1.20 – 1.07 (m, 2H), 1.00 (d, *J* = 6.0 Hz, 3H). δ (*minor*) 7.31 – 7.20 (m, 2H), 7.15 – 7.10 (m, 3H), 3.98 – 3.84 (m, 1H), 3.75 (dd, *J* = 10.8, 5.7 Hz, 1H), 3.59 (t, *J* = 6.0 Hz, 1H), 2.89 – 2.80 (m, 1H), 2.78 – 2.73 (m, 1H), 2.50 (dt, *J* = 9.1, 7.6 Hz, 1H), 1.63 – 1.48 (m, 2H), 1.37 – 1.27 (m, 1H), 1.20 – 1.07 (m, 1H), 0.83 (d, *J* = 6.2 Hz, 3H).

^{13}C NMR (125 MHz, $CDCl_3$): δ (*major*) 134.9 (C), 129.2 (CH), 128.1 (CH), 127.8 (CH), 67.5 (CH), 62.0 (CH), 60.9 (CH_2), 54.5 (CH_2), 45.3 (CH_2), 32.0 (CH_2), 21.5 (CH_2), 19.1 (CH_3). δ (*minor*) 138.4 (C), 129.0 (CH), 128.4 (CH), 127.8 (CH), 77.2 (CH), 63.4 (CH_2), 55.3 (CH), 52.5 (CH_2), 33.0 (CH_2), 22.4 (CH_2), 20.2 (CH_3).

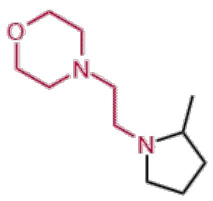

4-(2-(2-Methylpyrrolidin-1-yl)ethyl)morpholine 4u

The title compound was synthesized according to a modified **general procedure B** using urea hydrogen peroxide (0.124 g, 1.32 mmol), amine **1u** (0.119 g, 0.60 mmol), $B_2(OH)_4$ (0.172 g, 1.92 mmol) in TFE (0.1 M). The product was isolated after aqueous extraction to yield the title compound as a yellow oil (0.055 g, 46%).

TLC R*f*: 0.25 in 10% MeOH/DCM

^{1}H NMR (500 MHz, $CDCl_3$): δ 3.71 (t, *J* = 4.7 Hz, 4H), 3.17 (td, *J* = 8.7, 2.8 Hz, 1H), 2.97 (ddd, *J* = 11.7, 8.9, 6.5 Hz, 1H), 2.56 – 2.45 (m, 6H), 2.29 (dt, *J* = 8.5, 6.3 Hz, 1H), 2.19 (ddd, *J* = 11.7, 8.5, 6.4 Hz, 1H), 2.11 (q, *J* = 8.9 Hz, 1H), 1.90 (dddd, *J* = 12.4, 9.7, 7.2, 5.2 Hz, 1H), 1.77 (dddd, *J* = 17.3, 10.8, 8.5, 5.1 Hz, 1H), 1.69 (dddd, *J* = 12.5, 9.6, 6.3, 3.2 Hz, 1H), 1.41 (dddd, *J* = 12.4, 10.6, 8.7, 6.1 Hz, 1H), 1.10 (d, *J* = 6.1 Hz, 3H).

^{13}C NMR (125 MHz, $CDCl_3$): δ 66.9 (CH_2), 60.5 (CH), 58.1 (CH_2), 54.4 (CH_2), 54.2 (CH_2), 51.2 (CH_2), 32.4 (CH_2), 21.7 (CH_2), 18.9 (CH_3).

IR (FTIR): 2936, 2915, 2863, 1637, 1452, 1124, 994, 906, 763, 697 cm^{-1}.

---

[14] J. M. Andres, I. Herraiz-Sierra, R. Pedrosa and A. Perez-Encabo, *Eur. J. Org. Chem.* 2000, **9**, 1719.

HRMS (EI): Exact mass calcd for $C_{11}H_{22}N_2O$ [M]+ 198.1732. Found: 198.1760.

Gram – scale reaction

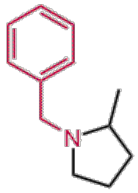

1-Benzyl-2-methylpyrrolidine 4a
The title compound was synthesized according to a modified **general procedure B** using urea hydrogen peroxide (0.678 g, 7.20 mmol), amine **1a** (1.05 g, 6.00 mmol), $B_2(OH)_4$ (0.646 g, 7.20 mmol) in TFE (55 mL, 0.11 M). The reaction was stirred in a 55 mL volume screw cap vial (no headspace). The product was isolated after aqueous extraction to yield the title compound as a pale yellow oil (0.904 g, 86%). Characterization data is in good agreement with previously reported data.[15]

^{1}H NMR (500 MHz, $CDCl_3$): δ 7.34 – 7.29 (m, 4H), 7.25 – 7.22 (m, 1H), 3.96 (d, *J* = 12.8 Hz, 1H), 3.08 (d, *J* = 12.8 Hz, 1H), 2.85 (td, *J* = 10.8, 2.6 Hz, 1H), 2.32 (dq, *J* = 13.5, 6.2 Hz, 1H), 2.04 (q, *J* = 9.0 Hz, 1H), 1.88 (dddd, *J* = 12.5, 9.7, 7.3, 5.3 Hz, 1H), 1.71 – 1.62 (m, 1H), 1.64 (dddd, *J* = 13.8, 8.7, 6.3, 3.3 Hz, 1H), 1.40 (dddd, *J* = 12.5, 10.8, 8.6, 5.8 Hz, 1H), 1.11 (d, *J* = 6.0 Hz, 3H).

^{13}C NMR (125 MHz, $CDCl_3$): δ 139.7 (C), 129.1 (CH), 128.1 (CH), 126.7 (CH), 59.6 (CH), 58.3 (CH2), 54.0 (CH2), 32.7 (CH2), 21.5 (CH2), 19.1 (CH3).

1-Benzyl-2-methylpyrrolidine 4a·HCl
To a stirred solution of pyrrolidine **4a** in 10 mL ether was added 1.4 mL of 4M HCl in dioxane dropwise and solids precipitated immediately. The product was isolated after filtration, washing with hexanes, to yield the title compound as a 9.5:1 diastereomeric mixture as a white solid (0.931 g, 73%).

TLC R*f*: 0.31 in 10% MeOH/DCM

^{1}H NMR (500 MHz, $CDCl_3$): δ (*major*) 12.45 (br s, 1H), 7.61 – 7.57 (m, 2H), 7.47 – 7.41 (m, 3H), 4.33 (dd, *J* = 13.2, 4.5 Hz, 1H), 4.06 (dd, *J* = 13.3, 5.4 Hz, 1H), 3.62 (dq, *J* = 11.7, 6.8, 5.1 Hz, 1H), 3.23 (p, *J* = 7.4 Hz, 1H), 2.83 (p, *J* = 8.5 Hz, 1H), 2.26 – 2.14 (m, 2H), 2.11 – 2.03 (m, 1H), 1.86 (tt, *J* = 13.8, 8.7 Hz, 1H), 1.86 (tt, *J* = 13.8, 8.7 Hz, 1H).

^{13}C NMR (125 MHz, $CDCl_3$): δ (*major*) 131.2 (CH), 130.0 (C), 129.29 (CH), 128.9 (CH), 62.6 (CH), 55.6 (CH_2), 52.4 (CH_2), 31.3 (CH_2), 20.9 (CH_2), 15.8 (CH_3).

[15] J. Zhang and S. Chang, *J. Am. Chem. Soc.* 2020, **142**, 12585.

NMR Spectra

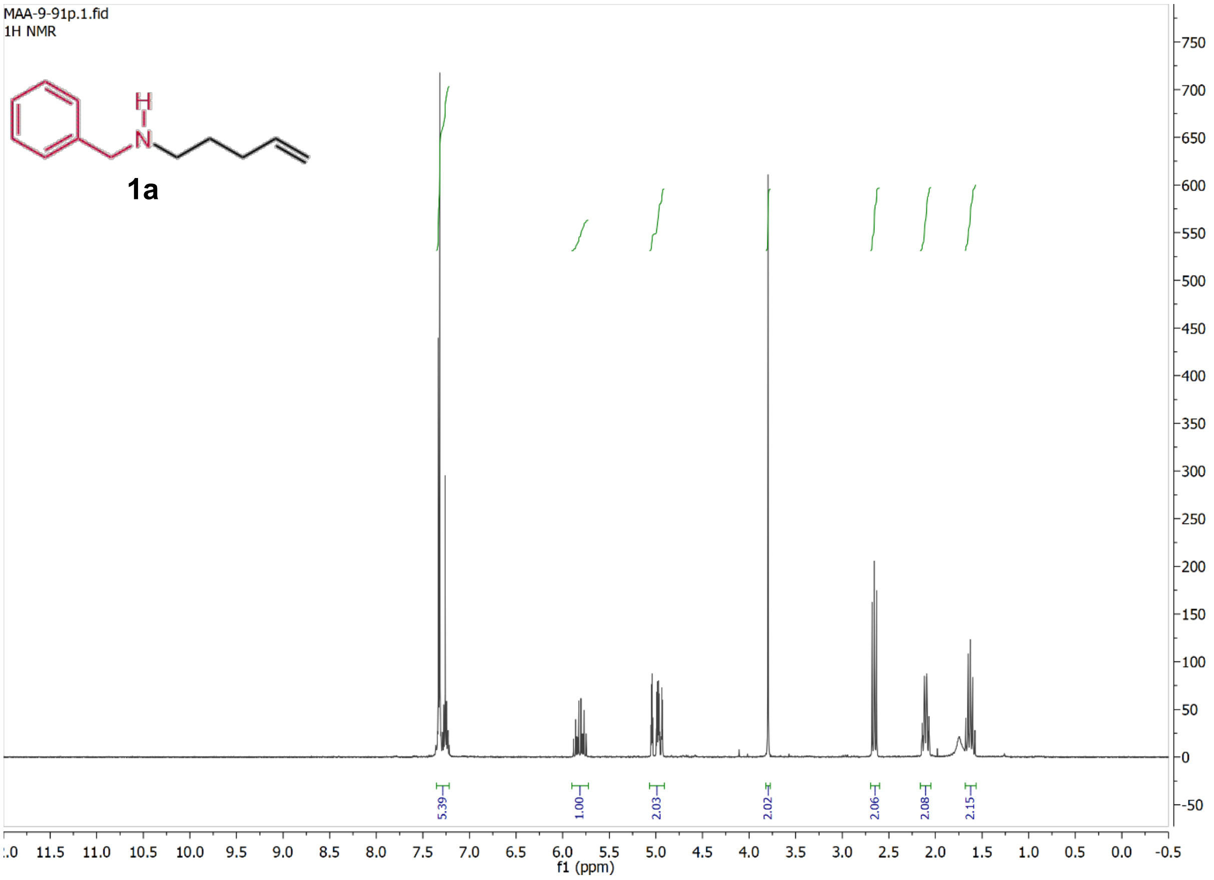

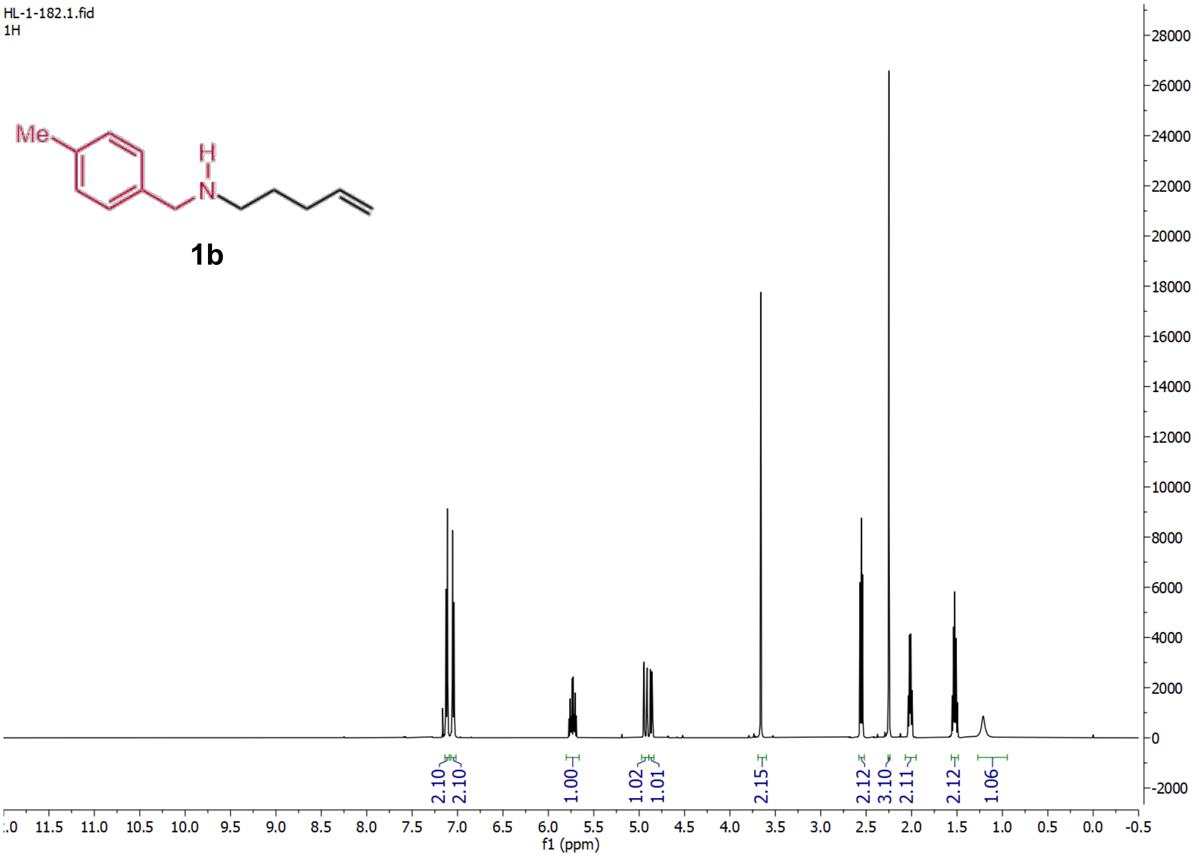
HL-1-182.1.fid
1H
Me
H
N
1b
2.10
2.10
1.00
1.02
1.01
2.15
2.12
3.10
2.11
2.12
1.06
f1 (ppm)

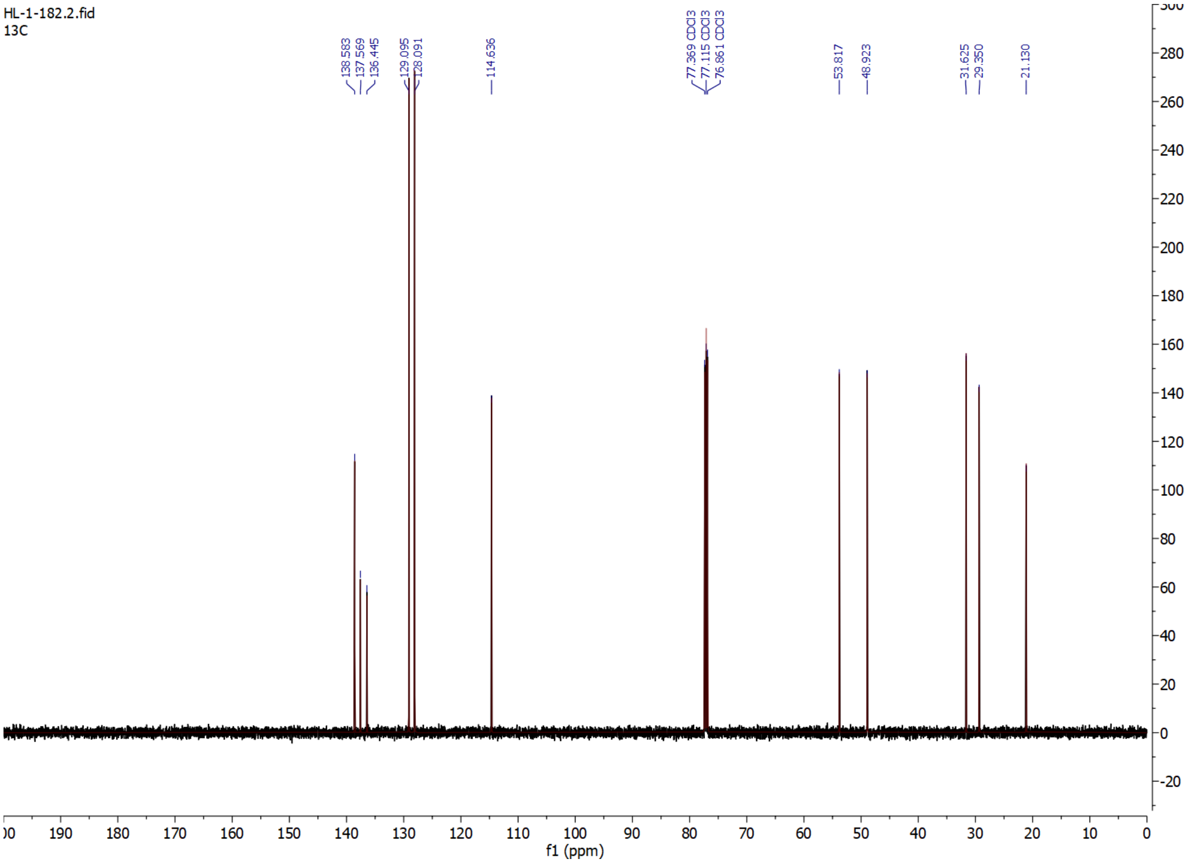
HL-1-182.2.fid
13C
138.583
137.569
136.445
129.095
128.091
114.636
77.369 CDCl3
77.115 CDCl3
76.861 CDCl3
53.817
48.923
31.625
29.350
21.130
f1 (ppm)

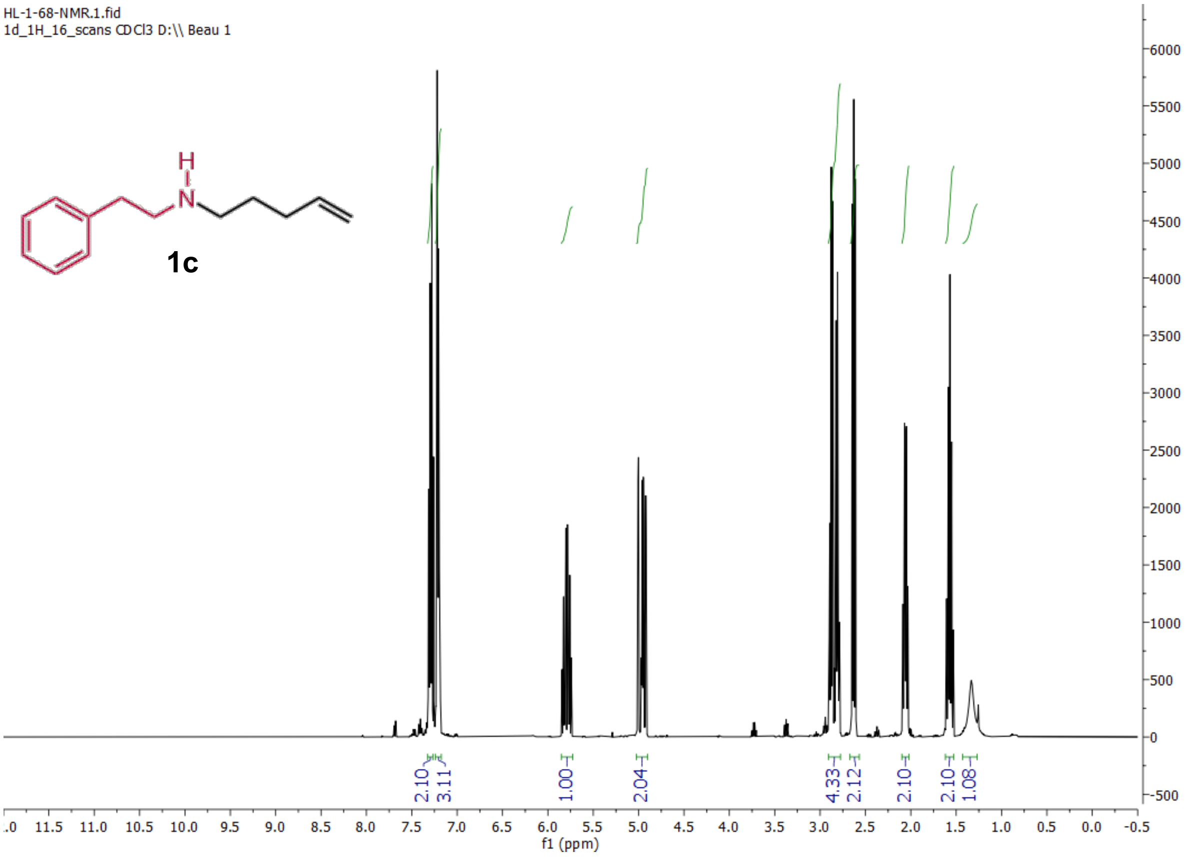
HL-1-68-NMR.1.fid
1d_1H_16_scans CDCl3 D:\\ Beau 1
1c
2.10
3.11
1.00
2.04
4.33
2.12
2.10
2.10
1.08
f1 (ppm)

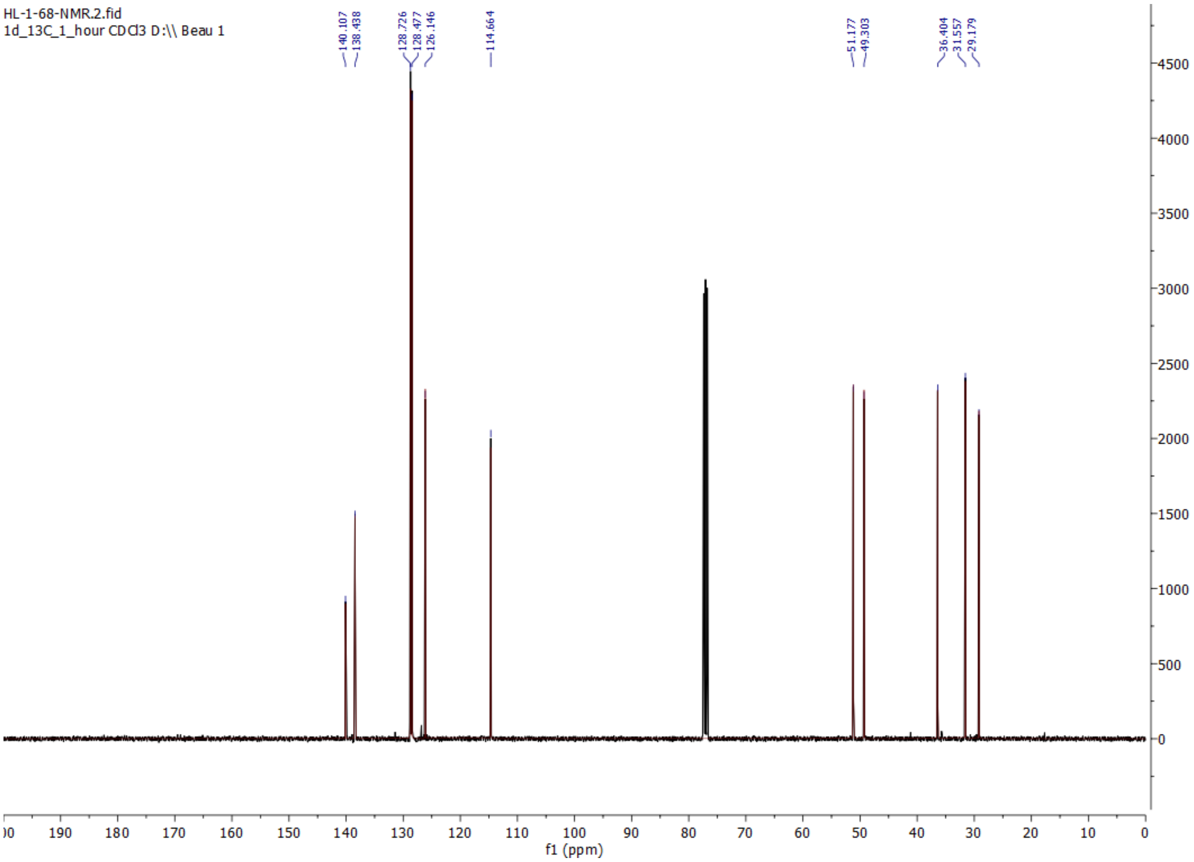
HL-1-68-NMR.2.fid
1d_13C_1_hour CDCl3 D:\\ Beau 1
140.107
138.438
128.726
128.477
126.146
114.664
51.177
49.303
36.404
31.557
29.179
f1 (ppm)

HL-1-188-dry2.1.fid
1H

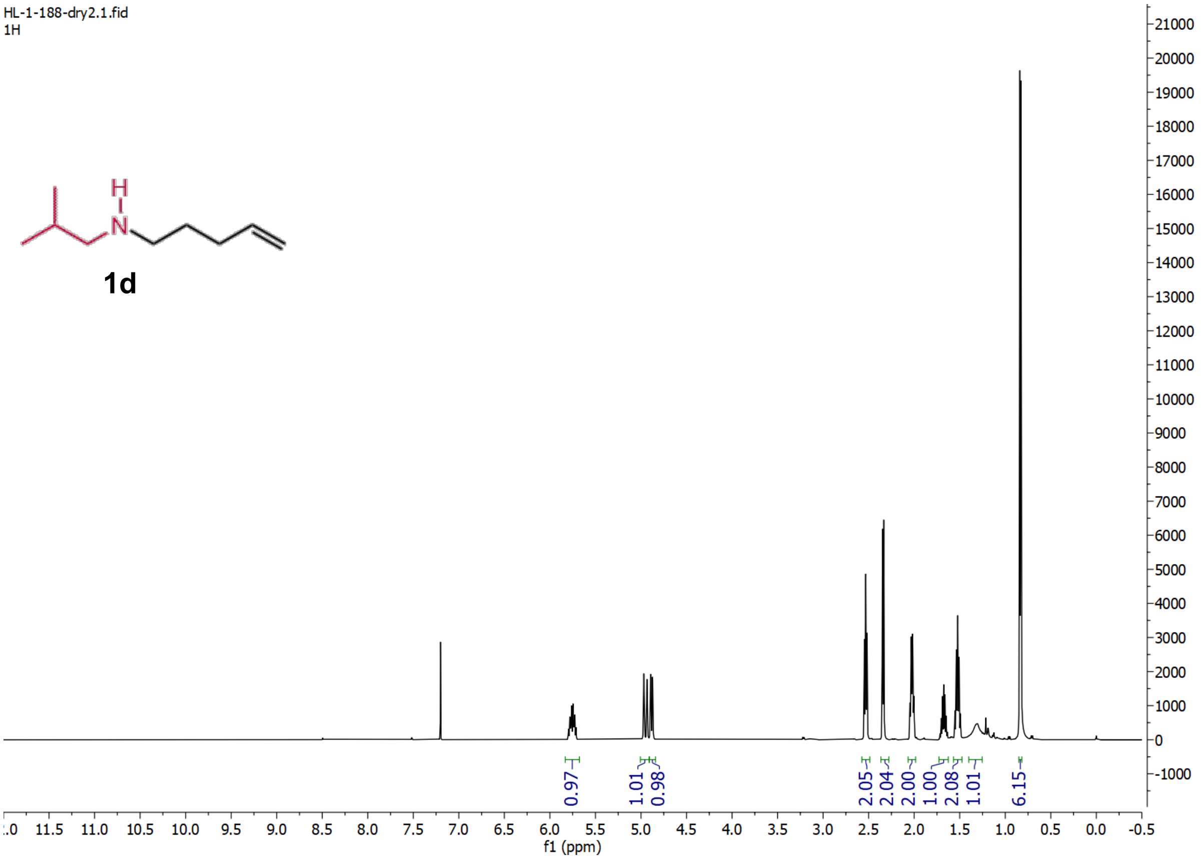

HL-1-188-dry2.2.fid
13C

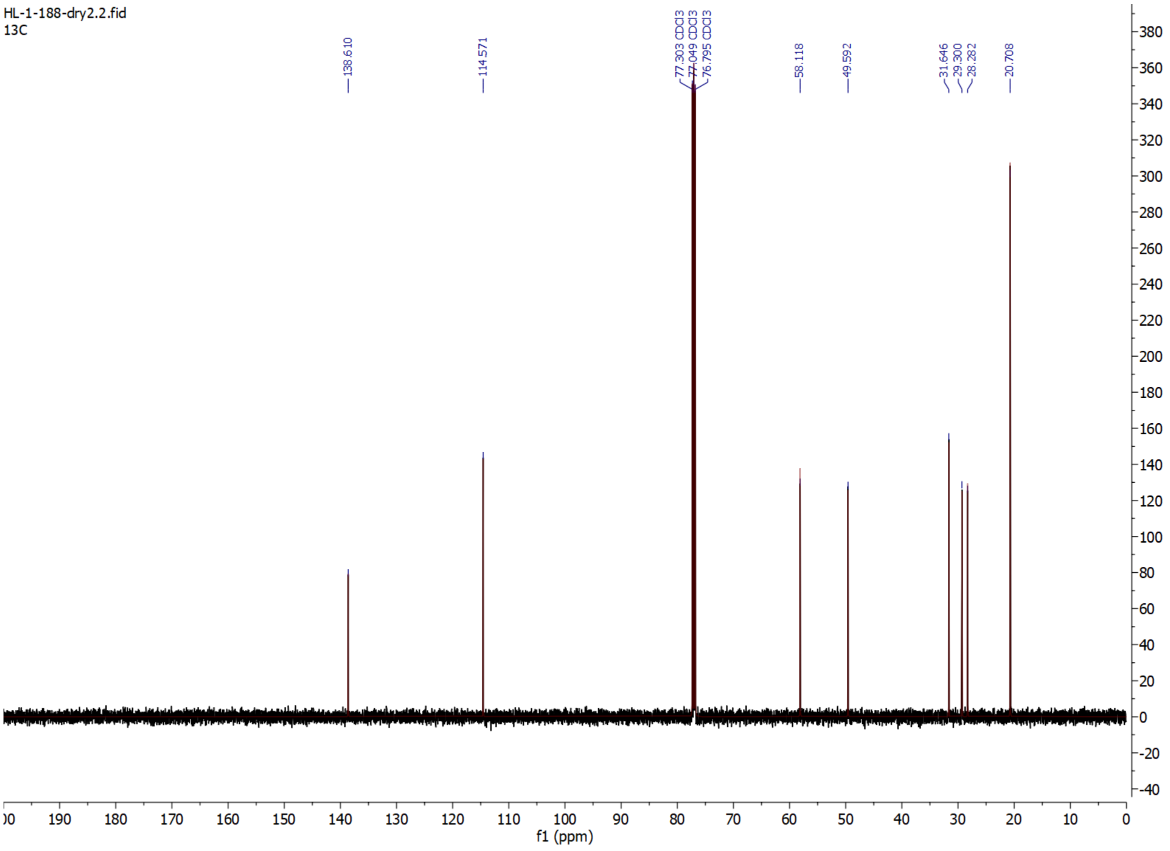

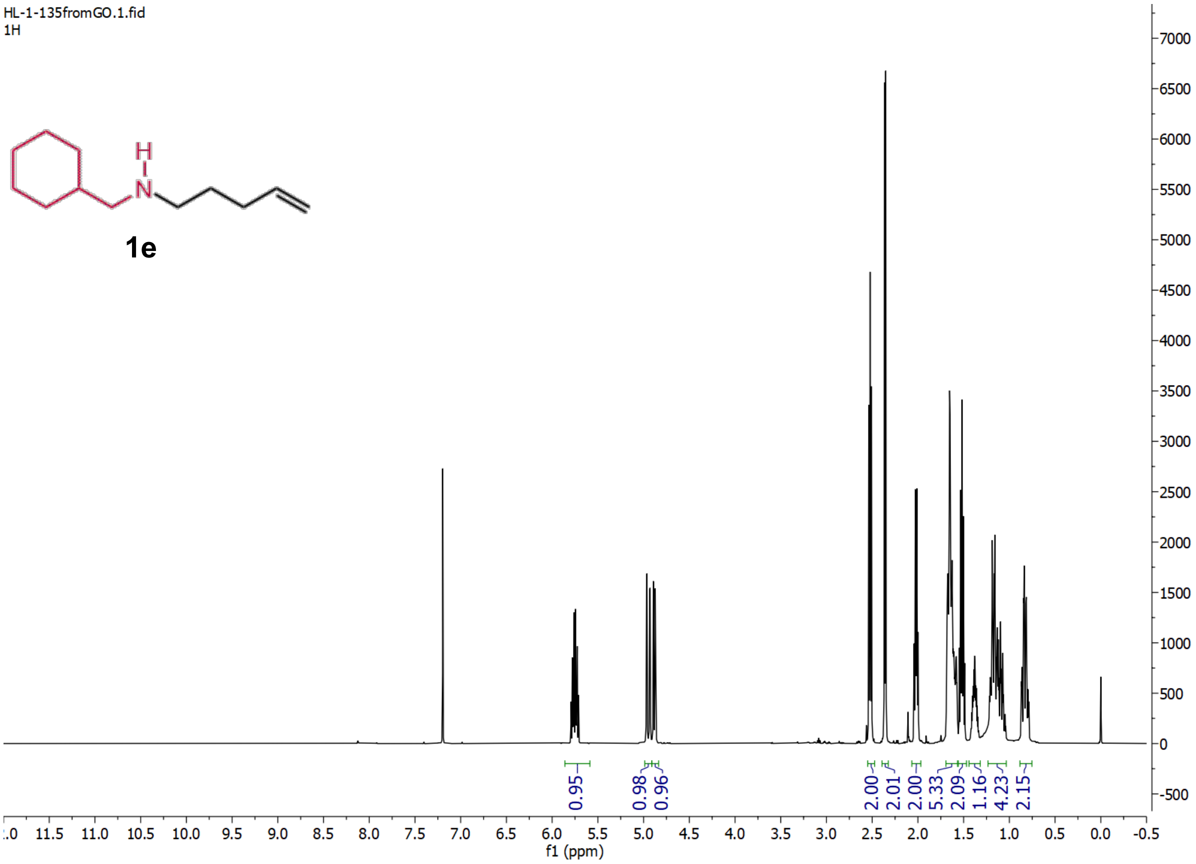
HL-1-135fromGO.1.fid
1H
1e
0.95
0.98
0.96
2.00
2.01
2.00
5.33
2.09
1.16
4.23
2.15
f1 (ppm)

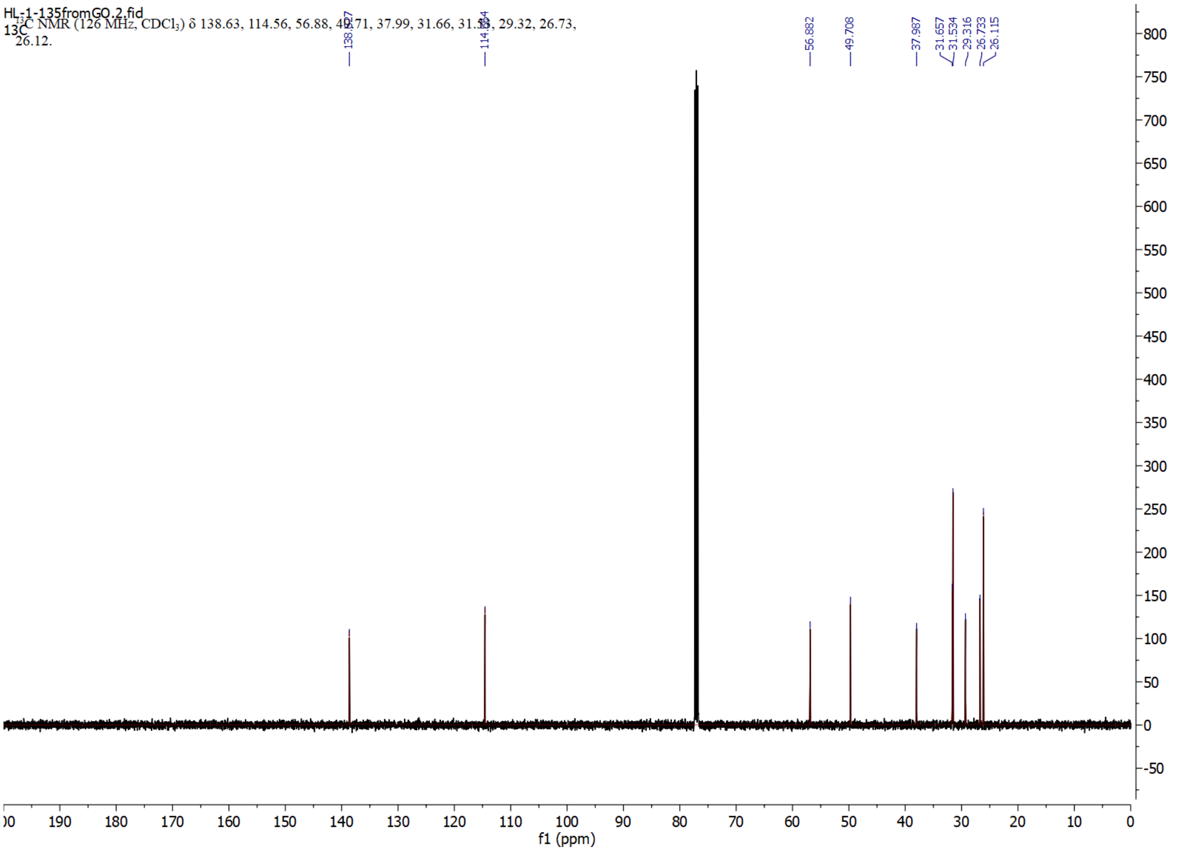
HL-1-135fromGO.2.fid
13C
^{13}C NMR (126 MHz, $CDCl_3$) δ 138.63, 114.56, 56.88, 49.71, 37.99, 31.66, 31.53, 29.32, 26.73, 26.12.
56.882
49.708
37.987
31.657
31.534
29.316
26.733
26.115
f1 (ppm)

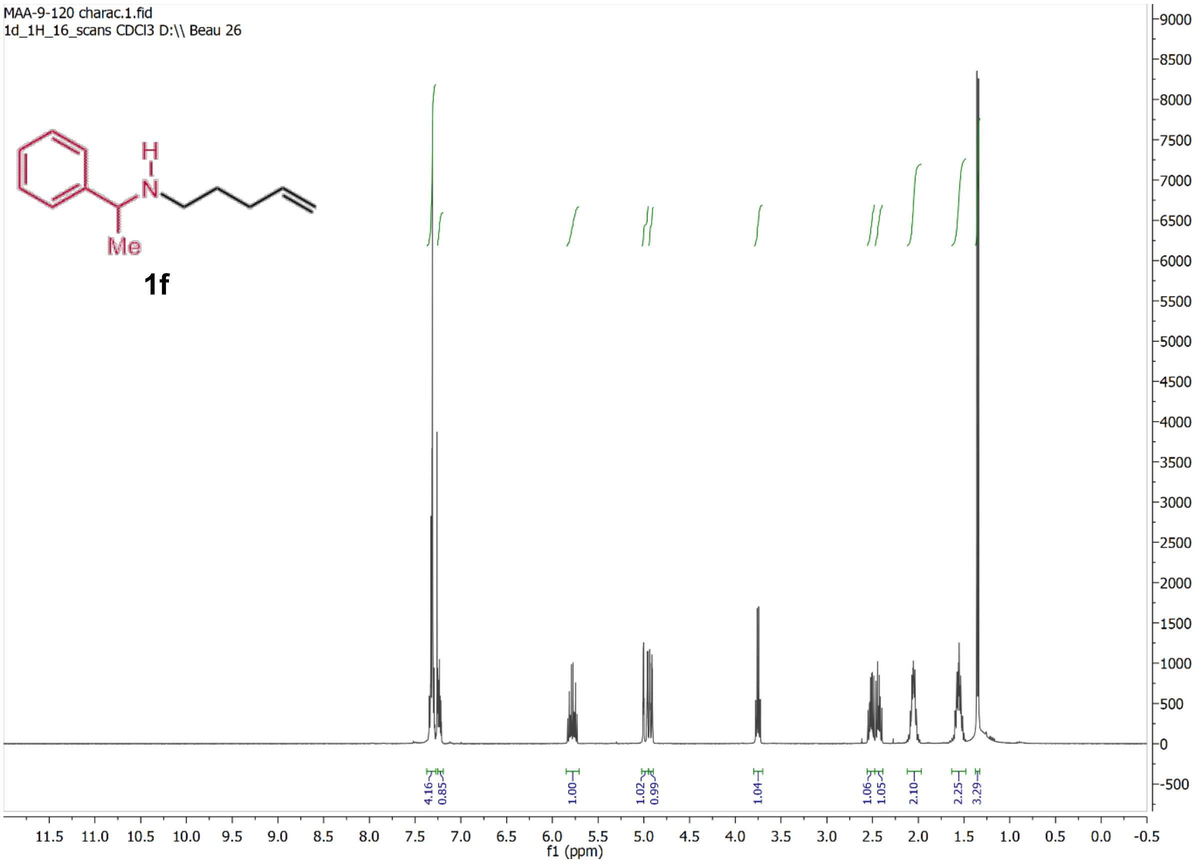
MAA-9-120 charac.1.fid
1d_1H_16_scans CDCl3 D:\\ Beau 26
H
N
Me
1f
4.16
0.85
1.00
1.02
0.99
1.04
1.06
1.05
2.10
2.25
3.29
f1 (ppm)

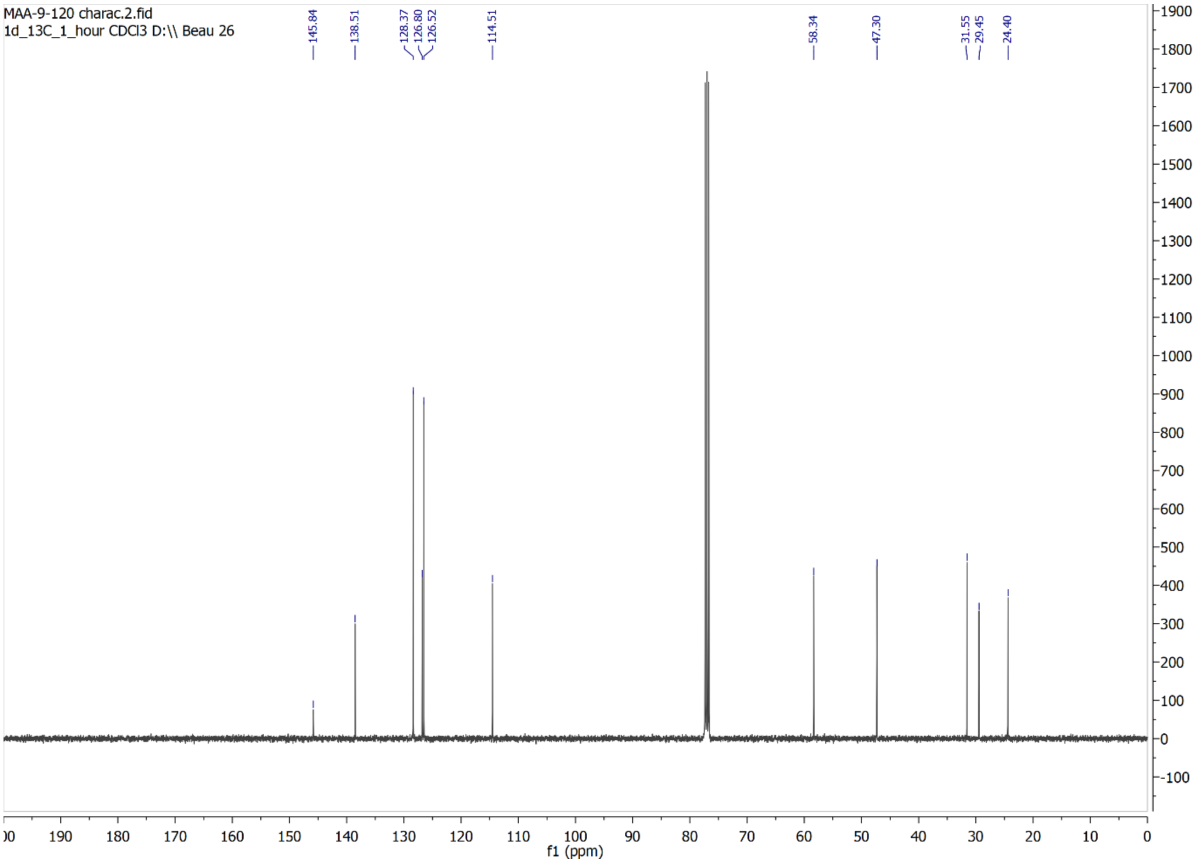
MAA-9-120 charac.2.fid
1d_13C_1_hour CDCl3 D:\\ Beau 26
145.84
138.51
128.37
126.80
126.52
114.51
58.34
47.30
31.55
29.45
24.40
f1 (ppm)

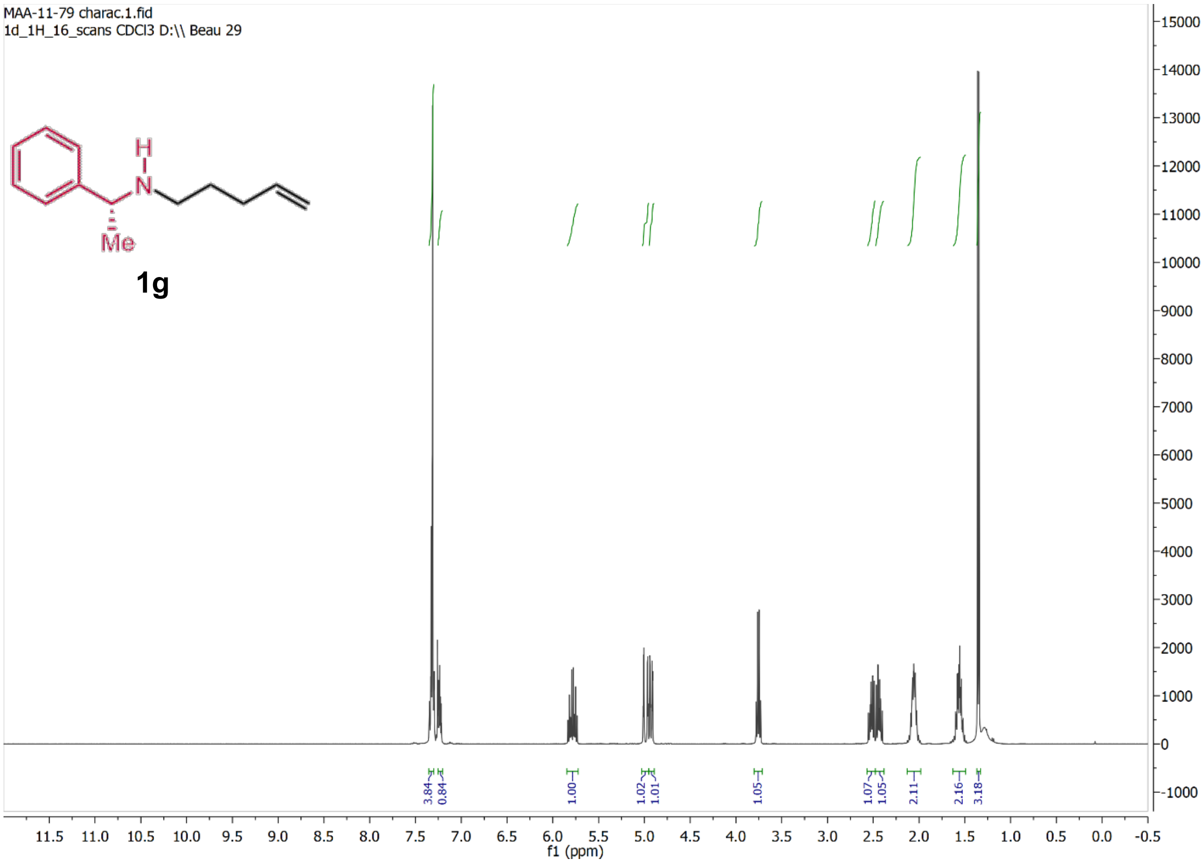
MAA-11-79 charac.1.fid
1d_1H_16_scans CDCl3 D:\\ Beau 29
1g
3.84
0.84
1.00
1.02
1.01
1.05
1.07
1.05
2.11
2.16
3.18
f1 (ppm)

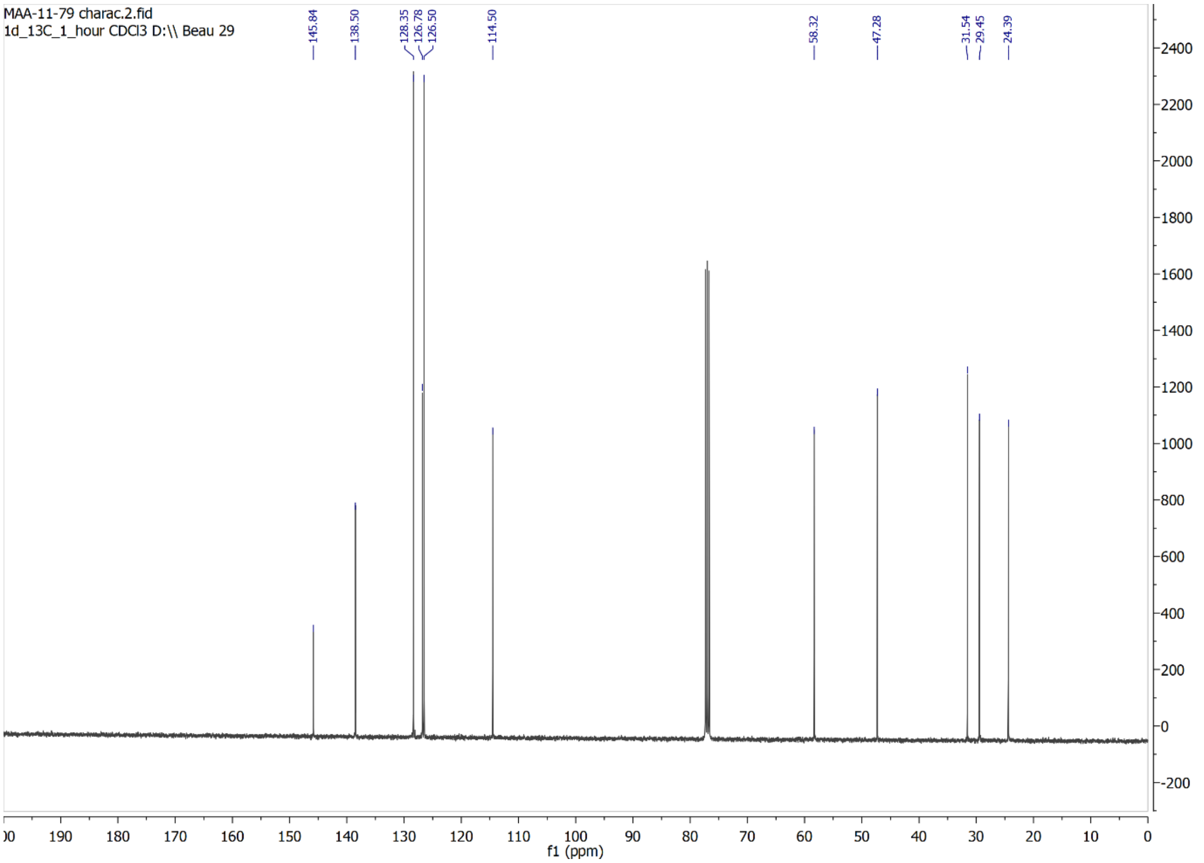
MAA-11-79 charac.2.fid
1d_13C_1_hour CDCl3 D:\\ Beau 29
145.84
138.50
128.35
126.78
126.50
114.50
58.32
47.28
31.54
29.45
24.39
f1 (ppm)

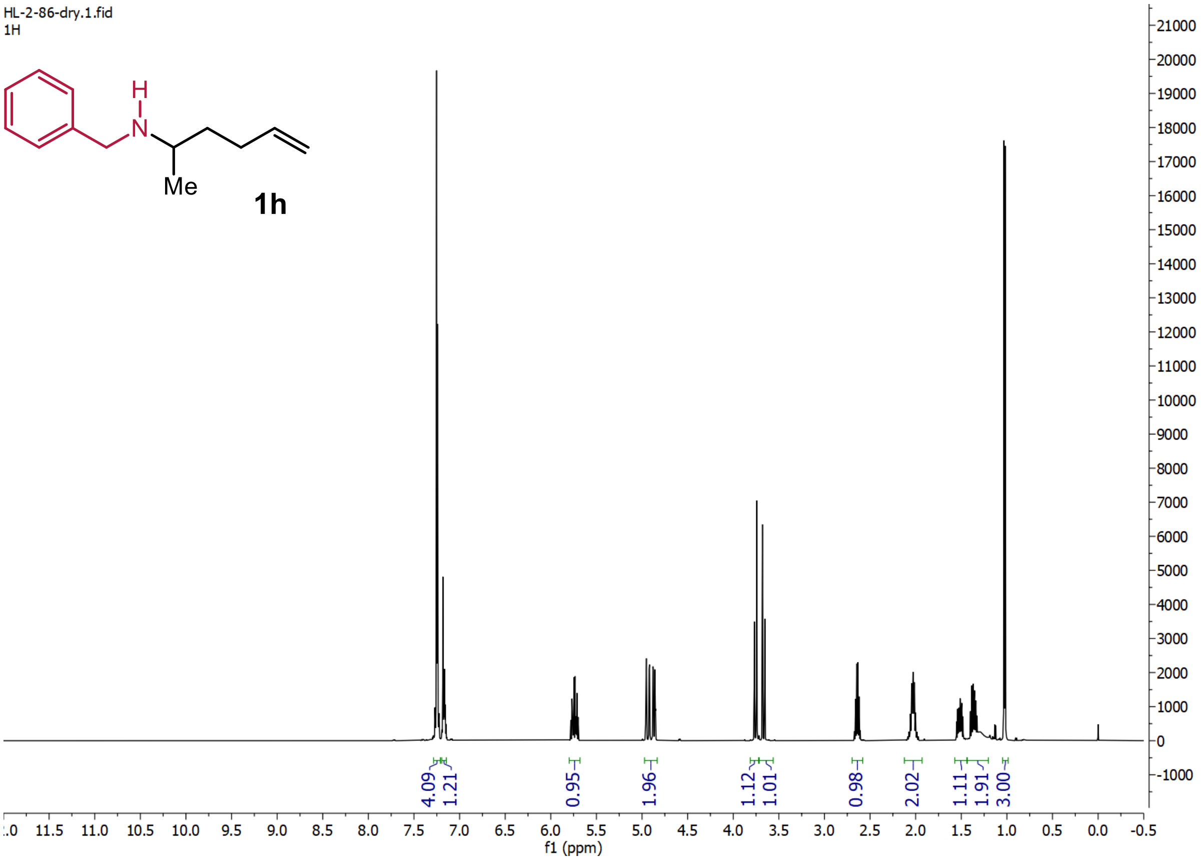
HL-2-86-dry.1.fid
1H
H
N
Me
1h
4.09
1.21
0.95
1.96
1.12
1.01
0.98
2.02
1.11
1.91
3.00
f1 (ppm)

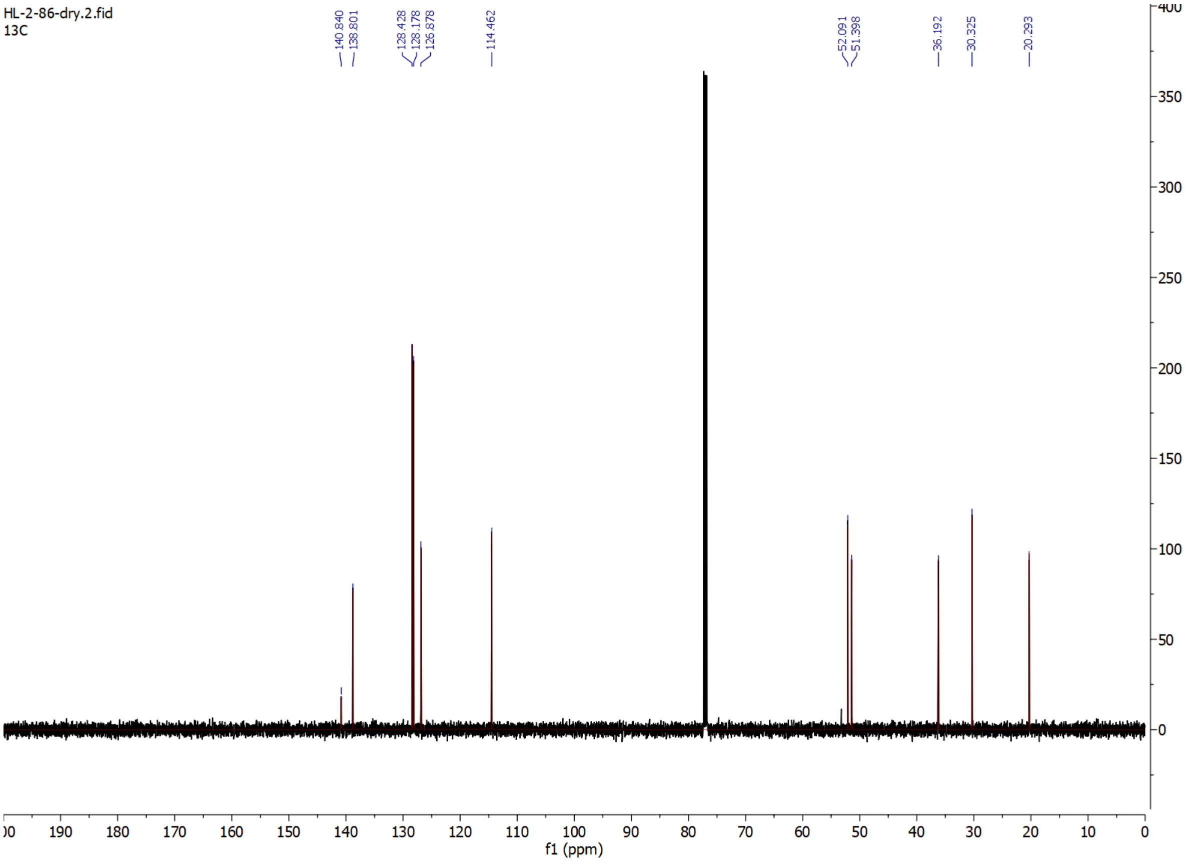
HL-2-86-dry.2.fid
13C
140.840
138.801
128.428
128.178
126.878
114.462
52.091
51.398
36.192
30.325
20.293
f1 (ppm)

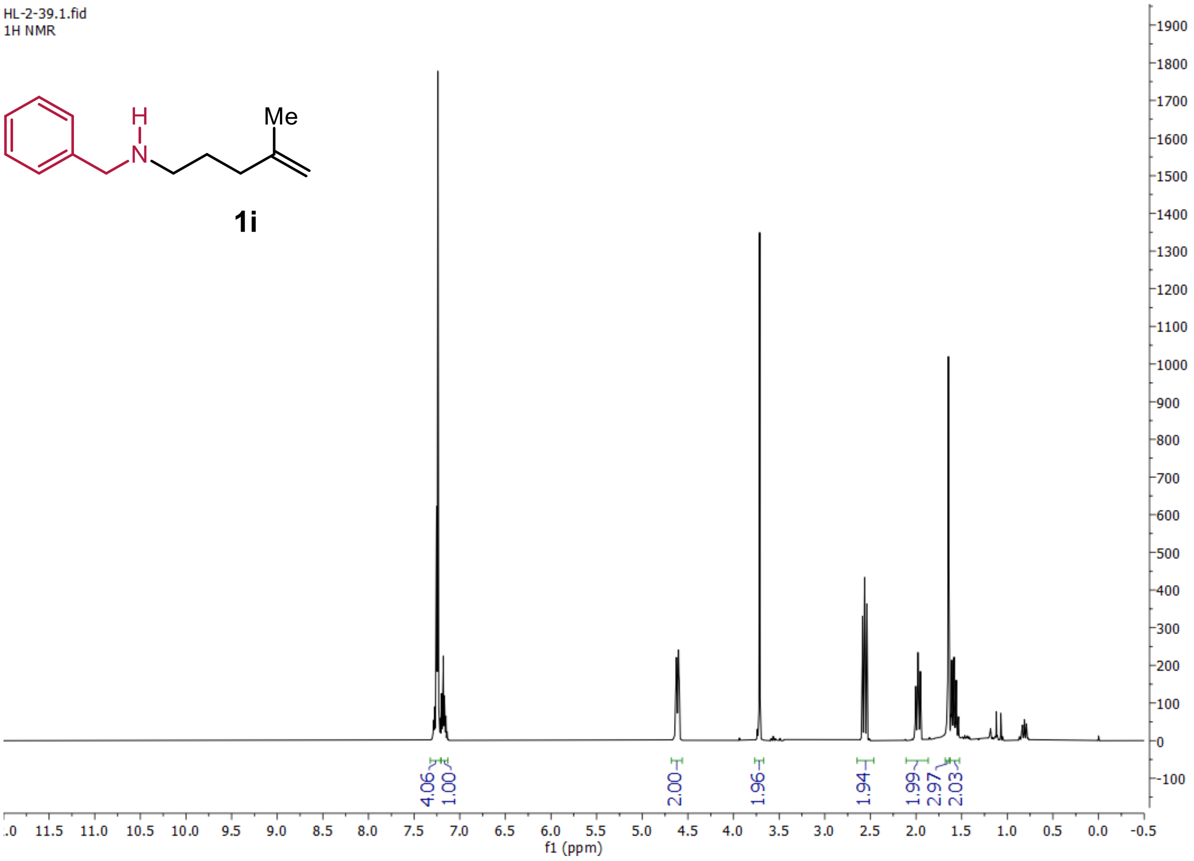
HL-2-39.1.fid
1H NMR
H
N
Me
1i
4.06
1.00
2.00
1.96
1.94
1.99
2.97
2.03
f1 (ppm)

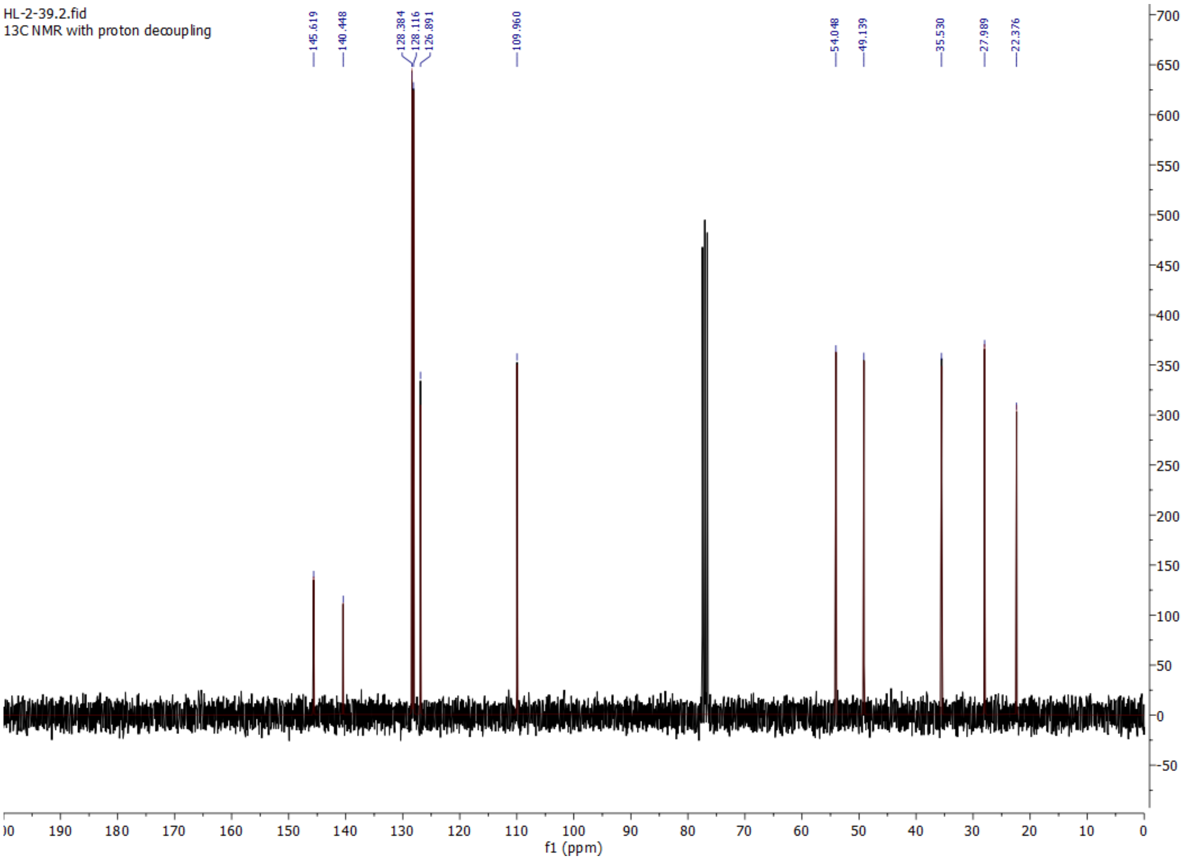
HL-2-39.2.fid
13C NMR with proton decoupling
145.619
140.448
128.384
128.116
126.891
109.960
54.048
49.139
35.530
27.989
22.376
f1 (ppm)

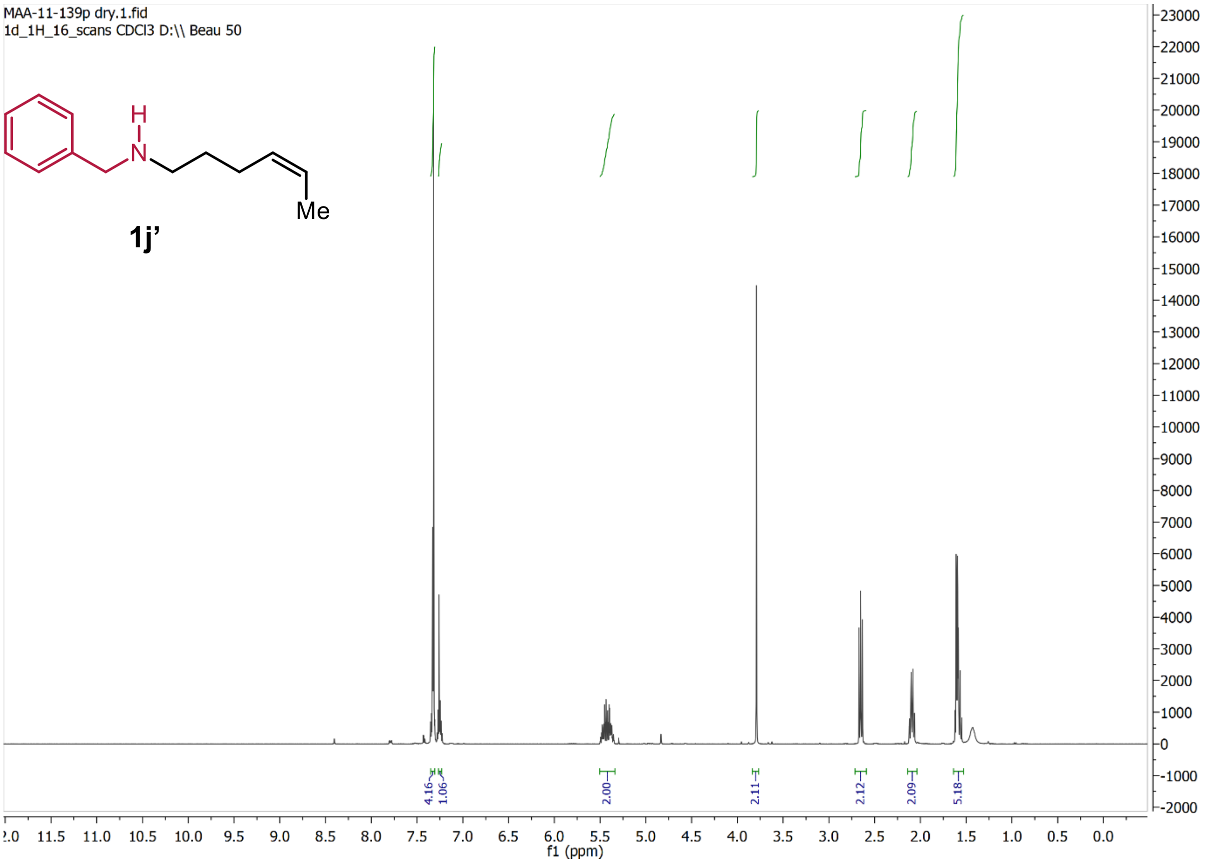
MAA-11-139p dry.1.fid
1d_1H_16_scans CDCl3 D:\\ Beau 50
H
N
Me
1j'
4.16
1.06
2.00
2.11
2.12
2.09
5.18
f1 (ppm)

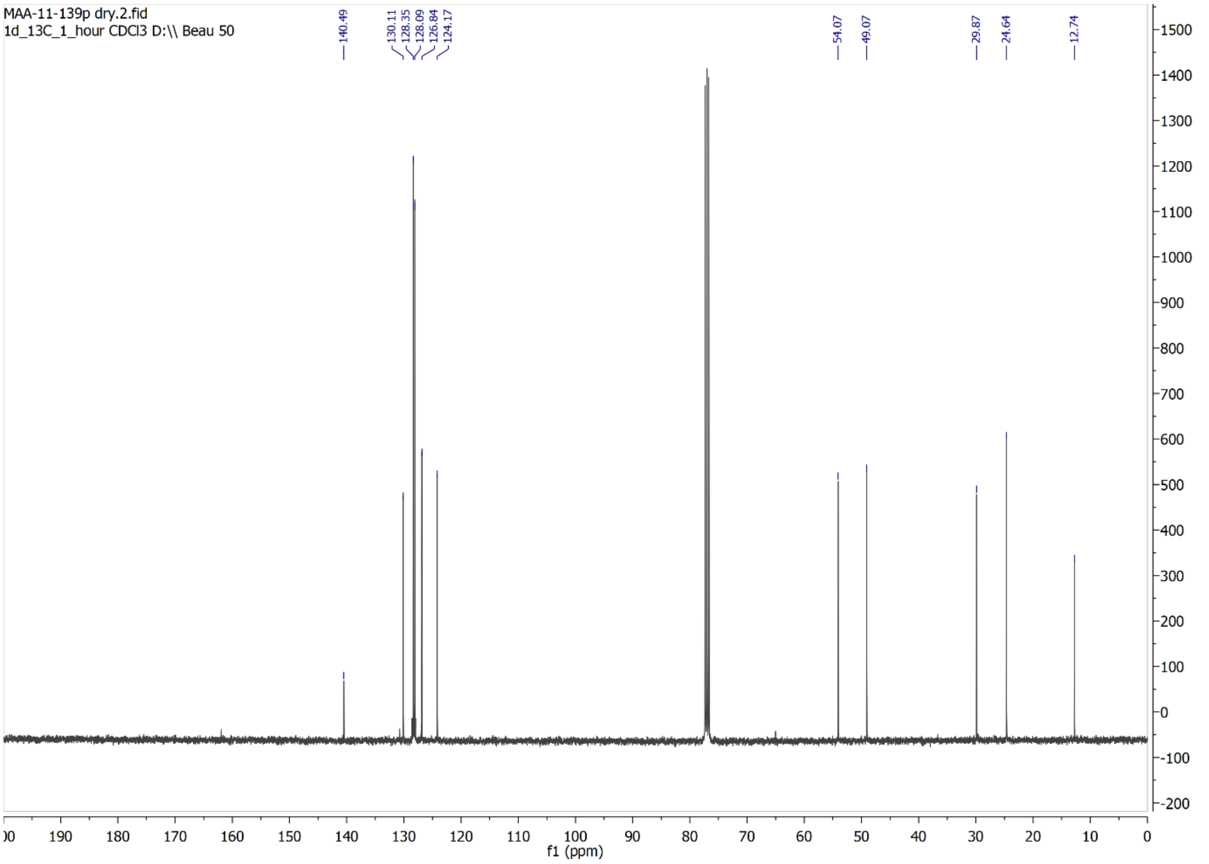
MAA-11-139p dry.2.fid
1d_13C_1_hour CDCl3 D:\\ Beau 50
140.49
130.11
128.35
128.09
126.84
124.17
54.07
49.07
29.87
24.64
12.74
f1 (ppm)

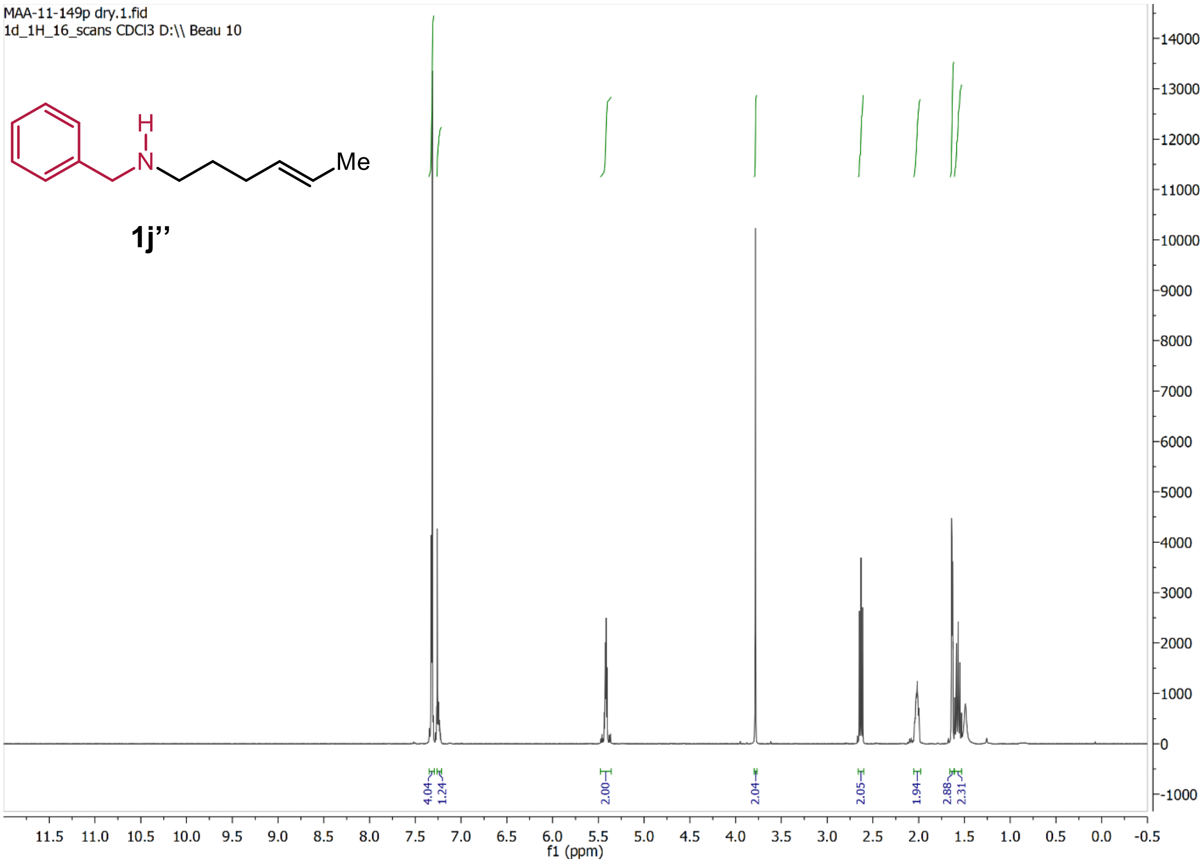
MAA-11-149p dry.1.fid
1d_1H_16_scans CDCl3 D:\\ Beau 10
H
N
Me
1j''
4.04
1.24
2.00
2.04
2.05
1.94
2.88
2.31
f1 (ppm)

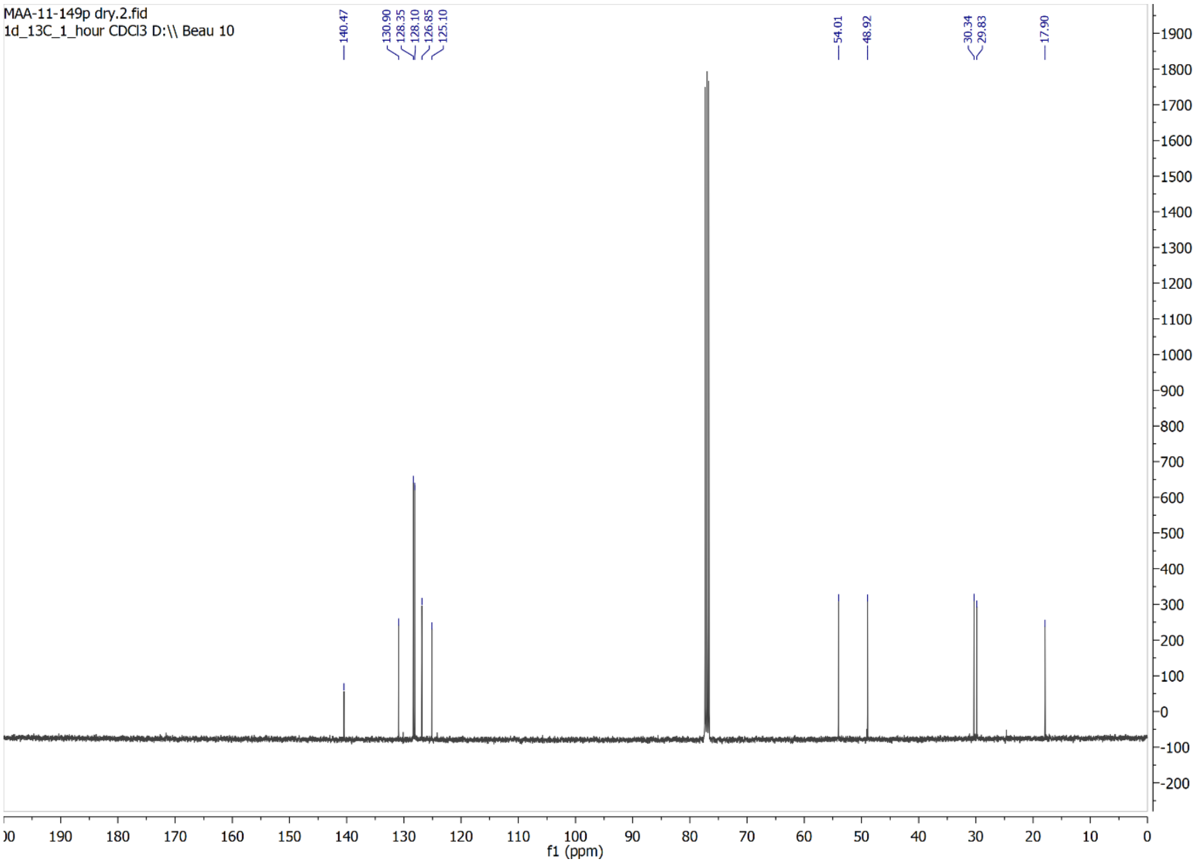
MAA-11-149p dry.2.fid
1d_13C_1_hour CDCl3 D:\\ Beau 10
140.47
130.90
128.35
128.10
126.85
125.10
54.01
48.92
30.34
29.83
17.90
f1 (ppm)

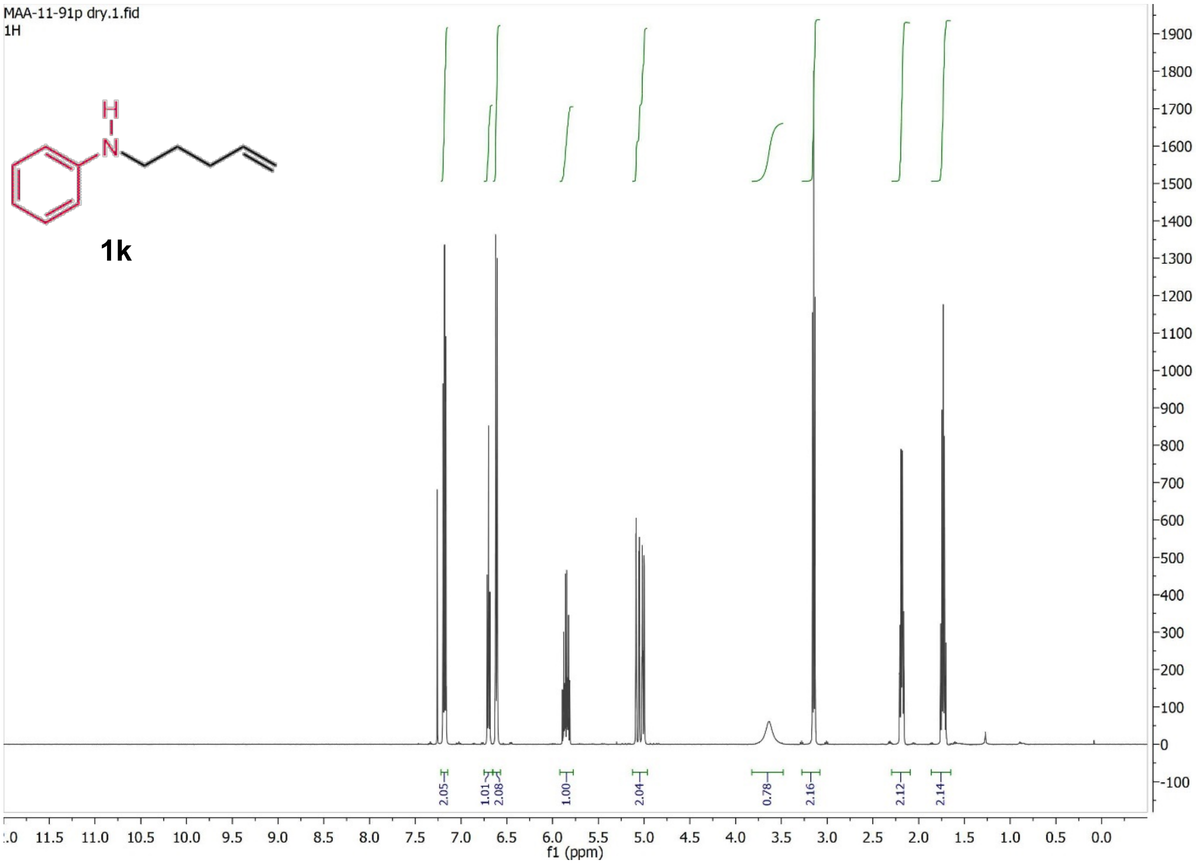
MAA-11-91p dry.1.fid
1H
H
N
1k
2.05
1.01
2.08
1.00
2.04
0.78
2.16
2.12
2.14
f1 (ppm)

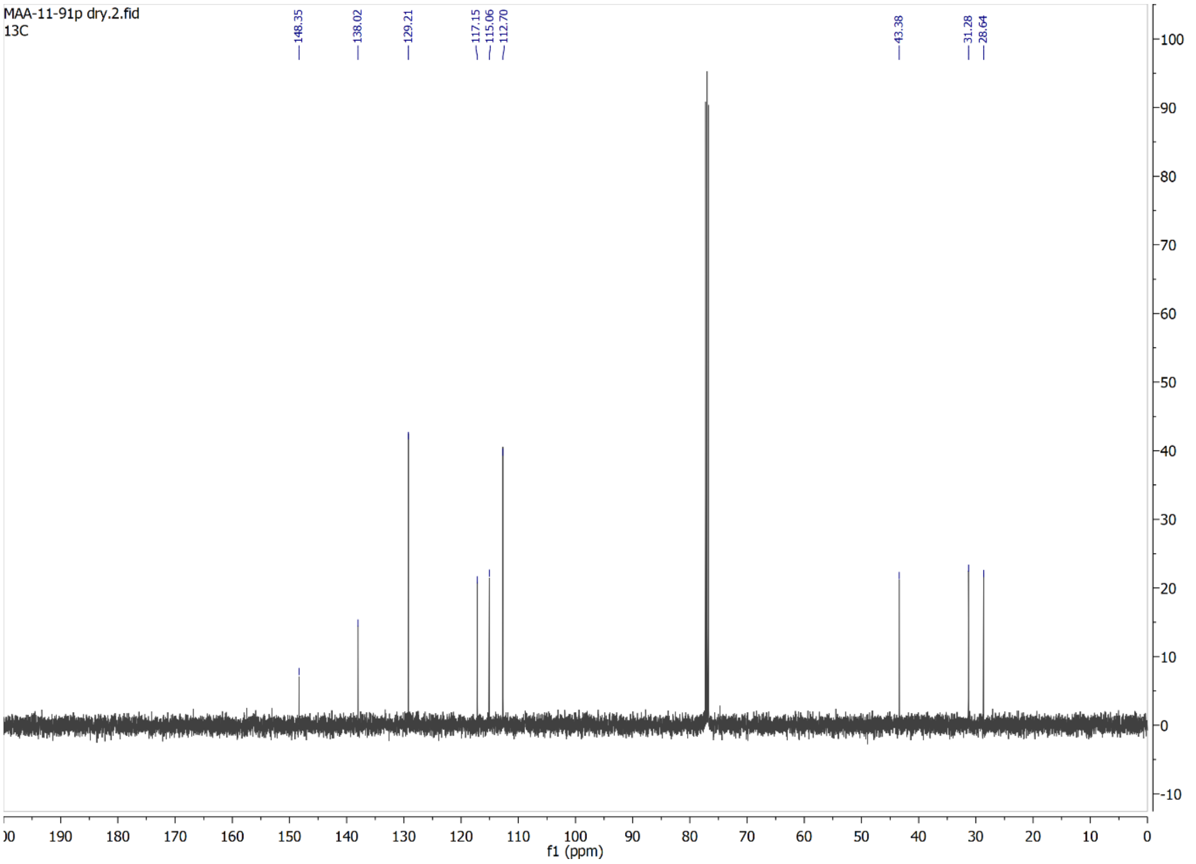
MAA-11-91p dry.2.fid
13C
148.35
138.02
129.21
117.15
115.06
112.70
43.38
31.28
28.64
f1 (ppm)

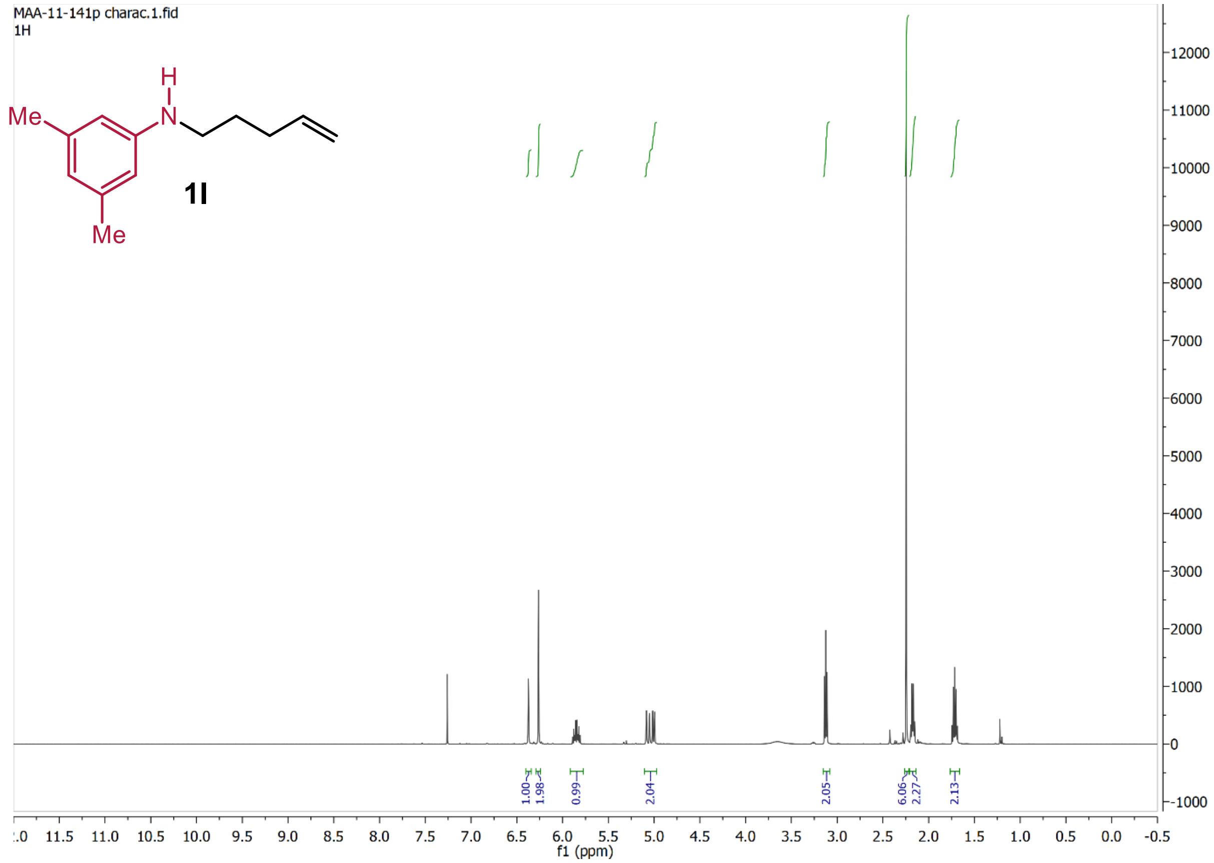
MAA-11-141p charac.1.fid
1H
Me
H
N
Me
1l
1.00
1.98
0.99
2.04
2.05
6.06
2.27
2.13
f1 (ppm)

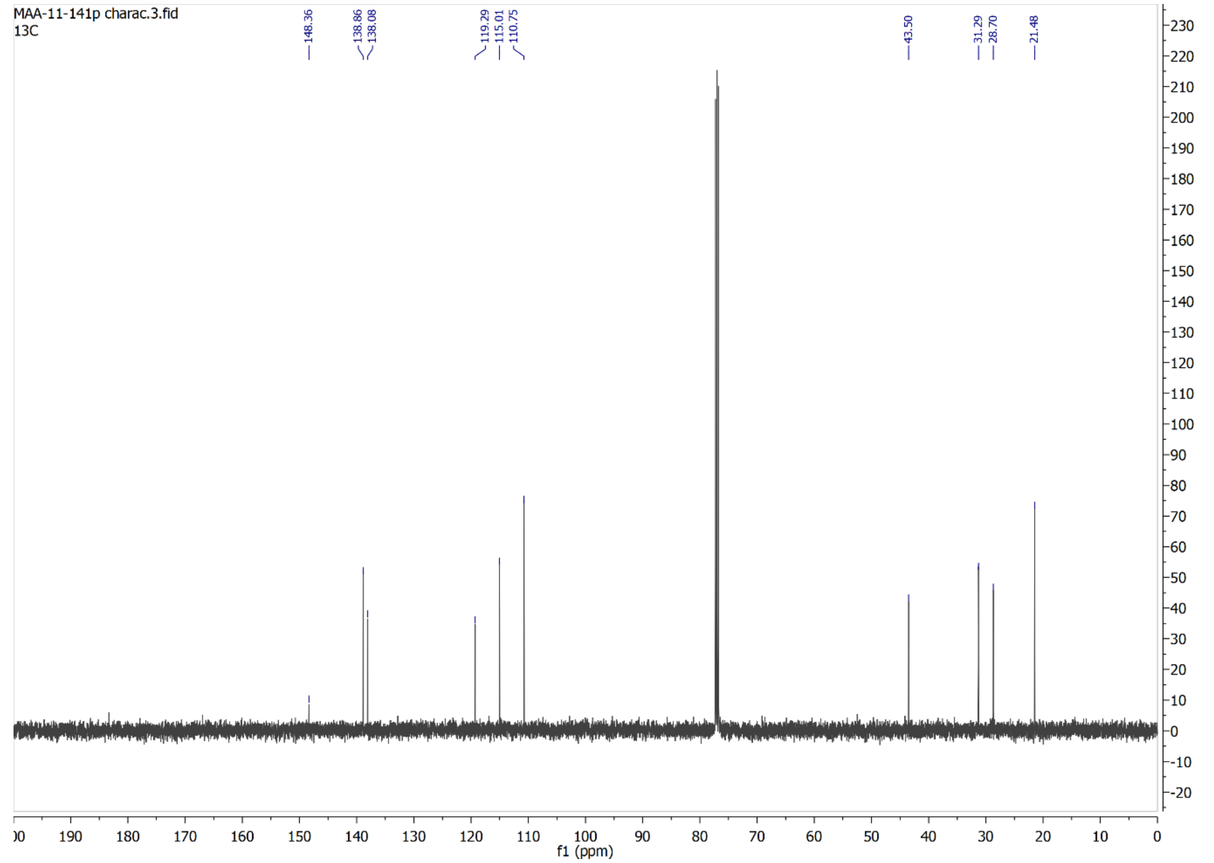
MAA-11-141p charac.3.fid
13C
148.36
138.86
138.08
119.29
115.01
110.75
43.50
31.29
28.70
21.48
f1 (ppm)

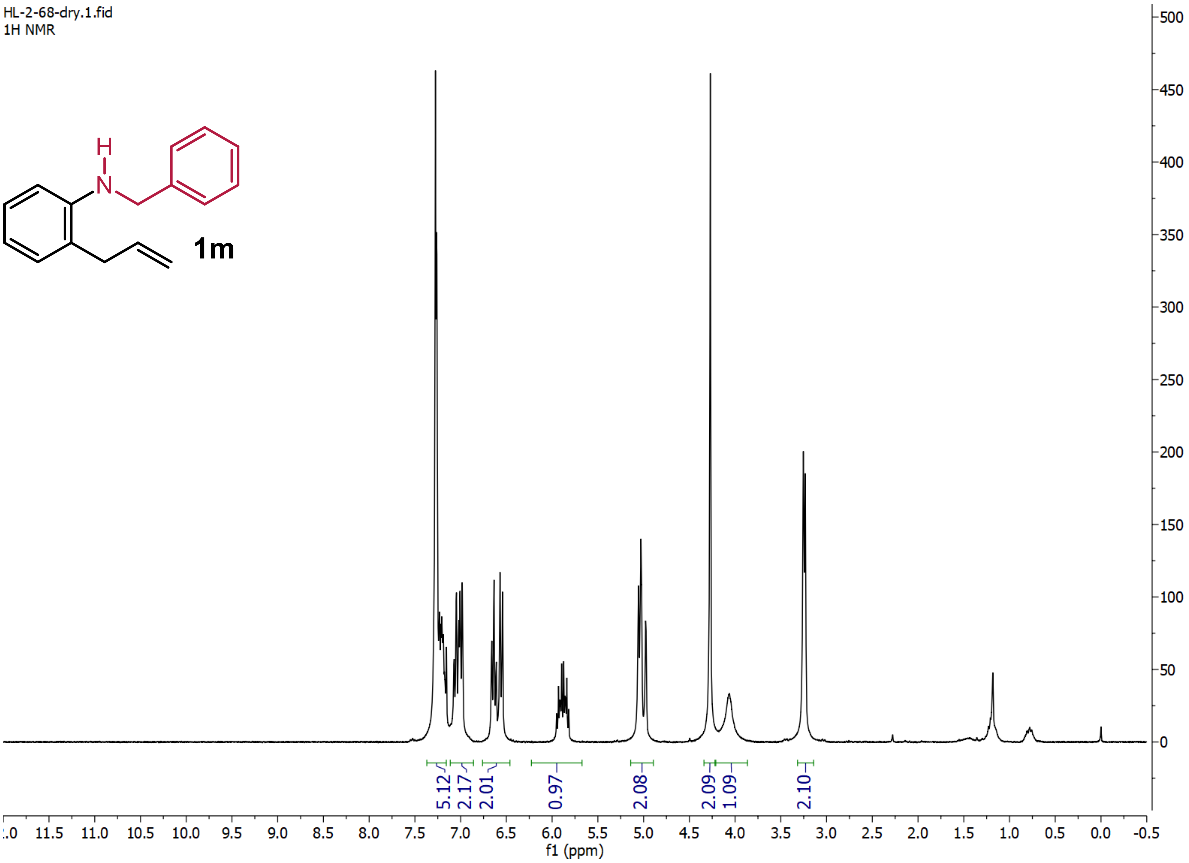
HL-2-68-dry.1.fid
1H NMR
1m
5.12
2.17
2.01
0.97
2.08
2.09
1.09
2.10
f1 (ppm)

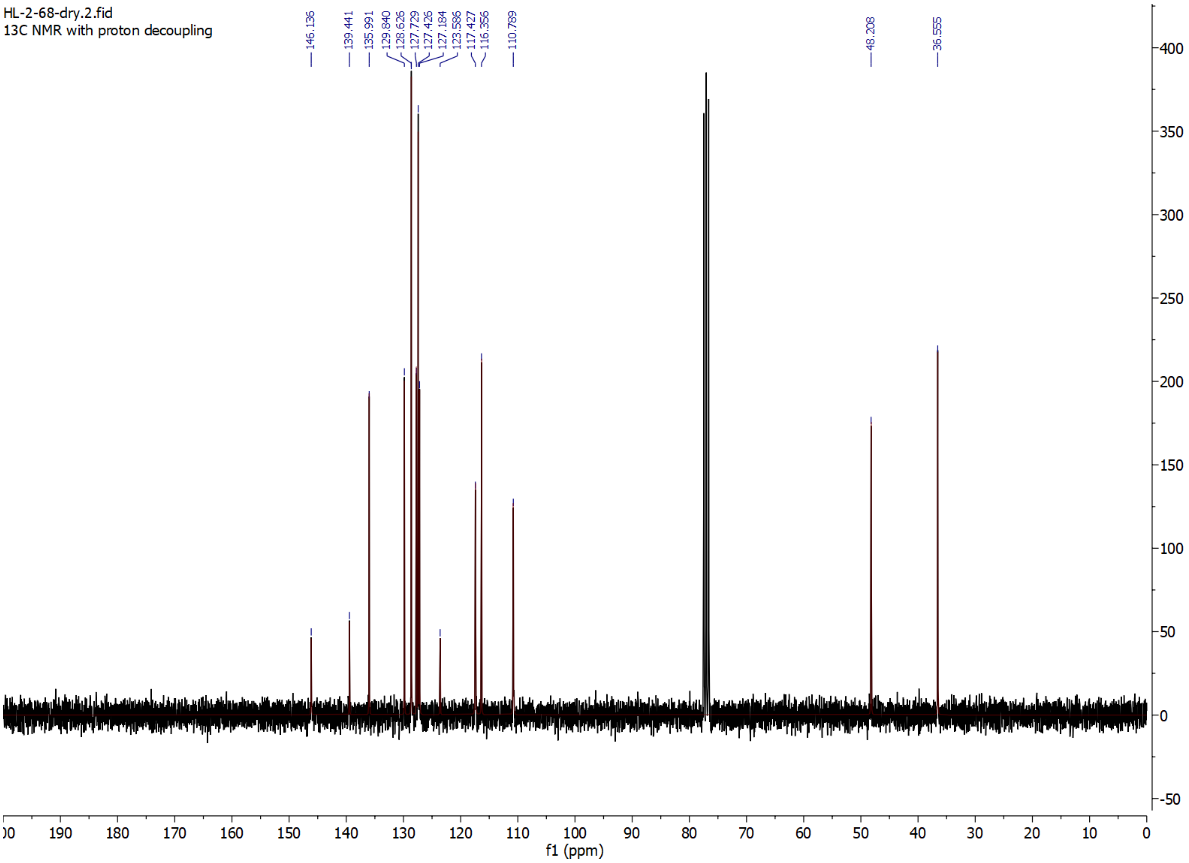
HL-2-68-dry.2.fid
13C NMR with proton decoupling
146.136
139.441
135.991
129.840
128.626
127.729
127.426
127.184
123.586
117.427
116.356
110.789
48.208
36.555
f1 (ppm)

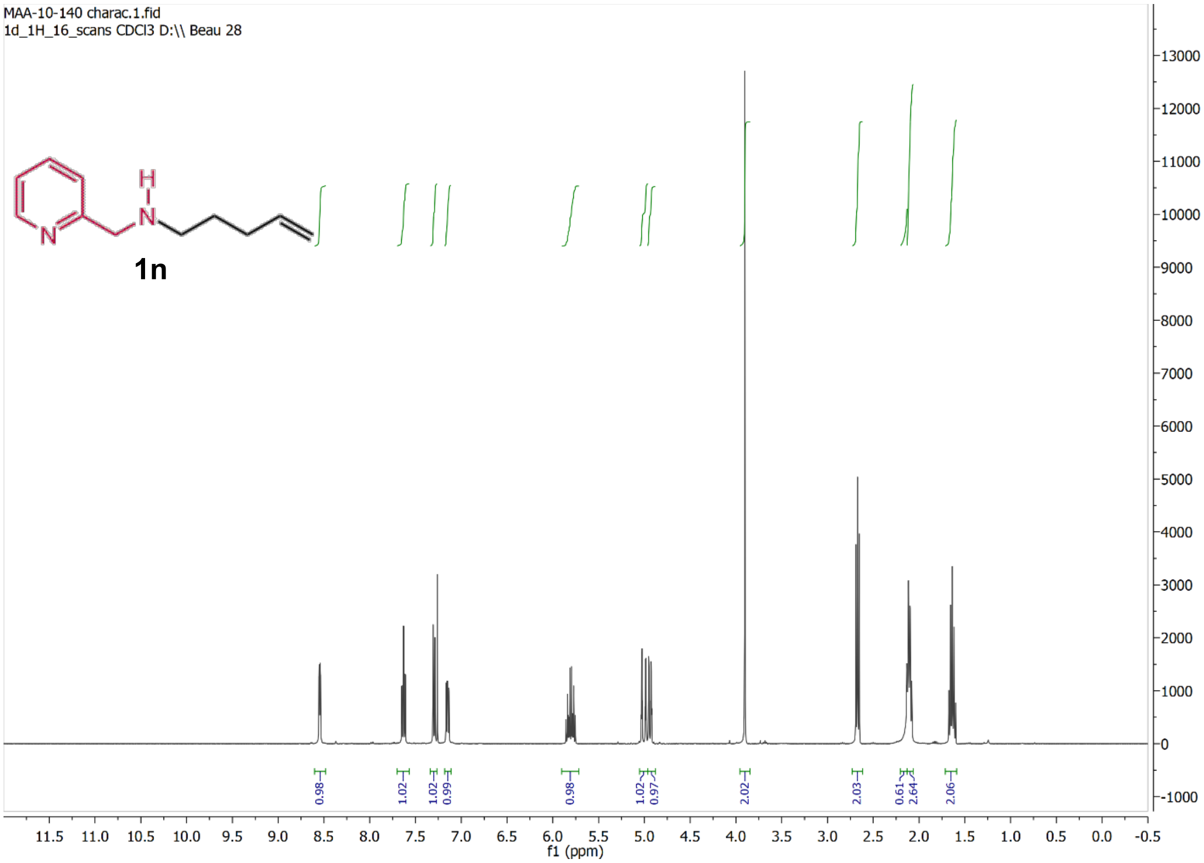
MAA-10-140 charac.1.fid
1d_1H_16_scans CDCl3 D:\\ Beau 28
1n
0.98
1.02
1.02
0.99
0.98
1.02
0.97
2.02
2.03
0.61
2.64
2.06
f1 (ppm)

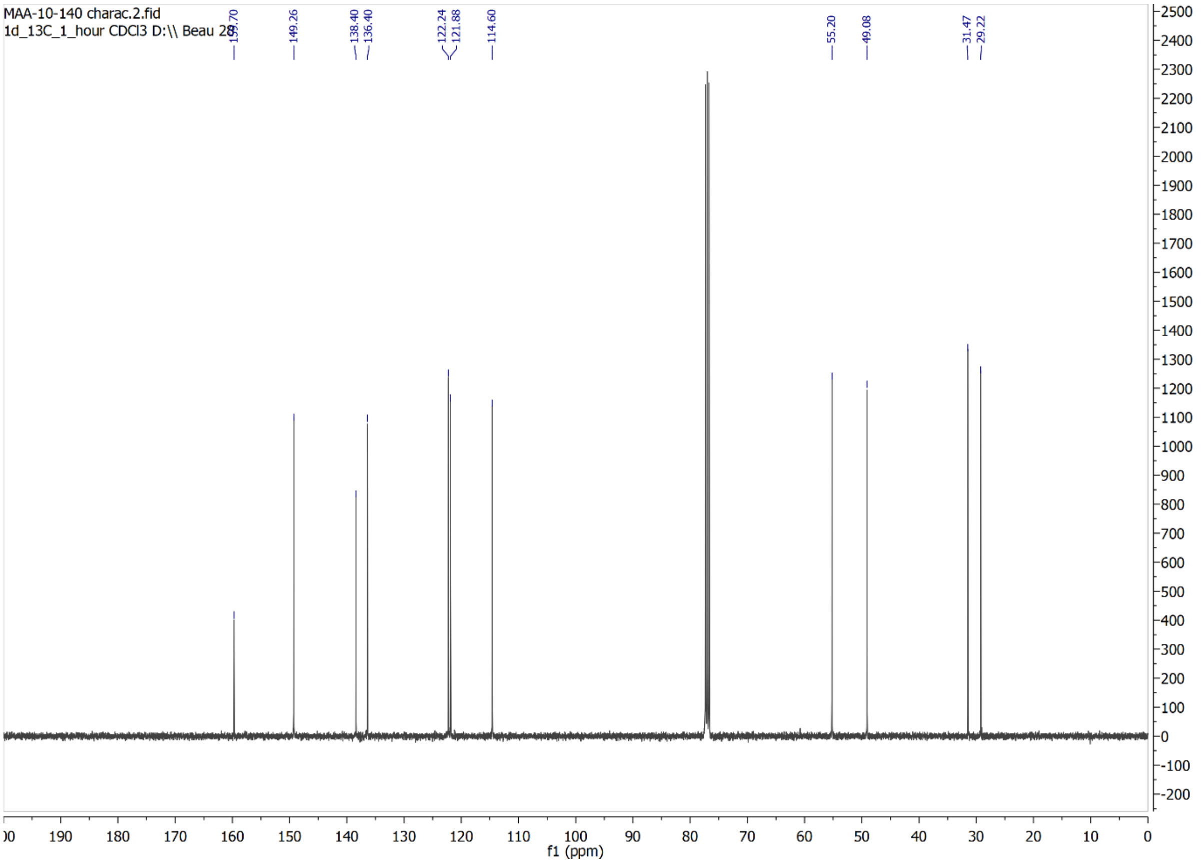
MAA-10-140 charac.2.fid
1d_13C_1_hour CDCl3 D:\\ Beau 28
149.26
138.40
136.40
122.24
121.88
114.60
55.20
49.08
31.47
29.22
f1 (ppm)

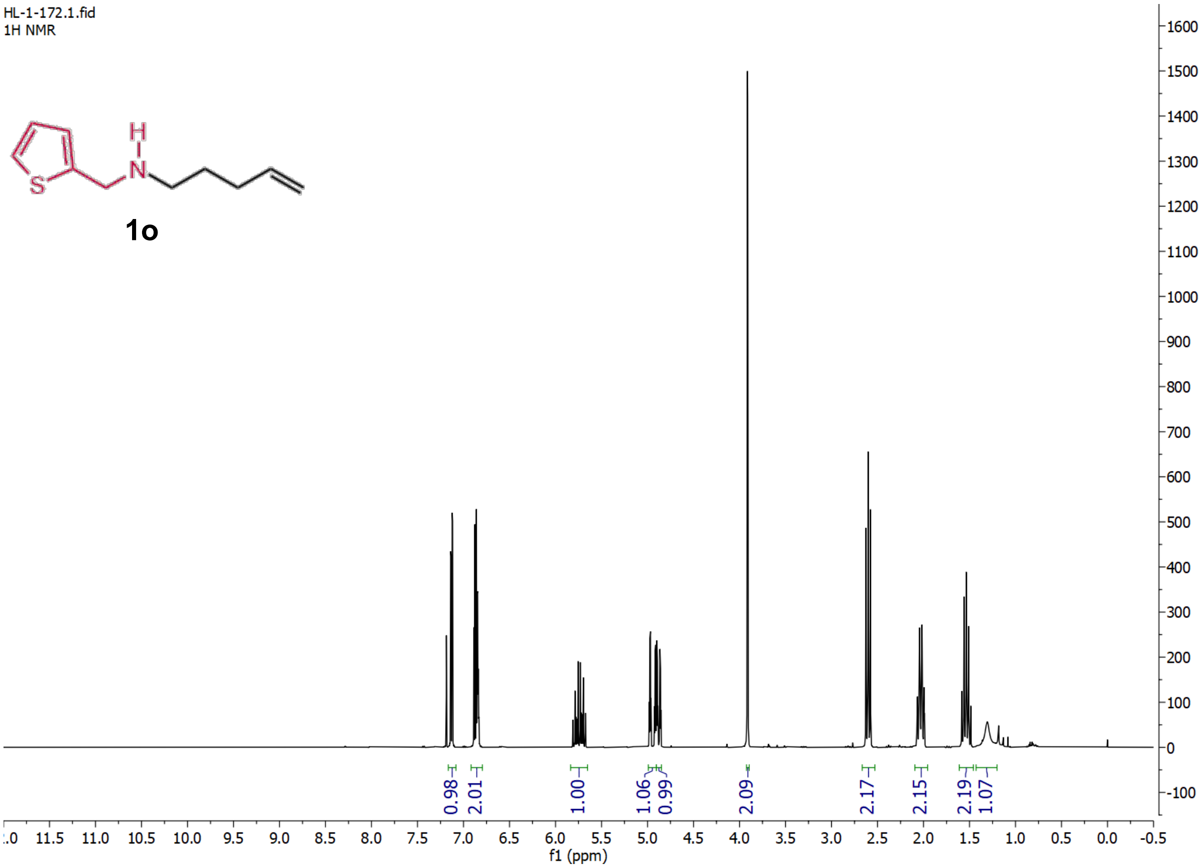
HL-1-172.1.fid
1H NMR
0.98
2.01
1.00
1.06
0.99
2.09
2.17
2.15
2.19
1.07
f1 (ppm)

1o

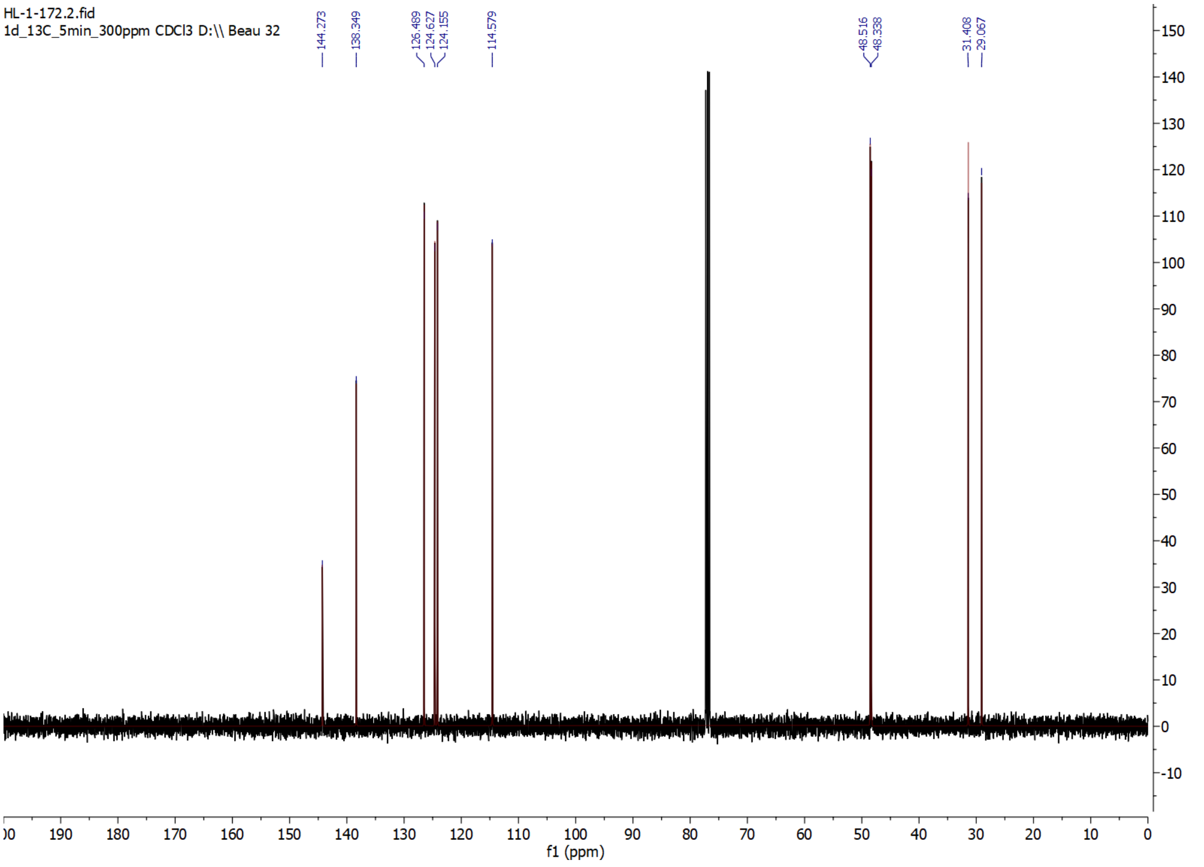
HL-1-172.2.fid
1d_13C_5min_300ppm CDCl3 D:\\ Beau 32
144.273
138.349
126.489
124.627
124.155
114.579
48.516
48.338
31.408
29.067
f1 (ppm)

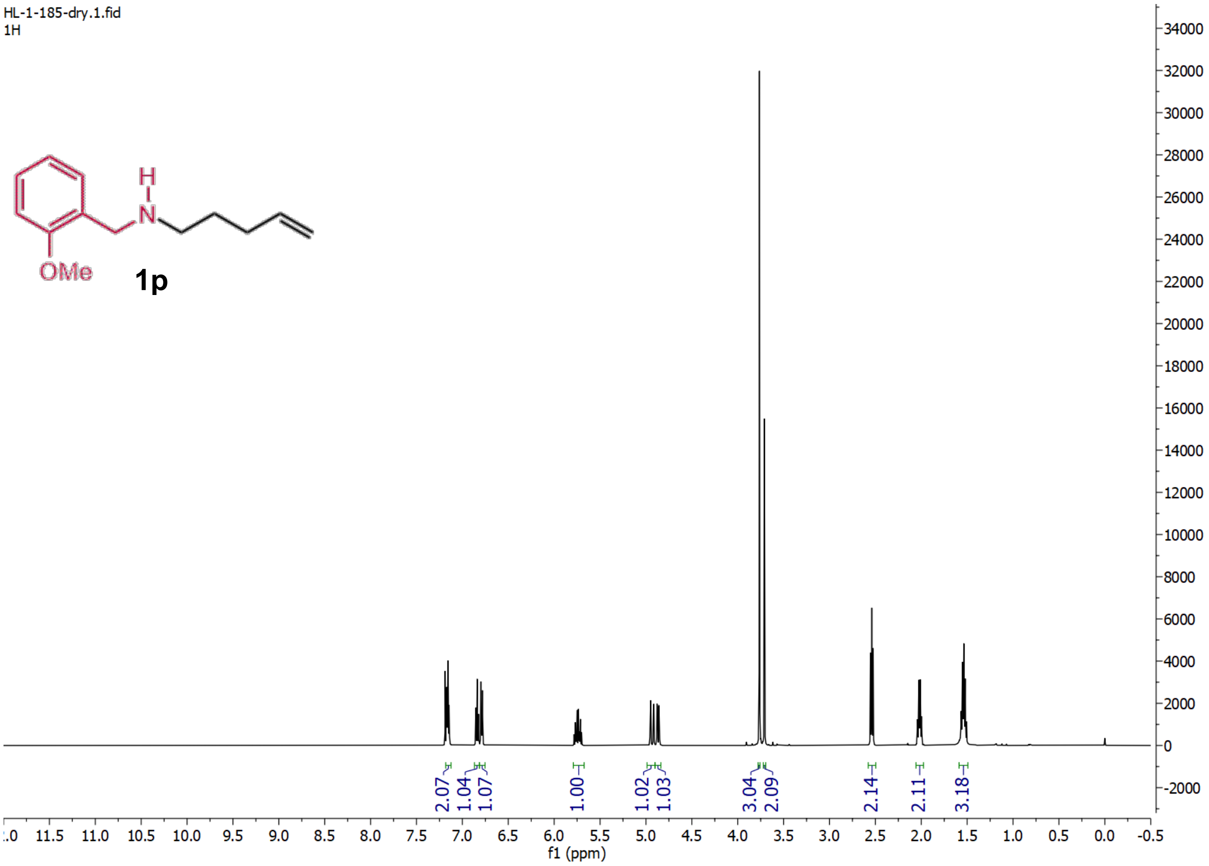
HL-1-185-dry.1.fid
1H
2.07
1.04
1.07
1.00
1.02
1.03
3.04
2.09
2.14
2.11
3.18
f1 (ppm)
H
N
OMe

1p

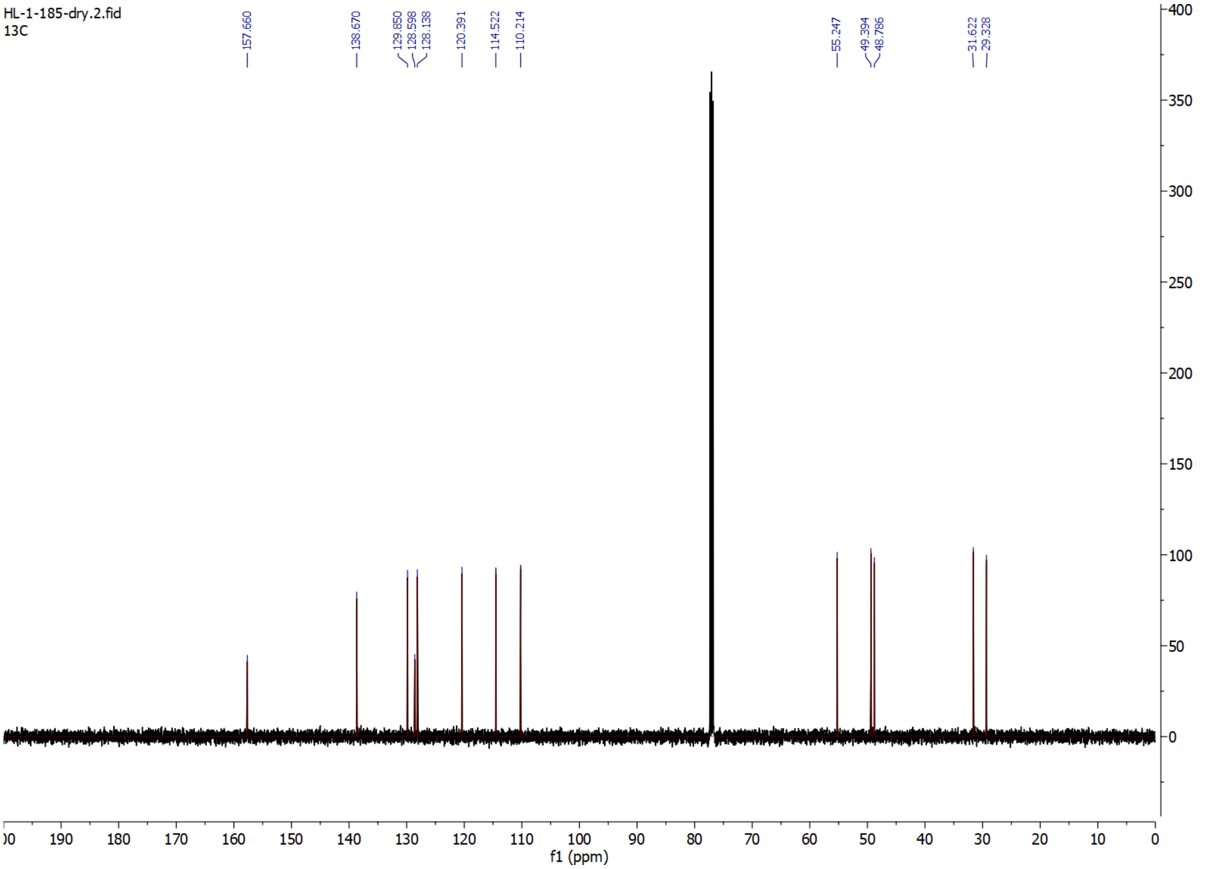
HL-1-185-dry.2.fid
13C
157.660
138.670
129.850
128.598
128.138
120.391
114.522
110.214
55.247
49.394
48.786
31.622
29.328
f1 (ppm)

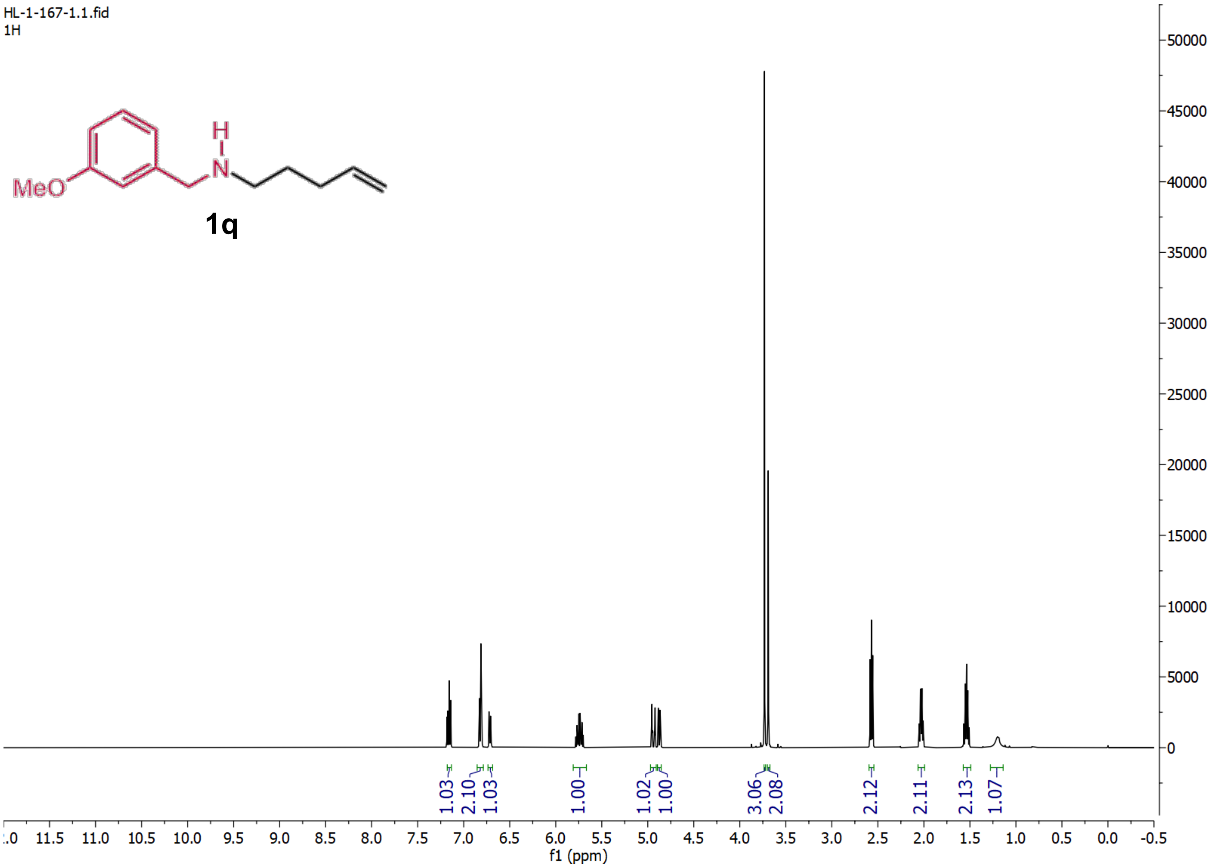
HL-1-167-1.1.fid
1H
MeO
H
N
1q
1.03
2.10
1.03
1.00
1.02
1.00
3.06
2.08
2.12
2.11
2.13
1.07
f1 (ppm)

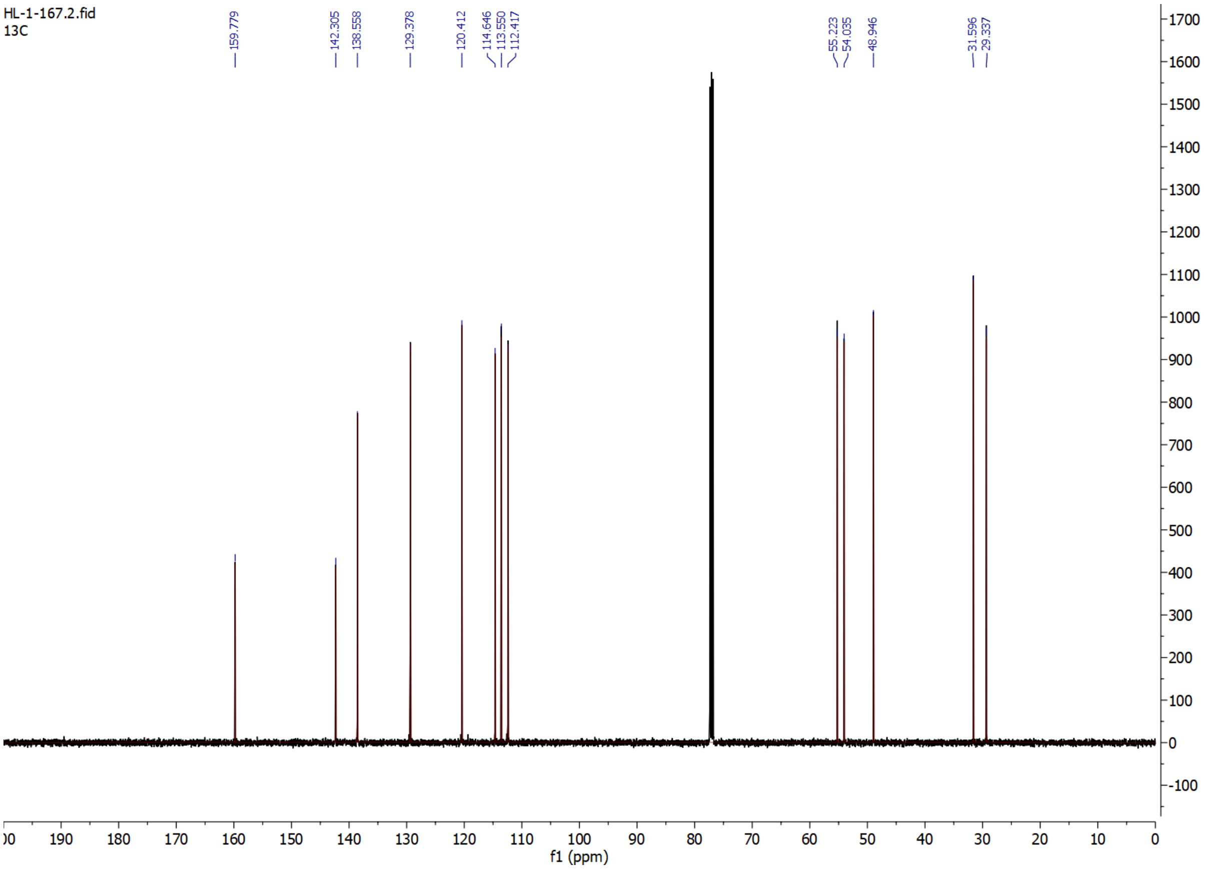
HL-1-167.2.fid
13C
159.779
142.305
138.558
129.378
120.412
114.646
113.550
112.417
55.223
54.035
48.946
31.596
29.337
f1 (ppm)

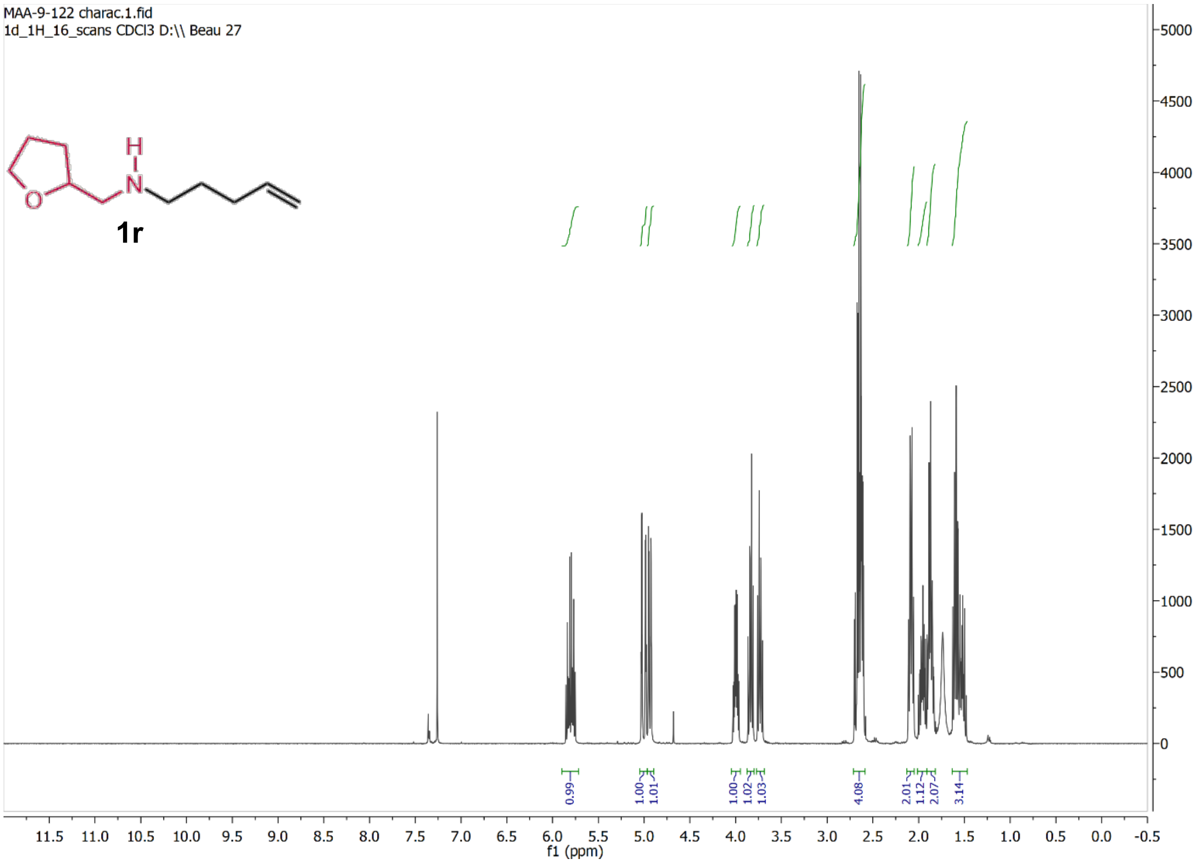
MAA-9-122 charac.1.fid
1d_1H_16_scans CDCl3 D:\\ Beau 27
1r
0.99
1.00
1.01
1.00
1.02
1.03
4.08
2.01
1.12
2.07
3.14
f1 (ppm)

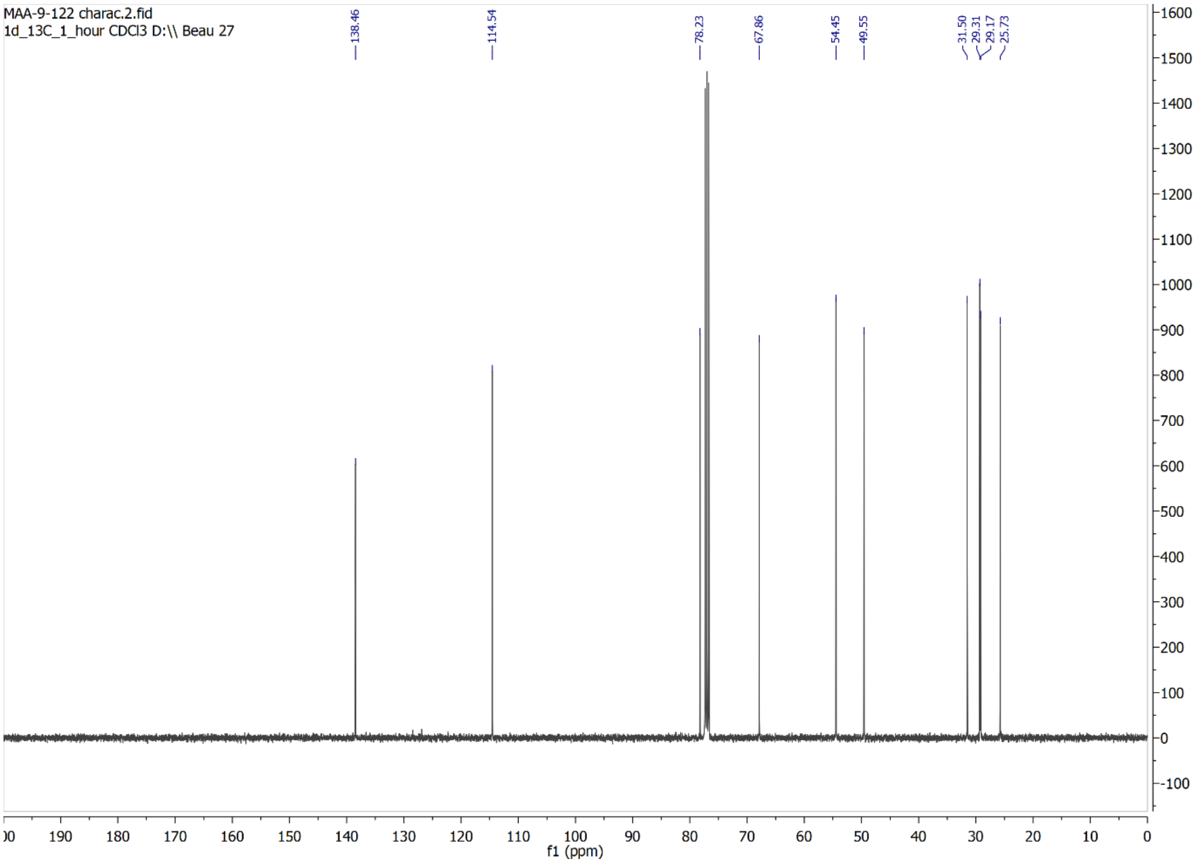
MAA-9-122 charac.2.fid
1d_13C_1_hour CDCl3 D:\\ Beau 27
138.46
114.54
78.23
67.86
54.45
49.55
31.50
29.31
29.17
25.73
f1 (ppm)

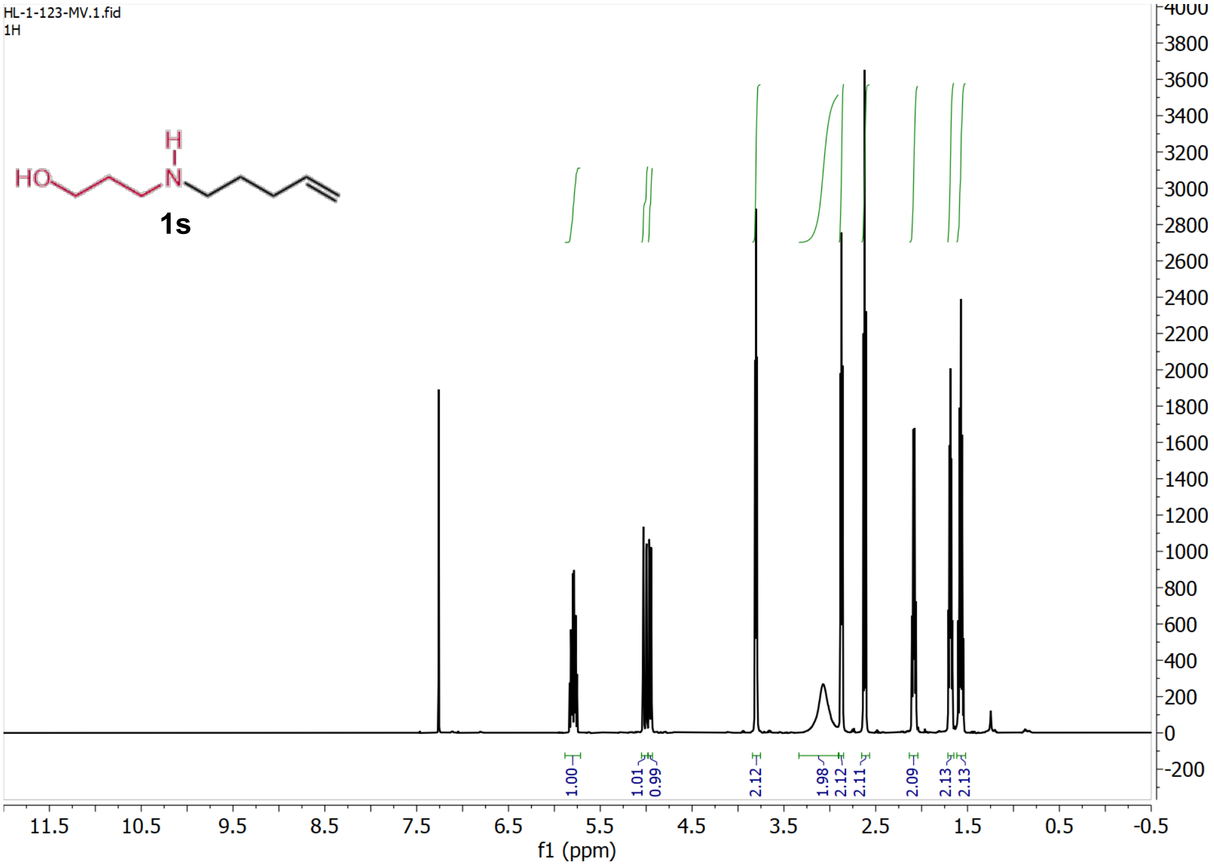
HL-1-123-MV.1.fid
1H
HO
H
N
1s
1.00
1.01
0.99
2.12
1.98
2.12
2.11
2.09
2.13
2.13
f1 (ppm)

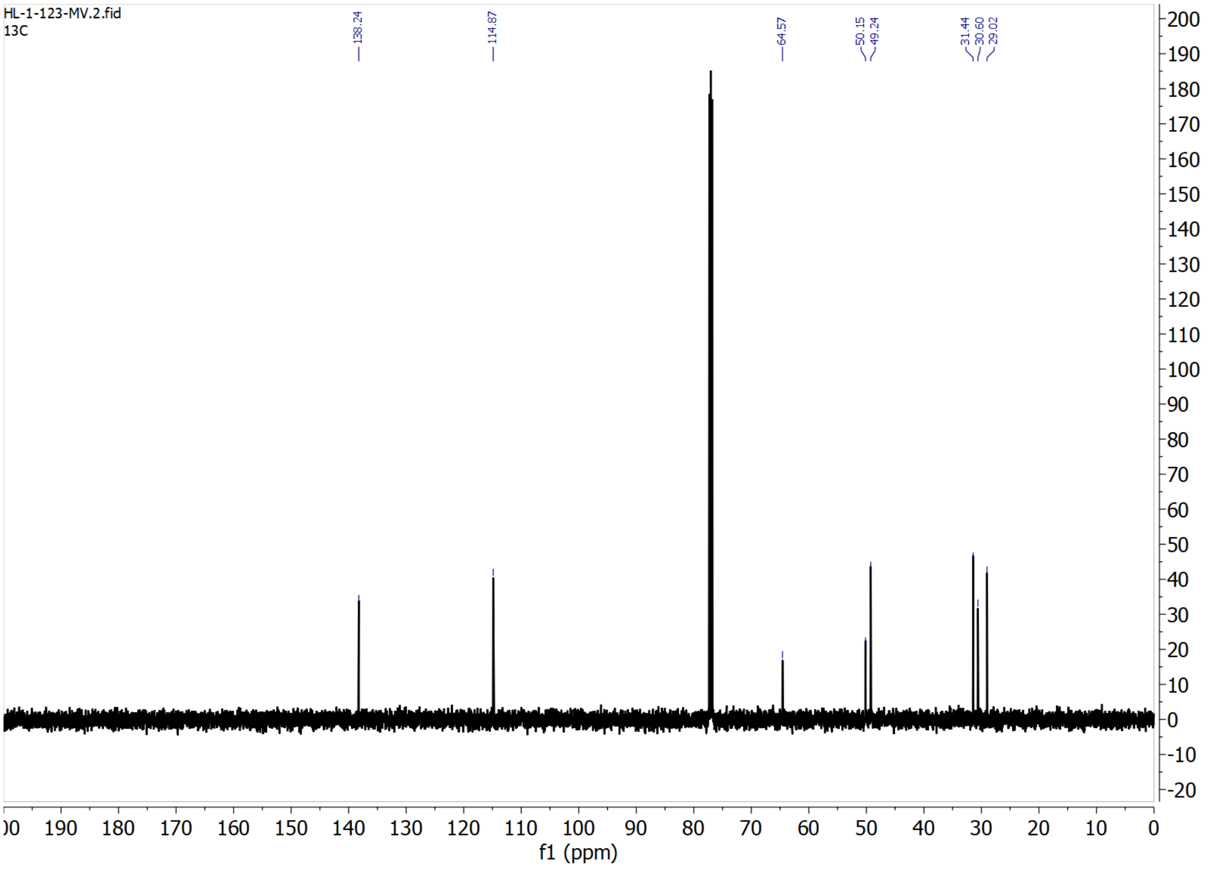
HL-1-123-MV.2.fid
13C
138.24
114.87
64.57
50.15
49.24
31.44
30.60
29.02
f1 (ppm)

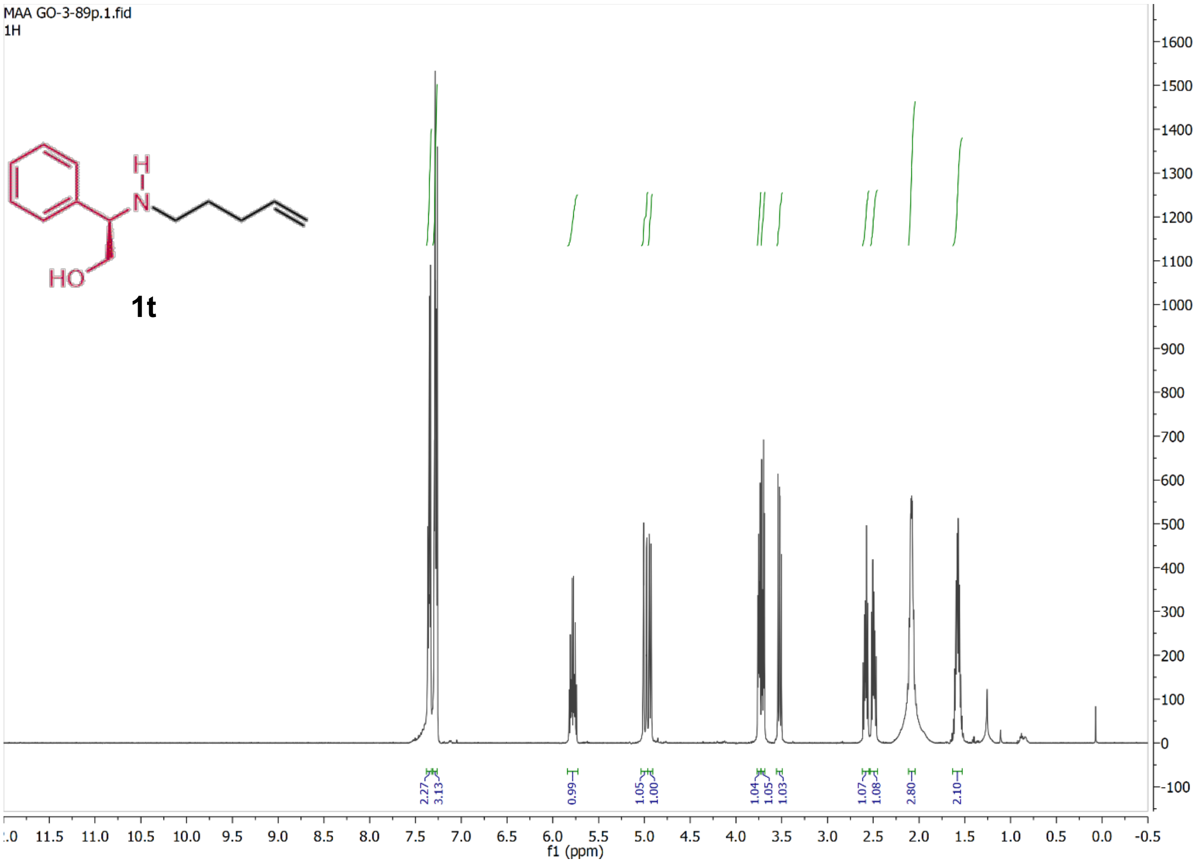
MAA GO-3-89p.1.fid
1H
HO
H
N
1t
2.27
3.13
0.99
1.05
1.00
1.04
1.05
1.03
1.07
1.08
2.80
2.10
f1 (ppm)

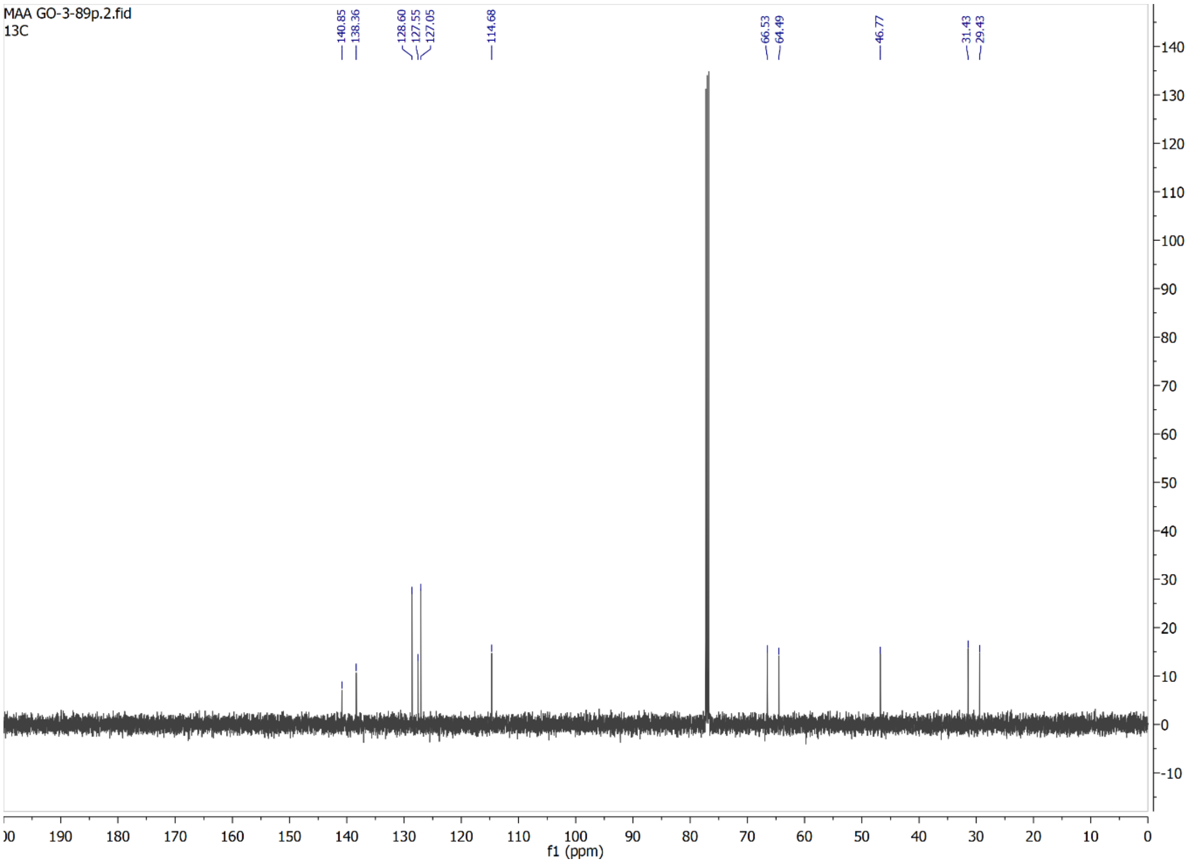
MAA GO-3-89p.2.fid
13C
140.85
138.36
128.60
127.55
127.05
114.68
66.53
64.49
46.77
31.43
29.43
f1 (ppm)

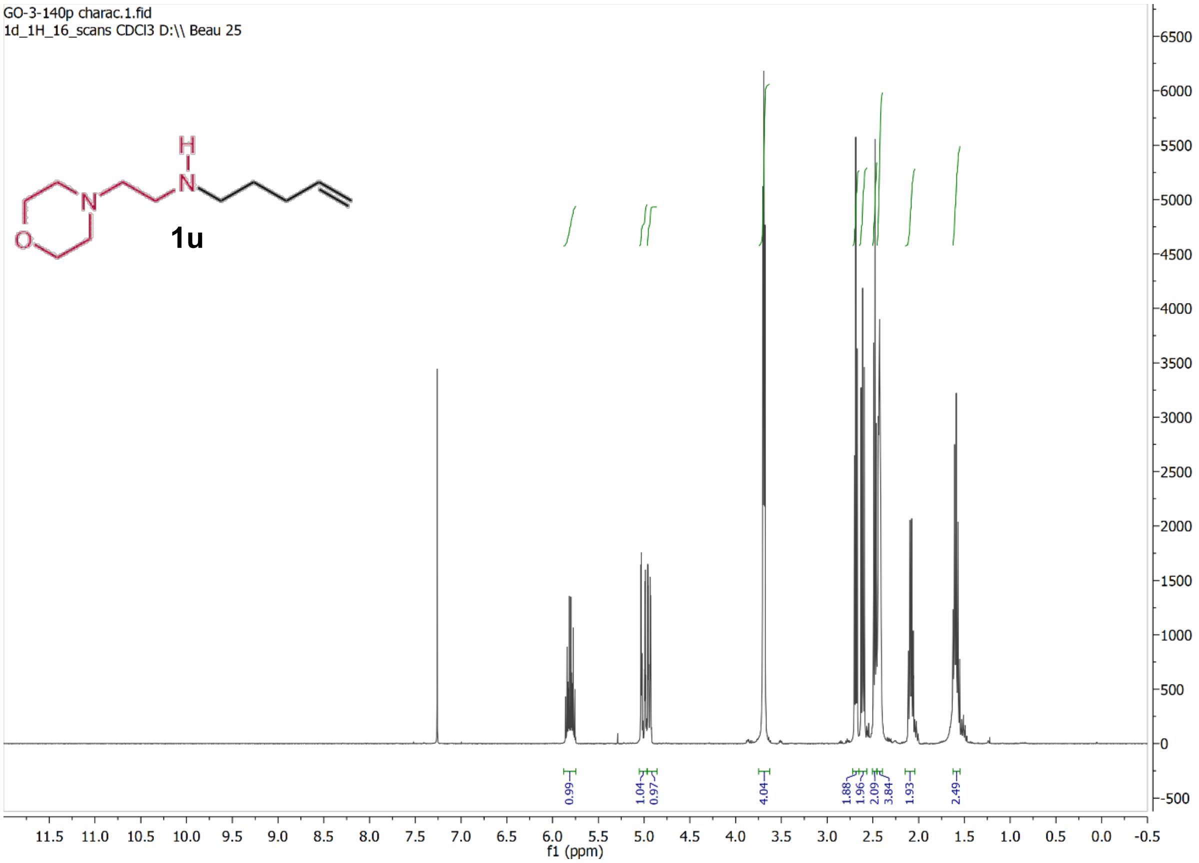
GO-3-140p charac.1.fid
1d_1H_16_scans CDCl3 D:\\ Beau 25
1u
0.99
1.04
0.97
4.04
1.88
1.96
2.09
3.84
1.93
2.49
f1 (ppm)

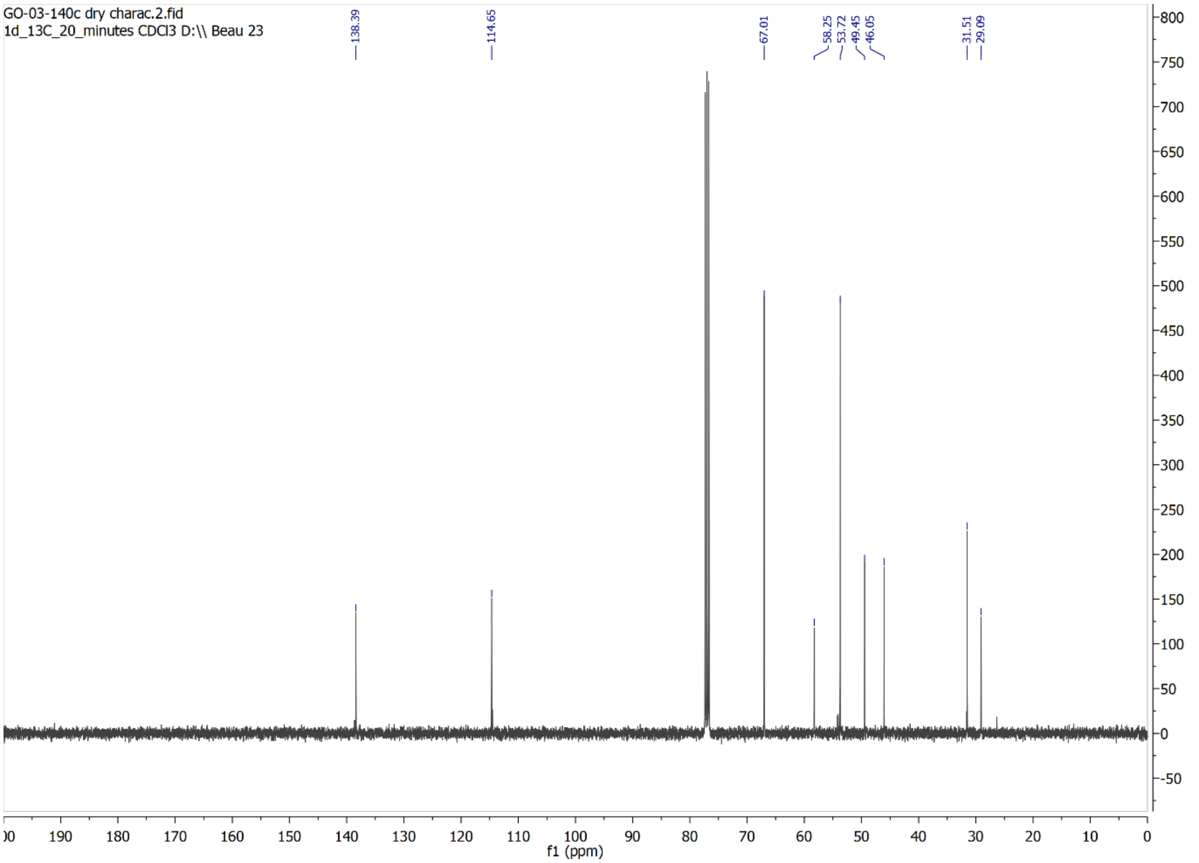
GO-03-140c dry charac.2.fid
1d_13C_20_minutes CDCl3 D:\\ Beau 23
138.39
114.65
67.01
58.25
53.72
49.45
46.05
31.51
29.09
f1 (ppm)

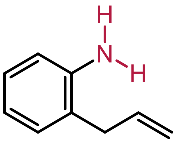
H
N
H

1v

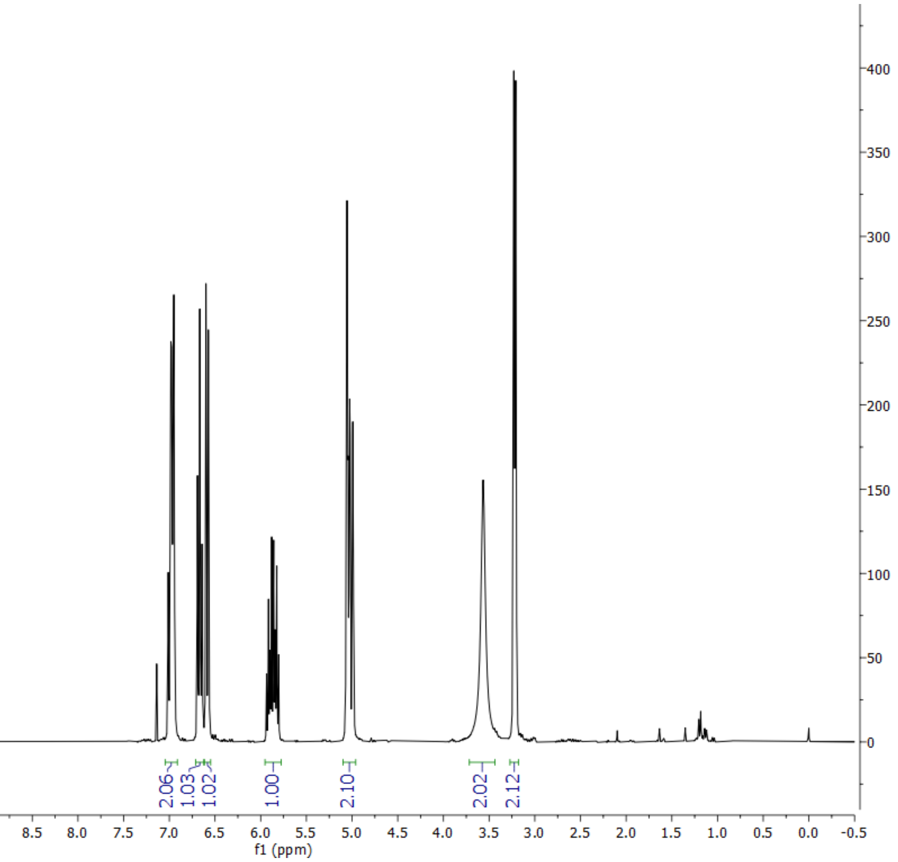
HL-2-59-dry.1.fid
1H NMR
2.06
1.03
1.02
1.00
2.10
2.02
2.12
f1 (ppm)

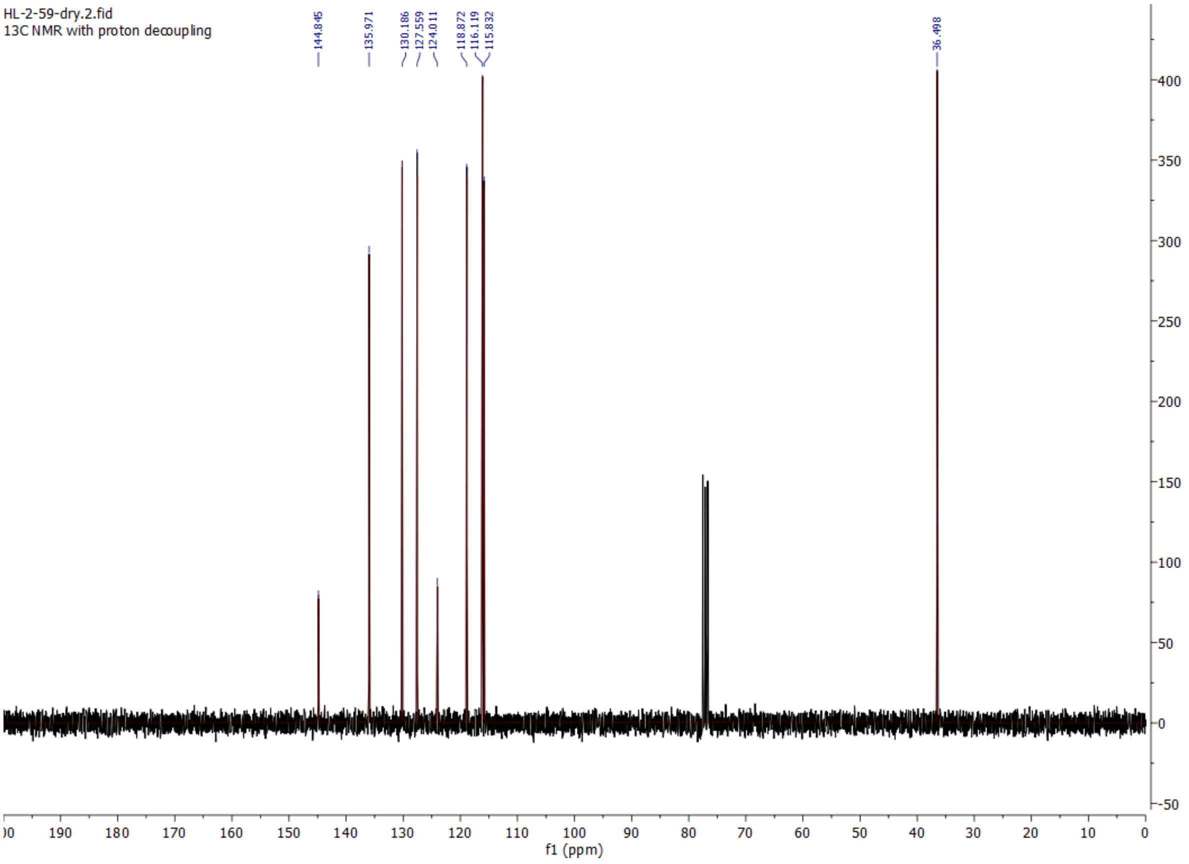
HL-2-59-dry.2.fid
13C NMR with proton decoupling
144.845
135.971
130.186
127.559
124.011
118.872
116.119
115.832
36.498
f1 (ppm)

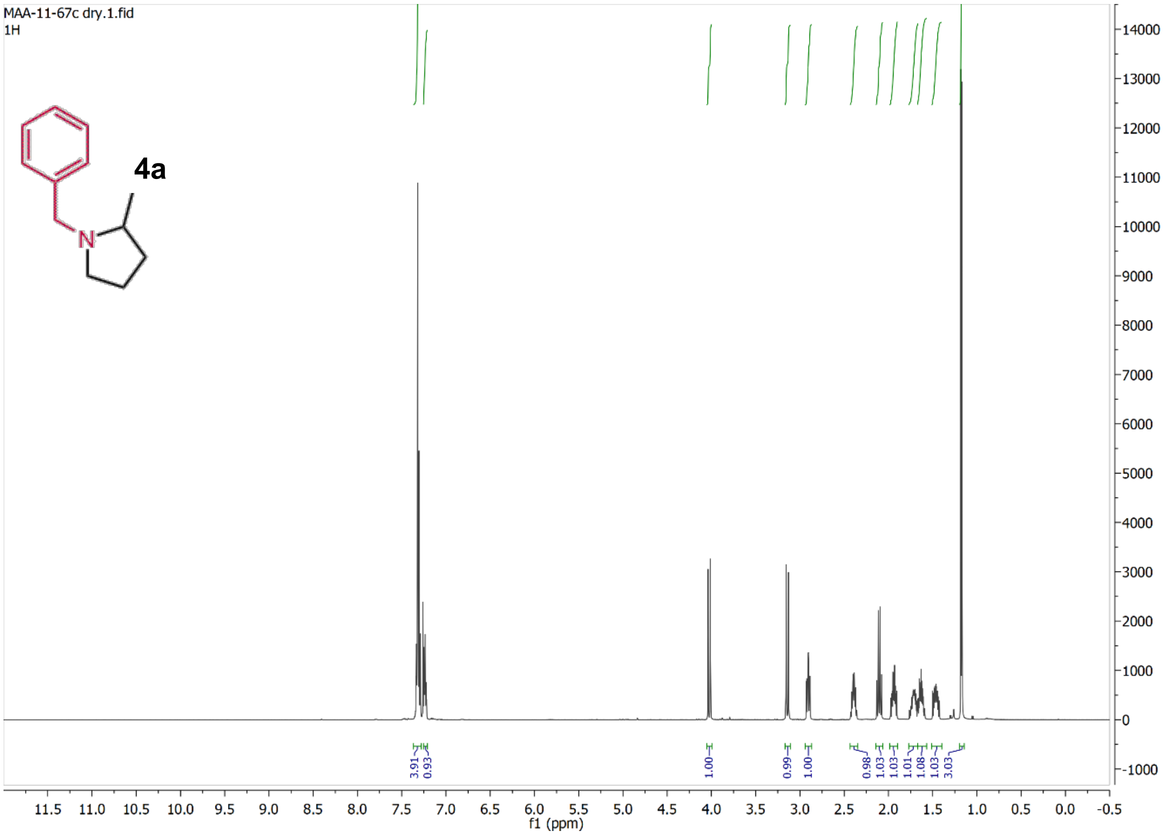
MAA-11-67c dry.1.fid
1H
4a
N
3.91
0.93
1.00
0.99
1.00
0.98
1.03
1.03
1.01
1.08
1.03
3.03
f1 (ppm)

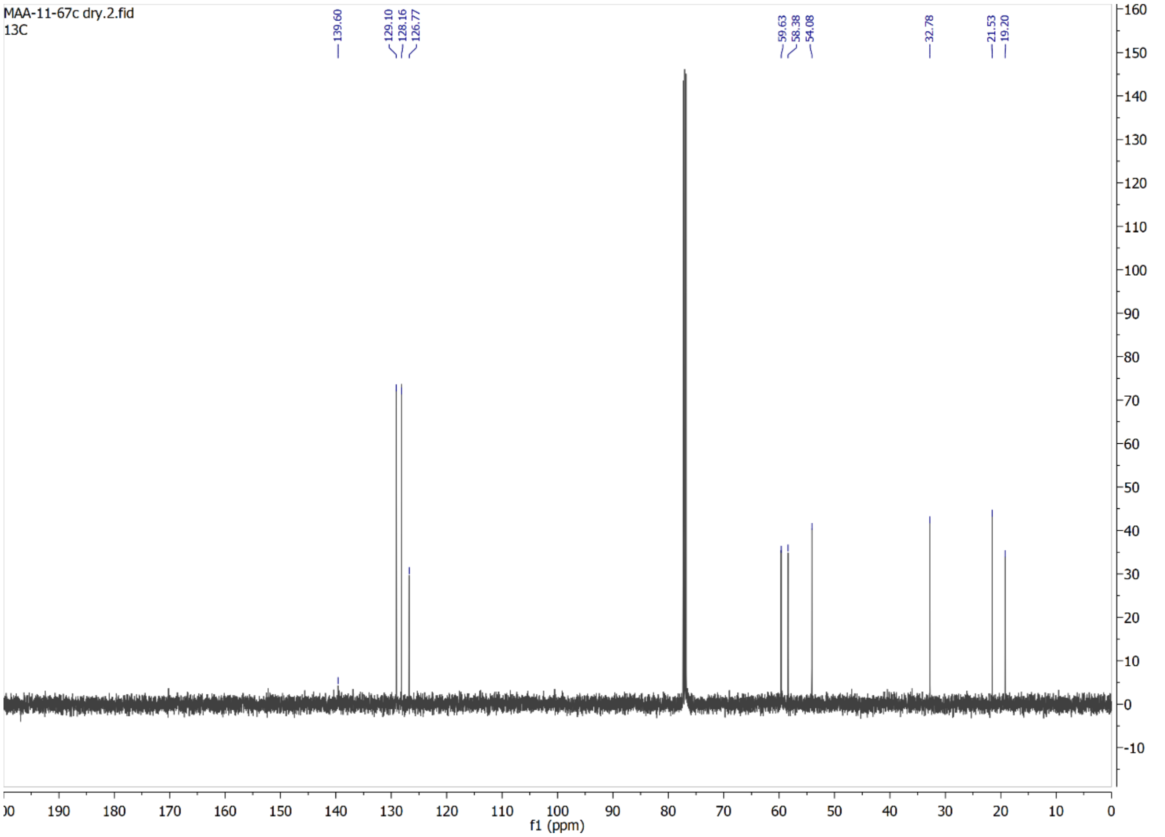
MAA-11-67c dry.2.fid
13C
139.60
129.10
128.16
126.77
59.63
58.38
54.08
32.78
21.53
19.20
f1 (ppm)

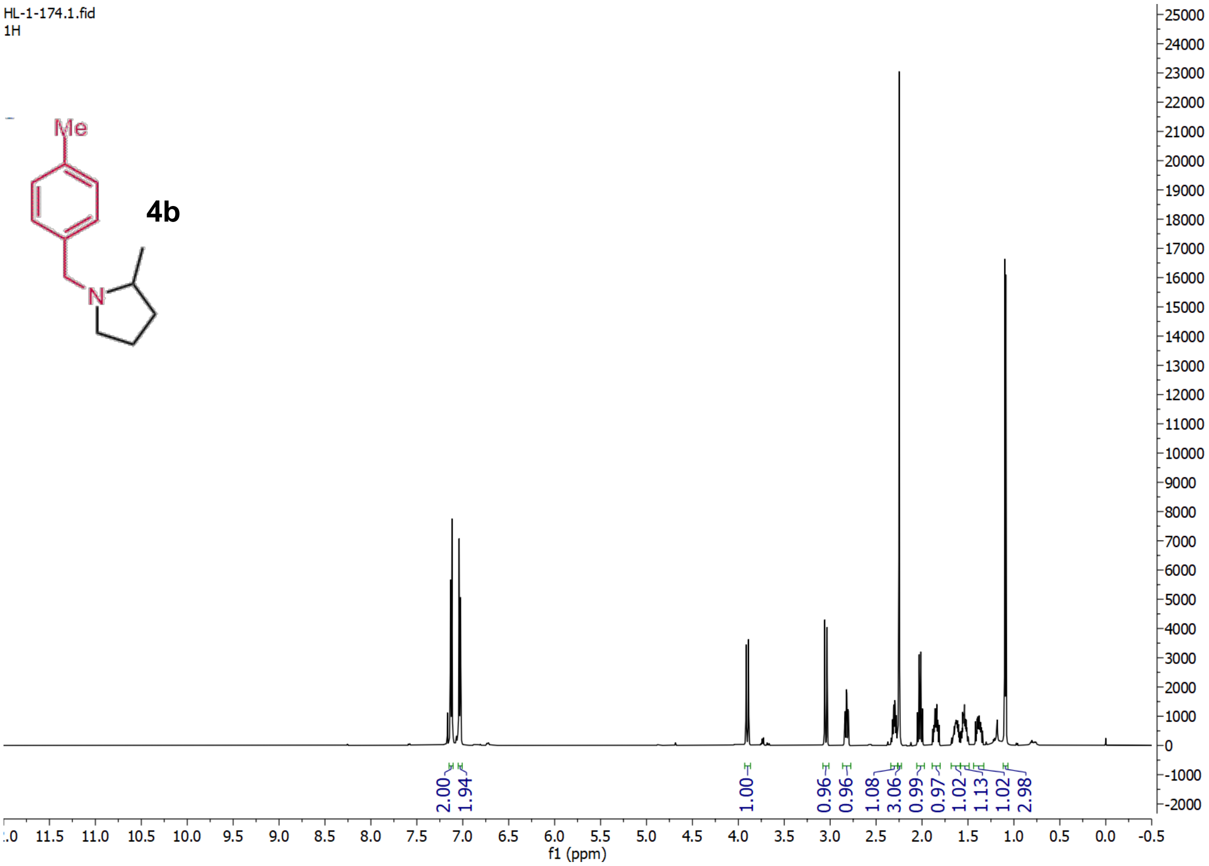
HL-1-174.1.fid
1H
Me
N
4b
2.00
1.94
1.00
0.96
0.96
1.08
3.06
0.99
0.97
1.02
1.13
1.02
2.98
f1 (ppm)

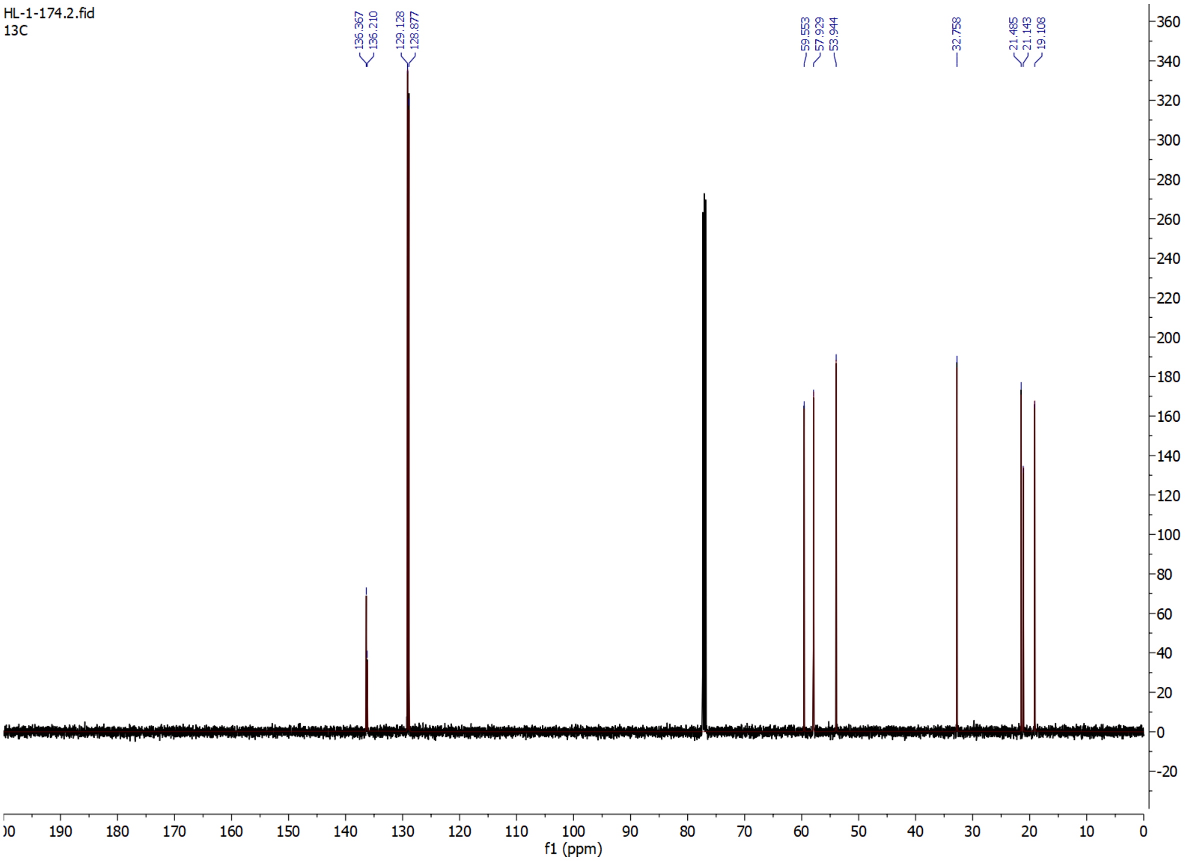
HL-1-174.2.fid
13C
136.367
136.210
129.128
128.877
59.553
57.929
53.944
32.758
21.485
21.143
19.108
f1 (ppm)

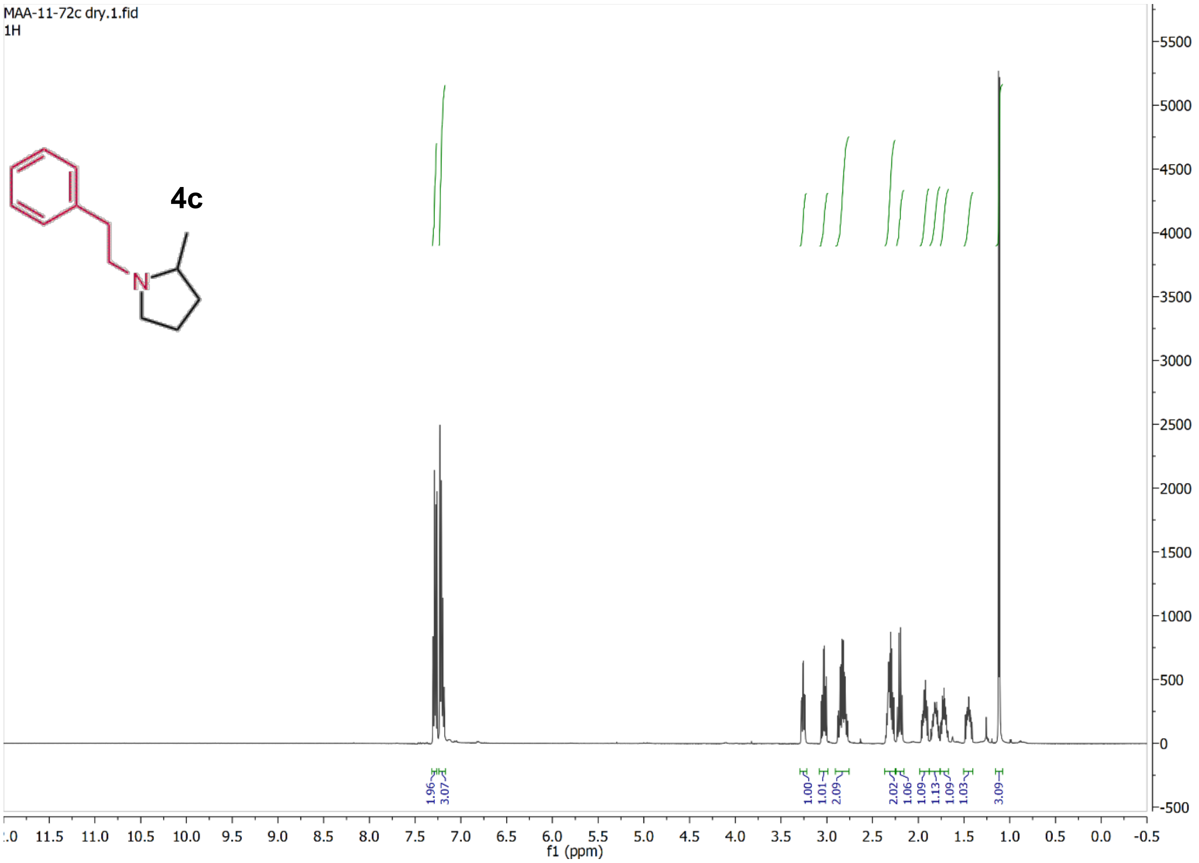
MAA-11-72c dry.1.fid
1H
4c
N
1.96
3.07
1.00
1.01
2.09
2.02
1.06
1.09
1.13
1.09
1.03
3.09
f1 (ppm)

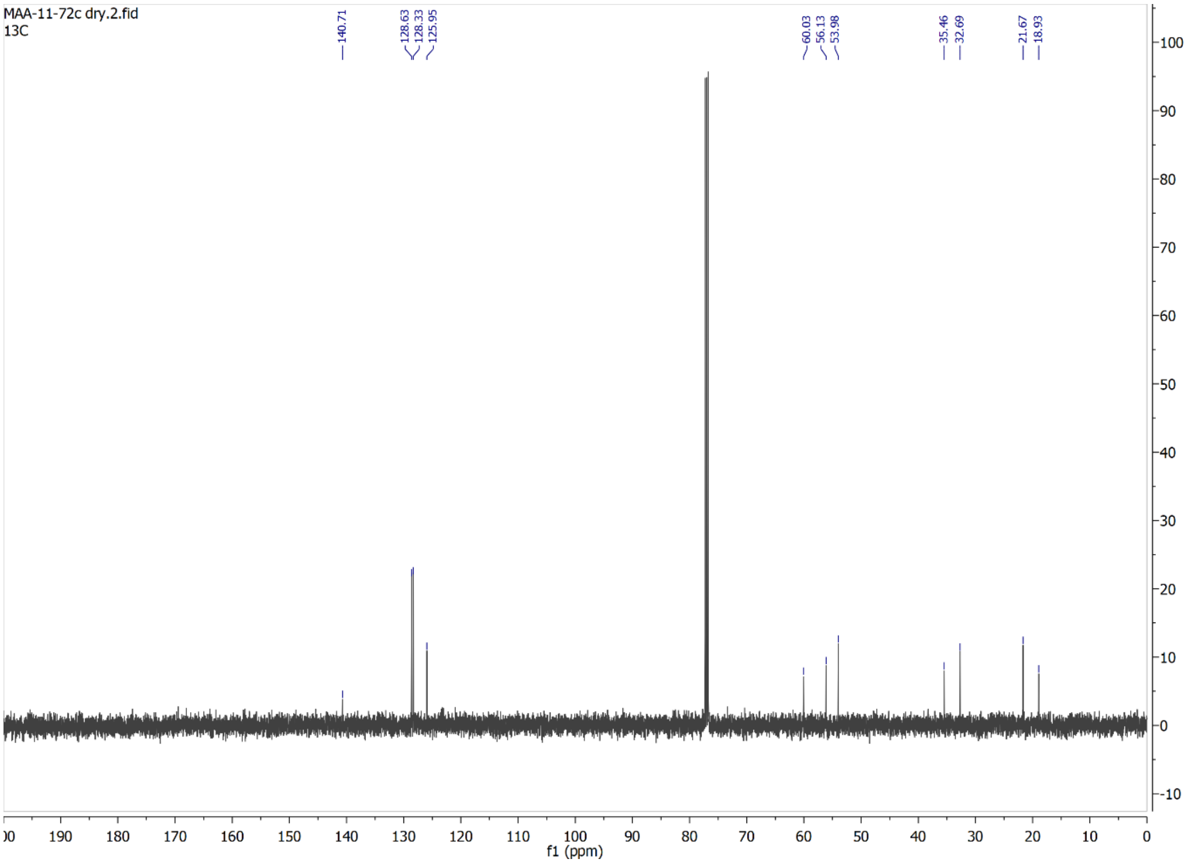
MAA-11-72c dry.2.fid
13C
140.71
128.63
128.33
125.95
60.03
56.13
53.98
35.46
32.69
21.67
18.93
f1 (ppm)

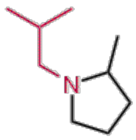

4d

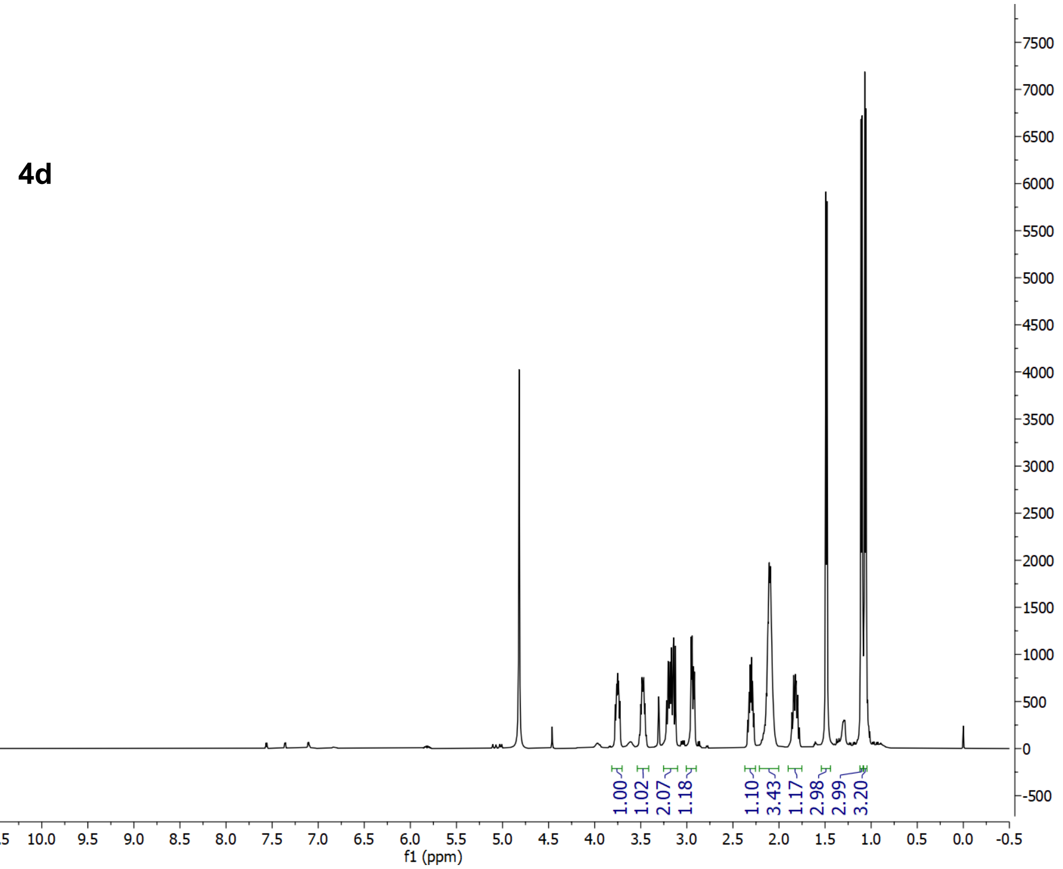
HL-1-181-2.1.fid
1H
1.00
1.02
2.07
1.18
1.10
3.43
1.17
2.98
2.99
3.20
f1 (ppm)

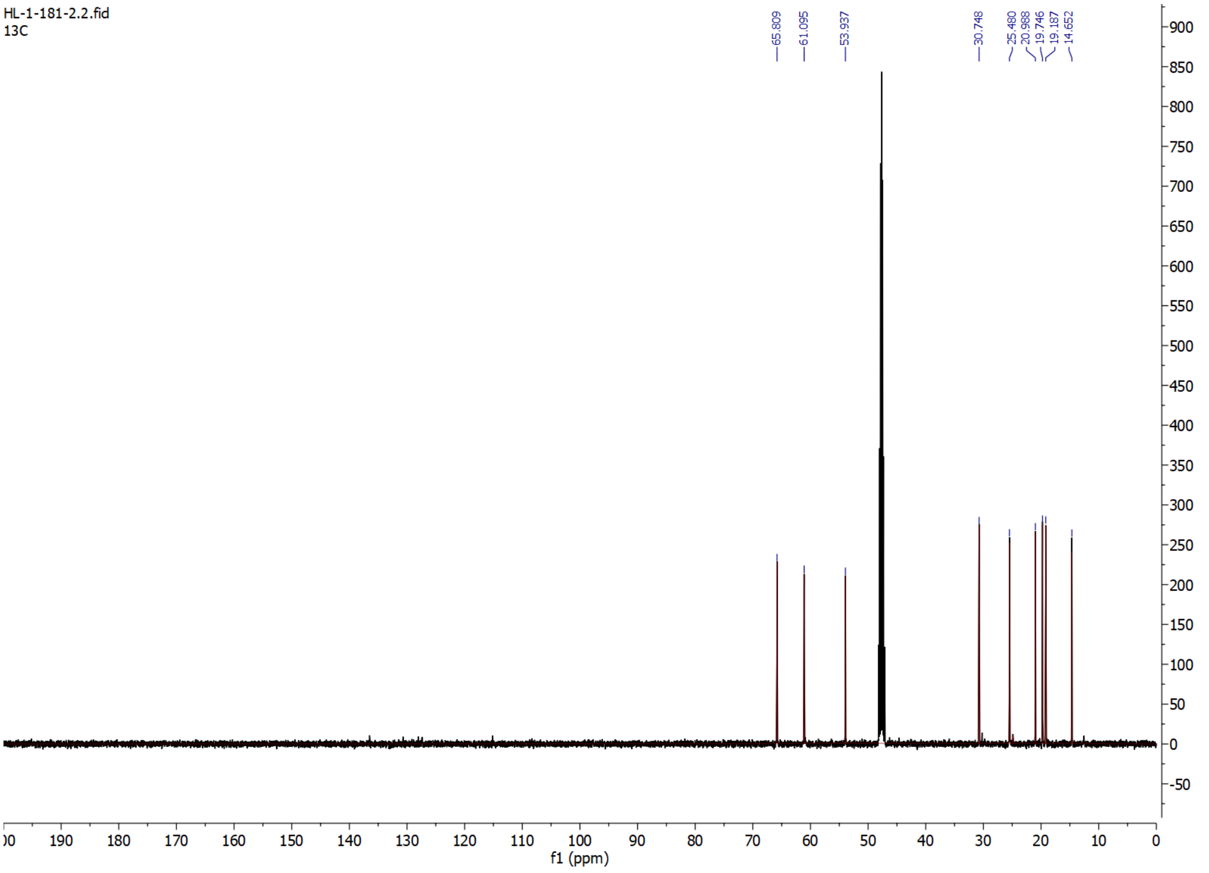
HL-1-181-2.2.fid
13C
65.809
61.095
53.937
30.748
25.480
20.988
19.746
19.187
14.652
f1 (ppm)

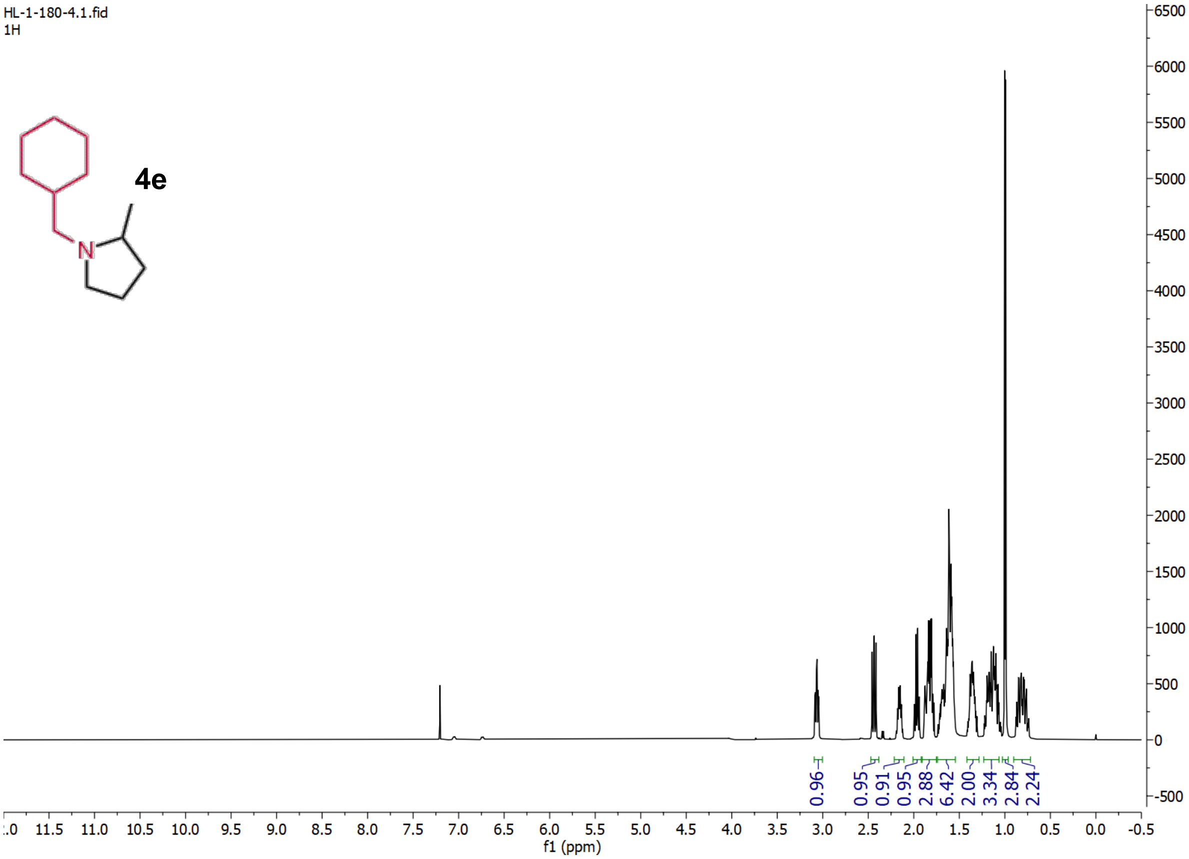
HL-1-180-4.1.fid
1H
4e
N
0.96
0.95
0.91
0.95
2.88
6.42
2.00
3.34
2.84
2.24
f1 (ppm)

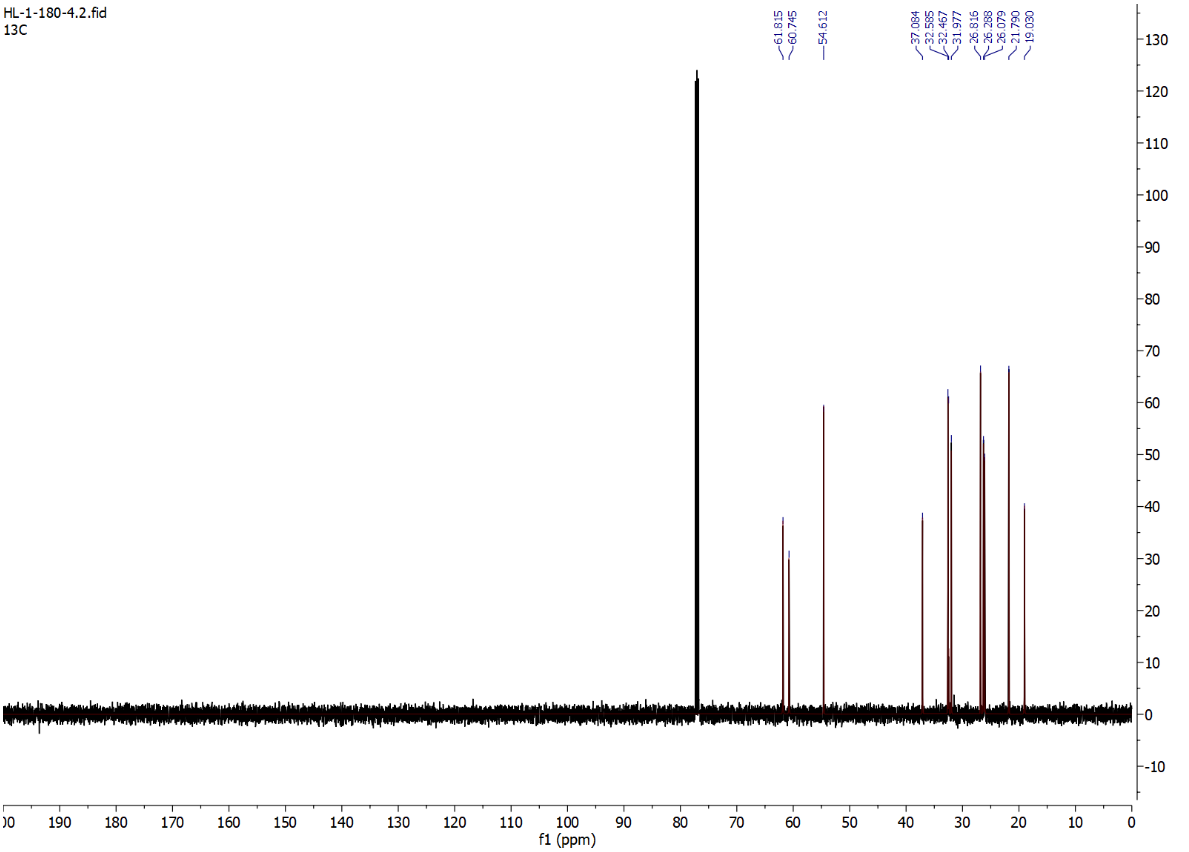
HL-1-180-4.2.fid
13C
61.815
60.745
54.612
37.084
32.585
32.467
31.977
26.816
26.288
26.079
21.790
19.030
f1 (ppm)

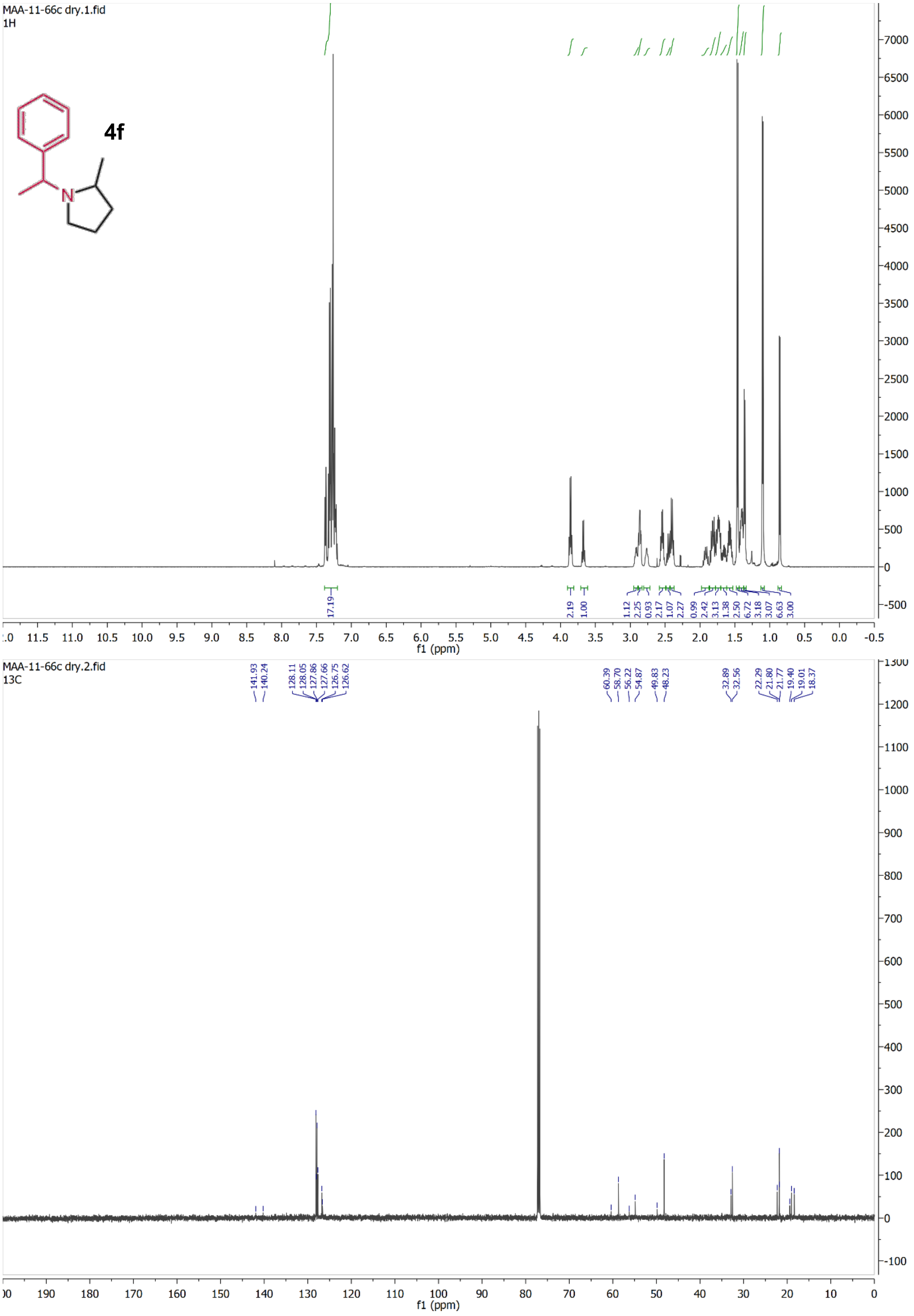
MAA-11-66c dry.1.fid
1H
4f
N
f1 (ppm)
MAA-11-66c dry.2.fid
13C
141.93
140.24
128.11
128.05
127.86
127.66
126.75
126.62
60.39
58.70
56.22
54.87
49.83
48.23
32.89
32.56
22.29
21.80
21.77
19.40
19.01
18.37
f1 (ppm)

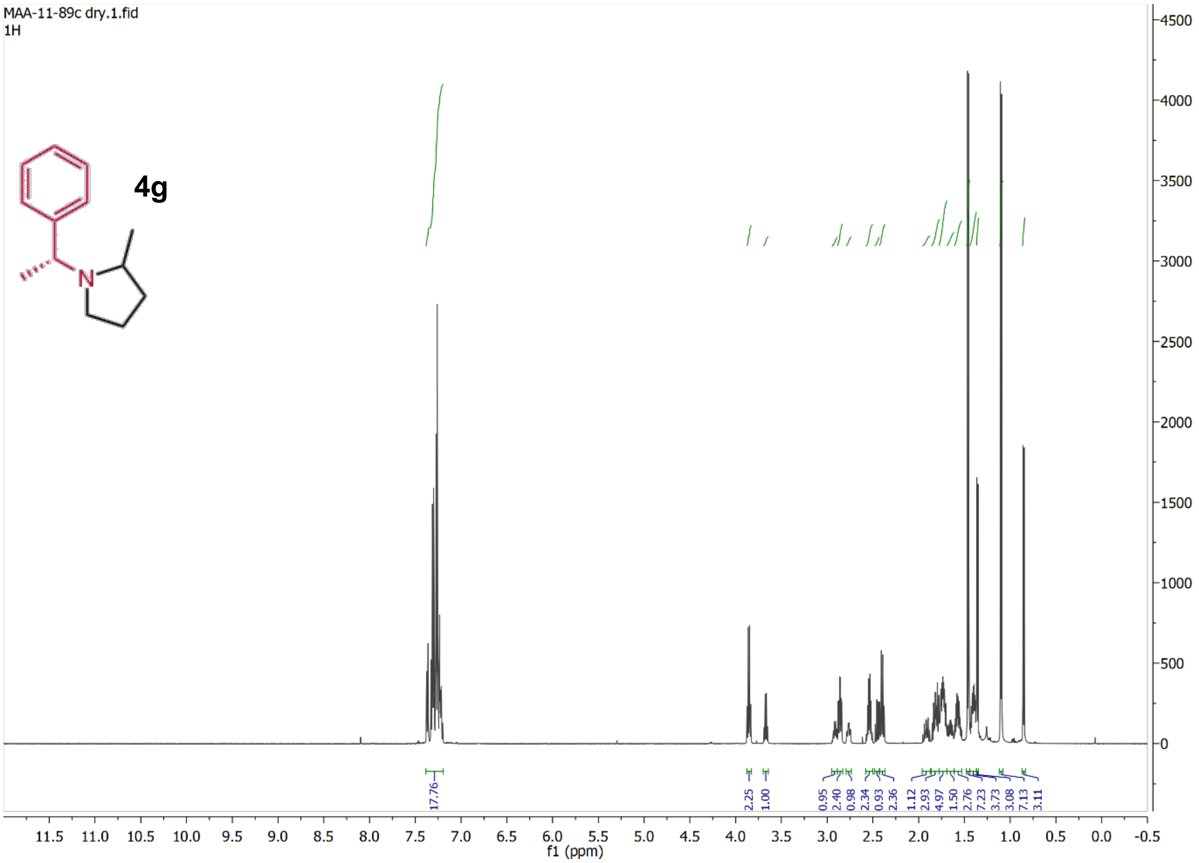
MAA-11-89c dry.1.fid
1H
4g
N
17.76
2.25
1.00
0.95
2.40
0.98
2.34
0.93
2.36
1.12
2.93
4.97
1.50
2.76
7.23
3.73
3.08
7.13
3.11
f1 (ppm)

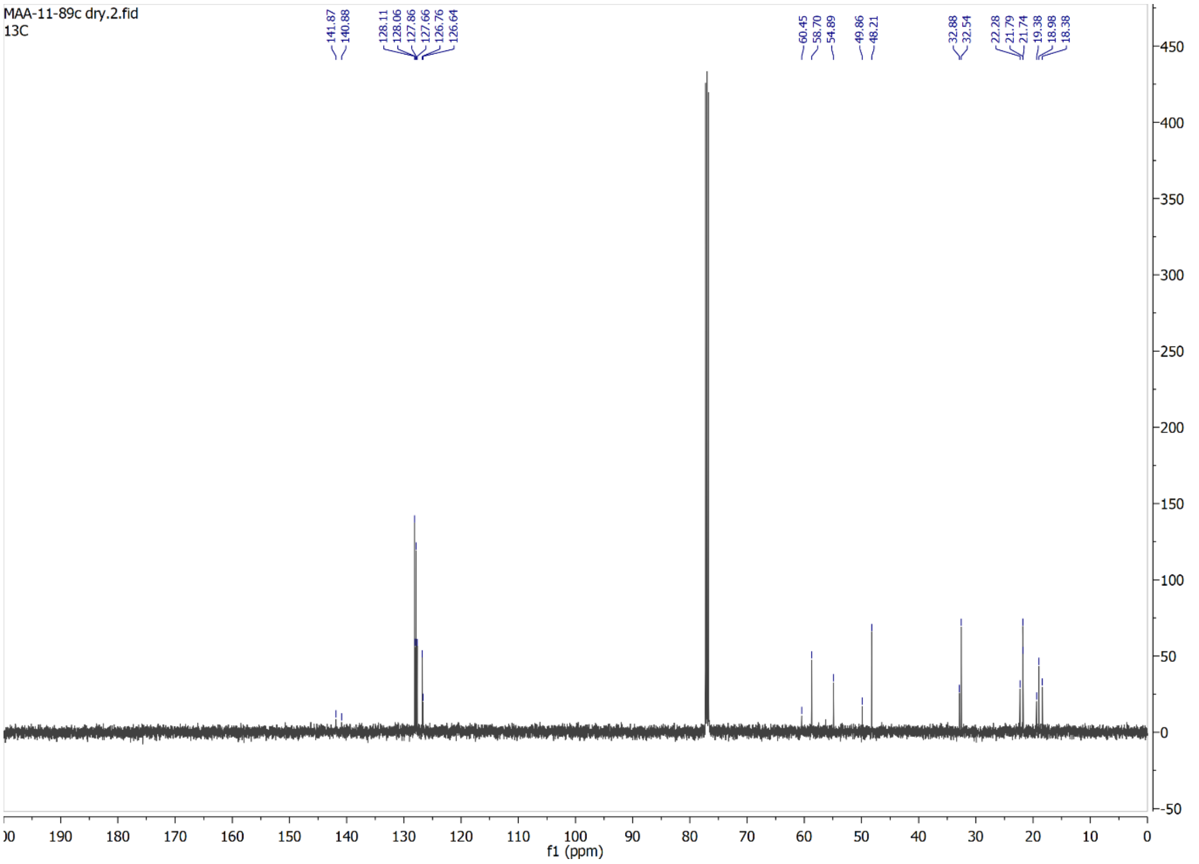
MAA-11-89c dry.2.fid
13C
141.87
140.88
128.11
128.06
127.86
127.66
126.76
126.64
60.45
58.70
54.89
49.86
48.21
32.88
32.54
22.28
21.79
21.74
19.38
18.98
18.38
f1 (ppm)

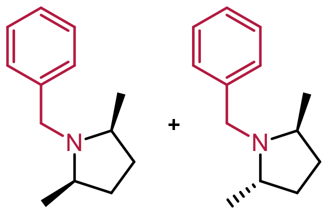

4h

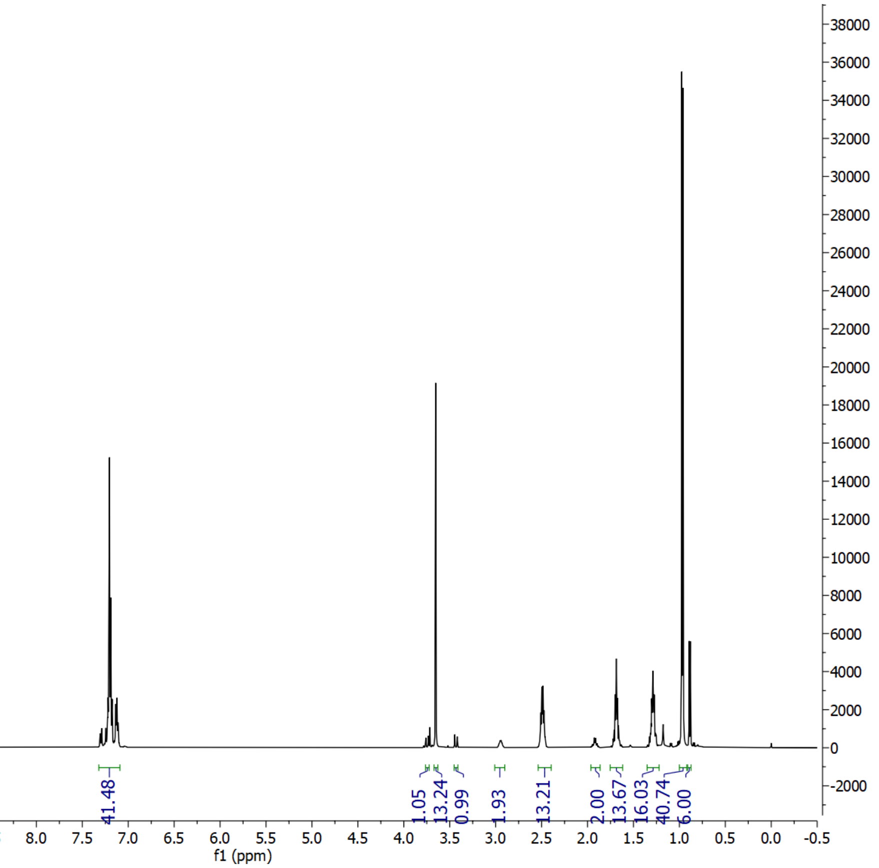

HL-2-87.6.1.1.fid
1H
41.48
1.05
13.24
0.99
1.93
13.21
2.00
13.67
16.03
40.74
6.00
f1 (ppm)

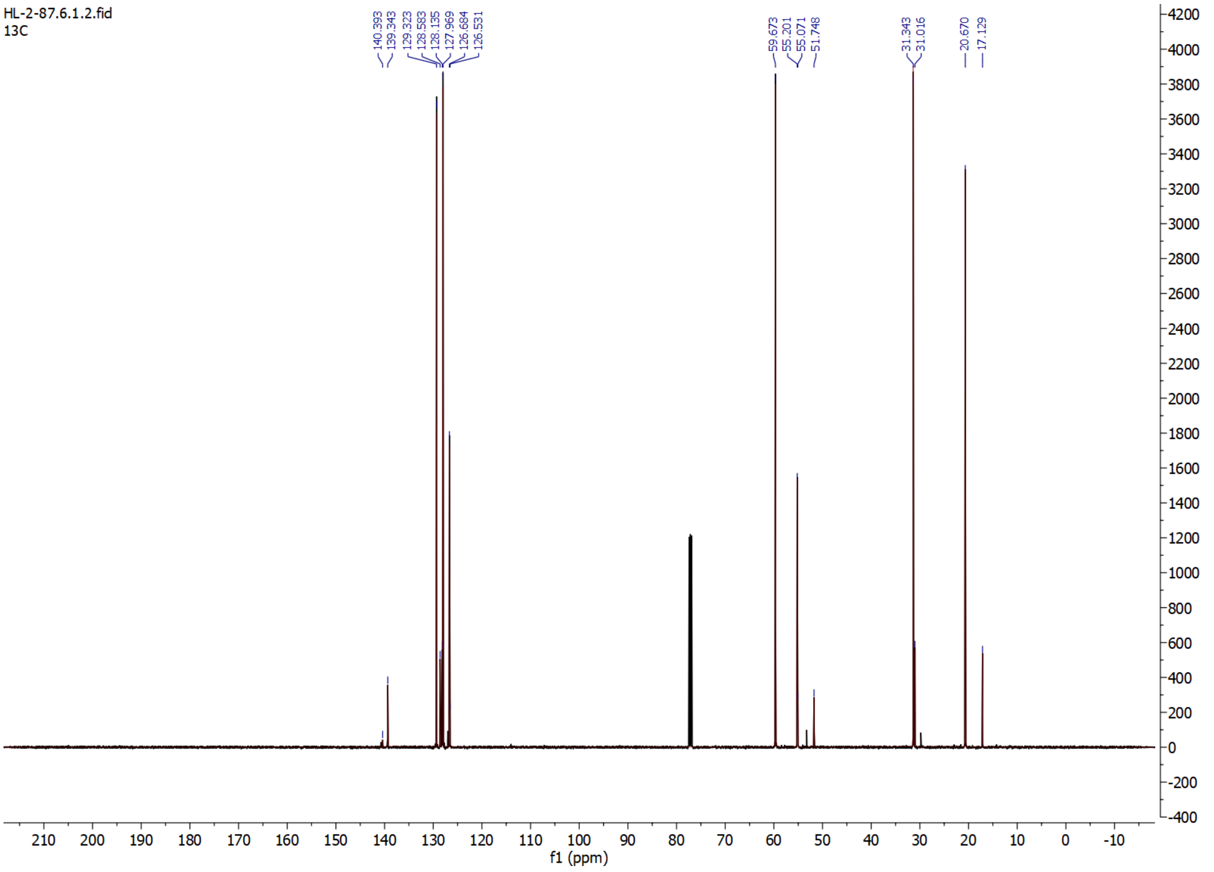

HL-2-87.6.1.2.fid
13C
140.393
139.343
129.323
128.583
128.135
127.969
126.684
126.531
59.673
55.201
55.071
51.748
31.343
31.016
20.670
17.129
f1 (ppm)

HL-2-44.1.fid
1H NMR

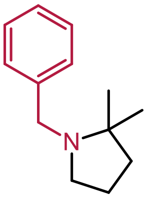

4i

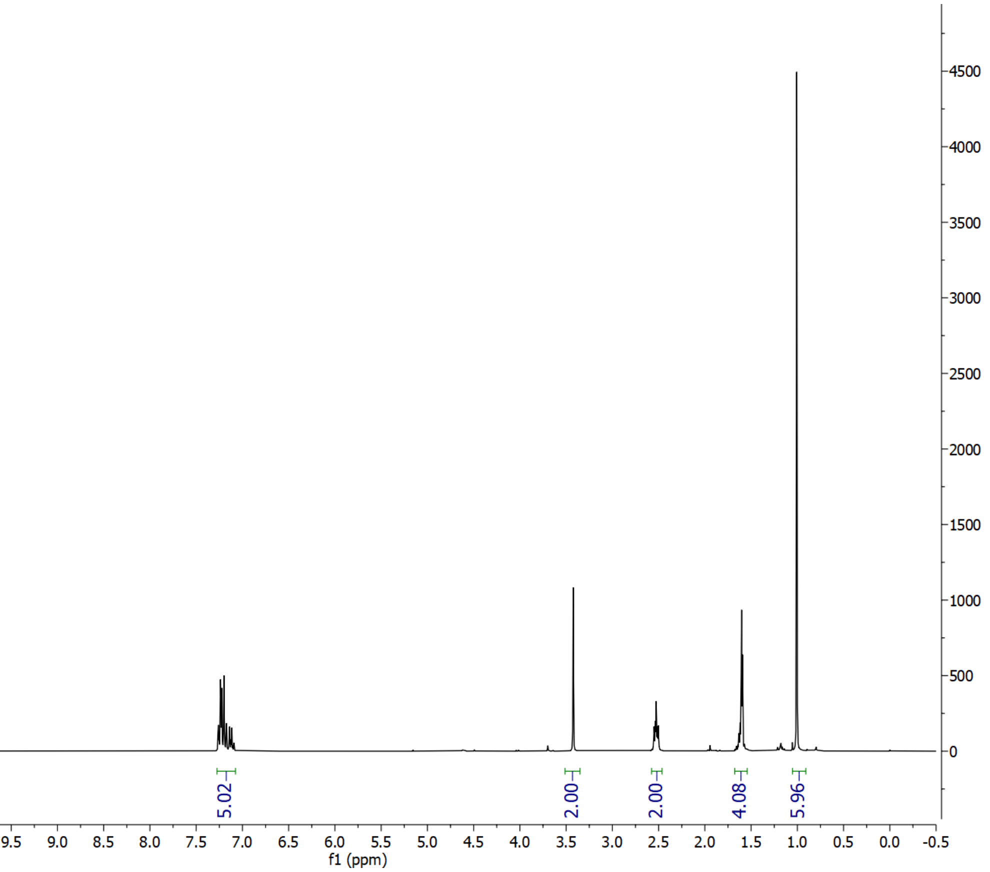

HL-2-44.2.fid
13C NMR with proton decoupling

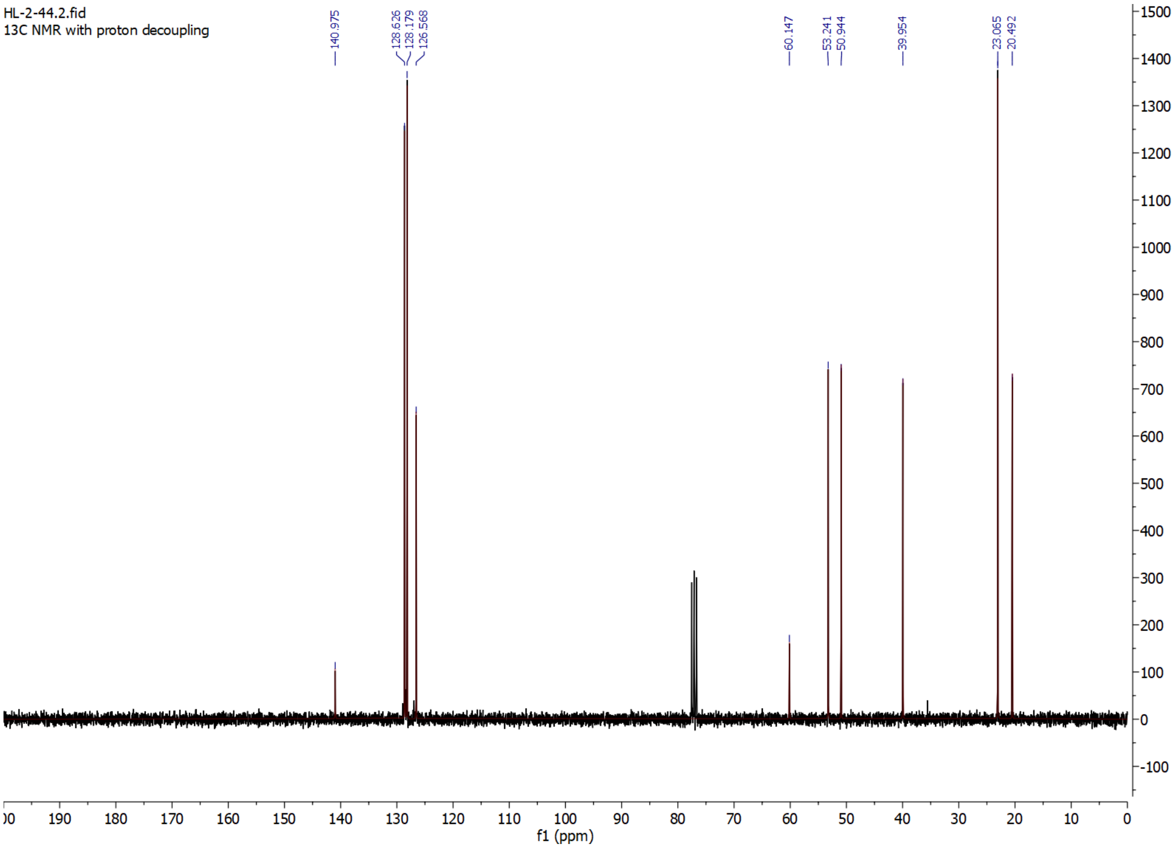

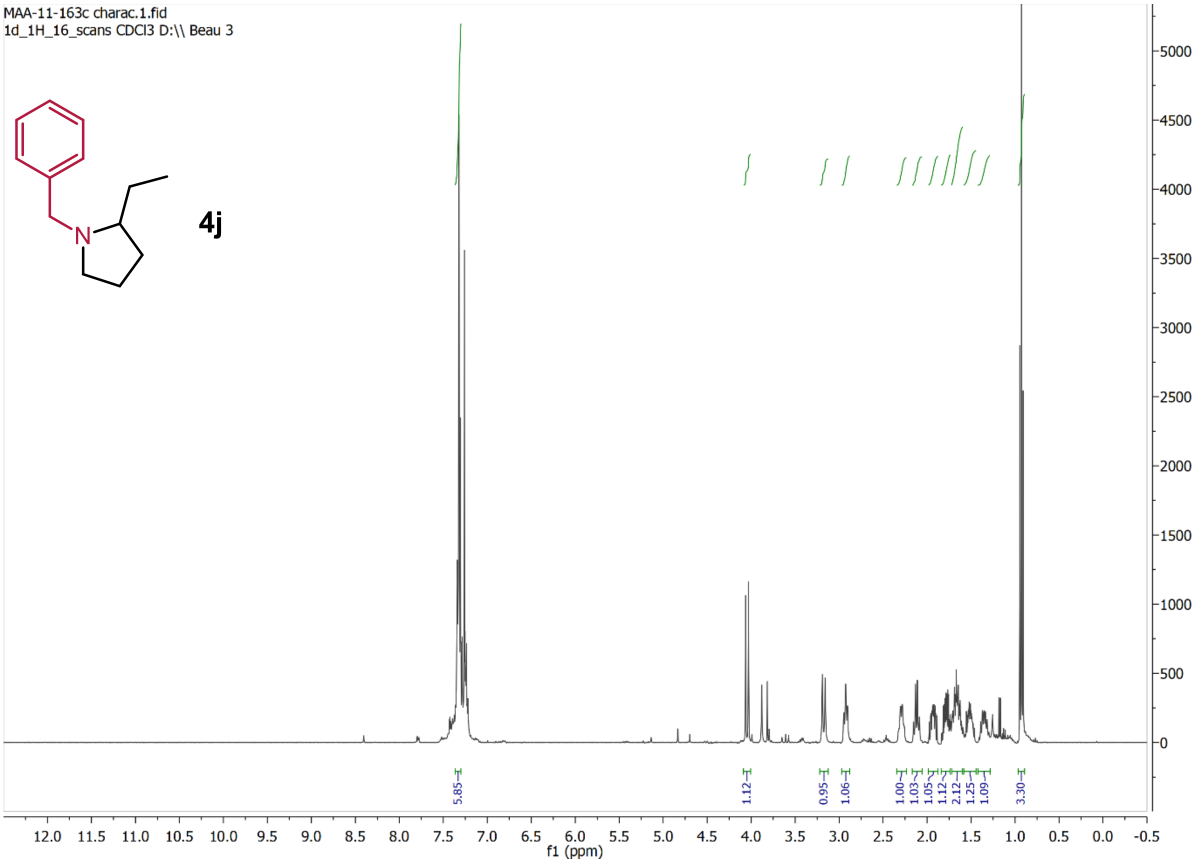
MAA-11-163c charac.1.fid
1d_1H_16_scans CDCl3 D:\\ Beau 3
N
4j
5.85
1.12
0.95
1.06
1.00
1.03
1.05
1.12
2.12
1.25
1.09
3.30
f1 (ppm)

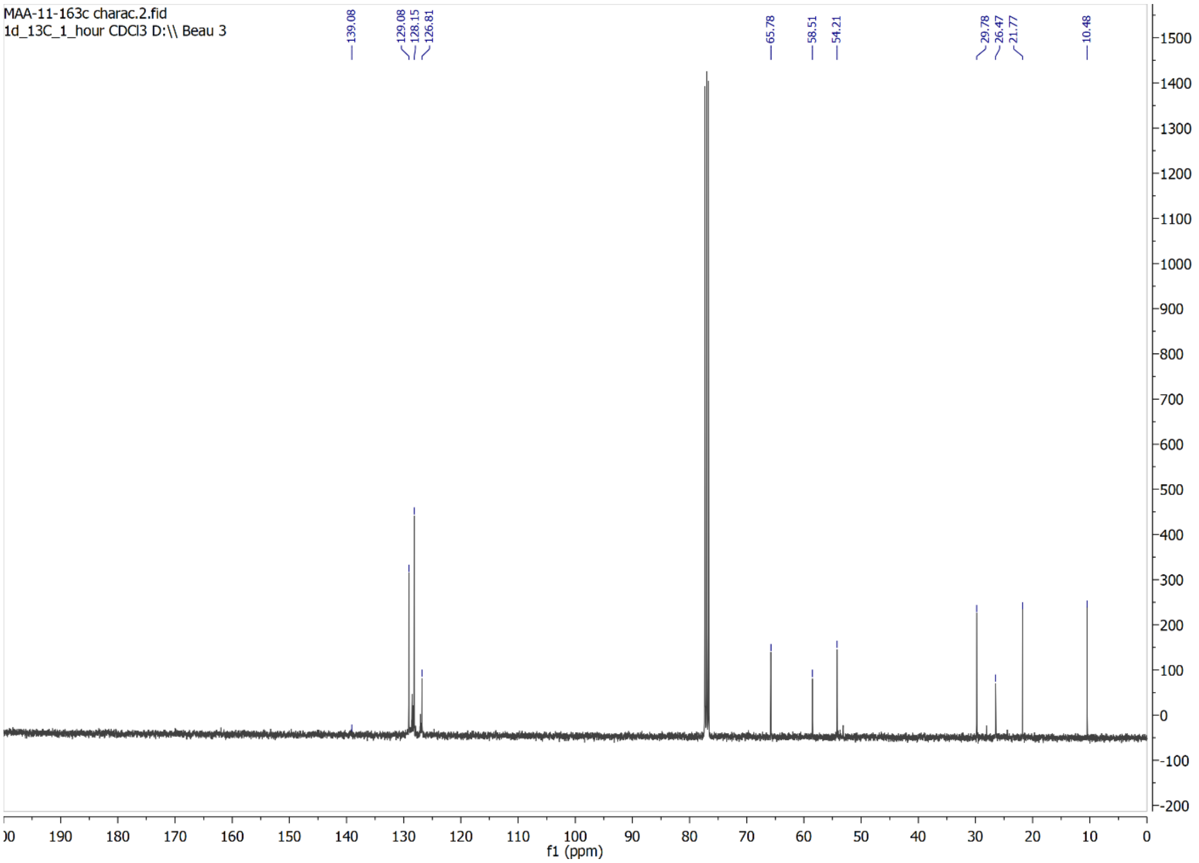
MAA-11-163c charac.2.fid
1d_13C_1_hour CDCl3 D:\\ Beau 3
139.08
129.08
128.15
126.81
65.78
58.51
54.21
29.78
26.47
21.77
10.48
f1 (ppm)

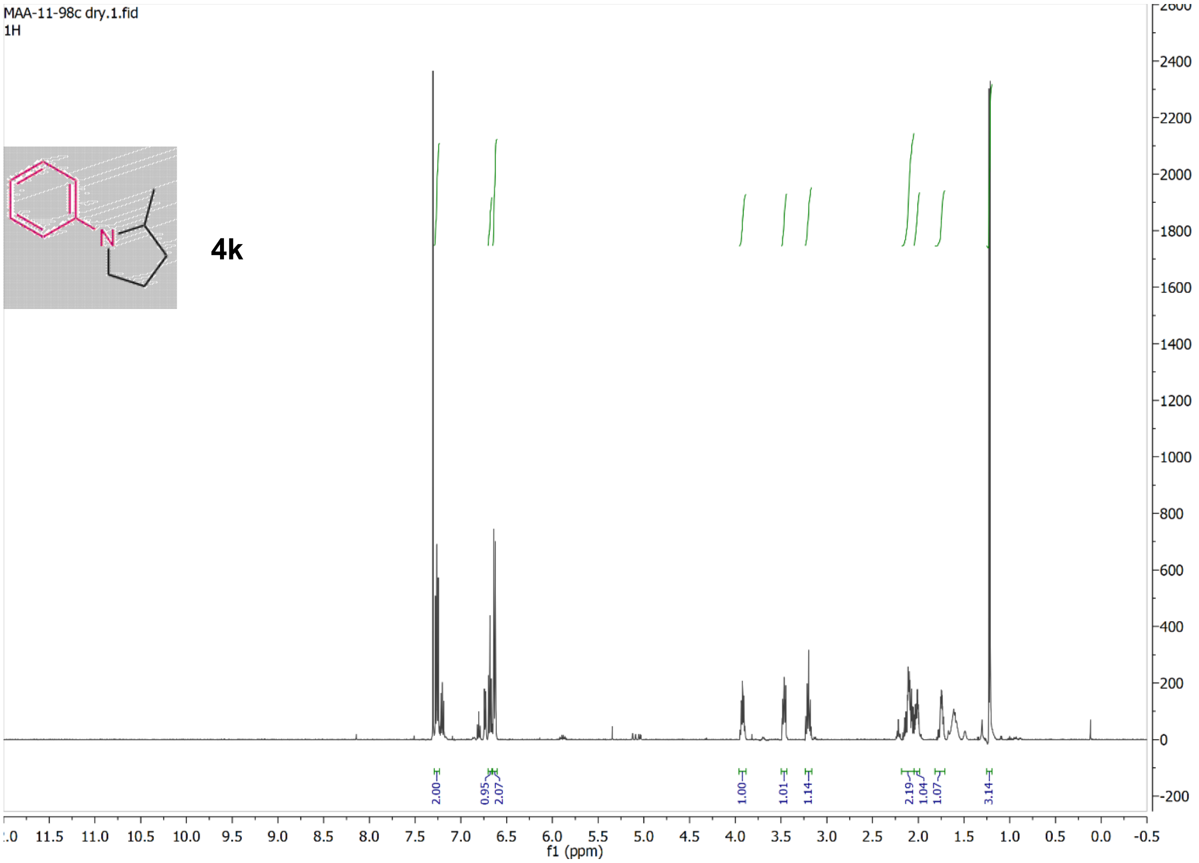
MAA-11-98c dry.1.fid
1H
4k
2.00
0.95
2.07
1.00
1.01
1.14
2.19
1.04
1.07
3.14
f1 (ppm)

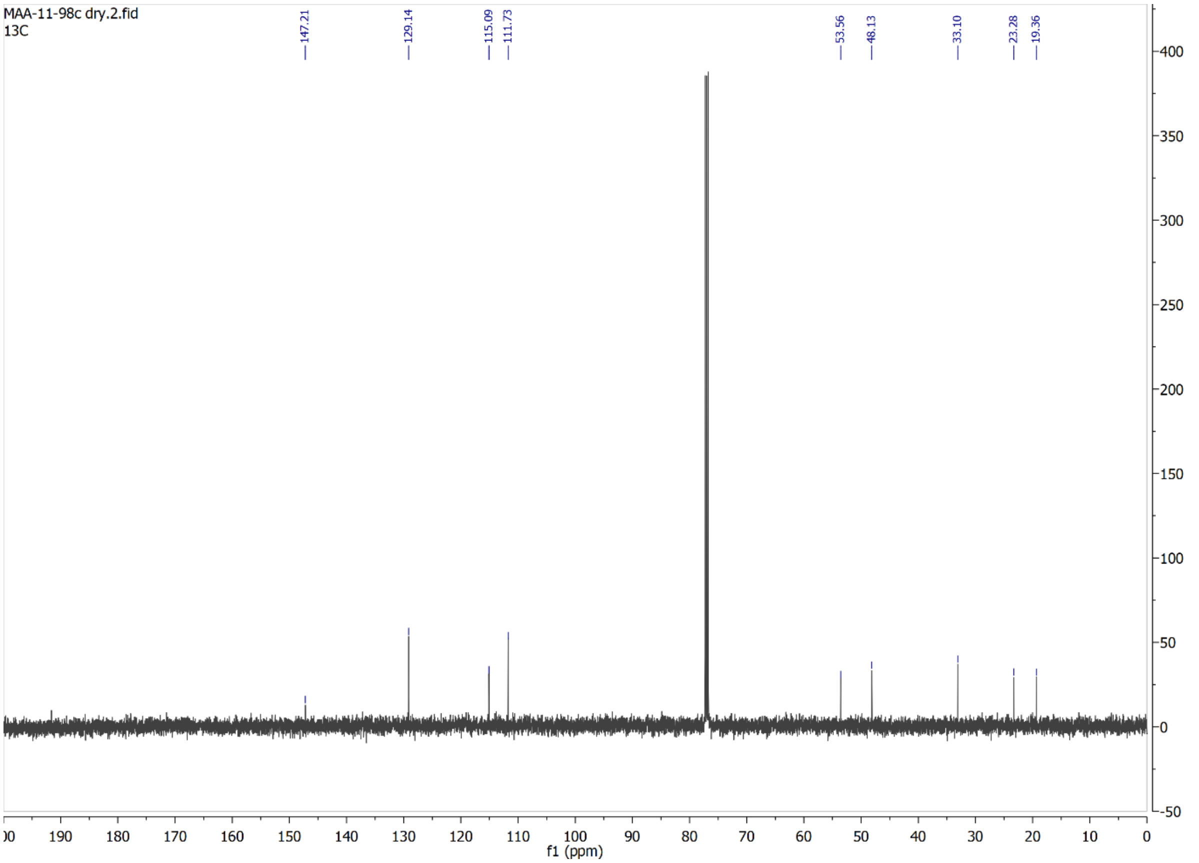
MAA-11-98c dry.2.fid
13C
147.21
129.14
115.09
111.73
53.56
48.13
33.10
23.28
19.36
f1 (ppm)

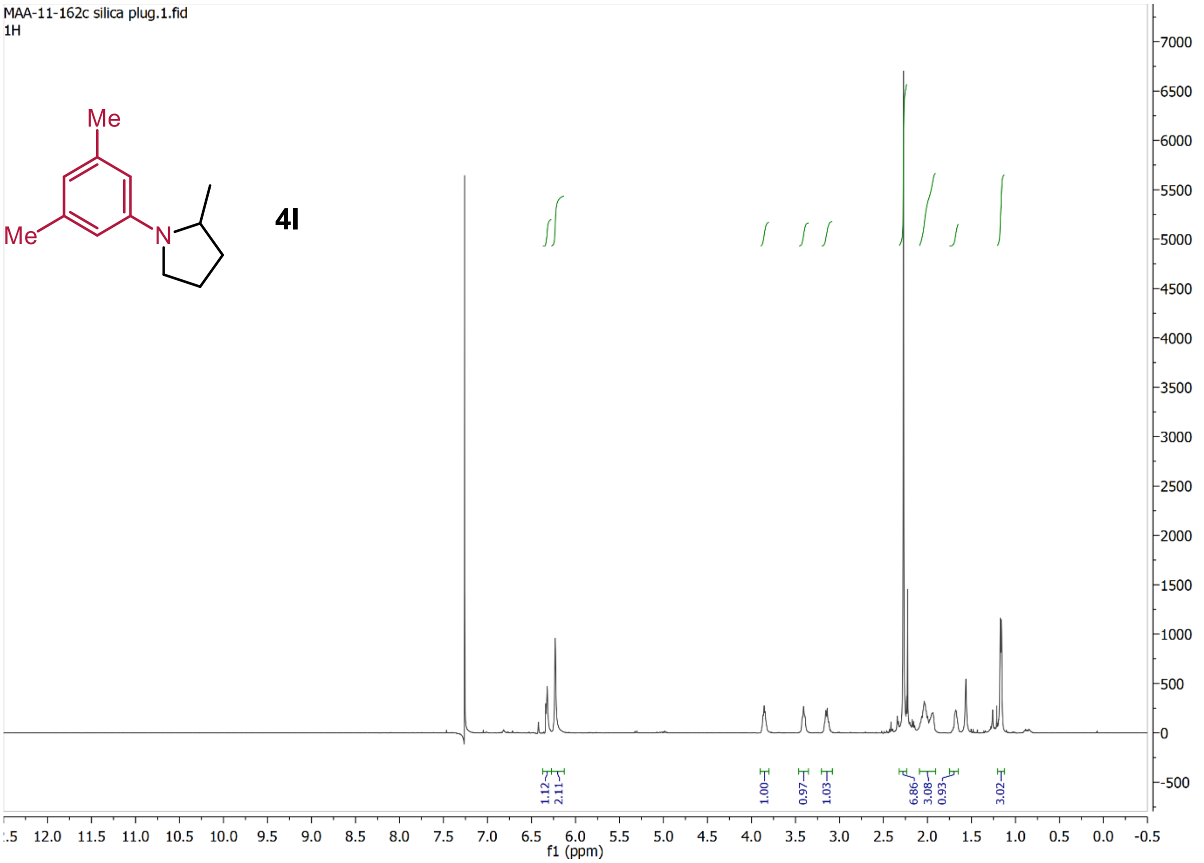

MAA-11-162c silica plug.1.fid
1H
Me
Me
N
4l
1.12
2.11
1.00
0.97
1.03
6.86
3.08
0.93
3.02
f1 (ppm)

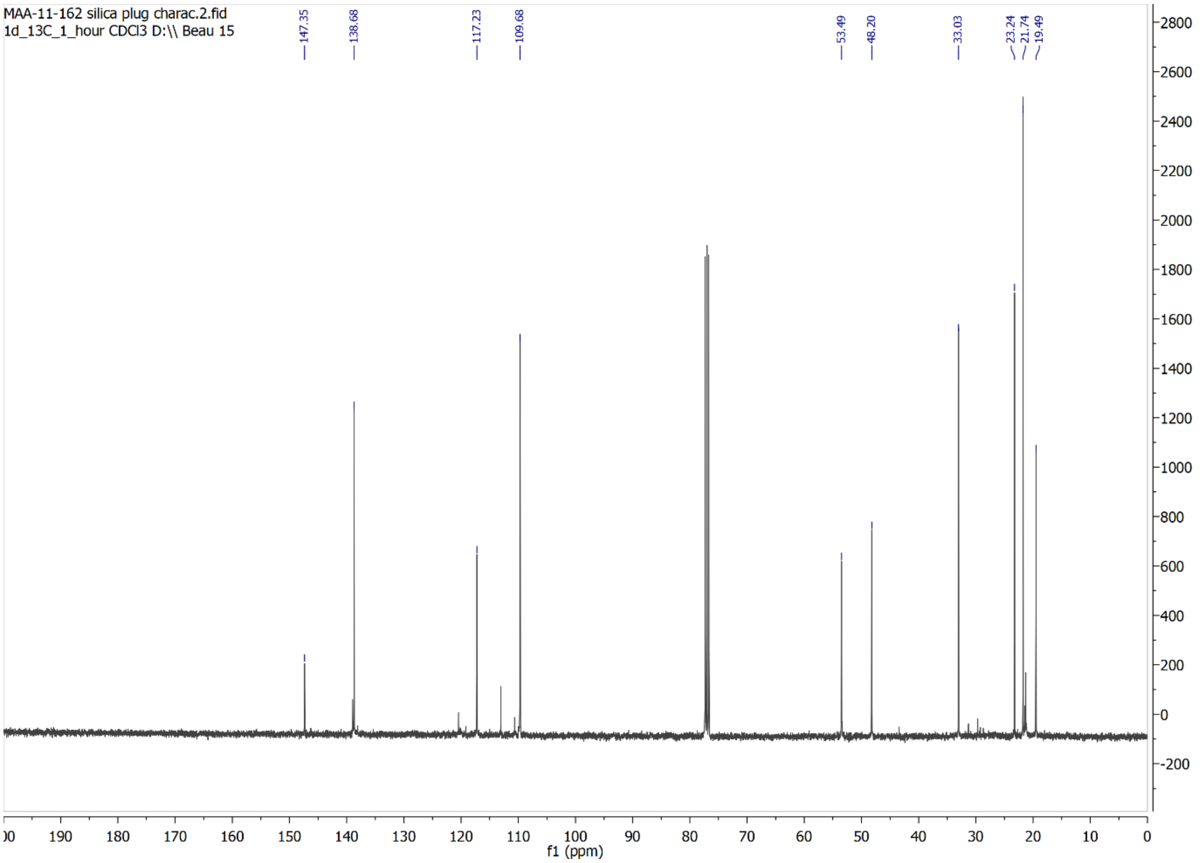

MAA-11-162 silica plug charac.2.fid
1d_13C_1_hour CDCl3 D:\\ Beau 15
147.35
138.68
117.23
109.68
53.49
48.20
33.03
23.24
21.74
19.49
f1 (ppm)

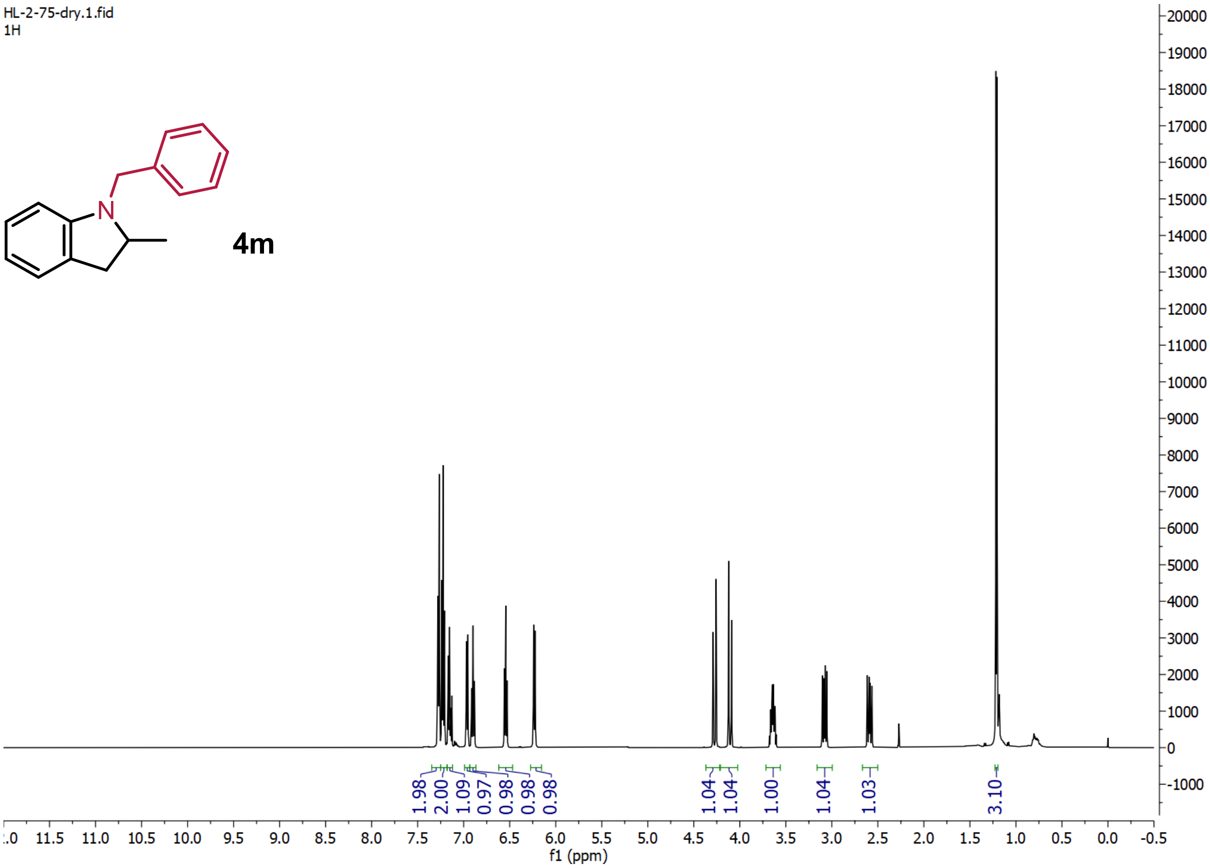
HL-2-75-dry.1.fid
1H
4m
N
1.98
2.00
1.09
0.97
0.98
0.98
0.98
1.04
1.04
1.00
1.04
1.03
3.10
f1 (ppm)

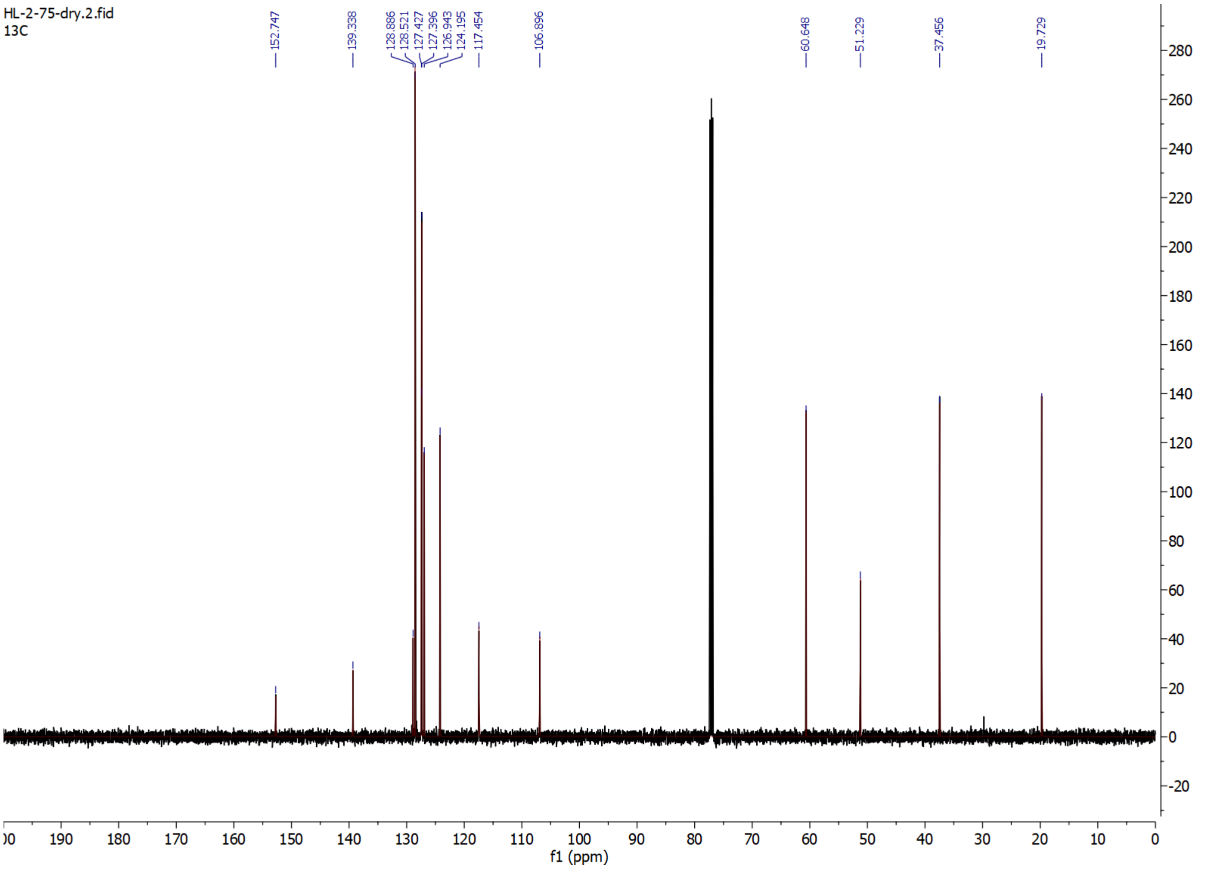
HL-2-75-dry.2.fid
13C
152.747
139.338
128.886
128.521
127.427
127.396
126.943
124.195
117.454
106.896
60.648
51.229
37.456
19.729
f1 (ppm)

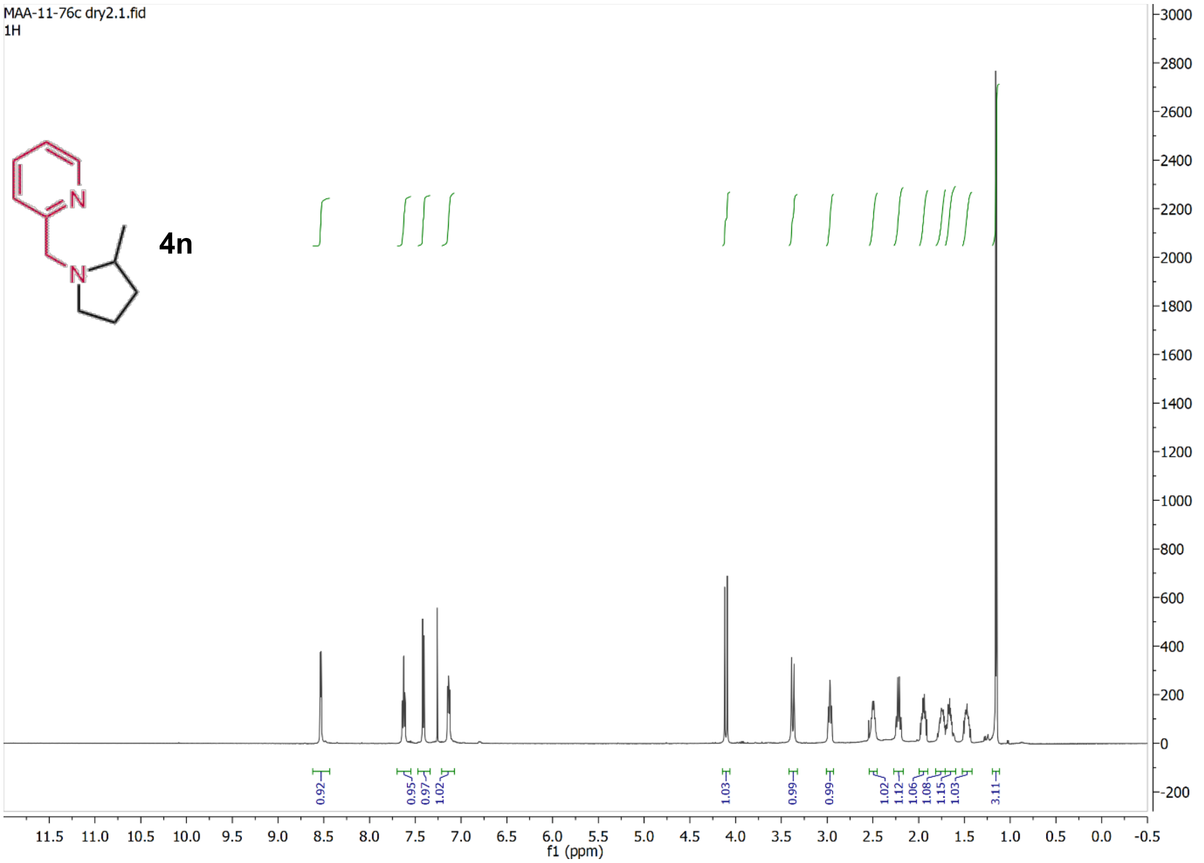
MAA-11-76c dry2.1.fid
1H
4n
N
0.92
0.95
0.97
1.02
1.03
0.99
0.99
1.02
1.12
1.06
1.08
1.15
1.03
3.11
f1 (ppm)

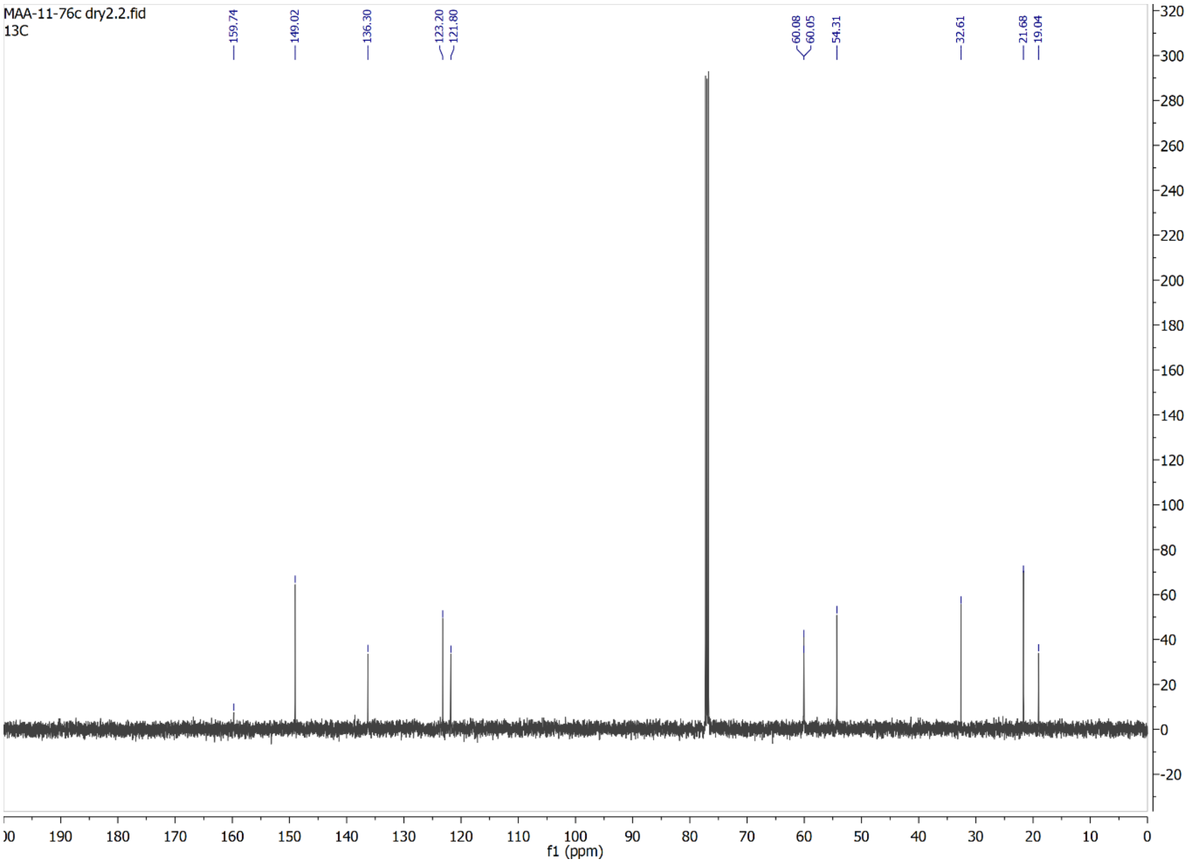
MAA-11-76c dry2.2.fid
13C
159.74
149.02
136.30
123.20
121.80
60.08
60.05
54.31
32.61
21.68
19.04
f1 (ppm)

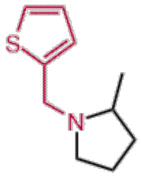

4o

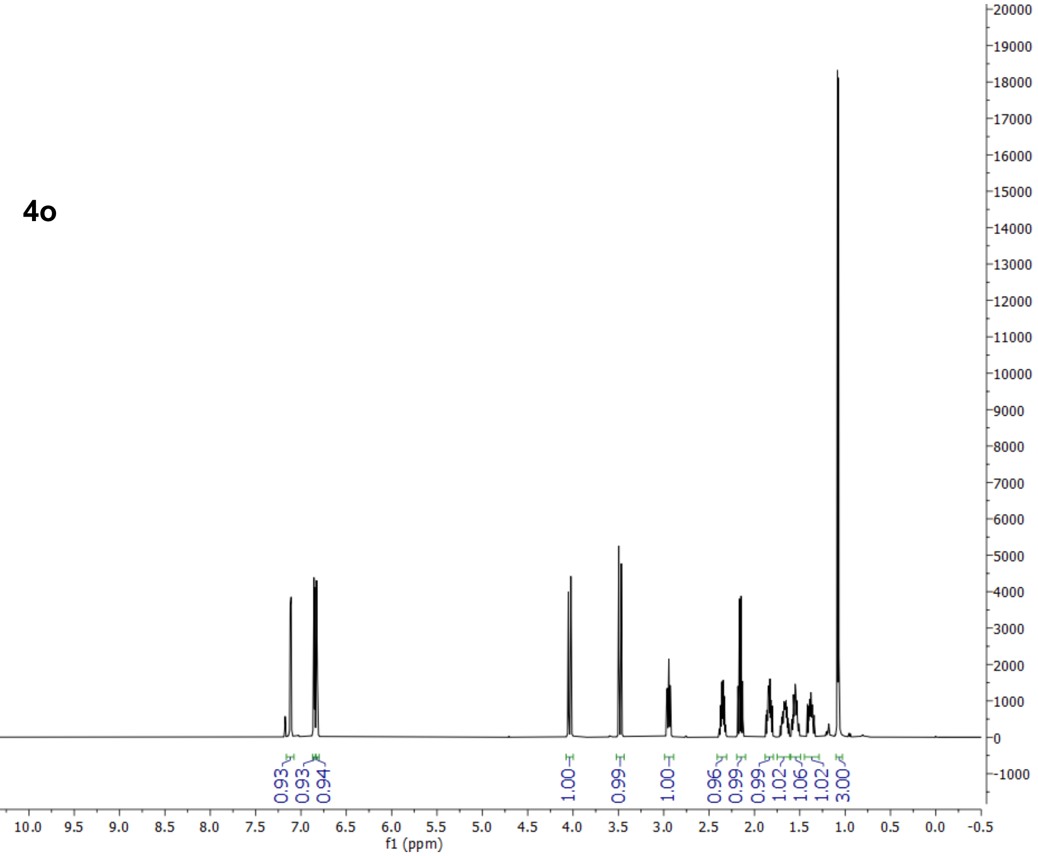

HL-1-179-2.1.fid
1H
0.93
0.93
0.94
1.00
0.99
1.00
0.96
0.99
0.99
1.02
1.06
1.02
3.00
f1 (ppm)

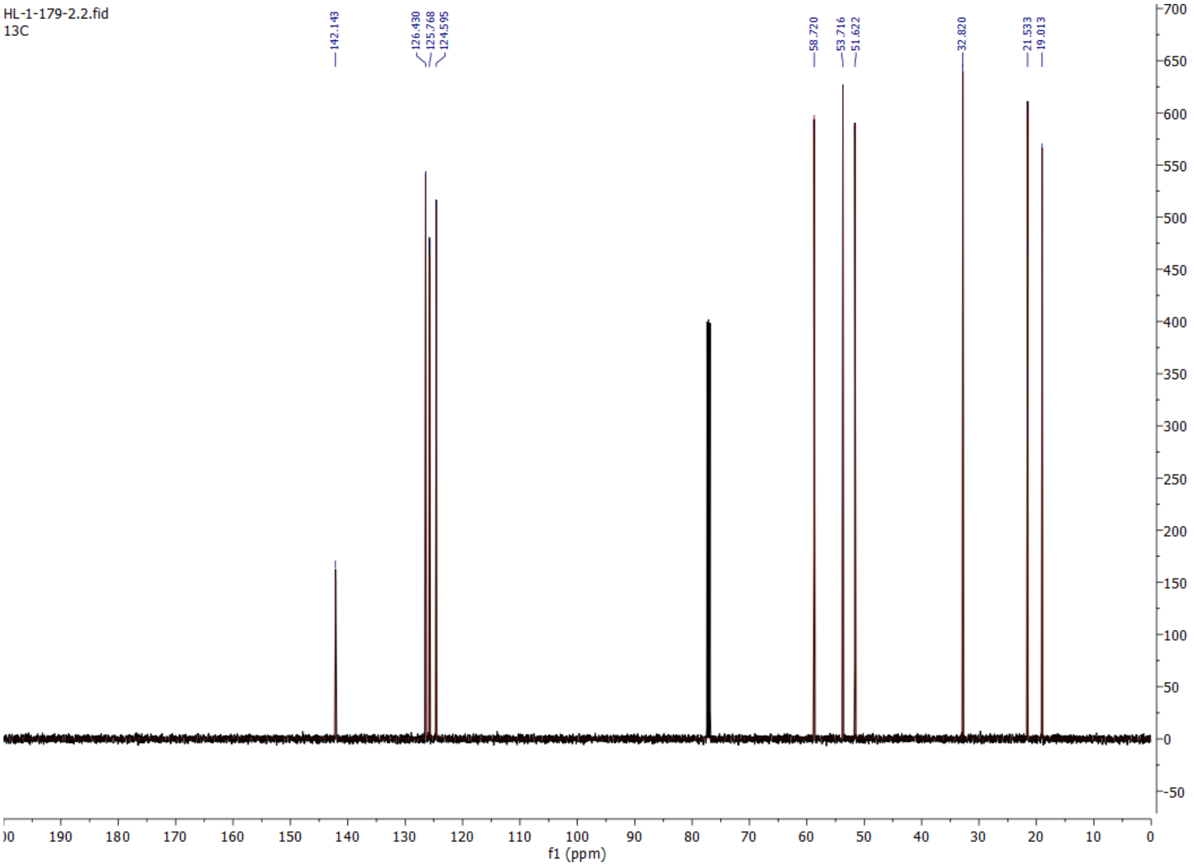

HL-1-179-2.2.fid
13C
142.143
126.430
125.768
124.595
58.720
53.716
51.622
32.820
21.533
19.013
f1 (ppm)

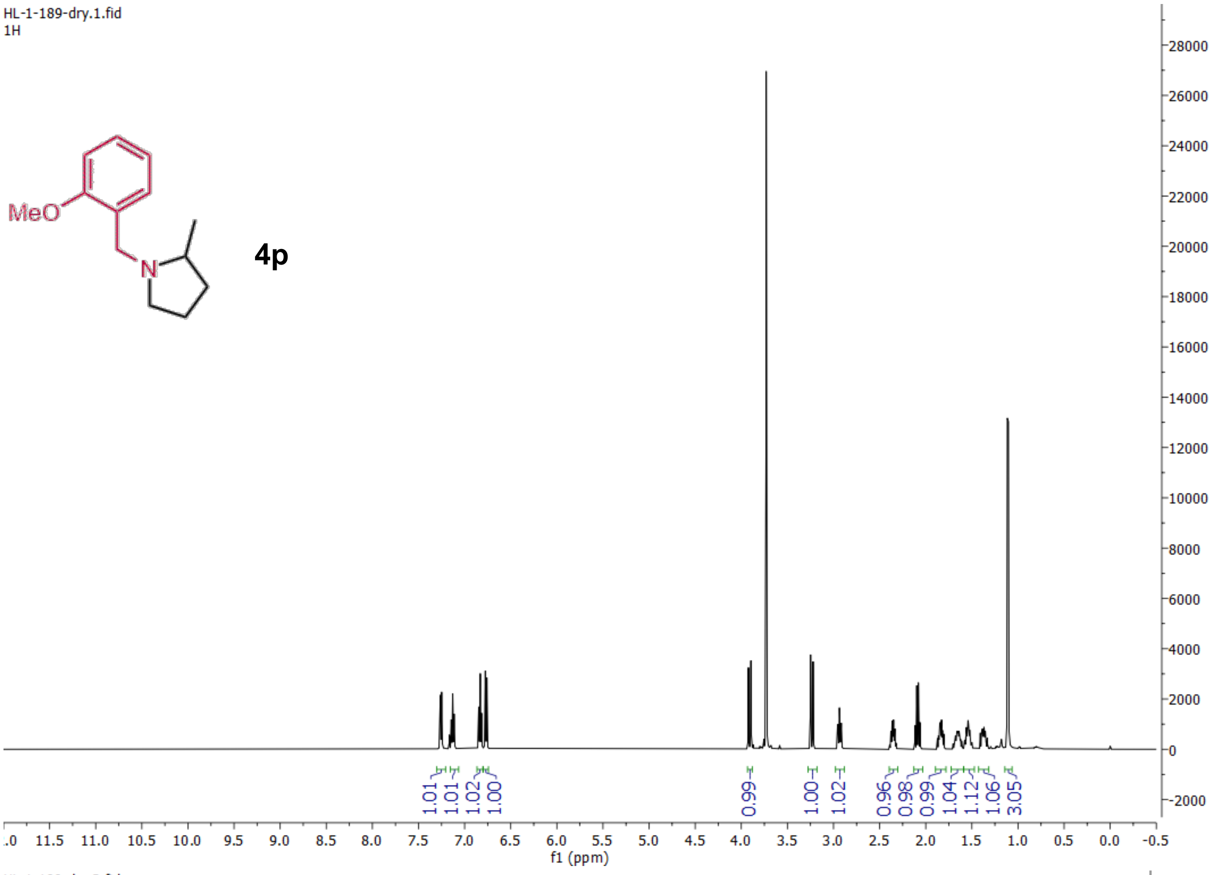
HL-1-189-dry.1.fid
1H
MeO
N
4p
1.01
1.01
1.02
1.00
0.99
1.00
1.02
0.96
0.98
0.99
1.04
1.12
1.06
3.05
f1 (ppm)

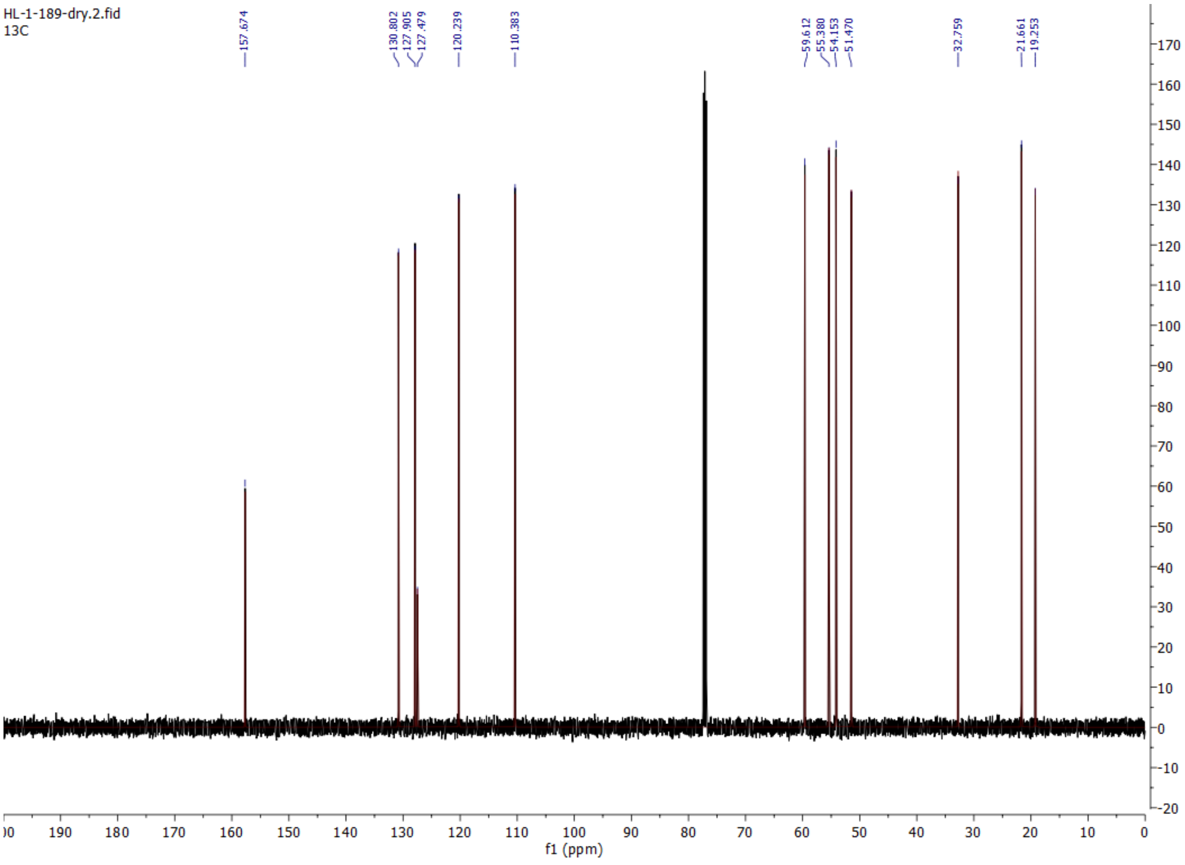
HL-1-189-dry.2.fid
13C
157.674
130.802
127.905
127.479
120.239
110.383
59.612
55.380
54.153
51.470
32.759
21.661
19.253
f1 (ppm)

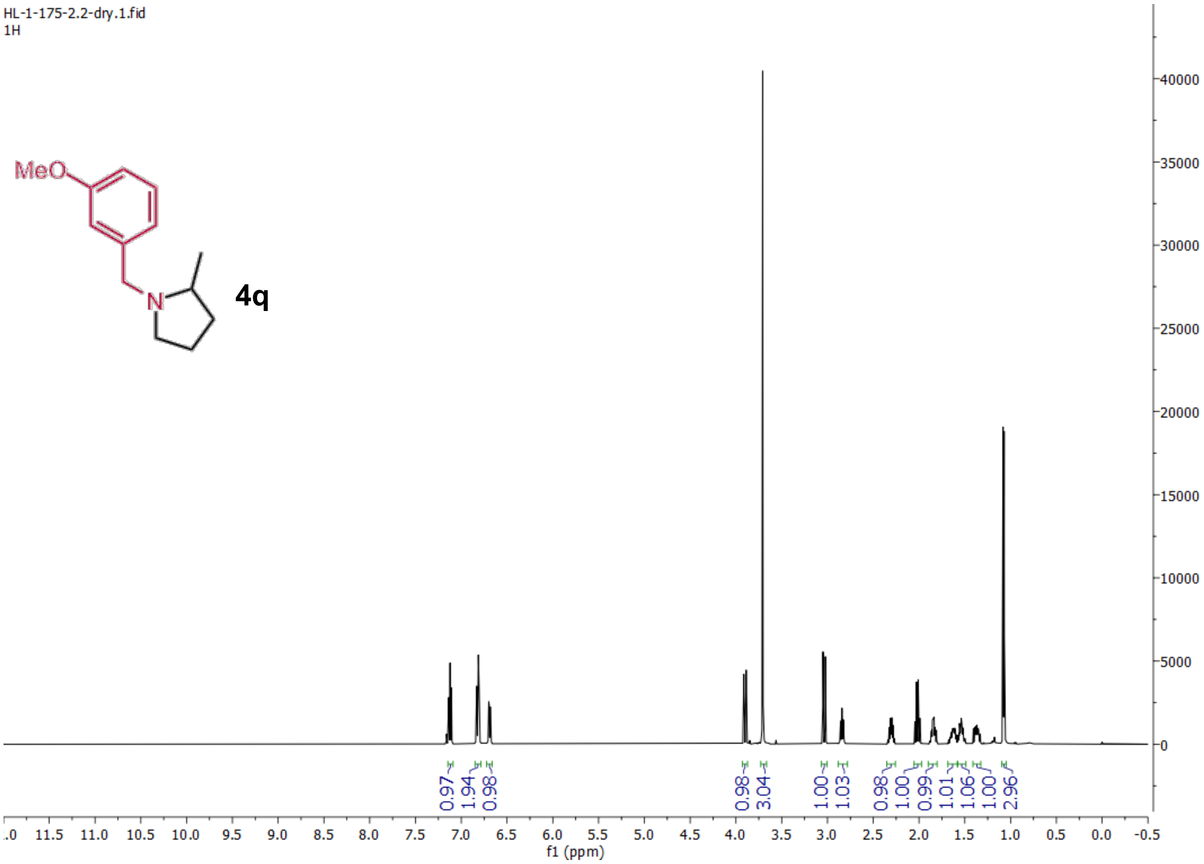
HL-1-175-2.2-dry.1.fid
1H
0.97
1.94
0.98
0.98
3.04
1.00
1.03
0.98
1.00
0.99
1.01
1.06
1.00
2.96
f1 (ppm)
MeO
N
4q

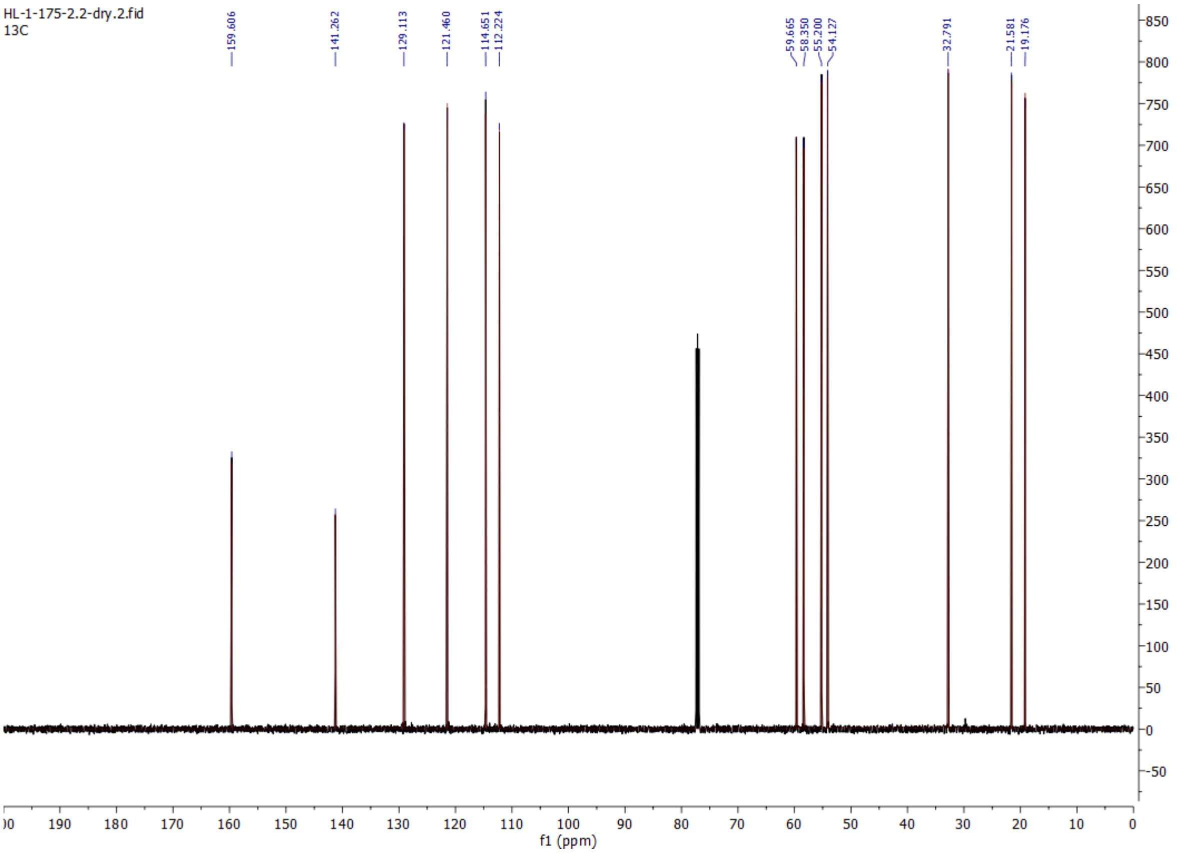
HL-1-175-2.2-dry.2.fid
13C
159.606
141.262
129.113
121.460
114.651
112.224
59.665
58.350
55.200
54.127
32.791
21.581
19.176
f1 (ppm)

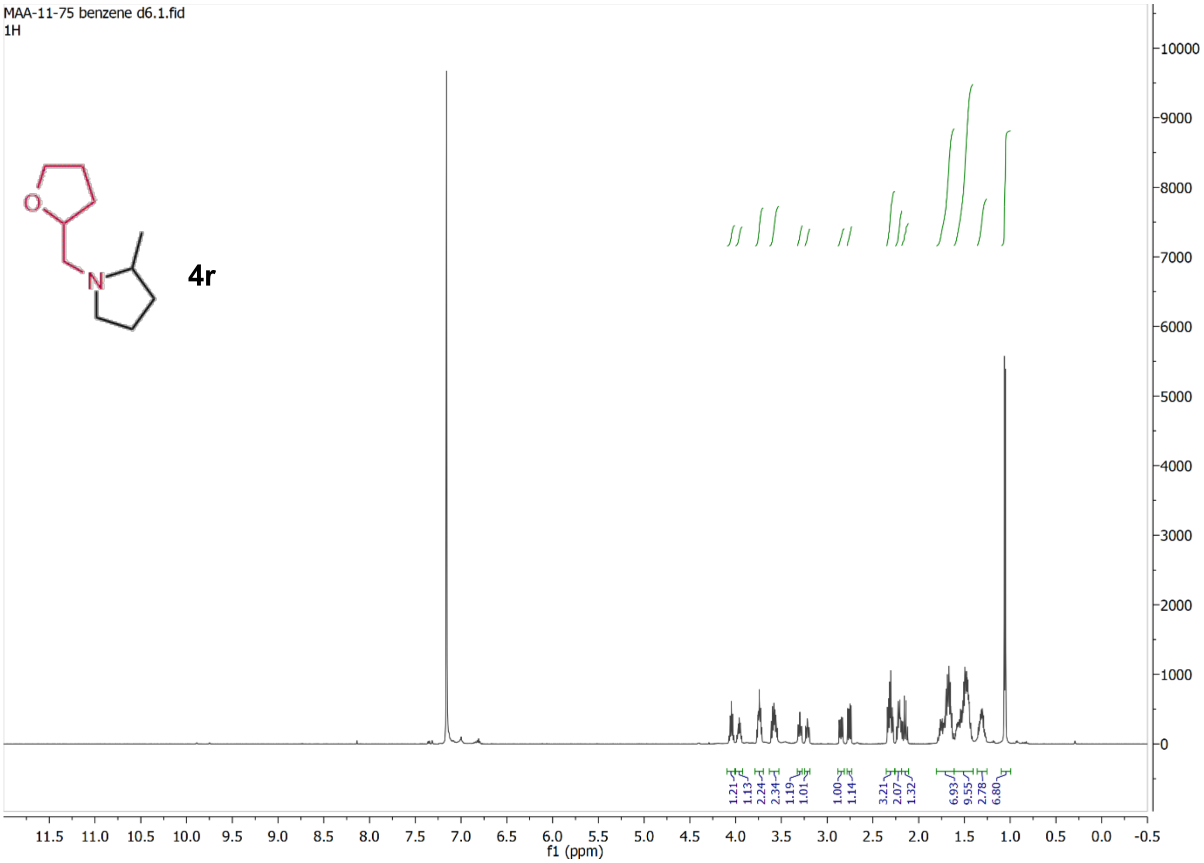
MAA-11-75 benzene d6.1.fid
1H
4r
1.21 1.13 2.24 2.34 1.19 1.01 1.00 1.14 3.21 2.07 1.32 6.93 9.55 2.78 6.80
f1 (ppm)

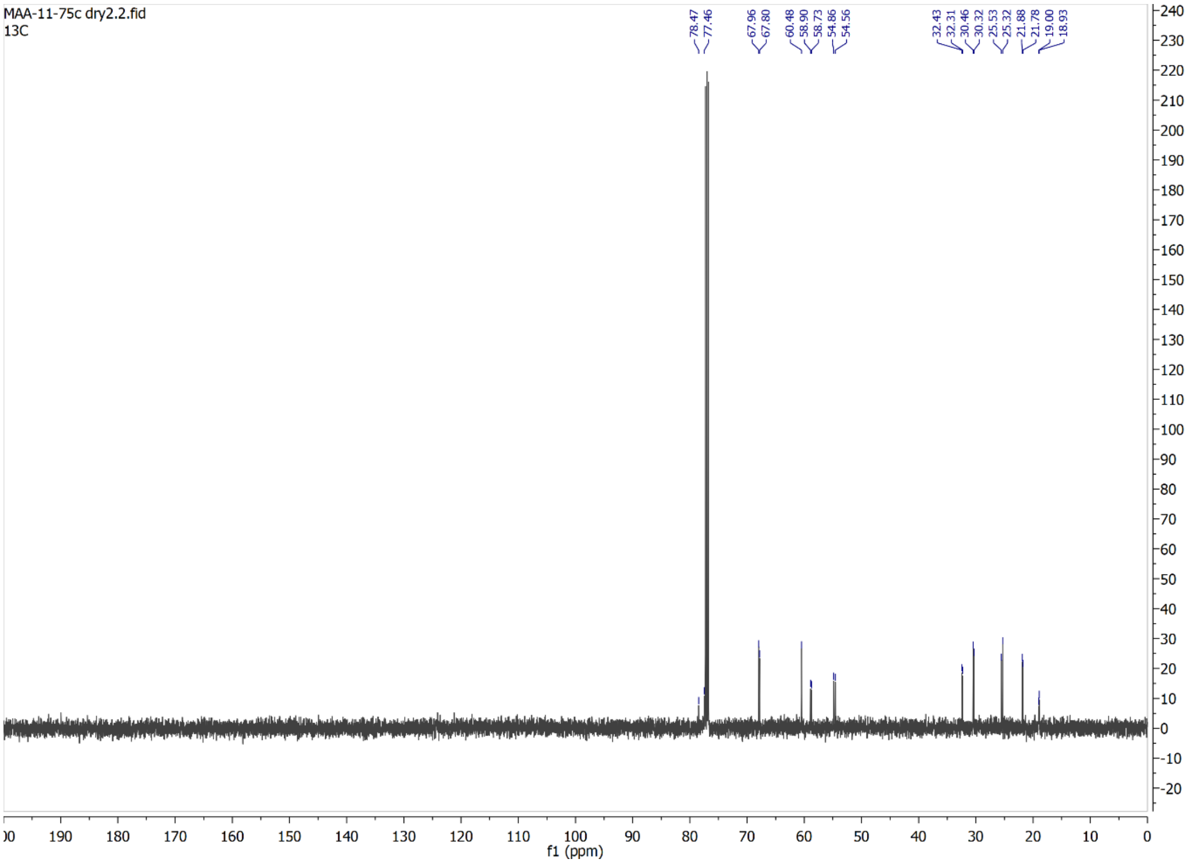
MAA-11-75c dry2.2.fid
13C
78.47 77.46 67.96 67.80 60.48 58.90 58.73 54.86 54.56 32.43 32.31 30.46 30.32 25.53 25.32 21.88 21.78 19.00 18.93
f1 (ppm)

4s

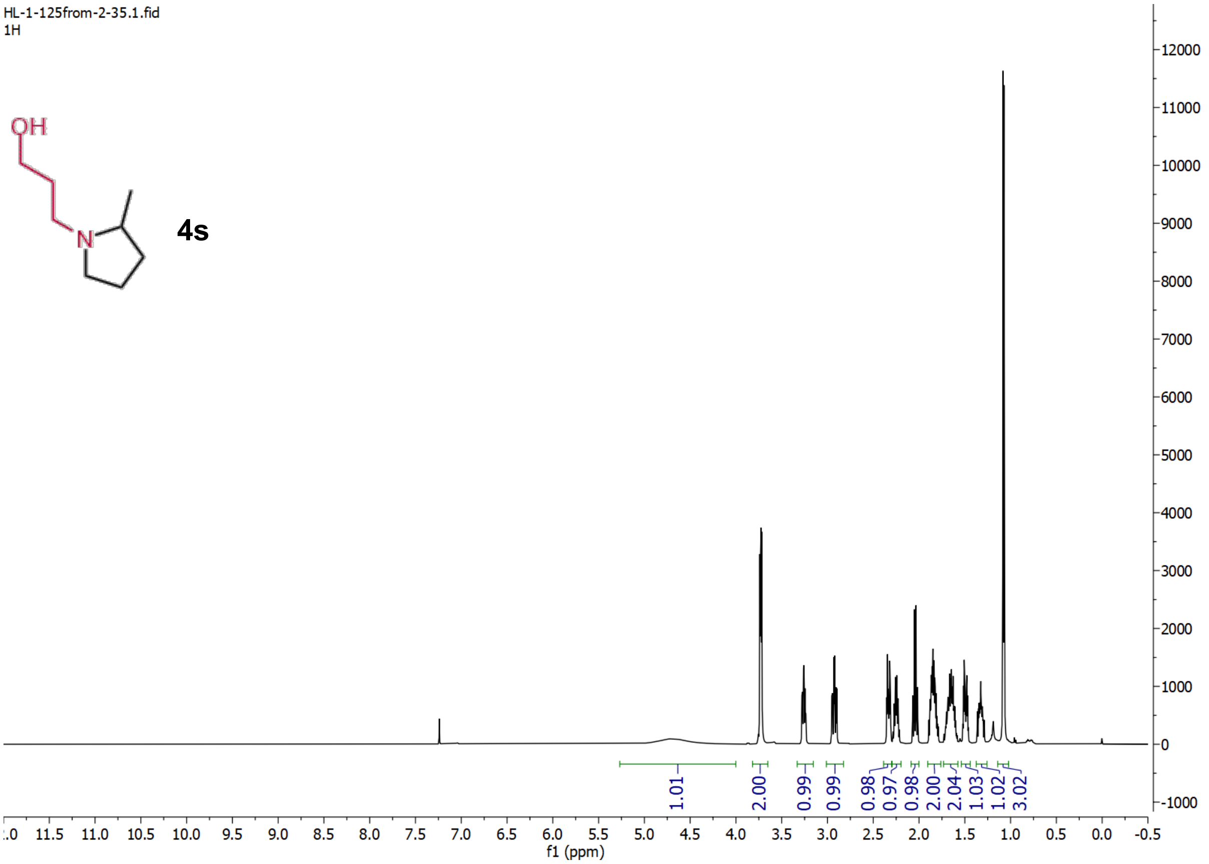

HL-1-125from-2-35.1.fid
1H
1.01
2.00
0.99
0.99
0.98
0.97
0.98
2.00
2.04
1.03
1.02
3.02
f1 (ppm)

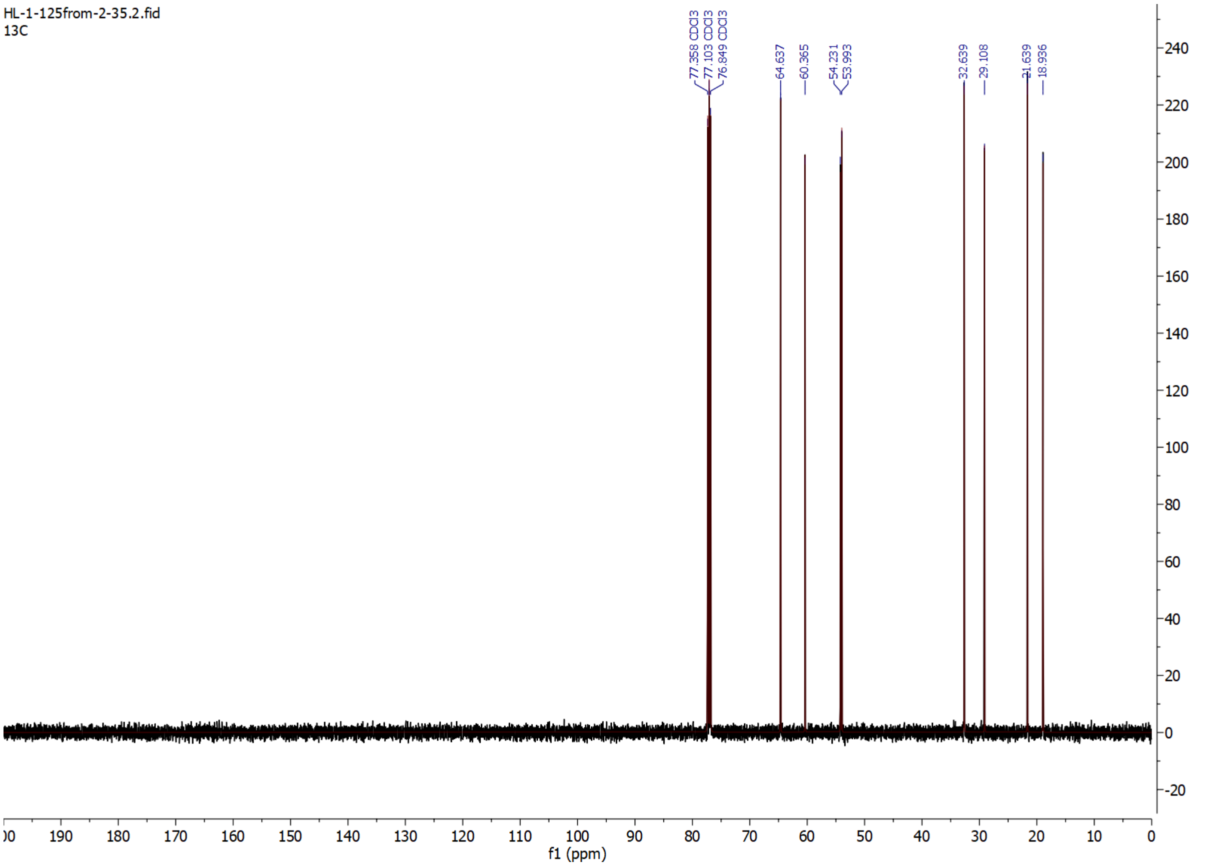

HL-1-125from-2-35.2.fid
13C
77.358 CDCl3
77.103 CDCl3
76.849 CDCl3
64.637
60.365
54.231
53.993
32.639
29.108
21.639
18.936
f1 (ppm)

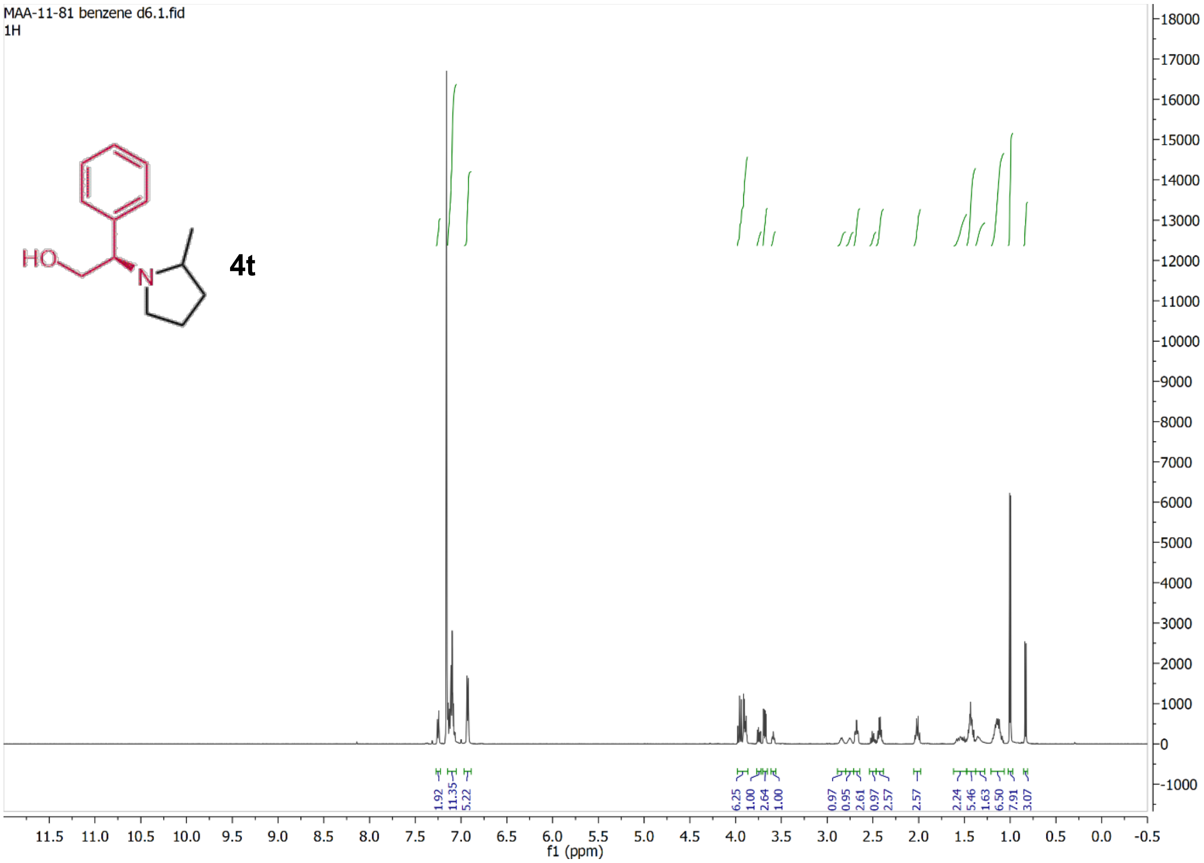

MAA-11-81 benzene d6.1.fid
1H
HO
N
4t
1.92
11.35
5.22
6.25
1.00
2.64
1.00
0.97
0.95
2.61
0.97
2.57
2.57
2.24
5.46
1.63
6.50
7.91
3.07
f1 (ppm)

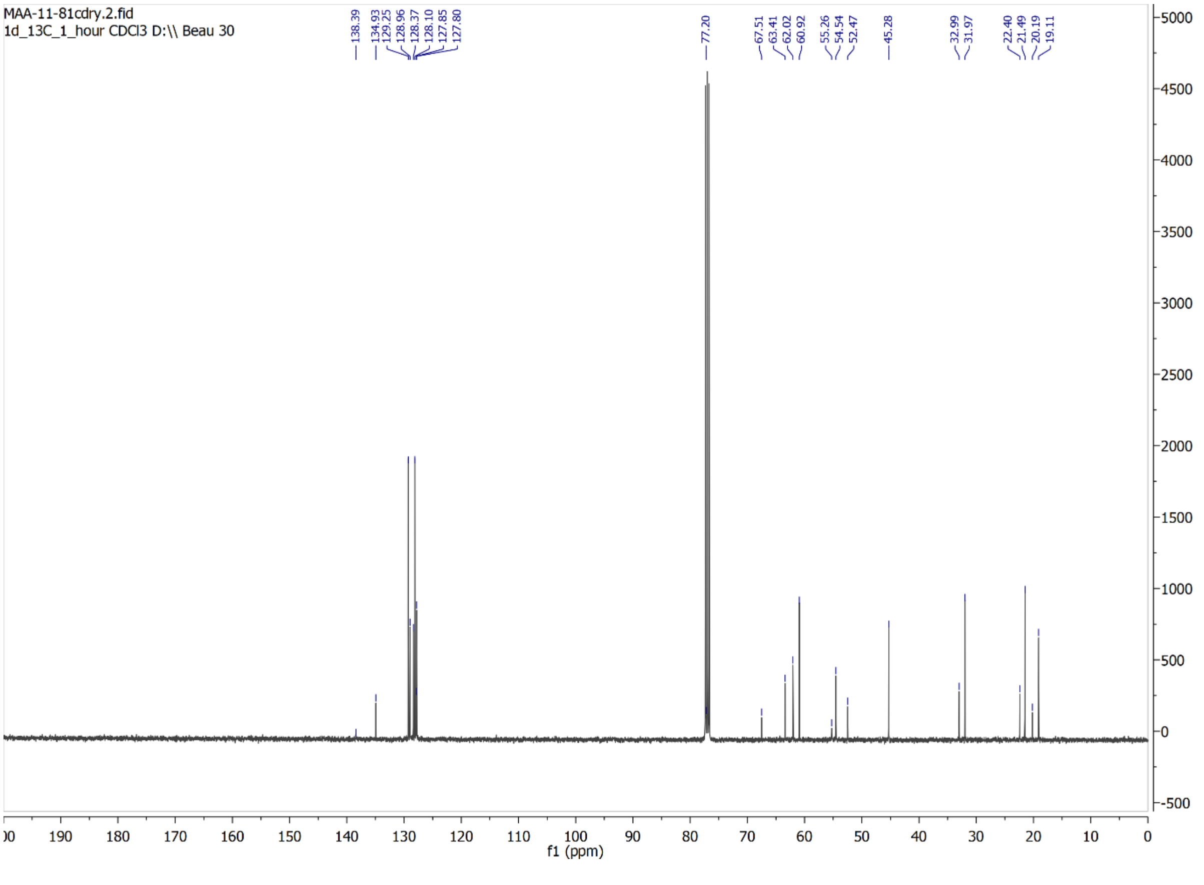

MAA-11-81cdry.2.fid
1d_13C_1_hour CDCl3 D:\\ Beau 30
138.39
134.93
129.25
128.96
128.37
128.10
127.85
127.80
77.20
67.51
63.41
62.02
60.92
55.26
54.54
52.47
45.28
32.99
31.97
22.40
21.49
20.19
19.11
f1 (ppm)

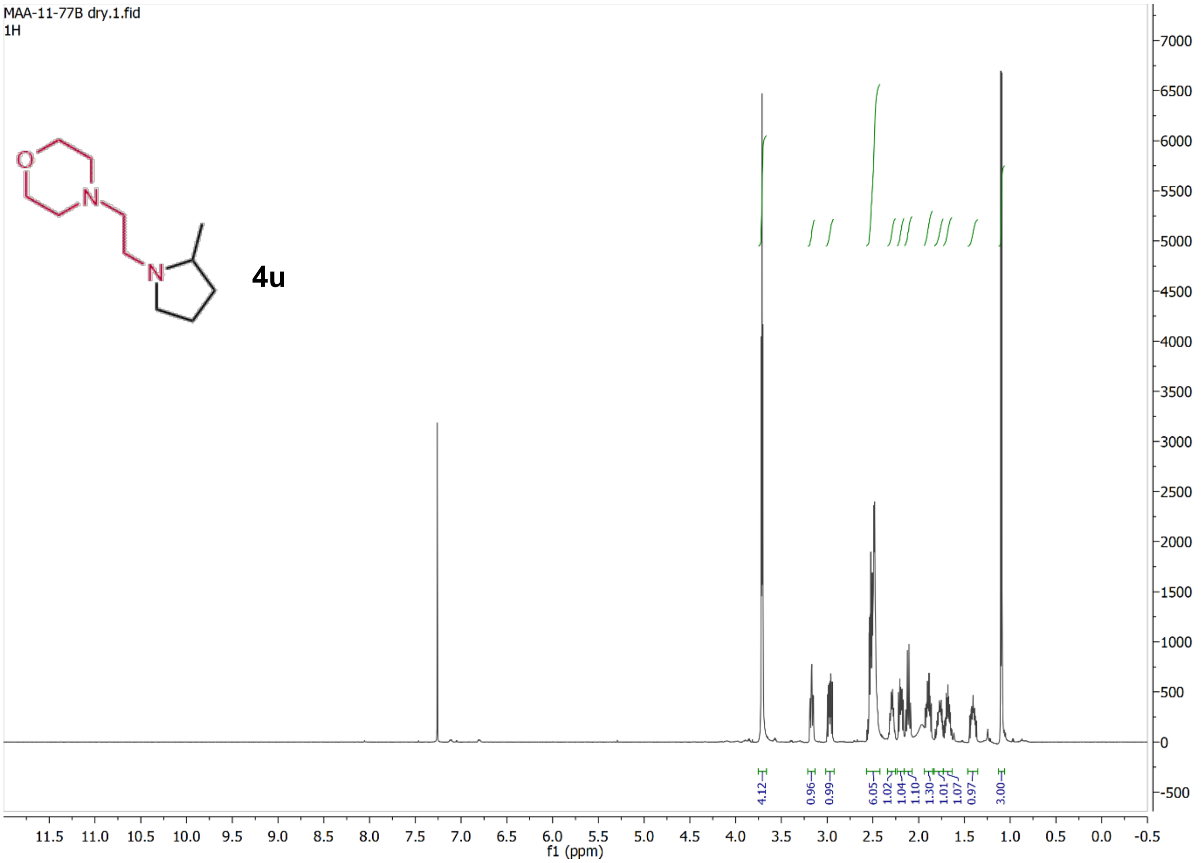
MAA-11-77B dry.1.fid
1H
O
N
N
4u
4.12
0.96
0.99
6.05
1.02
1.04
1.10
1.30
1.01
1.07
0.97
3.00
f1 (ppm)

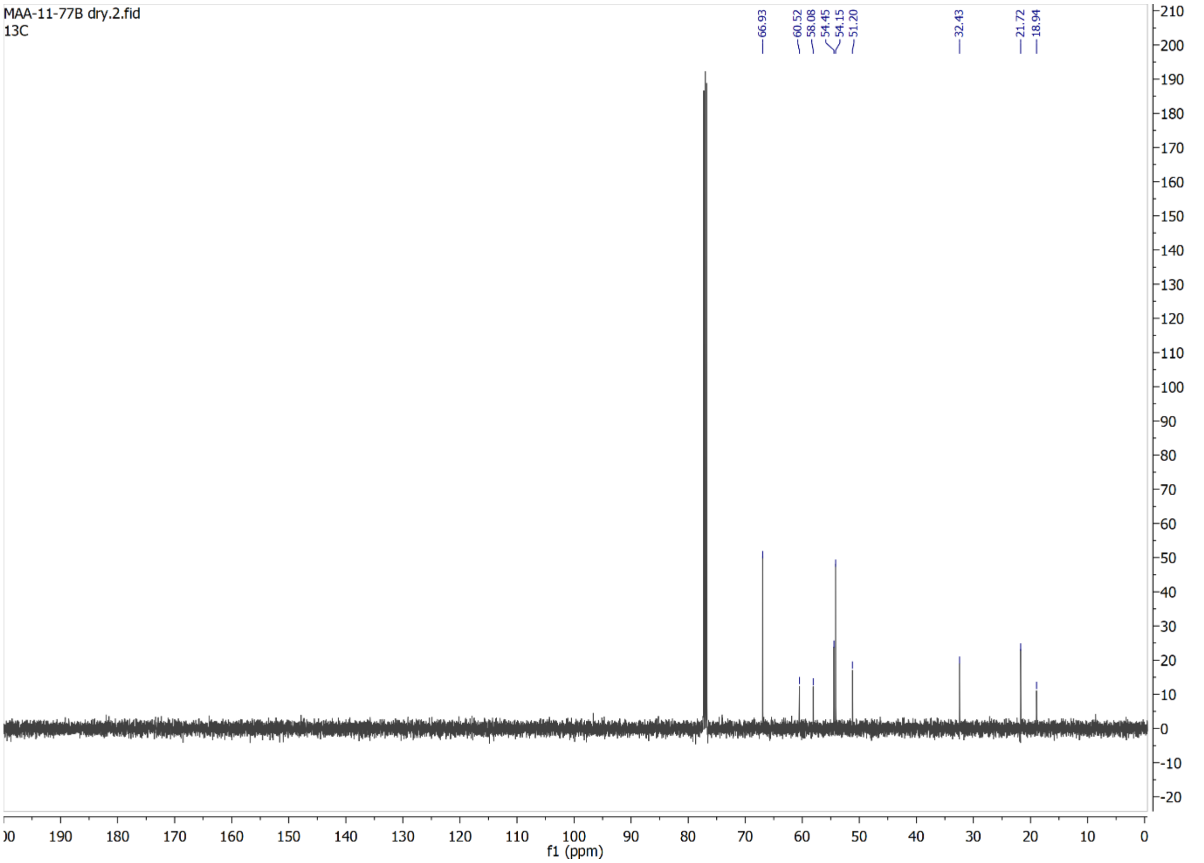
MAA-11-77B dry.2.fid
13C
66.93
60.52
58.08
54.45
54.15
51.20
32.43
21.72
18.94
f1 (ppm)

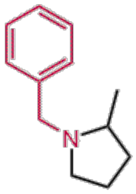

4a
(gram-scale)

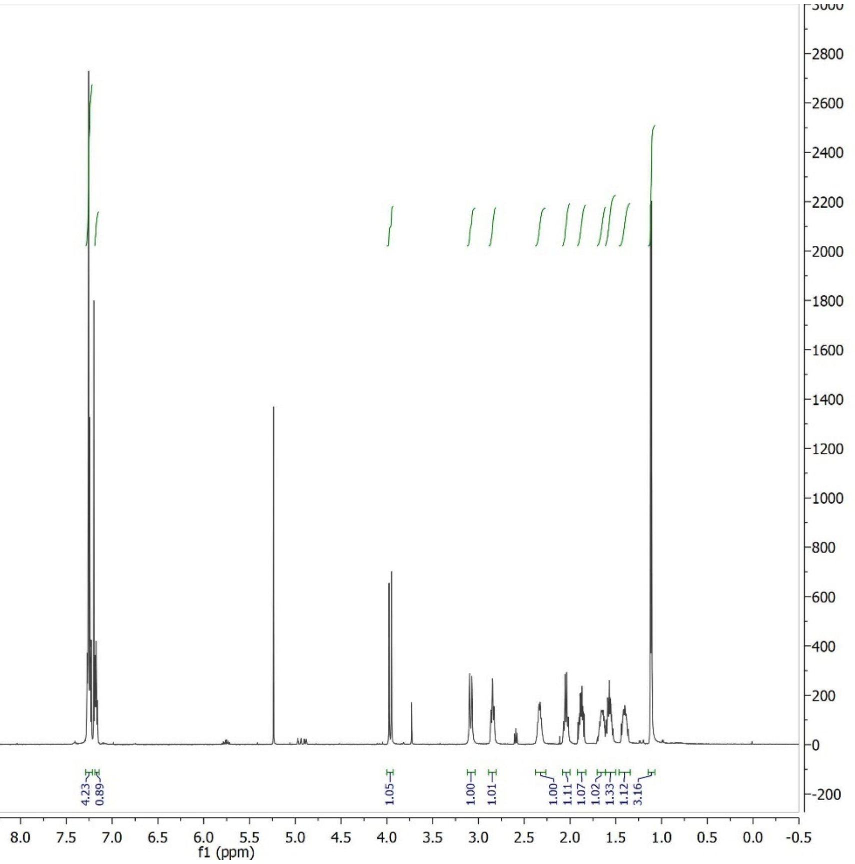

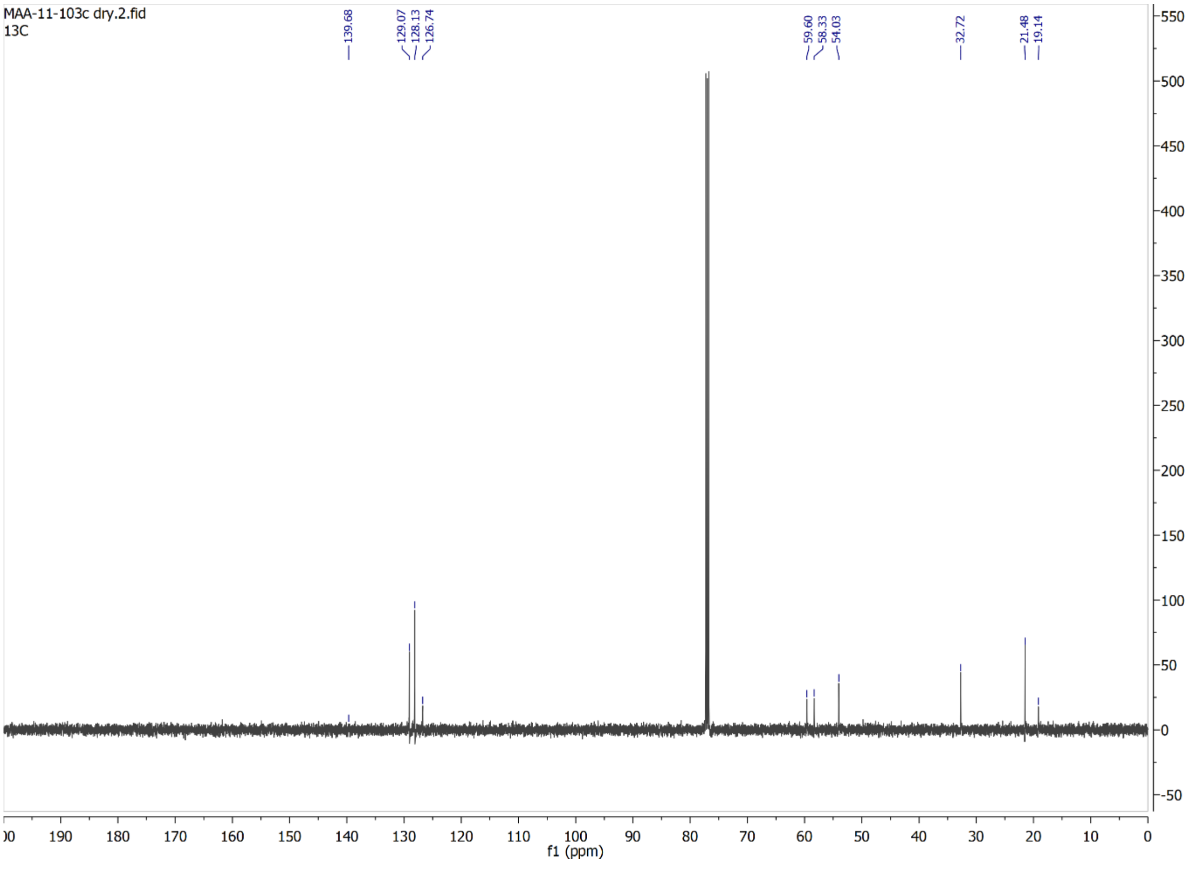

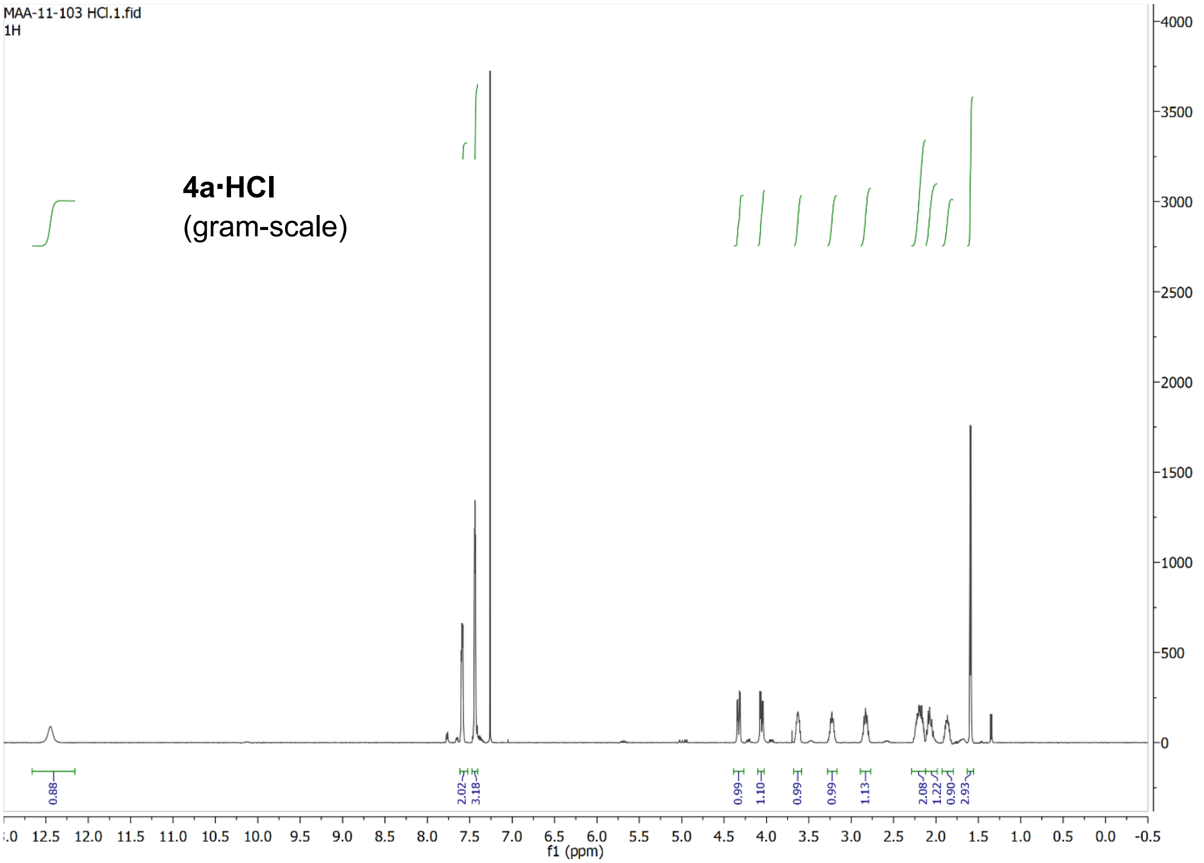
MAA-11-103 HCl.1.fid
1H
4a·HCl
(gram-scale)
f1 (ppm)

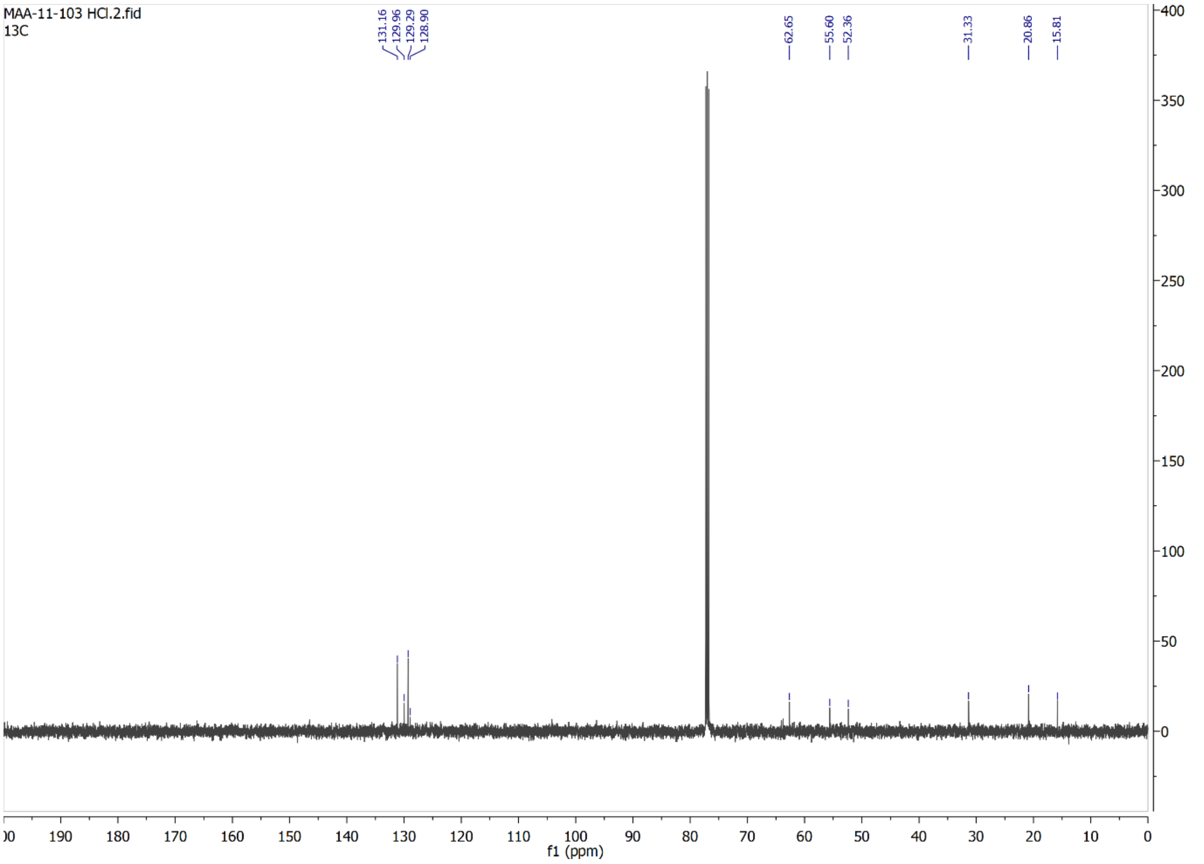
MAA-11-103 HCl.2.fid
13C
131.16
129.96
129.29
128.90
62.65
55.60
52.36
31.33
20.86
15.81
f1 (ppm)
